# Discovery of MDI-114215: A Potent and Selective LIMK Inhibitor To Treat Fragile X Syndrome

**DOI:** 10.1021/acs.jmedchem.4c02694

**Published:** 2024-12-23

**Authors:** Alex G. Baldwin, David W. Foley, Ross Collins, Hyunah Lee, D. Heulyn Jones, Ben Wahab, Loren Waters, Josephine Pedder, Marie Paine, Gui Jie Feng, Lucia Privitera, Alexander Ashall-Kelly, Carys Thomas, Jason A. Gillespie, Lauramariú Schino, Delia Belelli, Cecilia Rocha, Gilles Maussion, Andrea I. Krahn, Thomas M. Durcan, Jonathan M. Elkins, Jeremy J. Lambert, John R. Atack, Simon E. Ward

**Affiliations:** † Medicines Discovery Institute, School of Biosciences, Cardiff University, Main Building, Park Place, Cardiff CF10 3AT, U.K.; ‡ Centre for Medicines Discovery, University of Oxford, Roosevelt Drive, Oxford OX3 7DQ, U.K.; § Division of Neuroscience, School of Medicine, Medical Sciences Institute, Dundee University, Dow Street, Dundee DD1 5HL, U.K.; ∥ The Neuro’s Early Drug Discovery Unit (EDDU), Department of Neurology and Neurosurgery, Montreal Neurological Institute-Hospital, McGill University, 3801 University Street, Montreal, Quebec H3A 2B4, Canada

## Abstract

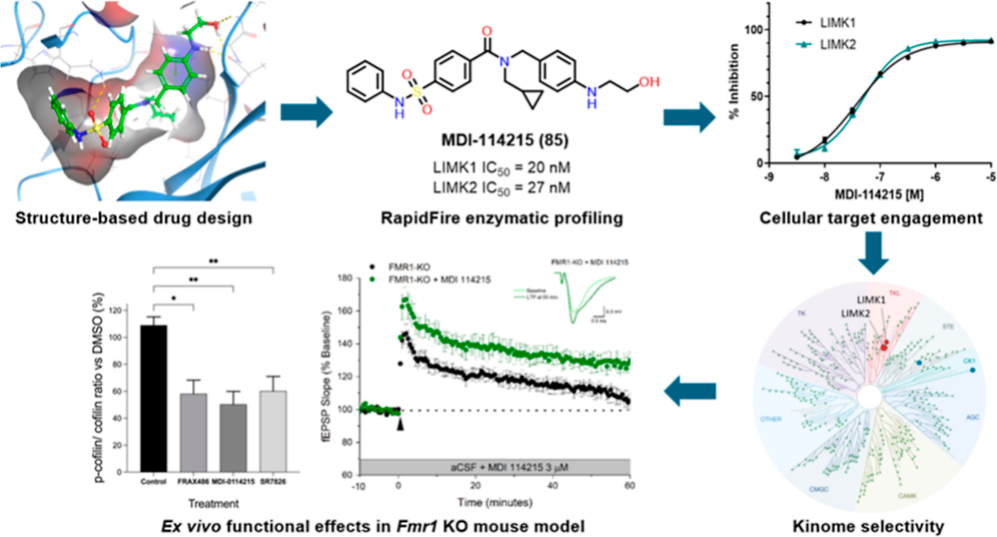

LIMKs are serine/threonine and tyrosine kinases responsible for controlling cytoskeletal dynamics as key regulators of actin stability, ensuring synaptic health through normal synaptic bouton structure and function. However, LIMK1 overactivation results in abnormal dendritic synaptic development that characterizes the pathogenesis of Fragile X Syndrome (FXS). As a result, the development of LIMK inhibitors represents an emerging disease-modifying therapeutic approach for FXS. We report the discovery of MDI-114215 (**85**), a novel, potent allosteric dual-LIMK1/2 inhibitor that demonstrates exquisite kinome selectivity. **85** reduces phospho-cofilin in mouse brain slices and rescues impaired hippocampal long-term potentiation in brain slices from FXS mice. We also show that LIMK inhibitors are effective in reducing phospho-cofilin levels in iPSC neurons derived from FXS patients, demonstrating **85** to be a potential therapeutic candidate for FXS that could have broad application to neurological disorders or cancers caused by LIMK1/2 overactivation and actin instability.

## Introduction

Fragile X Syndrome (FXS) is a neurodevelopmental condition where individuals are characterized by delayed language development and emerging hyperactivity, anxiety and sensory over-reactivity, typically in the second year of life.^1^ Affecting around 1 in 5000 males and 1 in 4–8000 females, FXS is the most common hereditary cause of intellectual disability and autism spectrum disorder (ASD). FXS is caused by absence of the RNA binding protein fragile X mental retardation 1 protein (FMRP) due to trinucleotide repeat expansion of the *FMR1* gene.^1^ Separate groups have shown that loss of FMRP leads to phosphorylation and activation of LIM domain kinase 1 (LIMK1) due to increased levels of full length bone morphogenetic protein type II receptor (BMPR2) which directly interacts with LIMK1^2^ in addition to increased activation of the Rho GTPase Rac1 through its effector p21-activated kinase 1 (PAK1).^3,4^ LIMK1, and the related LIMK2, regulate actin cytoskeletal dynamics by controlling the cellular ratio between filamentous (F) and globular (G) actin through phosphorylation and inactivation of its substrate actin depolymerising factor (ADF)/cofilin family of proteins (collectively referred to as cofilin).^5−7^ Inappropriate LIMK1 activation causes an imbalance in F/G-actin ratio where F-actin accumulates, causing abnormal synaptic and dendritic spine morphology which can be observed in the well-established *Fmr*1 KO mouse model^2,3^ and *Drosophila* model of FXS.^8^ Importantly, these changes are consistent with observations in FXS individuals in whom there is increased LIMK1 activity as measured either by the extent of p-LIMK1 or p-cofilin in post-mortem brain tissue^2^ and an abnormally high density of dendritic spines in cortical neurons,^9^ leading to defects in synaptic plasticity which underlie the clinical symptoms.^10^ Pharmacological inhibitors or genetic reduction of signaling through the BMPR and Rac1-PAK1 pathways rescues many of the phenotypes associated with FXS mouse and fly models.^2,3,8,11,12^ Given its critical role as a downstream point of convergence of both the BMPR2 and Rac1-PAK1 signaling pathways, selective LIMK1 inhibitors are desirable to reduce LIMK1-mediated cofilin phosphorylation and correct actin instability that results in abnormal dendritic spine morphology and synaptic function characteristic of FXS.

Several LIMK1/2 inhibitors have been previously reported,^13,14^ the most studied of which is the thiazole derivative LIMKi3 (also called BMS-5, **1**). Developed by Bristol-Myers Squibb, LIMKi3 is highly potent for LIMK1 (IC_50_ = 6 nM) and LIMK2 (IC_50_ = 33 nM) in inhibiting cofilin phosphorylation.^14,15^ LIMKi3 treatment reverses abnormal dendritic spine morphology, restores the number of immature spines to normal levels in cortical and hippocampal neurons^2^ and normalizes anxiety-related behavior in the *Fmr1* KO mouse model.^8^ However, LIMKi3 has not been progressed further, presumably due to its nonkinase cytotoxic effects on microtubule depolymerization.^15^ We previously showed that FRAX486 (**2**), a potent group I PAK inhibitor (PAK1 IC_50_ = 8 nM) and clinical candidate for FXS, also strongly inhibits both LIMK1 and LIMK2 (IC_50_ = 7 nM and 13 nM, respectively).^14^ FRAX486 has been shown to restore abnormal synaptic morphology and impaired sensory processing in addition to rescuing seizure and behavioral abnormalities in *Fmr1* KO mice by significantly reducing elevated p-LIMK1 levels and normalizing the F/G-actin ratio.^3,12^ Although FRAX486 is a brain-penetrant molecule, supporting its use in CNS indications, it demonstrates poor selectivity across a large kinase panel and has known dual LIMK/PAK inhibition,^14^ limiting the use of FRAX486 as a suitable tool compound to study mechanisms behind LIMK1/2 pathology. Lexicon Pharmaceuticals disclosed two programmes developing potent LIMK inhibitors: an allosteric type III aryl sulfonamide series showing exquisite kinome selectivity^16^ and a pyrrolopyrimidine series as dual LIMK/ROCK (Rho kinase) inhibitors that led to the clinical candidate LX7101 (**3**, LIMK1/2 IC_50_ = 32 nM and 4.3 nM, respectively) being progressed into phase I/2a clinical trials for the treatment of intraocular pressure in glaucoma.^17,18^ LX7101 has not been evaluated for FXS but given that LIMK1/2 activity can also be switched on by the upstream kinase ROCK1 and ROCK2,^7,19,20^ in vivo efficacy in FXS models would be difficult to attribute to either LIMK or ROCK inhibition, as highlighted previously.^17,21,22^ An LX7101 analogue devoid of ROCK1/2 inhibition, SR7826 (**4**), demonstrates high LIMK1 potency (IC_50_ = 43 nM)^23^ and rescues hippocampal thin spine loss and neuronal hyperexcitability in human amyloid precursor protein (hAPP) mice by protecting against amyloid-beta (Aβ)-induced dendritic spine degeneration.^21^ Nevertheless, we have determined that SR7826 and LX7101 have promiscuous kinase selectivity with a significant number of off-targets.^14^ Additionally, there are polycyclic molecules such as pyridocarbazole Pyr1 (**5**), a potent dual LIMK1/2 inhibitor (IC_50_ = 50 and 75 nM, respectively)^24^ that normalizes dendritic spine density in vitro and in vivo and improves long-term hippocampal synaptic transmission and plasticity in a schizophrenia mouse model.^25^ However, its reported high selectivity is based only on a limited panel of 110 kinases (approximately 20% of the human kinome) and despite progressing to preclinical trials for schizophrenia, no further developments have been reported. Other LIMK1 inhibitors are less well characterized^13^ and given there are no drugs specifically approved for the treatment of FXS, there is a significant unmet clinical need to develop selective LIMK inhibitors.

Here, we describe the discovery, proof of mechanism (by reducing p-cofilin levels in neuronal cells in a concentration-dependent manner) and preclinical efficacy of MDI-114215 (**85**), a highly selective dual LIMK1/2 inhibitor that is well tolerated and demonstrates proof-of-concept in the *Fmr1* KO mouse brain slice electrophysiology assay. These novel LIMK inhibitors significantly decrease p-cofilin in *Fmr1* KO mice and in stem cell-derived cortical neurons from FXS patients, thereby **85** represents a superior tool compound in vitro and in vivo to explore LIMK biology in addition to potential treatment of LIMK pathologies such as FXS.

## Results and Discussion

### Structure-Based Drug Design of Improved LIMK Inhibitors

**Figure 1:**
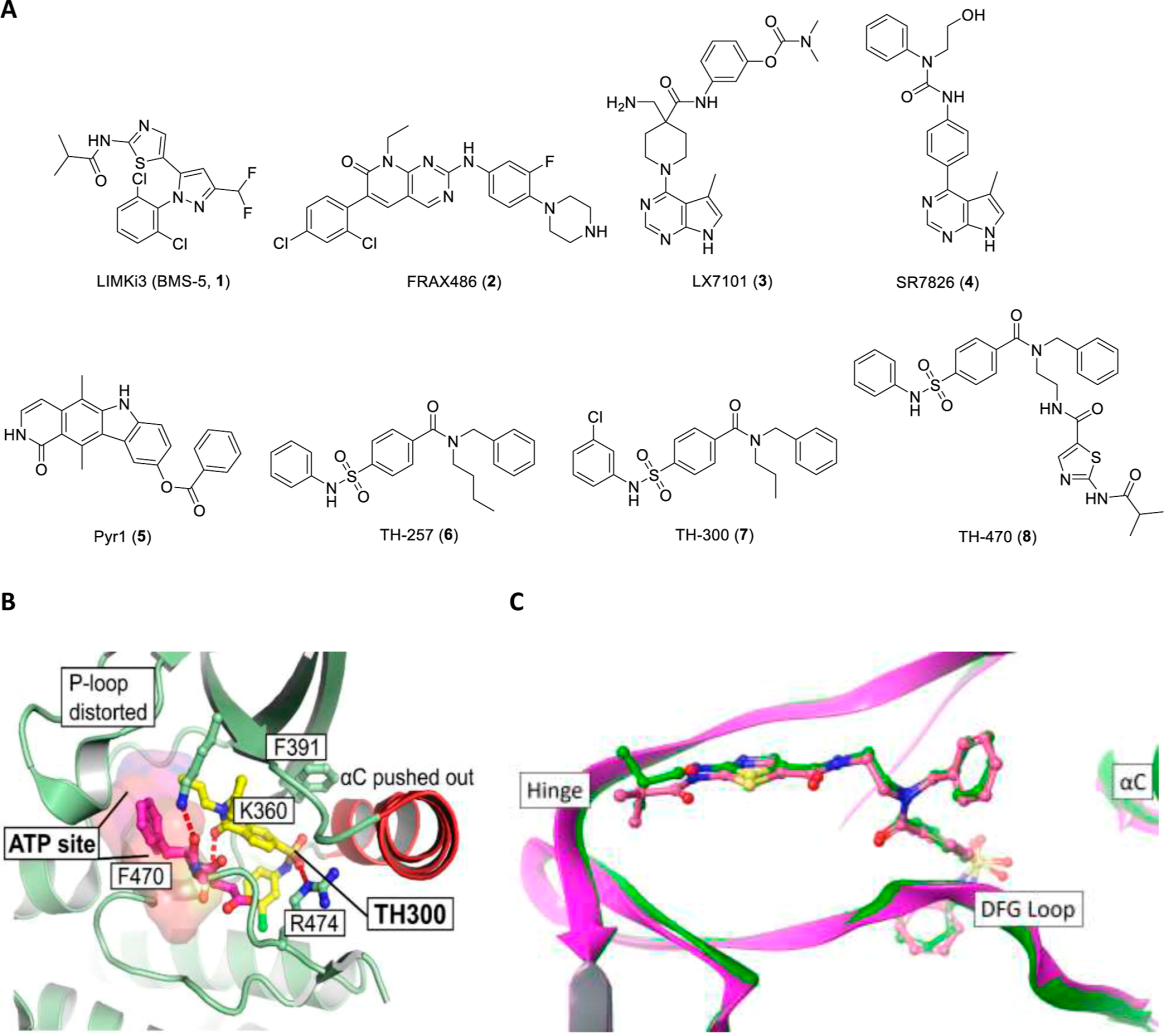
(a) Chemical structures of selected LIMK1/2 inhibitors. (b) Co-crystal structure of the allosteric inhibitor **7** (TH-300) bound to LIMK2 (PDB: 5NXD); (c) type II inhibitor **8** (TH-470) bound to LIMK1 (PDB: 7B8W, green) and LIMK2 (PDB:7QHG, magenta).

We selected TH-257 (**6**, Figure 1A), a type III (allosteric) kinase inhibitor^26^ that is structurally related to the aryl sulfonamide series disclosed by Lexicon Pharmaceuticals^16^ as a suitable starting point for developing more potent LIMK inhibitors on the basis of its moderate LIMK1/2 inhibition (LIMK1 IC_50_ = 200 nM, LIMK2 IC_50_ = 14 nM)^14^ and excellent kinome selectivity profile. The known challenges with this series included poor aqueous solubility and rapid in vitro microsomal turnover; key developability issues that limited in vivo proof-of-concept evaluation of these compounds.

**Scheme 1:**
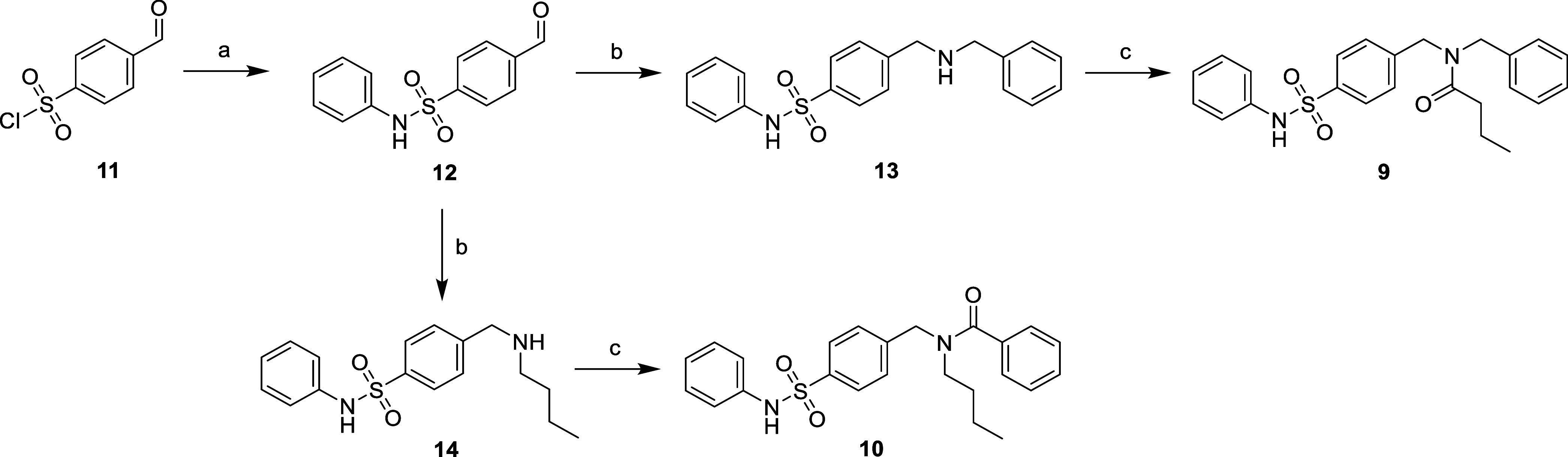
Synthesis of Tertiary Amide Isomers **9** and **10** Reagents and conditions: (a) PhNH_2_, Py, DCM, rt, 3 h, 41%; (b) BnNH_2_ or BuNH_2_, NaHCO_3_, MeOH, rt, 18 h then NaBH_4_, 0 °C, 4 h; (c) butyric acid or benzoic acid, HOBt, EDC.HCl, DCM, rt, 30 min then **13** or **14**, rt, 18 h, 38–62% (over two steps).

**Scheme 2:**
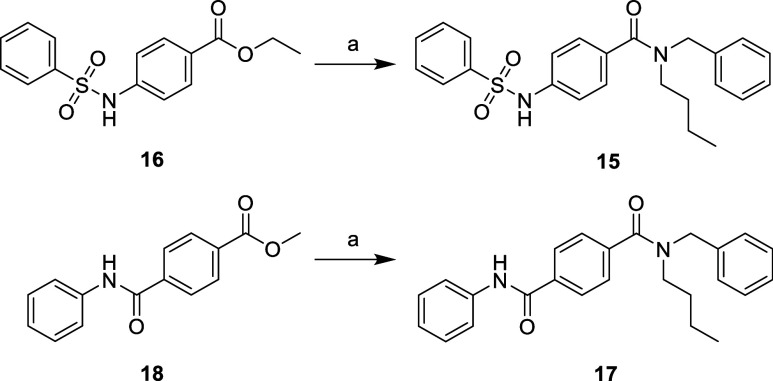
Synthesis of Reverse Sulfonamide **15** and Amide Analogue **17** Reagents and conditions: (a) *N*-benzylbutylamine, AlEt_3_, 1,2-DCE, 0–80 °C, 18 h, 62–81%.

**Scheme 3:**
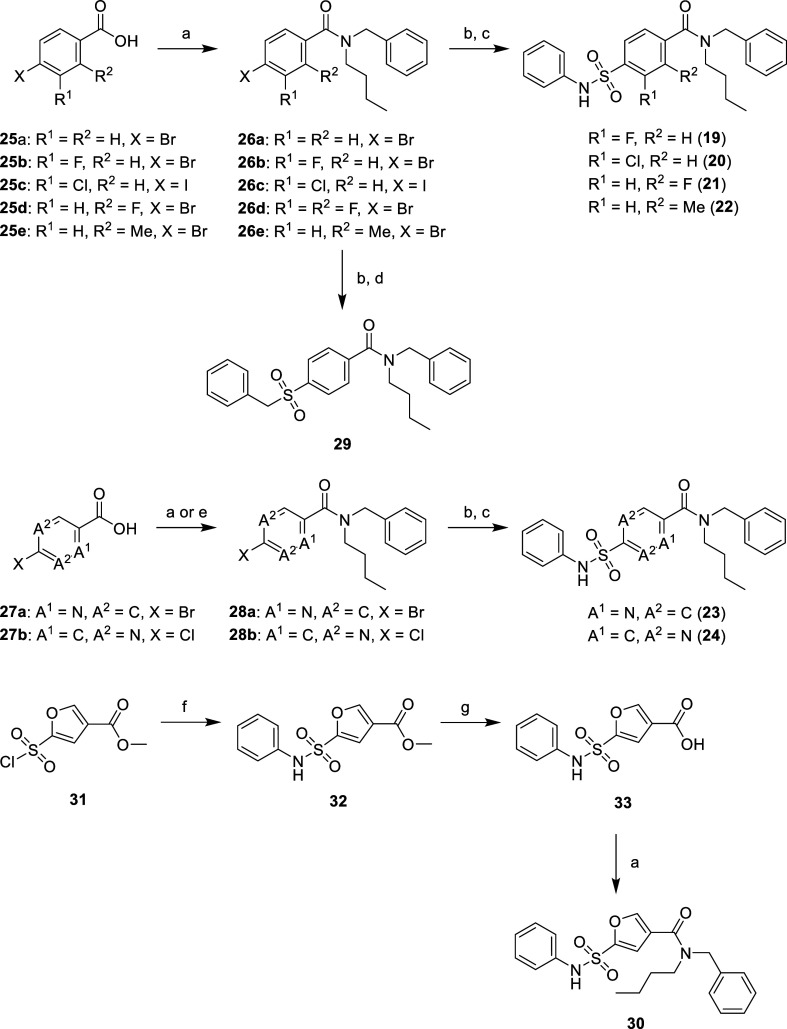
Synthesis of Sulfone Derivative **29** and Aromatic Core Analogues **19–24** and **30** Reagents and conditions: (a) HOBt, EDC.HCl, DCM, rt, 30 min then *N*-benzylbutylamine, rt, 18 h, 78–97%; (b) **26a**, K_2_S_2_O_5_, NaHCO_2_, Pd(OAc)_2_, PPh_3_, phen, TBAB, DMSO, 70 °C, 3 h; (c) PhNH_2_, NCS, THF, 0 °C, 2 h, 7–24% (over two steps); (d) benzyl bromide, rt, 18 h, 62% (over two steps); (e) **27b**, *N*-benzylbutylamine, T3P, Et_3_N, DMF, rt, 1 h, 33%; (f) PhNH_2_, THF, rt, 18 h, 66%; (g) LiOH, H_2_O/MeOH/THF, rt, 18 h, 95%.

**Scheme 4:**
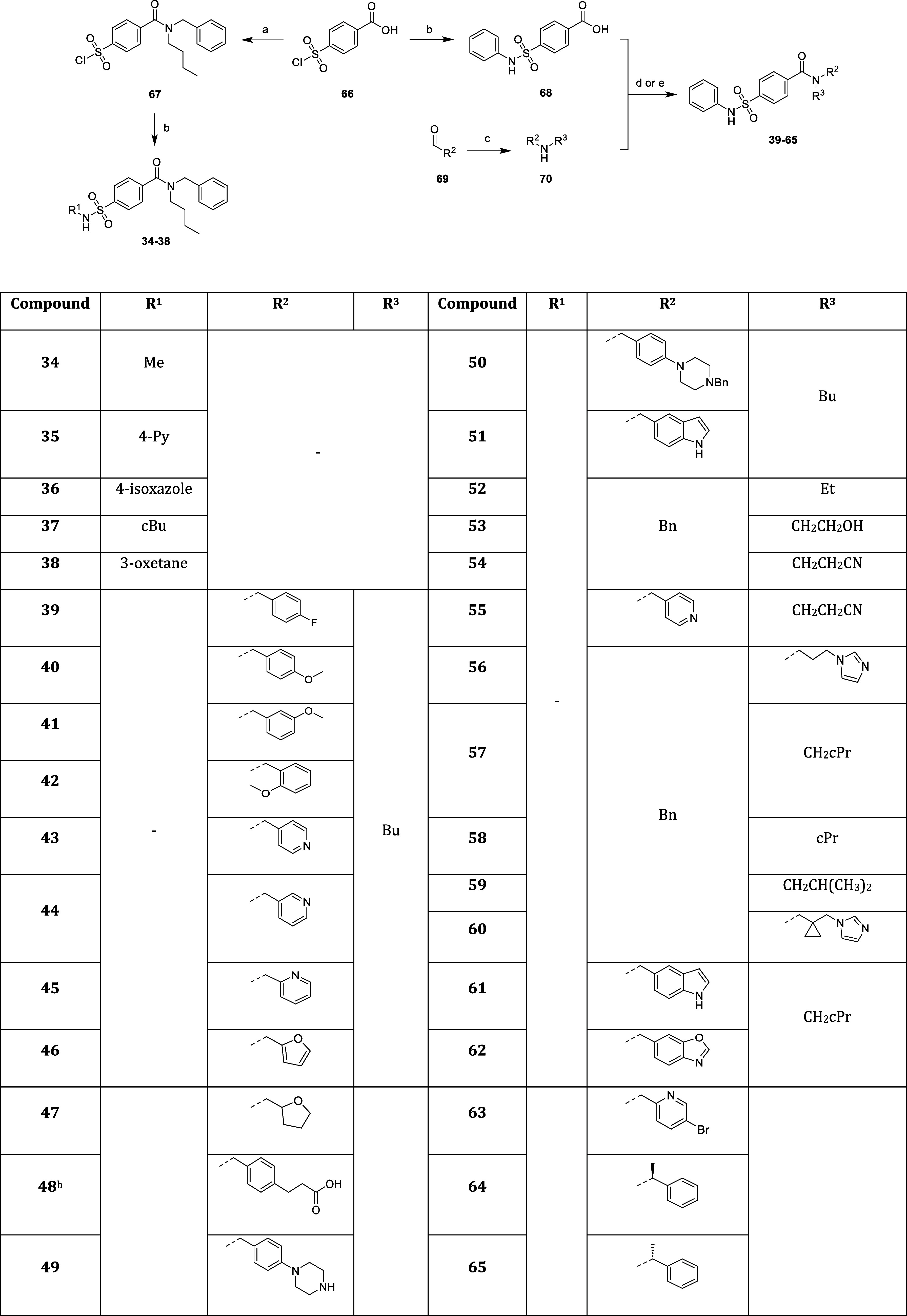
Synthesis of Substituted *N*-Phenylsulfonamide Analogues **34–38** and *N*-Benzylbutylamide Analogues **39–65** Reagents and conditions: (a) SOCl_2_, DMF (cat.), 70 °C, 3 h then *N*-benzylbutylamine, Et_3_N, THF, −78 °C, 30 min, 77%; (b) R^1^NH_2_, THF, rt, 18 h, 17–78%; (c) R^3^NH_2_, NaHCO_3_, MeOH, rt, 18 h then NaBH_4_, 0 °C, 1–4 h; (d) HOBt, EDC.HCl, DCM, rt, 30 min then **70**, rt, 18 h, 5–97% (over two steps); (e) (COCl)_2_, DMF (cat.), DCM, rt, 5 h then **70**, Et_3_N, 0 °C, 1 h, 56–77% (over two steps). Synthesized from methyl 3-(4-((butylamino)methyl)phenyl)propanoate precursor over four steps.

An X-ray crystal structure of a close analogue, TH-300 (**7**, Figure 1A), has been reported in complex with LIMK2 (PDB: 5NXD).^26^ Compound **7** binds in a narrow lipophilic back pocket with four key ligand–protein contacts: (i) the sulfonamide N–H interacts with L389 via a water bridge, (ii) the sulfonamide O anchors the ligand by forming a hydrogen bond with R474 adjacent to the DFG motif on the activation loop, (iii) the backbone N–H of D469 of the DFG motif participates in an interaction with the carbonyl of the amide, and (iv) a π-cation interaction is formed between the *N*-benzylamide moiety and the protonated K360 (Figure 1B). To confirm the importance of these interactions, we made chemical modifications to compound **6** that alter: (i) the position of the carbonyl amide onto the benzyl or butyl vectors (Table S1, Scheme 1), (ii) changing the sulfonamide (Table S2, Schemes 2 and 3), (iii) *N*-phenylsulfonamide replacements (Table S3, Scheme 4), or (iv) core phenyl ring substitutions or heteroaryl replacements (Table S4, Scheme 3).

Tertiary amide isomers **9** and **10** were synthesized from aldehyde **12** through reductive amination to afford intermediates **13** and **14** that were subsequently subjected to amide coupling (Scheme 1). Compounds **15** and **17** were directly synthesized from esters **16** and **18** using triethylaluminum (AlEt_3_)-mediated amide coupling with *N*-benzylbutylamine in good yields (Scheme 2). Core phenyl substituted derivatives **19**–**22** and six-membered heterocyclic replacements **23**–**24** were synthesized from the corresponding 4-halo-(hetero)aryl carboxylic acid using a two-step, one-pot palladium-catalyzed sulfination and chlorination procedure (Scheme 3).^27^ We adapted the reported procedure using *N*-chlorosuccinimide (NCS) rather than *N*-bromosuccinimide (NBS) as we found sulfonyl chlorides gave greater conversion to the desired sulfonamide derivative relative to undesired sulfonic acid side-product when compared to sulfonyl bromides, owing to its reduced hydrolytic susceptibility. When 2-chloropyrimidine **27b** was subjected to amide coupling under HOBt/EDC coupling conditions, only the bis-amine adduct was formed due to a competing S_N_Ar reaction. An alternative amide coupling method using T3P successfully formed desired product **28b** in satisfactory yield to synthesize pyrimidine analogue **24**. Sulfone derivative **29** was synthesized using the same procedure as previously described^27^ from the sulfinic acid of unsubstituted intermediate **26a** and benzyl bromide in good yield. Five-membered core heterocyclic replacements, exemplified by **30**, were synthesized directly from the commercially available (chlorosulfonyl)aryl carboxylic acid through a facile three-step method involving sulfonamide coupling to give ester **32**, LiOH-mediated hydrolysis to afford acid **33** and subsequent amide coupling (Scheme 3). Substituted *N*-phenylsulfonamides **34**–**38** were synthesized using common intermediate **67**, derived from starting material **66** through SOCl_2_-mediated bis-acid chloride formation and regioselective amide coupling with *N*-benzylbutylamine at −78 °C (Scheme 4).

Our initial SAR assessment around **6** confirmed the importance of these interactions and narrow pocket within LIMK2, as all of these modifications caused significant decreases in LIMK1/2 inhibition (Tables S1–S4). The ligand therefore binds away from the hinge region of LIMK1/2 through a unique binding mode resulting from distortion of the P-loop, outward displacement of the αC helix and the DFG motif adopts the DFG-out conformation, thus conferring the exquisite selectivity consistent with an allosteric, type III inhibitor. This binding mode is also consistent with a related compound in complex with LIMK2 previously reported by Lexicon Pharmaceuticals (PDB: 4TPT).^16^

A cocrystal structure of the type II inhibitor TH-470 (**8**, Figure 1A), a fusion of **6** with the aminothiazole hinge binding moiety from **1**, with LIMK1 further supports this binding mode. **8** is a highly potent LIMK1/2 inhibitor (LIMK1 IC_50_ = 6 nM, LIMK2 IC_50_ = 5 nM).^14,26^ We solved the cocrystal structure of **8** in complex with LIMK1 (PDB: 7B8W), expectedly showing that **8** binds both the hinge via the thiazole N and pendant amide N–H with I416 in addition to the allosteric DFG-out pocket through similar interactions observed in the TH-300-LIMK2 structure. The short amide linker permits a type II inhibitor through the short linker amide N–H interaction with gatekeeper residue T413. A comparison of the cocrystal structures of **8** with LIMK1 and LIMK2 (PDB: 7QHG)^26^ showed a very similar binding mode (Figure 1C), consistent with the 90% sequence homology within 10 Å of the active site between LIMK1 and LIMK2. Contrary to Hanke et al., we did not observe increased solubility and metabolic stability for **8** with respect to **6**(14) and given its lower kinome selectivity in the KINOMEscan panel,^26^ we decided to undertake a structure-based drug design approach based on our cocrystal structure and a LIMK1 homology model based on the TH-300-LIMK2 structure to synthesize more potent, selective dual LIMK1/2 inhibitors that have improved DMPK properties suitable for in vivo evaluation in the *Fmr1* KO FXS model.

In light of our extensive structure–activity relationship (SAR) assessment of the *N*-phenylsulfonamide moiety, we turned our attention to the tertiary amide of **6**, encouraged by previous SAR demonstrating greater scope for modifications at the butyl and *N*-benzylamide vectors.^16^ Substituted *N*-benzylbutylamides **39**–**65** were synthesized through reductive amination of aldehyde derivatives **69** with the corresponding primary amine and amide coupling of key intermediate acid **68** with secondary amines **70** (Scheme 4). Although nonsterically hindered amines were amenable to amide coupling using HOBt/EDC as previously reported,^14,16,26^ sterically hindered benzylbutylamine analogues (in particular *N*-benzylcyclopropylamines) gave little to no desired product under these conditions. Therefore, bulky amines were subjected to amide coupling using preformed 4-(phenylsulfamoyl)benzoyl chloride. Carboxylic acid **48** was synthesized in a four step method involving: (i) reductive amination of methyl (2*E*)-3-(4-formylphenyl)prop-2-enoate and *N*-butylamine, (ii) alkene reduction using Et_3_SiH and Pd–C, (iii) amide coupling with **68**, and (iv) ester hydrolysis (see Experimental Section for details). Briefly, there was scope for a variety of elongated, neutral or weakly basic groups at either the para position of the *N*-benzyl ring or *N*-butyl vector (Tables S5 and S6). Notable analogues that improved microsomal CL_int_ include the 3,4-bridged bicyclic heterocycle **51** (LIMK1 pIC_50_ = 6.86, LIMK2 pIC_50_ = 7.68) and pyridyl compound **55** (LIMK1 pIC_50_ = 5.33, LIMK2 pIC_50_ = 7.07, Table S6), the latter demonstrating >50-fold selectivity for LIMK2 over LIMK1. Importantly, we discovered that the *N*-butyl to methylene cyclopropylmethyl (CH_2_cPr) switch led to a significant and consistent ∼10-fold increase in LIMK1 potency in the RapidFire assay (Table S6), exemplified by parent **57** (Table 1). Removing the methylene linker (**58**) or ring opening to the *iso*-butyl analogue **59** led to a nearly 30-fold drop in LIMK1 inhibition (LIMK pIC_50_ = 5.97 and 5.99, respectively, Table S6) compared to parent **57**. Therefore, we hypothesized that the pseudoaromaticity and better space-filling of the cyclopropyl ring was essential for potent inhibition. A further compound array around **57** highlighted several interesting compounds, particularly 5-(pyridin-2-yl) substituted analogues such as **63** (LIMK1 pIC_50_ = 7.72, LIMK2 pIC_50_ = 7.59, Table S6) that were now tolerated in contrast to the TH-like series (Table S5).

**Table 1:**
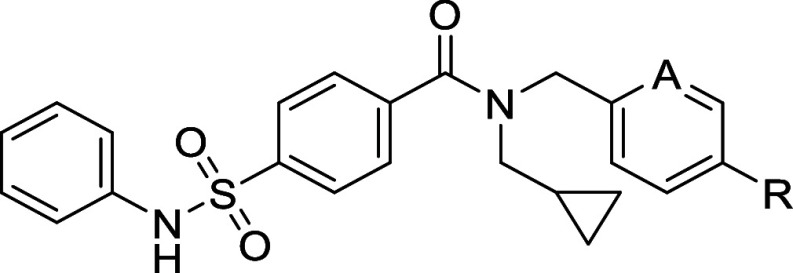

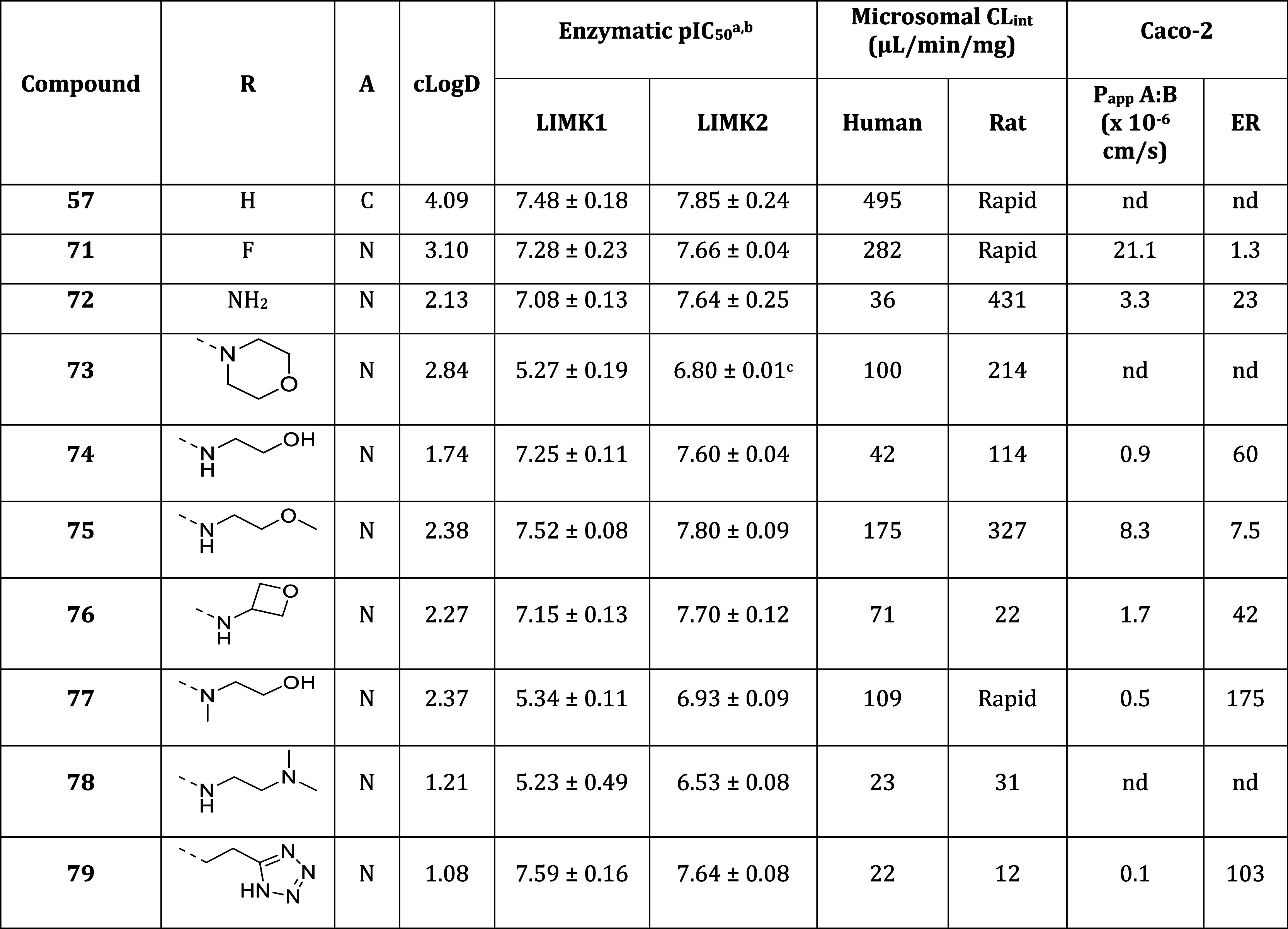
LIMK1/2 Inhibitory Activities of **57** and 5-Substituted-*N*-pyridyl Analogues **71–79**^d^

a The phosphorylation of cofilin was assessed by mass spectrometry following an enzymatic assay.

b pIC50 is the negative logarithm of the IC_50_ value.

c Mean of two independent experiments. nd, not determined.

d Data are reported as mean ± SEM of at least 3 independent experiments.

At this stage, we analyzed structure–clearance relationships by plotting microsomal CL_int_ against *c*log*D* (Figure S1) to understand potential sites of metabolism as we were unable to combine high LIMK1/2 potency (IC_50_ ≤ 30 nM) and high metabolic stability (HLM/RLM = ≤100 μL/min/mg) in a single molecule. Generally, microsomal stability was greater in HLM than RLM. Compounds with *c* log *D* ≤ 2 had low-moderate microsomal CL_int_ but these were largely populated with highly basic molecules devoid of LIMK1/2 potency, except for the potent carboxylic acid **48** (LIMK1 pIC_50_ = 6.78, LIMK2 pIC_50_ = 7.68, Table S5). Compounds with *c* log *D* > 2 had high and variable metabolism, however a subset of structurally similar 4-substituted *N*-benzyl analogues of **6** that clustered together had surprisingly low-moderate microsomal CL_int_ despite high lipophilicity (Figure S1), exemplified by **50** (Table S5). Additionally, the *para* benzyl vector points toward a solvent-exposed region in the LIMK1 homology model and **8**-LIMK1 cocrystal structure (Figure 1B,C). Thus, we reasoned that the para position of the benzyl ring was a major site of metabolism and blocking this position with a water-solubilizing pendant while incorporating the CH_2_cPr group would lead to a highly potent LIMK1/2 inhibitor with significantly improved metabolic stability.

### Lead Optimization of MDI-65658 and Discovery of MDI-114215

**Scheme 5:**
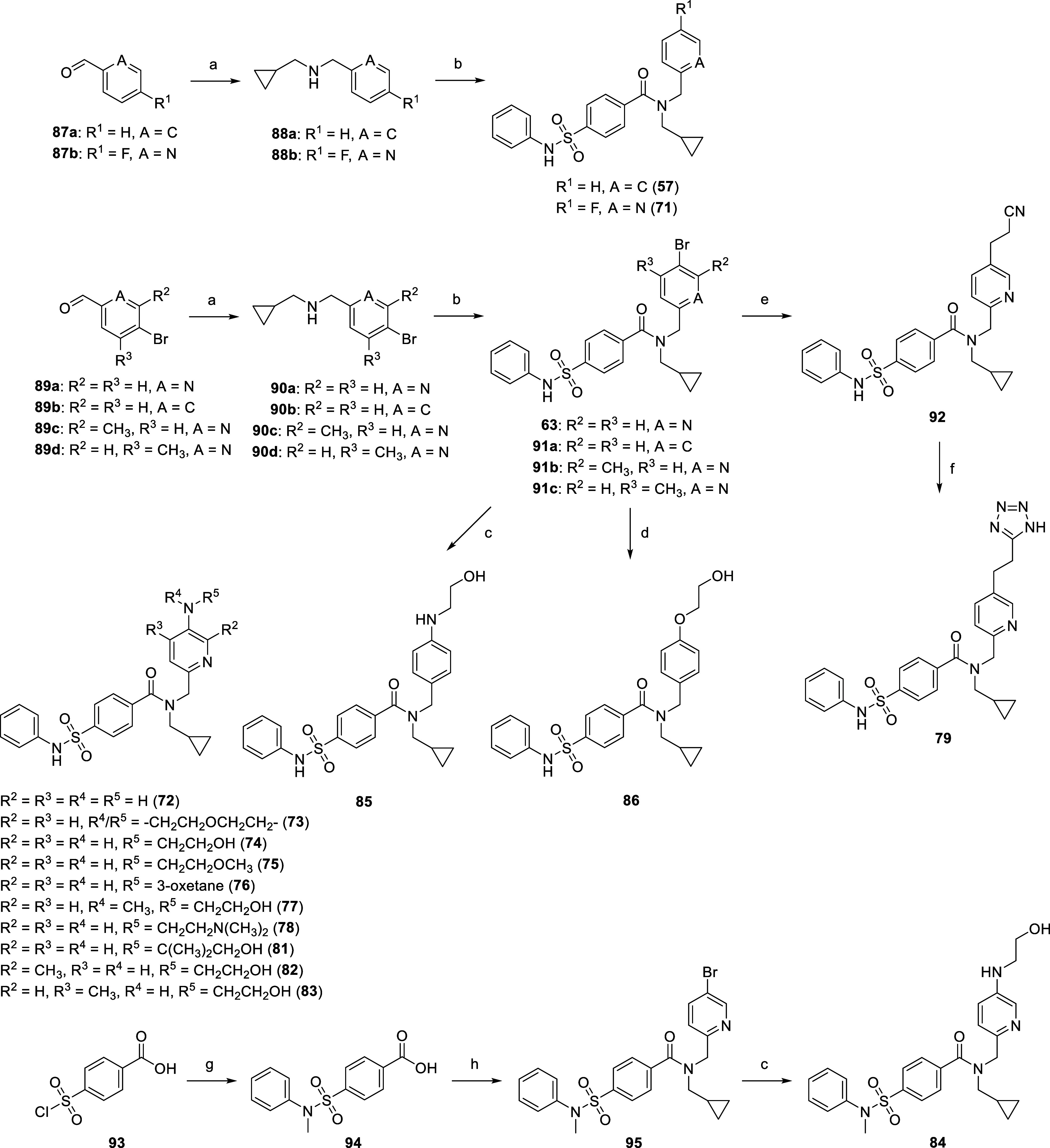
Synthesis of MDI-65658 (**74**), MDI-114215 (**85**) and Their Derivatives Reagents and conditions: (a) CPrCH_2_NH_2_, NaHCO_3_, MeOH, rt, 18 h then NaBH_4_, 0 °C, 1 h; (b) 4-(phenylsulfamoyl)benzoyl chloride, Et_3_N, 0 °C, 1 h, 54–77% (over two steps); (c) R^4^NHR^5^, CuI, l-proline, K_2_CO_3_, DMSO, 80–100 °C, 18 h, 10–51%; (d) LiO^*t*^Bu, ethylene glycol, rt, 5 min then **91a**, CuI, 110 °C, 18 h, 50%; (e) acrylonitrile, Pd(OAc)_2_, NaHCO_3_, TBAB, DMF, 110 °C, 4 h then Et_3_SiH, Pd–C, MeOH, rt, 24 h, 39%; (f) NaN_3_, NH_4_Cl, DMF, 120 °C, 18 h, 63%; (g) PhNHMe, THF, rt, 18 h, 57%; (h) (COCl)_2_, DMF (cat.), DCM, rt, 5 h then **90a**, Et_3_N, 0 °C, 1 h, 21% (over two steps).

Using **63** (Scheme 4) as a suitable building block, we synthesized MDI-65658 (**74**), MDI-114215 (**85**) and its water-solubilizing analogues at the 5-pyridyl position through a three-step synthesis (Scheme 5). The first two steps involved reductive amination with aldehyde **89a**–**d**, cyclopropylmethylamine and NaBH_4_, followed by amide coupling using 4-(phenylsulfamoyl)benzoyl chloride, derived from acid **68** (Scheme 4). Acid chloride formation using SOCl_2_ at reflux consistently led to sulfonamide hydrolysis, therefore milder conditions using oxalyl chloride (COCl)_2_ and DMF catalyst were employed. Cu(I)-catalyzed nucleophilic aromatic substitution of **63** or intermediates **91a**–**c** with polar amines and ethylene glycol then yielded 5-pyridyl substituted analogues **72**–**78**, **81** and **85**–**86**. Disubstituted pyridines **82**–**83** were synthesized in a similar manner from **91b**–**c** derived from the appropriate aldehyde **89c**–**d** (Scheme 5), while ethyl analogue **80** was afforded by using ethylamine in place of cyclopropylethylamine in the reductive amination step. *N*-Methylaniline sulfonamide **84** was synthesized from sulfonyl chloride **93** and aniline prior to amide coupling with intermediate **90a** and Cu(I)-mediated aromatic substitution of **95** with ethanolamine. Analogues **57** and **71** were afforded directly from intermediates **88a**–**b** and 4-(phenylsulfamoyl)benzoyl chloride. Tetrazole **79** was synthesized through an alternative three-step sequence from **63** via a Pd-catalyzed Heck reaction with acrylonitrile, Pd-induced catalytic transfer hydrogenation using triethylsilane (Et_3_SiH), and thermal azide-nitrile cycloaddition (Scheme 5).

We first attempted to block metabolism with the 5-fluoropyridin-2-yl analogue **71**, however rapid in vitro RLM clearance was observed (Table 1). However, installing polar, water-soluble pendant groups led to consistent and significantly lowered human and rat microsomal CL_int_ (**72**–**76**, Table 1). Neutral pendants maintained similar LIMK1/2 potencies to **57**, however *N*-linked tertiary amines such as **77** or highly basic analogues such as **78** led to a large drop off in potency, in line with previously observed SAR. We deprioritised C-linked tethers due to their increased *c* log *D*, however the few analogues synthesized all demonstrated worse potency compared to parent compound **57**, except for tetrazole **79** (Table 1). We hypothesized that **79** could be gaining an additional π-cation interaction with K368 that offset the potency drop associated with the C-linker. However, **79** was not progressed based on very poor cell permeability. Overall, MDI-65658 (**74**) combined the desired features of LIMK1/2 potency and metabolic stability, although cell permeability and very high drug efflux were not optimal. We rationalized based on **71** that poor permeability within the series was due to either high TPSA (≥90 Å) and/or increased HBD count (≥1), two parameters that are also expected to limit CNS penetration.^28,29^ Therefore, we began a lead optimization campaign around **74** aimed addressing HBD count and TPSA while maintaining good LIMK1/2 potency and in vitro metabolic stability.

Blocking the ethanolamine HBD through *gem*-dimethyl analogue **81** significantly impacted LIMK1 potency compared to lead compound **74** (Table 2), and therefore was not profiled further. 6-Me or 4-Me substitution of the pyridine ring (**82** and **83** respectively), expected to also block *N*-pyridine oxidation, failed to address poor permeability. Our previous ethanolamine SAR indicated that NH/OH substitutions adversely impacted potency (**77**) or microsomal CL_int_ (**75**, Table 1), and unfortunately removing the remaining HBD through sulfonamide *N*-methylation similarly reduced potency and increased metabolic instability (**84**, Table 2). We also tried curtailing the cPr ring of **74** with ethyl analogue **80**; however, this caused over a 10-fold drop in LIMK1 potency (Table 2) consistent with our previous SAR (Table S6). Finally, we attempted to reduce TPSA by switching the pyridine to a phenyl ring of **74**. This led to MDI-114215 (**85**), the most optimal tool molecule identified to-date with excellent LIMK1/2 potency, significantly improved Caco-2 permeability and lowered drug efflux (Table 2). The close ethylene glycol analogue **86** expectedly improved cell permeability due to further lowering TPSA, however rat/human metabolic instability slightly increased in parallel with *c* log *D*.

**Table 2:**
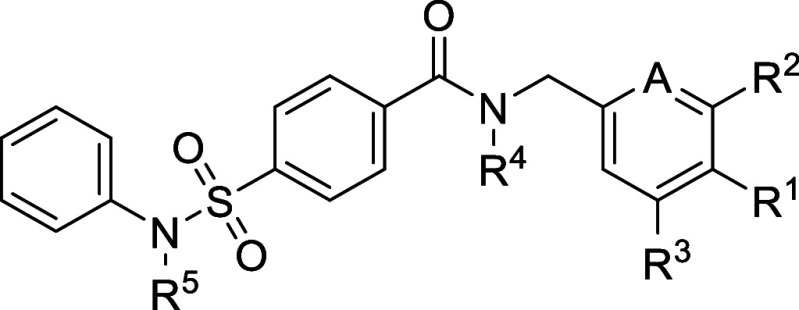

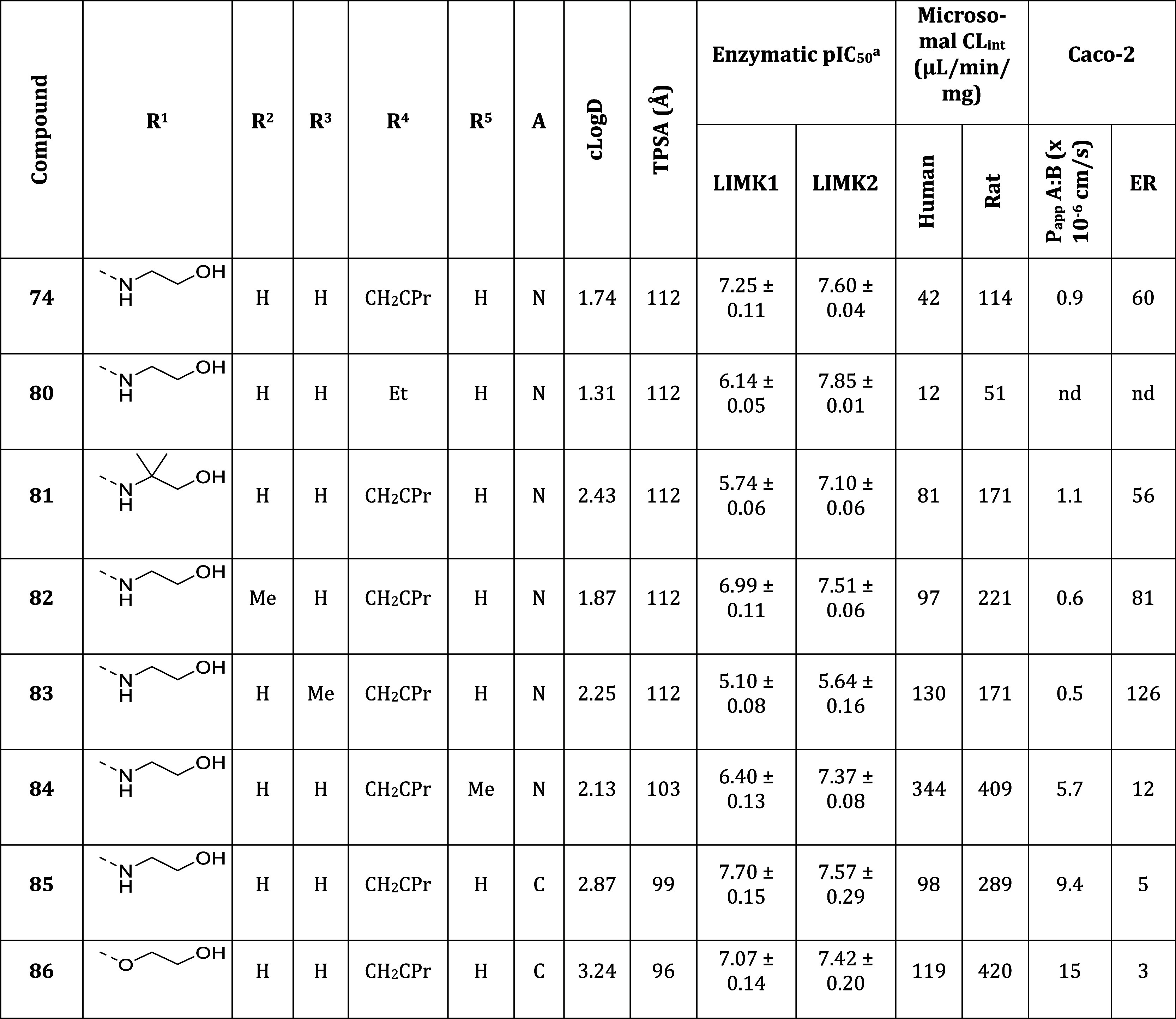
LIMK1/2 Inhibitory Activities of MDI-65658 (**74**) and Analogues **80–86**^b^

a pIC50 is the negative logarithm of the IC_50_ value. nd, not determined.

b Data are reported as mean ± SEM of at least 3 independent experiments.

**Figure 2:**
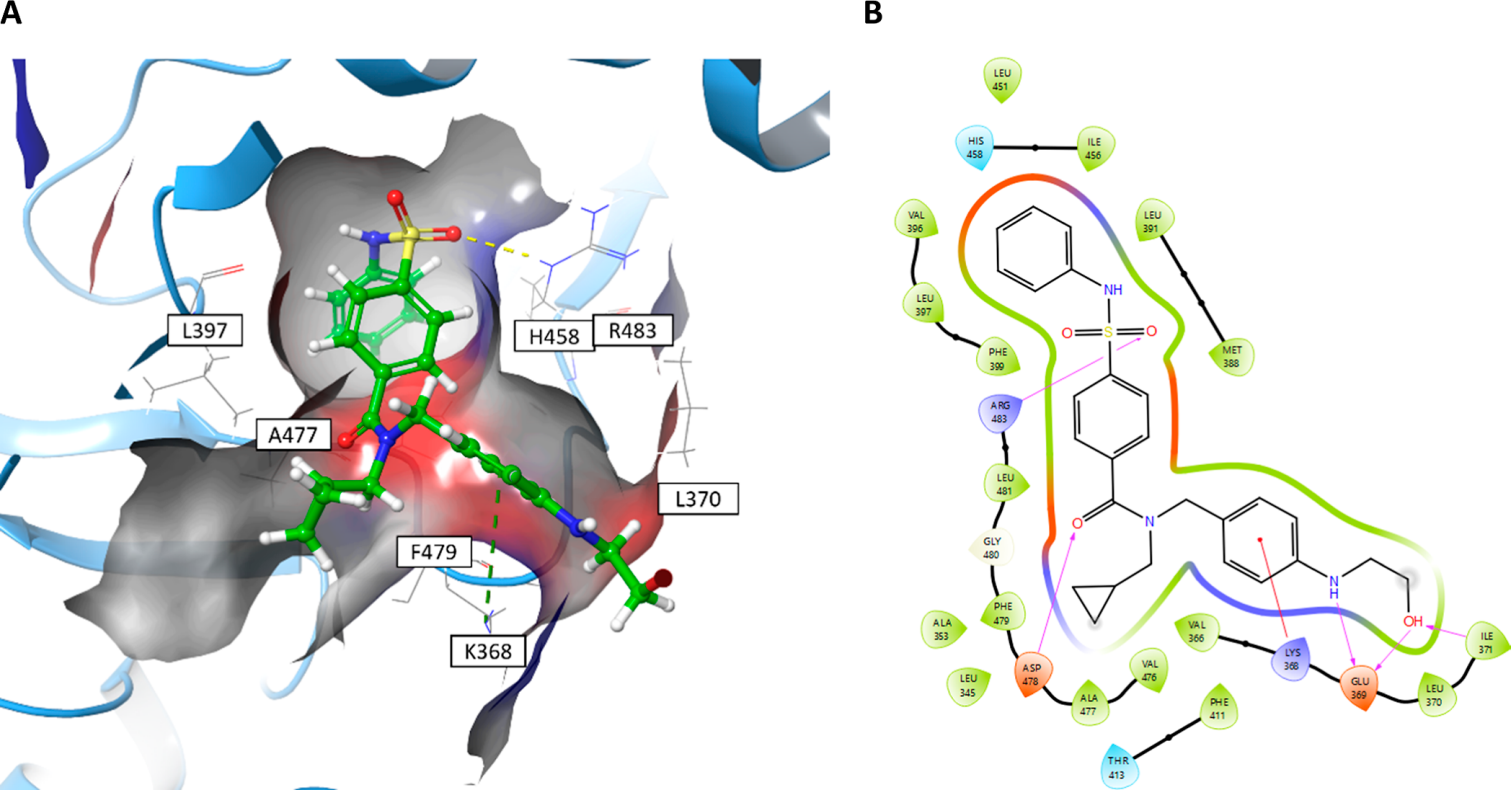
New structural insights of LIMK1 highlighted by novel compound **85**. (a) **85** docked into the homology model of LIMK1 (generated from PDB code 5NXD). Interactions involving key residues are labeled and drawn using dashed lines. The protein surface and nearby allosteric residues have been hidden for clarity. (b) Ligand interaction diagram of **85**. Shading represents the following: hydrophobic region (green), charged interaction (positive, blue; negative, red), polar (teal).

Modeling **85** into the LIMK1 homology structure suggests that the compound sits away from the classic Type-I kinase hinge binding pocket (Figure 2). The cyclopropyl terminus sits in a hydrophobic region flanked by V366, the hydrophobic chain of catalytic K368, L397, T413 and F479, and the amide O acceptor interacts with D478 on the DFG loop. Importantly, the substituted ethanolamine NH donor forms a new interaction with E369, which terminates with a pendant ethyl alcohol that acts as both a donor and acceptor to E369 and I371, respectively. As the pendant group is flexible and projects toward solvent, the terminal hydroxyl group is also free to rotate and interact with environmental water. As expected, other protein–ligand interactions were consistent with previous LIMK1/2 cocrystal structures.

### MDI-114215 Inhibits PAK1-Phosphorylated LIMK Activity and Reduces Cellular p-Cofilin Levels

Key compounds of interest were further profiled in a series of affinity enzymatic and cellular inhibition assays against LIMK1 and LIMK2 (Table 3). Dissociation constant (*K*_d_) determinations to measure binding affinity were performed using KINOMEscan at Eurofins/DiscoverX. As LIMK1/2 is activated by phosphorylation on Thr508/Thr505 by PAK1–4,^30^ we modified the previously reported RapidFire mass spectrometry assay^31^ by conducting our enzymatic inhibition assay in both the presence and absence of the PAK1 kinase domain. Cellular target engagement and selectivity was assessed using LIMK1 and LIMK2 NanoBRET assays in HEK293 cells. Cellular proof-of-mechanism was assessed by measuring the effect of LIMK inhibitors on reducing p-cofilin levels in SH-SY5Y cells using the AlphaLISA platform, an assay that cannot discriminate between LIMK1 and/or LIMK2 inhibition since cofilin is a substrate for both enzymes.

**Table 3:** Evaluation of Key LIMK Inhibitors against PAK-Phosphorylated LIMK Enzymes and Cellular Activities^a^

compound	binding affinity (p*K*_d_)^b^		enzymatic pIC_50_^c^		nanoBRET pIC_50_^c^		alphaLISA pIC_50_^c^
	LIMK1	LIMK2	PAK pLIMK1	PAK pLIMK2	LIMK1	LIMK2	p-cofilin
**51**	nd	nd	6.71 ± 0.06	7.77 ± 0.08	6.61 ± 0.10	6.88 ± 0.02	6.15 ± 0.17
**55**	6.66^d^	7.00^d^	5.19^d^	7.30 ± 0.01	5.69 ± 0.09^e^	5.86 ± 0.16^e^	5.63 ± 0.01^e^
**74**	7.01 ± 0.05^e^	6.94 ± 0.06^e^	7.04 ± 0.10	7.54 ± 0.05	6.78 ± 0.02	6.74 ± 0.02	6.18 ± 0.10
**75**	nd	nd	7.14 ± 0.06	7.43 ± 0.02	7.30 ± 0.24	7.11 ± 0.31	7.11 ± 0.11
**85**	7.48 ± 0.18	7.40 ± 0.06	7.89 ± 0.07	7.96 ± 0.04	7.34 ± 0.06	7.30 ± 0.08	7.30 ± 0.11
**86**	nd	nd	7.35 ± 0.03	7.74 ± 0.02	7.41 ± 0.29	7.11 ± 0.30	7.25 ± 0.02

a Data are reported as mean ± SEM of at least 3 independent experiments, unless otherwise stated.

b Screened at DiscoverX, Eurofins (San Diego, U.S.A.) using KdELECT.

c pIC50 is the negative logarithm of the IC_50_ value.

d *n* = 1.

e Mean of two independent experiments. nd, not determined.

The results generally showed very consistent effects on LIMK1/2 affinity and enzymatic inhibition of PAK1-phosphorylated LIMK1/2 (PAK1-pLIMK1/2) in addition to potent cellular target engagement and decreased p-cofilin levels (Table 3). Caco-2 cell permeability correlated well with observed inhibitory activities in cellular assays, underlining our strategy to optimize physicochemical properties responsible for poor permeability. Although **55** continued to demonstrate greater selectivity for LIMK2 over LIMK1 in recombinant assays (approximately 55-fold and 130-fold selectivities for non-pLIMK1/2 and PAK1-pLIMK1/2 RapidFire assay, respectively), LIMK2 potency dropped substantially when profiled in cellular assays due to poor permeability (data not shown). Overall, our most advanced tool compound **85** demonstrated very high affinity and enzymatic non-pLIMK1/2 and PAK1-pLIMK1/2 inhibition (IC_50_ ≤ 40 nM), excellent cellular NanoBRET target engagement and potently reduced cellular p-cofilin levels in the AlphaLISA assay.

### MDI-114215 Has Acceptable DMPK Properties Suitable for In Vivo Evaluation

Moreover, we evaluated key compounds for aqueous solubility and in vivo PK to determine their suitability as molecules to investigate LIMK pathologies in vivo. Despite the low solubilities for most compounds profiled, the 5-((2-hydroxyethyl)amino)pyridine feature significantly improved the aqueous solubility of compound **74** (Table 4). Generally, in vivo clearance was moderate-high for most selected inhibitors, particularly for **74**, leading to suboptimal drug exposures after normalizing for dose (Table 4). There was an expectedly strong correlation between increased drug efflux and lowered CNS penetration, for example **51** was significantly more brain penetrant (B/P = 0.4, *K*_p,uu_ = < 0.03, ER = 1.9) than **74** (B/P and *K*_p,uu_ = 0, ER = 60, Table 4), despite similar Caco-2 permeability. Although **85** showed limited CNS penetration (B/P = 0.1, *K*_p,uu_ = < 0.1, Table 4), its lower in vivo clearance and more optimal permeability led us to believe it could attain high enough brain concentrations through greater drug exposure in vivo.

**Table 4:** In Vitro and In Vivo DMPK Properties for Selected Compounds **51**, **55**, **74**, **75**, **85** and **86**

compound	in vitro DMPK		in vivo DMPK									
			i.v. dosing						p.o./i.p. dosing			
	aq. solubility (μM, pH 7)^a^	fu_brain_/fu_plasma_ (%)	dose (mg/kg)	CL_int_ (mL/min/kg)	*V*_D_ (L/kg)	*T*_1/2_ (h)	AUC_inf_ (h ng/mL)	B/P/*K*_p,uu_	route	*C*_max_ (ng/mL)	*T*_max_ (h)	*F* (%)
**51**	3	<0.1/1.3	1	67.8	2.3	0.5	247	0.4/<0.03	p.o.^b^	4.6	1.0	2
**55**	27	nd	0.2^d^	16.5	0.4	0.3	217	nd	nd	nd	nd	nd
**74**	186	2.8/8.0	1	51.9	1.8	0.7	322	0	p.o.^b^	50.8	0.4	15
**75**	28	2.2/4.7	0.5	374	10.9	0.4	22.6	<0.1^c^/<0.1	p.o.^b^	16.8	0.4	13
									i.p.^c^	134	0.3	55
**85**	11	0.8/1.8	0.2^d^	33.1	1.3	0.6	101	0.1^c^/< 0.1	i.p.^e^	1385	0.4	52
**86**	6	0.9/1.4	nd	nd	nd	nd	nd	<0.1^c^/0.1	nd	nd	nd	nd

a Average of two separate experiments.

b 3 mg/kg dose.

c Determined internally after i.p. dosing at 10 mg/kg.

d Dosed as a cassette of five compounds.

e 30 mg/kg dose. nd, not determined.

Based on these results, we then further profiled **51**, **74**, and **85** for i.p./p.o. dosing. Consistent with its high in vivo clearance, **51** had minimal drug exposure p.o. with very low *C*_max_ and oral bioavailability (Table 4). Therefore, despite its improved CNS penetration, **51** could not be progressed further. **74** had improved exposure in vivo and oral bioavailability, however the measured peak total, and preferably, free drug concentration (106 and 4 nM, respectively) were not sufficient to cover in vitro LIMK1/2 RapidFire IC_50_ by several fold to ensure efficacy could be observed. **85** had the most optimal in vivo PK profile, demonstrating good bioavailability and significantly greater peak total plasma concentrations at 30 mg/kg dose sufficient to achieve approximately 100-fold LIMK1/2 IC_50_ (Table 4). Taking into account plasma protein binding (98.2%, Table 4), the total free plasma drug exposure of **85** is expected to cover LIMK1/2 IC_50_ ([**85**]_plasma_ = 52 nM). A dose escalation study ranging from 10, 30, and 50 mg/kg via i.p. administration also identified 30 mg/kg as the most suitable dose for in vivo evaluation (Figure S2).

### MDI-114215 Is Highly Selective for LIMK1/2 with Minimal Off-target Liabilities

**Figure 3:**
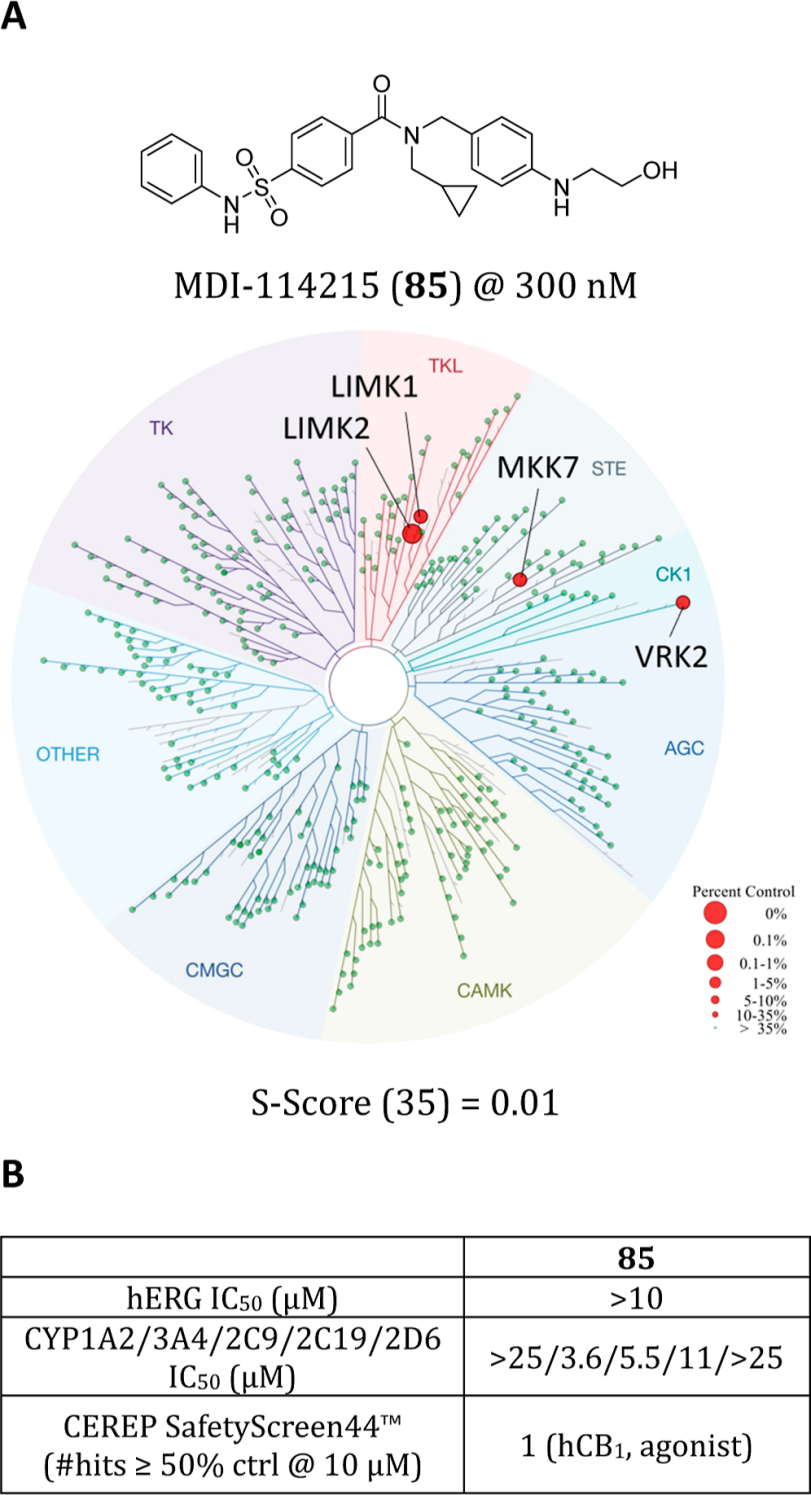
Kinome selectivity and safety profiling of **85**. (a) Chemical structure of **85** and kinome screen data illustrated using the TREEspot interaction map (DiscoverX). (b) hERG, CYP450 and CEREP panel profiling data of **85**.

Wider kinome profiling was performed on **85** using the Eurofins/DiscoverX scanMAX panel of 468 kinases at 300 nM, approximately 10-fold LIMK1/2 RapidFire IC_50_ (Table S7). The selectivity score (*S*_35_) is calculated by measuring the number of kinases that the compound binds to by ≥35% relative to control, divided by the total number of distinct kinases tested, which facilitates comparisons of different compounds. We observed remarkable selectivity for LIMK1/2 with a *S*_35_ of 0.01 (Figure 3A), making **85** one of the most selective LIMK1/2 inhibitors reported to-date.^14^ Importantly, **85** did not bind TESK1, a close neighbor that is a member of the TKL kinase family, nor to CaMKIV, MRCKα, PAK1–2/4 or ROCK1/2, all of which are known to activate LIMK1/2 through phosphorylation^30,32−34^ and thereby could confound assay interpretation similar to the dual LIMK/PAK inhibitor FRAX486.^12,14^

We also evaluated **85** for off-target pharmacological activity in a panel of receptors, ion channels, transporters and enzymes using the CEREP SafetyScreen44 panel (Eurofins, France, Table S8) in addition to key safety liabilities. There were no major liabilities against hERG (IC_50_ > 10 μM) and minimal off-target activities were identified (Figure 3B). CYP_450_ profiling showed moderate inhibition of CYP3A4 and CYP2C9 (IC_50_ = 3.6 μM and 5.5 μM, respectively), however **85** did not significantly inhibit the other CYP isoforms tested (Figure 3B). Taken together, these data strongly suggests that **85** is a highly selective, potent LIMK1/2 inhibitor with optimized in vivo PK suitable as a tool compound for investigating LIMK pathologies.

### MDI-114215 Is Well Tolerated in a 28 day Study in Young Male Mice

**Figure 4:**
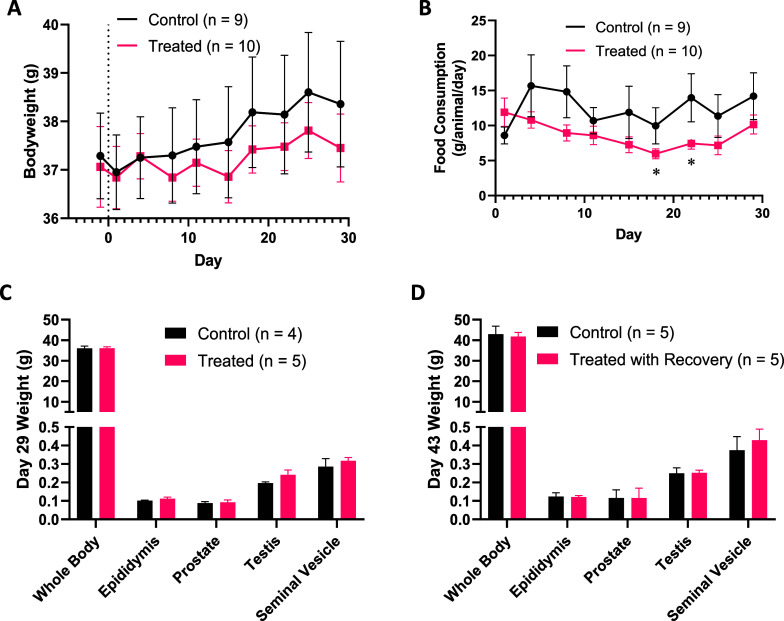
MDI-114215 (**85**) was well tolerated in male CD-1 mice (30 mg/kg/day i.p. for 28 days). (a) No significant change in bodyweight was detected between animals dosed with vehicle or 30 mg/kg q.d. i.p. with **85** for 28 days. (b) Food consumption over course of treatment. A small difference (*P < 0.05) in consumption was detected between days 15–22 only but had resolved by the end of the study. (c) Weights of key male sexual organs after 29 days dosing of **85** and, (d) after a further two-week recovery period. Data is represented as mean ± SEM. One control animal was euthanised on day 14 due to convulsions.

*Limk2*^–/–^ mice were previously reported to have impaired spermatogenesis and phenotypic abnormalities such as reduced size and weight of testes.^35^ To investigate the potential impact of chronic LIMK2 inhibition on testicular toxicity, compound **85** was evaluated in a 28 day i.p. toxicology study with two week recovery in male CD-1 mice and potential adverse effects on male sexual organs assessed. Young male mice (6 weeks age at study start) were selected to maximize vulnerability of sexual organs to possible developmental disruption. **85**-treated mice (dosed at 30 mg/kg/day, i.p.) showed no changes in body weight or food consumption compared to healthy control mice (Figure 4A,B). No adverse clinical signs, macro- or microscopic findings were observed in either vehicle-treated or **85**-treated mice. There were no statistically significant differences in organ weights measured when compared to controls, particularly male sexual organs (Figure 4C,D). Nonadverse, multifocal unilateral minimal inflammatory cell infiltrate was observed in the epididymis of some animals, however substantial recovery was observed after 14 days treatment-free period. Bioanalysis of plasma and brain tissue samples taken at the end of the 28 day treatment period (approximately 1–2 h after the last administration) showed significant total levels of **85** in plasma (903 ± 219 nM) compared to brain (62 ± 6 nM). This equates to approximately 40-fold and 3-fold enzymatic LIMK1/2 IC_50,_ respectively. It should be noted that compound **85** demonstrates a relatively short duration of exposure (Figure S2) and will likely be at or below LIMK1/2 IC_50_, if free drug levels are considered. In conclusion, these data suggests that daily administration of **85** by i.p. injection at a dose of 30 mg/kg to CD-1 male mice for 28 days is well tolerated.

### MDI-114215 Reduces p-Cofilin in Mouse Hippocampal Slices of Fragile X Syndrome Mice

**Figure 5:**
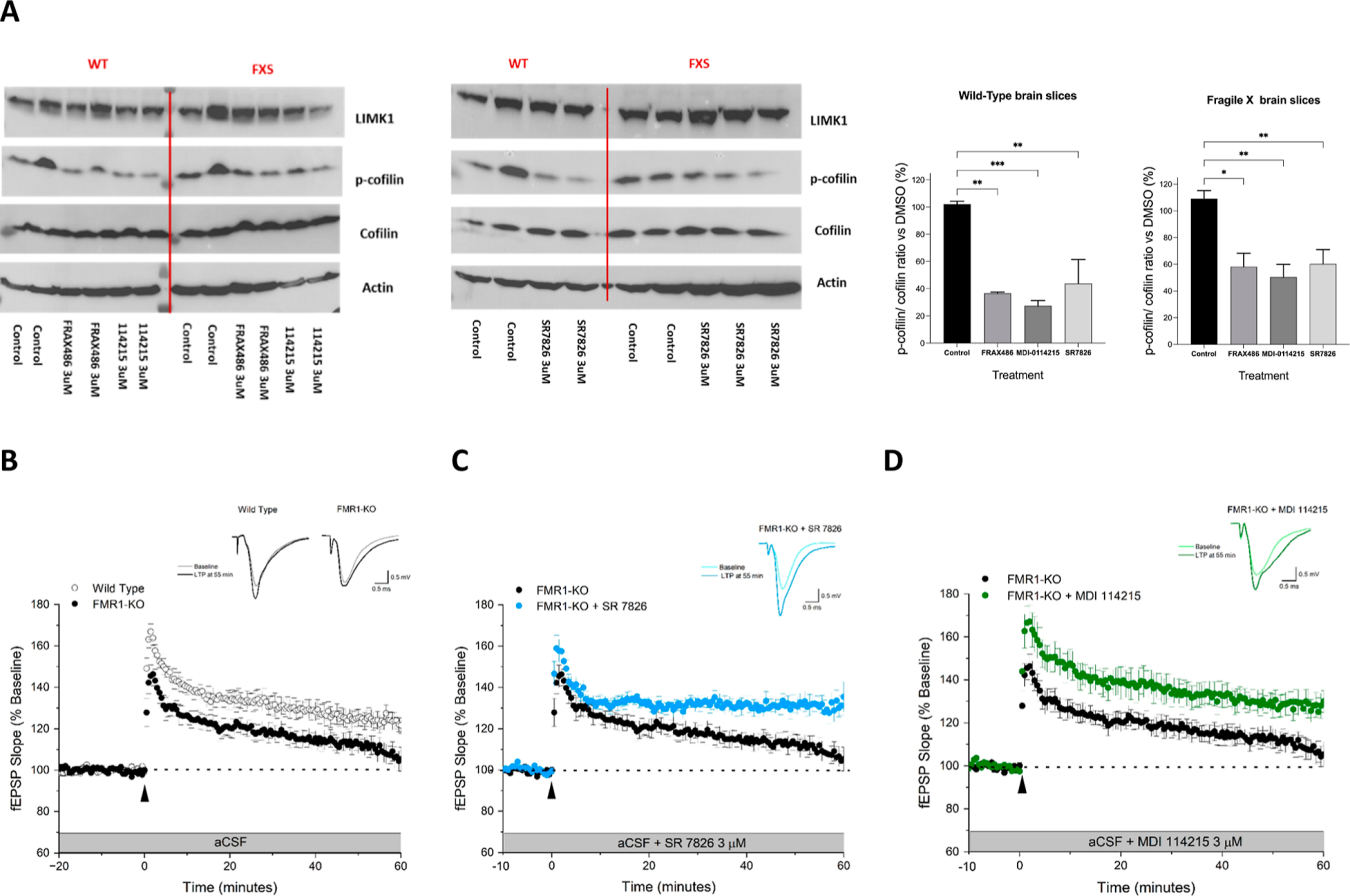
MDI-114215 (**85**) decreases phosphorylated cofilin levels ex vivo and reverses the deficit in LTP of mouse *Fmr1* KO hippocampal CA1 pyramidal neurons. (a) Western blot analysis of treated brain slices isolated from young WT or *Fmr1* KO (P7–9) showing significant reductions (*P < 0.05, **P < 0.01, ***P < 0.001 determined by one-way ANOVA with Dunnett’s multiple comparisons test) in p-cofilin upon incubation with 3 μM of DMSO control (P7 WT *n* = 4, *Fmr1* KO *n* = 4), **2** (P7 WT *n* = 2, *Fmr1* KO *n* = 2), **4** (P7 WT *n* = 2, *Fmr1* KO *n* = 3), **85** (P7 WT *n* = 2, *Fmr1* KO *n* = 2). Quantification of p-cofilin to cofilin ratio are also presented. (b–d) Illustrated plots of the field excitatory postsynaptic potential (fEPSP) slope against time (mean ± s.e.m). All fEPSPs were recorded from the hippocampal CA1 dendritic field region of neonatal (P7–9) wild type (WT) and *Fmr1* KO mice. LTP was expressed as a percentage of the control normalized mean fEPSP slope and was determined between 50 and 60 min postdelivery of the 4-TBS. For each plot representative traces of fEPSPs obtained at baseline and 55 min after the TBS are shown overlaid.

We evaluated **85** alongside the dual PAK/LIMK inhibitor FRAX486 (**2**), previously progressed as a clinical candidate for FXS, and LIMK1 inhibitor SR7826 (**4**), in the *Fmr1* KO mouse model of FXS. We selected compounds **2** and **4** as positive controls as **2** has been previously shown to restore (reduce) p-cofilin levels in the somatosensory cortex of 1 week-old *Fmr1* KO mice,^3^ while **4** reduces p-cofilin levels in rat neurons and synaptosome fractions isolated from the hippocampus of nontransgenic and hAPP mice.^21^ Consequently, **4** is reported to provide dendritic spine resilience to amyloid-β (Aβ) and rescue Aβ-induced hippocampal spine loss and morphological abnormalities.^21^ Brain slices from young (P7–9) WT and *Fmr1* KO mice were treated with vehicle or 3 μM LIMK inhibitor and p-cofilin levels were quantitatively compared by Western blot analysis (Figures S3 and S4). We observed a consistent reduction in p-cofilin upon treatment of **2** and **4** compared to vehicle control in both WT and *Fmr1* KO mice (Figure 5A). Interestingly, p-cofilin to cofilin ratio remained unchanged between nontreated, control WT and *Fmr1* KO mice. Importantly, **85** potently decreased p-cofilin levels in both WT and *Fmr1* KO mice to a similar level to **2** (Figure 5). Taken together, these data indicate that **85** significantly inhibits LIMK1/2 activity and decreases p-cofilin levels, demonstrating ex vivo target engagement in FXS mice suitable for in vivo efficacy evaluation.

### MDI-114215 Rescues Impaired Hippocampal Long-Term Potentiation in Neonatal Fragile X Syndrome Mice

A prior study reported the magnitude of LTP recorded from the hippocampal CA1 region of neonatal (P6–9) *Fmr1* KO mice to be impaired in comparison to that of equivalent WT mice.^36^ Here, delivery of a 2 s 4-TBS protocol enhanced the fEPSP slope recorded from the hippocampal CA1 dendritic region of neonatal (P7–9) WT mice (24 ± 3.9% increase of the fEPSP, *n* = 10 slices) determined between 50 and 60 min post the 4-TBS i.e. LTP (Figure 5B). In agreement with Banke and Barria, equivalent recordings made from hippocampal CA1 neurons of age matched *Fmr1* KO mice revealed the magnitude of LTP to be significantly reduced in comparison to their WT counterparts [*Fmr1* KO = 9.8 ± 4.1% increase of the fEPSP, *n* = 11 slices (*p* = 0.02, independent *t*-test)].

Having confirmed an LTP deficit in the hippocampus of the neonatal *Fmr1* KO mouse, we then investigated the effect of the LIMK inhibitor **4**(21) upon the magnitude of LTP in neonatal (P7–9) WT and *Fmr1* KO mouse hippocampus. The hippocampus was perfused with **4** (3 μM) for 30 min prior to delivery of the 4-TBS and was continually perfused for a further 60 min following the high frequency electrical stimulation (see General Methods). **4** significantly increased the magnitude of LTP recorded from the *Fmr1* KO mouse hippocampus (SR7826 = 32 ± 4.3% increase, *n* = 7 slices, *p* = 0.002, independent *t*-test, Figure 5C). By contrast, **4** (3 μM) had no significant effect on the magnitude of hippocampal LTP of the neonatal (P7–9) WT mice (WT + SR7826 = 14 ± 2.9% increase, *n* = 7 slices *p* = 0.069, independent *t*-test, data not shown). Having established the efficacy of the known LIMK inhibitor **4**, we now investigated whether the novel LIMK inhibitor **85** (3 μM) employing the same perfusion protocol (see Methods) was effective in enhancing the magnitude of hippocampal LTP in neonatal *Fmr1* KO mice. In common with SR7826, the perfusion of **85** produced a significant enhancement of hippocampal LTP (*Fmr1* KO + **85** = 28 ± 5.3% increase of the fEPSP, *n* = 8, *p* = 0.0125, independent *t*-test; Figure 5D).

### Inhibition of LIMK Reduces p-Cofilin in Stem Cell-Derived Human Neurons

**Figure 6:**
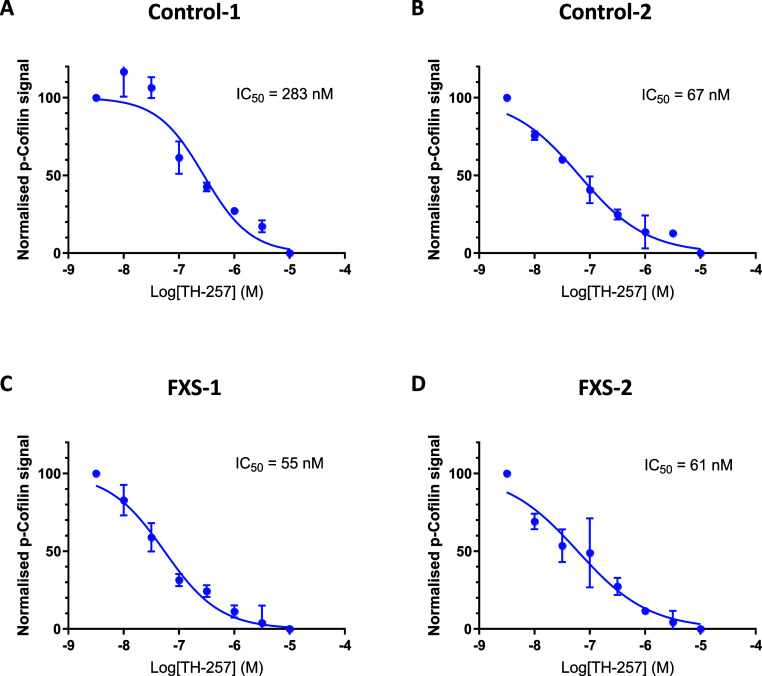
Dose–response curves showing reduction in p-cofilin by TH-257 (**6**) in stem cell-derived neurons from two control individuals: (a) KYOU 1 week neurons, (b) AIW002 1 week neurons, and two FXS individuals: (c) FX11–7 and (d) FX8–1. Levels of p-cofilin were measured using the AlphaLISA assay. Data are reported as mean ± SEM of at least 3 independent experiments.

To further assess proof-of-mechanism and demonstrate the potential of selective LIMK inhibitors to treat FXS, human induced pluripotent stem cells (iPSCs)-derived neural progenitors from control and FXS patients were differentiated over 1 week into cortical neurons and then treated with various concentrations of **6** with p-cofilin levels analyzed by AlphaLISA. **6** dose-dependently inhibited p-cofilin in human cortical neurons derived from both normal and patient-derived stem cells (Figure 6). Similar dose–responses were also observed for ATP-competitive LIMK inhibitors such as **2** and **3** (data not shown), which have previously demonstrated efficacy in the *Fmr1* KO mouse model^12^ or ameliorated aberrant differentiation phenotypes of FMRP-deficient human neural progenitor cells (NPCs) and neurons.^37^ Thus, our lead series, exemplified by lead compound **6**, is able to potently inhibit LIMK1/2 activity ex vivo in stem-cell derived cortical neurons isolated from FXS patients.

LIMK1 is a master regulator of actin stability and consequently synaptic formation and development. Therefore, inhibitors that attenuate increased LIMK1 activity due to increased activation of both BMPR2 and Rac1-PAK1 signaling pathways would correct the defects in synaptic function that occurs in FXS. There are currently no available therapies for FXS and despite the clear medical need, individuals typically receive help with management of specific symptom domains, such as by administration of anticonvulsants, SSRIs and psychostimulants.^1^ Most therapeutic strategies to reverse intellectual disability in FXS previously focused on addressing the excitatory/inhibitory imbalance by modulating the mGluR and GABA systems, in particular the development of mGluR5 NAMs from Novartis, Roche and Merck/Seaside Therapeutics. However, these programmes have been discontinued after failing to show efficacy in Phase II trials.^38^ Based upon recent mechanistic studies identifying increased LIMK1 activation as a causative factor of synaptic dysfunction in FXS,^2,3,8,12^ a LIMK1 inhibitor represents an attractive approach for treating FXS pathology. Here we report the discovery and proof-of-mechanism of **85**, a novel, potent and highly selective pan-LIMK inhibitor that significantly reduces p-cofilin levels ex vivo and reverses hippocampal LTP deficits in FXS mice.

Application of structure-based drug design using a LIMK1 homology model around a previously selective but nonoptimized compound series^26^ guided the synthesis of 4-benzyl substituted molecules incorporating polar, water solubilizing groups with greater metabolic stability and solubility. During the course of our study, we were unable to achieve LIMK1 selectivity over LIMK2. LIMK1 and LIMK2 share high sequence similarity within their kinase domains (73%) with only one residue variance in the allosteric pocket [F411 (LIMK1), L403 (LIMK2)], likely explaining the lack of isoform selectivity. Previous studies using hippocampal brain slices derived from LIMK1^–/–^, LIMK2^–/–^ and LIMK1/2^–/–^ double KO mice have shown that while LIMK1 is the major kinase responsible for decreasing p-cofilin levels, a further and significant reducing in p-cofilin was observed in LIMK1/2 double KO compared to LIMK1^–/–^ alone,^39,40^ highlighting that LIMK2 also plays an additional role in maintaining hippocampal p-cofilin levels in the absence of LIMK1. Indeed, LIMK2^–/–^ mice do not show detrimental CNS-related effects^40^ and although a previous report showed LIMK2 KO mice have impaired spermatogenesis,^35^ we demonstrated that chronic treatment of **85** does not cause testicular toxicity (Figure 4C,D). These data suggest that on a background of increased LIMK1 activity in FXS, any LIMK2 inhibitory activity should not be a significant liability.

Our mechanistic studies demonstrated that **85** is an equipotent inhibitor of nonphosphorylated and PAK1-phosphorylated LIMK1/2, with similar potencies attained in the cellular NanoBRET target engagement and AlphaLISA p-cofilin assays. We previously reported that ATP-competitive LIMK inhibitors, such as FRAX486 and LX7101, showed a clear loss of potency in vitro when evaluated against PAK1-pLIMK1/2.^14^ As LIMK1/2 is activated by phosphorylation at T508/T505 by PAK1^30^ and p-T508-LIMK1 levels are significantly elevated in the somatosensory cortex of FXS mice,^3^ non-ATP competitive inhibitors such as **85** would be highly desirable as they can inhibit both unmodified LIMK and PAK1-pLIMK observed in FXS.

A previous study reported a deficit of hippocampal CA1 LTP in neonatal (P6–9) *Fmr1* KO mice, an impairment of synaptic plasticity not evident a few days later in older (P14–19) *Fmr1* KO mice.^36^ In agreement, we found the magnitude of LTP recorded from the dendritic field of hippocampal CA1 neurons obtained from *Fmr1* KO (P7–9) mice was greatly impaired compared to their age matched WT counterparts. There are numerous studies implicating LIMKs, particularly LIMK1, in aspects hippocampal synaptic plasticity, including LTP.^41^ In mice, global genetic deletion of LIMK1 had no influence on basal glutamatergic transmission in the hippocampal CA1 region but did enhance LTP in response to high frequency presynaptic stimulation.^39^ This profile was common to the LIMK1/2^–/–^ double KO mouse, suggesting a dominant role for the LIMK1 isoform.^40^ In support, the magnitude of LTP in the LIMK2 KO mouse was similar to that of WT.^40^ Previously, acute inhibition by the dual LIMK1/2 inhibitor Pyr1 was shown to enhance the impaired hippocampal LTP evident in a mouse model of schizophrenia.^25^ Here, application of the LIMK1 inhibitor SR7826 selectively enhanced the magnitude of LTP recorded from the *Fmr1* KO hippocampus but no effect was observed on the LTP of WT mice. **85** was similarly effective in enhancing LTP of the *Fmr1* KO hippocampus.

The ratio of p-cofilin to cofilin in the somatosensory cortex is reported to be greater in neonatal (P7) *Fmr1* KO mice, compared to age matched WT mice, a genotype difference that is not evident in 4 week-old mice.^3^ By contrast, here there was no genotype difference in the p-cofilin/cofilin ratio for our hippocampal slices obtained from P7–9 mice. However, incubation of the tissue with LIMK inhibitors **4** or **85** (3 μM) significantly reduced the p-cofilin/cofilin ratio for both WT and *Fmr1* KO slices. As discussed above, the rescue of impaired LTP by acute inhibition of LIMK in P7 *Fmr1* KO mice is consistent with the studies on the LIMK KO mice^39,40^ and with the effects of a LIMK inhibitor in a mouse model of schizophrenia.^25^ However, inactivation of cofilin by phosphorylation has been proposed to facilitate LTP (see Zablah et al.^41^, 2021 for review). Our demonstration that both **4** and **85** (3 μM) decrease the p-cofilin/cofilin ratio but nevertheless rescue impaired LTP suggests a cofilin-independent effect of LIMK inhibition on this form of synaptic plasticity in neonatal *Fmr1* KO mice. Clearly, further investigation is required to dissect the mechanism of action of LIMK inhibitors on LTP, specifically in FXS mice.

We anticipate that **85** will serve as a useful probe to further interrogate LIMK biology. Pathological changes in synaptic structure and function are associated with several psychiatric and neurodegenerative disorders and therefore a LIMK1/2 inhibitor such as **85** could have broader therapeutic potential in other CNS disorders, including amyotrophic lateral sclerosis,^42^ schizophrenia^43^ and Alzheimer’s disease.^21^ There is also growing evidence that LIMK1/2 overexpression and dysregulation leads to tumorigenesis and metastasis of several cancers caused by aberrant actin cytoskeleton remodelling.^20^

## Conclusions

Extensive optimization of the *N*-phenylsulfamoylbenzamide series with appropriate substituents exploiting a small hydrophobic cleft and solvent-accessible region by using a SBDD approach generated more potent dual LIMK1/2 inhibitors. Structure–clearance relationships and a focused chemistry strategy aimed to optimize potency, in vitro microsomal clearance and permeability led to discovery of **85**, a highly potent, selective and well tolerated LIMK1/2 inhibitor with significantly improved in vitro metabolic stability and optimal PK profile suitable for in vivo proof-of-concept evaluation. Compound **85** effectively inhibited both nonphosphorylated and PAK1-phosphorylated LIMK1/2 with good cellular target engagement. Compound **85** potently suppressed cellular cofilin phosphorylation in vitro and ex vivo, and reversed hippocampal LTP deficits in a mouse model of FXS. We further demonstrate the potential utility of LIMK inhibitors in FXS, which potently decrease phosphorylated cofilin levels in iPSC-derived neurons from FXS patients. Compound **85** is an excellent tool compound for researchers to study the role of LIMKs in FXS and, more generally, in health and disease. Further evaluation of compound **85** is currently ongoing in several cancers that have significant unmet clinical need.

## Experimental Section

### General Methods

4-(Phenylsulfamoyl)benzoic acid was prepared as previously described.^14^ All other commercial materials were used as received without further purification. Identity and purity checks were carried out prior to use in biological experiments using ^1^H NMR spectroscopy and UPLC–MS analysis as detailed in our previous publication.^14^

### Synthetic Procedures and Compound Characterization

The majority of final compounds were determined to be >95% pure, as determined by ^1^H NMR or UPLC–MS analyses. An example VT-NMR spectrum showing presence of rotamers for asymmetric tertiary amides synthesized herein can be found in Figure S5. ^1^H, ^13^C and ^19^F NMR spectra were recorded on a Bruker Avance III HD 500 or 400 MHz equipped with a Prodigy cryoprobe. Chemical shifts (δ) are defined in parts per million (ppm). ^1^H NMR spectra were referenced to tetramethylsilane (TMS, δ = 0.0 ppm) or residual undeuterated solvent (DMSO-*d*_6_, δ = 2.50 ppm; MeOD-*d*_4_, δ = 3.31 ppm; CDCl_3_, δ = 7.26 ppm). ^13^C NMR spectra were referenced to residual undeuterated solvent as an internal reference and ^19^F NMR spectra were pseudo referenced to the ^1^H chemical shift of undeuterated solvent. Multiplicities are abbreviated as follows: s, singlet; d, doublet; t, triplet; q, quartet; dd, doublet of doublets; tt, triplet of triplets; pent, pentet; hept, heptet; m, multiplet; br, broad, or combinations thereof. Coupling constants were measured in Hertz (Hz). Liquid chromatography–mass spectrometry (LCMS) was carried out on a Waters Acquity Hclass plus UPLC coupled to a Waters Acquity HPLC PDA detector and a Waters Acquity QDa API-ES mass detector. Samples were eluted through a BEH C18 2.1 mm × 50 mm, 1.7 μm column or a Cortecs C18 2.1 mm × 50 mm, 1.6 μm column using H_2_O and MeCN acidified by 0.1% formic acid. The gradient runs H_2_O/MeCN/formic acid at 90:10:0.1–10:90:0.1 for 3 min at 1.5 mL/min and detected at 254 nm. Molecular ion peaks are defined as mass/charge (*m*/*z*) ratios. Analytical thin-layer chromatography (TLC) was performed using VWR silica gel 60 on aluminum plates coated with *F*_254_ indicator. All spots were visualized with ultraviolet light using a UVP C-10 Chromato-Vue cabinet or stained using KMnO_4_. Normal-phase purifications were completed using a Teledyne ISCO CombiFlash NEXTGEN 300+ using silica gel with particle size 40–63 μm; reverse-phase purifications were completed using a Teledyne ACCQPrep system equipped with a 20 mm × 150 mm C18 column and eluted with a 10–100% MeOH/H_2_O gradient. Evaporation of solvents was conducted on a Buchi Rotavapor R-300.

#### General Procedure A—Sulfonamide Coupling

Unless otherwise stated, a primary or secondary amine (5 equiv) was added to a solution of a 4-(chlorosulfonyl)(hetero)aryl derivative (1 equiv) in THF (8–10 mL). The reaction mixture was stirred at room temperature overnight. The reaction mixture was filtered and the precipitate dried thoroughly to afford the sulfonamide coupled product.

#### General Procedure B—Reductive Amination

Unless otherwise stated, a primary amine (1.2 equiv) was added to a solution of a substituted aldehyde (1 equiv) and NaHCO_3_ (3–4 equiv) in MeOH (4–8 mL). The reaction mixture was stirred at room temperature overnight. The reaction mixture was then cooled to 0 °C and NaBH_4_ (1.2 equiv) was added portion-wise. The reaction mixture was left to stir at 0 °C for 1 h, allowing to warm to room temperature. The reaction mixture was quenched with water (10 mL) and the organic components were concentrated under reduced pressure. The reaction mixture was diluted with DCM (10 mL) and the organic phase separated using a phase separator. The filtrate was concentrated under reduced pressure to afford the reductive aminated product.

#### General Procedure C—Amide Coupling: HOBt/EDC Method

Unless otherwise stated, EDC·HCl (1.2 equiv) was added to a solution of (hetero)aryl carboxylic acid (1 equiv) and HOBt hydrate (1.1 equiv) in DCM (2–8 mL). The reaction mixture was stirred at room temperature for 30 min. A secondary amine (1.5 equiv) was then added. The reaction mixture was further stirred at room temperature overnight. Saturated NaHCO_3_ (10 mL) was added and the reaction mixture stirred for 15 min at room temperature. The organic phase was separated using a phase separator and the filtrate concentrated under reduced pressure with silica. The crude mixture was purified by flash column chromatography and fractions containing product were combined and concentrated under reduced pressure to afford the amide coupled product.

#### General Procedure D—Amide Coupling: Acid Chloride Method

Unless otherwise stated, Et_3_N (1.5 equiv) was added to a solution of a secondary amine (1.5 equiv) in DCM (3–12 mL). The reaction mixture was cooled to 0 °C, followed by addition of a 4-(phenylsulfamoyl)benzoyl chloride derivative (1 equiv) in DCM (2–8 mL). The reaction mixture was stirred at 0 °C for 1 h, allowing to warm to room temperature. The reaction mixture was diluted with DCM (20 mL) and washed with saturated NaHCO_3_ (20 mL), 1 M HCl (20 mL), water (20 mL) and brine (20 mL). The organic layer separated using a phase separator and the filtrate was concentrated under reduced pressure with silica. The crude mixture was purified by flash column chromatography and fractions containing product were combined and concentrated under reduced pressure to afford the amide coupled product.

#### General Procedure E—Synthesis of Sulfonamides from (Hetero)aryl Bromides

Using an adapted procedure,^27^ a substituted (hetero)aryl bromide (1 equiv) was added to a solution of potassium disulfite (2 equiv), tetrabutylammonium bromide (1.1 equiv), sodium formate (2.2 equiv), Pd(OAc)_2_ (5% mol), PPh_3_ (0.15 equiv) and 1,10-phenanthroline (0.15 equiv) in anhydrous DMSO (2 mL). The reaction mixture was degassed (bubbling N_2_) for 10 min, then heated to 70 °C while stirring for 3 h. The reaction mixture was then cooled to room temperature. Aniline (10 equiv) was added and the reaction mixture cooled to 0 °C. A solution of NCS (2 equiv) in anhydrous THF (2 mL) was then added. The reaction mixture stirred at 0 °C for 2 h, allowing to warm to room temperature. The reaction mixture was diluted with EtOAc (20 mL) and washed with water (2 × 20 mL) and brine (20 mL). The organic phase was dried over MgSO_4_, filtered and concentrated under reduced pressure with silica. The crude mixture was purified by flash column chromatography and fractions containing product were combined and concentrated under reduced pressure to afford the sulfonamide product.

#### General Procedure F—Ullmann Coupling

Unless otherwise stated, an appropriate amine or alcohol (2 equiv) was added to a solution of a substituted *N*-(hetero)aryl-*N*-(cyclopropylmethyl)-4-(phenylsulfamoyl)benzamide (1 equiv), l-proline (0.2 equiv) and K_2_CO_3_ (2 equiv) in DMSO (3 mL). The reaction mixture was degassed with N_2_ for 5 min, before CuI (0.1 equiv) was added. The reaction mixture was further degassed with N_2_ for another 5 min before heating to 80 °C overnight. The reaction mixture was filtered through Celite, extracted with EtOAc (20 mL) and washed with water (2 × 20 mL) and brine (20 mL). The organic layer was dried over MgSO_4_, filtered and concentrated under reduced pressure with silica. The crude mixture was purified by flash column chromatography using the solvent conditions stated. Fractions containing product were combined and concentrated under reduced pressure to afford the Ullmann coupled products.

#### Key Intermediate A—Synthesis of 4-(Phenylsulfamoyl)benzoyl Chloride

A thick suspension of 4-(phenylsulfamoyl)benzoic acid (2 g, 7.2 mmol) in SOCl_2_ (5 mL, 69 mmol) had DMF (20 μL) added. The reaction mixture was heated to 70 °C for 2 h, whereupon the suspension became more fluid. The reaction mixture was cooled to room temperature and the excess SOCl_2_ was removed under vacuum to yield a light brown solid which was dissolved in DCM to be used directly as crude.

Alternatively, 4-(phenylsulfamoyl)benzoic acid (500 mg, 1.80 mmol) in (COCl)_2_ (0.18 mL, 2.16 mmol) and anhydrous DCM (5 mL) had DMF (20 μL) added. The reaction mixture was stirred at room temperature for 5 h. The reaction mixture was then concentrated under reduced pressure to yield a brown solid which was dissolved in DCM to be used directly as crude.

#### Key Intermediate B—Synthesis of 4-Formyl-*N*-phenylbenzenesulfonamide (**12**)

A solution of 4-formylbenzene-1-sulfonyl chloride (500 mg, 2.44 mmol), aniline (0.26 mL, 2.69 mmol), and pyridine (0.22 mL, 2.69 mmol) in DCM (10 mL) were stirred at room temperature for 3 h. The reaction was concentrated under reduced pressure and purified by flash column chromatography (silica, 24 g, 1:0 petrol/EtOAc to 1:1 petrol/EtOAc). Fractions containing product were combined and concentrated under reduced pressure to afford 4-formyl-*N*-phenylbenzenesulfonamide (260 mg, 1.00 mmol, 41% yield) as yellow foam. ^1^H NMR (500 MHz, CDCl_3_): δ 10.08 (s, 1H), 8.02–7.83 (m, 4H), 7.31–7.26 (m, 2H), 7.21–7.16 (m, 1H), 7.12–7.08 (m, 2H). NH not observed. ACQUITY UPLC BEH C18 1.7 μm: *R*_t_ = 1.60 min; *m*/*z* 262.0 [M + H]^+^.

#### Key Intermediate C—Synthesis of 4-[Benzyl(butyl)carbamoyl]benzenesulfonyl Chloride (**67**)

4-(Chlorosulfonyl)benzoic acid (1.00 g, 4.53 mmol), DMF (0.01 mL) and SOCl_2_ (5.00 mL, 68.55 mmol) were heated at 70 °C for 3 h in a sealed 20 mL Biotage microwave vial using an aluminum heating mantle, whereupon the mixture became homogeneous. Following cooling, the mixture was transferred to a 100 mL round bottomed flask with the aid of DCM (20 mL) and the mixture concentrated under reduced pressure in a fumehood to give the crude acid chloride, which was used without further purification.

The crude acid chloride was dissolved in anhydrous THF (25 mL) under a N_2_ atmosphere. Et_3_N (0.95 mL, 6.8 mmol) was added and the mixture cooled to −78 °C using a dry ice–acetone bath. To a 20 mL Biotage microwave vial under a nitrogen atmosphere was added *N*-benzylbutylamine (0.81 mL, 4.53 mmol) and anhydrous THF (5 mL). The solution was cooled to −78 °C under a N_2_ atmosphere. Once cooled, the solution of the amine was transferred slowly via cannula using a gentle positive pressure of nitrogen to a rapidly stirred solution of the acid chloride. Upon completion of addition (approximately 5 min), the cooling bath was removed and the reaction mixture left to warm up gradually for 30 min. The reaction mixture was diluted with EtOAc (50 mL), washed with water (30 mL), saturated NaHCO_3_ (3 × 30 mL), 0.5 M HCl solution (2 × 30 mL), the organic phase dried over MgSO_4_, filtered and concentrated under reduced pressure to give the crude acid chloride 4-[benzyl(butyl)carbamoyl]benzenesulfonyl chloride (1.36 g, 3.486 mmol, 77% yield), which was used without further purification. A 1 M stock solution of the sulfonyl chloride was prepared in THF and used in subsequent reactions. ^1^H NMR (500 MHz, CDCl_3_): δ 8.10 (d, *J* = 8.1 Hz, 1H), 8.02 (d, *J* = 8.1 Hz, 1H), 7.68–7.57 (m, 2H), 7.42–7.28 (m, 4H), 7.13 (d, *J* = 7.4 Hz, 1H), 4.78 (s, 1H), 4.44 (s, 1H), 3.51 (t, *J* = 7.7 Hz, 1H), 3.08 (t, *J* = 7.6 Hz, 1H), 1.71–1.61 (m, 1H), 1.53–1.44 (m, 1H), 1.42–1.32 (m, 1H), 1.16–1.06 (m, 1H), 0.95 (t, *J* = 7.4 Hz, 1.5H), 0.76 (t, *J* = 7.4 Hz, 1.5H). Rotamers observed in approximately 1:1 ratio. ACQUITY UPLC BEH C18 1.7 μm: *R*_t_ = 1.90 min; *m*/*z* 366.1 [M + H, ^35^Cl]^+^, 368.1 [M + H, ^37^Cl]^+^.

#### 4-[(Benzylamino)methyl]-*N*-phenyl-benzenesulfonamide (**13**)

Synthesized according to general procedure B. *N*-Benzylamine (0.03 mL, 0.23 mmol), 4-formyl-*N*-phenyl-benzenesulfonamide (50 mg, 0.19 mmol, Key Intermediate B), NaHCO_3_ (24 mg, 0.29 mmol), NaBH_4_ (8 mg, 0.21 mmol). The reduction was conducted at 0 °C for 4 h, allowing to warm to room temperature. Crude product taken to next step without further purification. ^1^H NMR (500 MHz, CDCl_3_): δ 7.67–7.59 (m, 2H), 7.40–7.32 (m, 2H), 7.29–7.22 (m, 4H), 7.19–7.14 (m, 3H), 7.10–7.01 (m, 1H), 7.00–6.97 (m, 2H), 3.76 (s, 2H), 3.71 (s, 2H). 2× NH not observed. ACQUITY UPLC BEH C18 1.7 μm: *R*_t_ = 1.30 min; *m*/*z* 353.1 [M + H]^+^.

#### *N*-Benzyl-*N*-(4-(*N*-phenylsulfamoyl)benzyl)butyramide (**9**)

Synthesized according to general procedure C. Butyric acid (24 μL, 0.33 mmol, 1.2 eq used), HOBt hydrate (48 mg, 0.31 mmol), EDC.HCl (65 mg, 0.34 mmol), 4-[(benzylamino)methyl]-*N*-phenyl-benzenesulfonamide (100 mg, 0.28 mmol), DCM (4 mL). The reaction mixture was directly concentrated under reduced pressure and purified by flash column chromatography (silica, 24 g, 1:0 petrol/EtOAc to 0:1 petrol/EtOAc). Yield: 48 mg, 0.11 mmol, 38%. Colorless solid. mp 121–123 °C; IR (neat) ν_max_/cm^–1^: 3146, 2963, 2932, 2874, 1622, 1135. ^1^H NMR (500 MHz, CDCl_3_): δ 7.80–7.75 (m, 1H), 7.73–7.68 (m, 1H), 7.37–7.05 (m, 12H), 4.58 (d, *J* = 13.4 Hz, 2H), 4.44 (d, *J* = 13.3 Hz, 2H), 2.49–2.39 (m, 1H), 2.36–2.24 (m, 1H), 1.81–1.64 (m, 2H), 1.01–0.84 (m, 3H). NH not observed. Rotamers observed in approximately 2:1 ratio. ^13^C NMR (151 MHz, CDCl_3_): δ 174.1, 173.8, 143.0, 142.3, 138.6, 138.3, 136.9, 136.7, 136.6, 135.9, 129.3, 129.1, 128.7, 128.5, 128.3, 128.0, 127.5, 126.9, 126.5, 125.3, 121.6, 121.5, 50.6, 49.7, 48.5, 48.0, 35.1, 18.9, 14.0. ACQUITY UPLC BEH C18 1.7 μm: *R*_t_ = 1.82 min; *m*/*z* 432.2 [M + H]^+^. HRMS (EI) calcd for C_24_H_27_O_3_N_2_S, 423.1742; found, 423.1733.

#### 4-(Butylaminomethyl)-*N*-phenyl-benzenesulfonamide (**14**)

Synthesized according to general procedure B. *N*-Butylamine (46 μL, 0.46 mmol), 4-formyl-*N*-phenyl-benzenesulfonamide (100 mg, 0.38 mmol, Key Intermediate B), NaHCO_3_ (51 mg, 0.61 mmol), NaBH_4_ (20 mg, 0.54 mmol, 1.4 eq used). The reduction was conducted at 0 °C for 4 h, allowing to warm to room temperature. Crude product taken to next step without further purification. ^1^H NMR (500 MHz, CDCl_3_): δ 7.72 (d, *J* = 8.3 Hz, 2H), 7.41 (d, *J* = 8.0 Hz, 2H), 7.25 (t, *J* = 7.9 Hz, 2H), 7.14 (q, *J* = 7.7 Hz, 1H), 7.07 (d, *J* = 7.8 Hz, 2H), 3.83 (s, 2H), 2.61 (t, *J* = 7.2 Hz, 2H), 1.49 (h, *J* = 6.6, 5.8 Hz, 2H), 1.36 (h, *J* = 7.4 Hz, 2H), 0.92 (t, *J* = 7.4 Hz, 3H). Two x NH not observed. ACQUITY UPLC BEH C18 1.7 μm: *R*_t_ = 1.31 min; *m*/*z* 319.1 [M + H]^+^.

#### *N*-Butyl-*N*-(4-(*N*-phenylsulfamoyl)benzyl)benzamide (**10**)

Synthesized according to general procedure C. Benzoic acid (25 μL, 0.26 mmol), HOBt hydrate (60 mg, 0.39 mmol, 1.5 equiv used), EDC.HCl (81 mg, 0.42 mmol, 1.6 equiv used), 4-(butylaminomethyl)-*N*-phenyl-benzenesulfonamide (104 mg, 0.33 mmol, 1.3 equiv used), DCM (5 mL). The reaction mixture was directly concentrated under reduced pressure and purified by flash column chromatography (silica, 24 g, 1:0 petrol/EtOAc to 0:1 petrol/EtOAc). Yield: 90 mg, 0.21 mmol, 62%. Colorless solid. IR (neat) ν_max_/cm^–1^: 3155, 2957, 2930, 2872, 1597, 1155. ^1^H NMR (500 MHz, CDCl_3_): δ 7.73 (d, *J* = 7.9 Hz, 2H), 7.49–7.28 (m, 7H), 7.23 (d, *J* = 7.7 Hz, 2H), 7.12 (t, *J* = 7.4 Hz, 1H), 7.06 (d, *J* = 7.9 Hz, 2H), 6.71 (s, 1H), 4.77 (s, 1.5H), 4.51 (s, 0.7H), 3.56–3.30 (m, 0.8H), 3.25–3.05 (m, 1.4H), 1.51–1.29 (m, 2H), 1.19–1.01 (m, 1H), 1.01–0.81 (m, 0.8H), 0.81–0.62 (m, 2.1H). CH not observed as overlapping with HDO peak. Rotamers observed in approximately 2:1 ratio. ^13^C NMR (151 MHz, CDCl_3_): δ 172.5, 143.1, 142.6, 138.4, 136.6, 136.1, 129.7, 129.3, 128.6, 128.3, 127.7, 126.5, 125.3, 121.6, 52.3, 48.8, 47.5, 45.1, 30.4, 29.2, 20.2, 19.6, 13.9, 13.5. ACQUITY UPLC BEH C18 1.7 μm: *R*_t_ = 1.82 min; *m*/*z* 423.1 [M + H]^+^. HRMS (EI) calcd for C_24_H_26_O_3_N_2_S, 422.1659; found, 422.1655.

#### *N*-Benzyl-*N*-butyl-4-(phenylsulfonamido)benzamide (**15**)

Ethyl 4-(phenylsulfonamido)benzoate (142 mg, 0.47 mmol) was dissolved in 1,2-dichloroethane (5 mL) and the mixture degassed (N_2_ bubbling) for 10 min while cooling at 0 °C. In a separate vial triethylaluminum (1 M in hexanes, 2.3 mL, 2.3 mmol) and *N*-benzylbutylamine (0.42 mL, 2.34 mmol) were added to degassed (N_2_ bubbling) 1,2-dichloromethane (5 mL) at 0 °C. The mixture was warmed to room temperature and then transferred via syringe to the vial containing the solution of ethyl 4-(phenylsulfonamido)benzoate. The reaction mixture was then warmed to room temperature and then heated to 80 °C and stirred overnight. The reaction was cooled to 0 °C and then quenched with 1 M aq. HCl until pH 1 was observed. The mixture was diluted with dichloromethane (25 mL) and the phases separated. The organics were washed with 1 M aq HCl (3 × 20 mL), water (3 × 20 mL) and brine (20 mL). The organic layer was dried over MgSO_4_, filtered and concentrated under reduced pressure with silica. The crude product was purified by flash column chromatography (silica, 12 g, 1:0 DCM/MeOH to 49:1 DCM/MeOH over 25 CV’s), followed by additional purification by reverse-phase column chromatography (9:1 H_2_O/MeOH to 0:1 H_2_O/MeOH over 25 min). Fractions containing product were combined and concentrated under reduced pressure to afford *N*-benzyl-*N*-butyl-4-(phenylsulfonamido)benzamide (160 mg, 0.38 mmol, 81% yield) as a colorless glass. ^1^H NMR (500 MHz, MeOD-*d*_4_): δ 7.78 (t, *J* = 9.9 Hz, 2H), 7.59–7.39 (m, 3H), 7.37–7.25 (m, 6H), 7.24–7.03 (m, 3H), 4.72 (s, 1.2H), 4.47 (s, 0.9H), 3.40 (s, 0.9H), 3.13 (s, 1.1H), 1.66–1.52 (m, 0.9H), 1.50–1.39 (m, 1.3H), 1.38–1.26 (m, 1.2H), 1.10–0.98 (m, 0.9H), 0.97–0.84 (m, 1.5H), 0.68 (t, *J* = 7.4 Hz, 1.6H). Rotamers observed in approximately 1:1 ratio. ACQUITY UPLC BEH C18 1.7 μm: *R*_t_ = 1.84 min; *m*/*z* 423.1 [M + H]^+^.

#### *N*^1^-Benzyl-*N*^1^-butyl-*N*^4^-phenylterephthalamide (**17**)

Methyl 4-(anilinocarbonyl)benzoate (100 mg, 0.39 mmol) was suspended in 1,2-dichloroethane (5 mL) and the mixture degassed for 10 min (N_2_ bubbling purge). In a separate vial, 1,2-dichloroethane (5 mL) was degassed for 10 min (N_2_ bubbling purge) before cooling the solvent to 0 °C and then adding concurrently *N*-benzylbutylamine (0.35 mL, 1.96 mmol) and triethylaluminum solution (1 M in hexanes, 2 mL, 2 mmol). The mixture was warmed to room temperature and stirred for 30 min. Meanwhile, the suspension of methyl 4-(anilinocarbonyl)benzoate in 1,2-dichloroethane was cooled to 0 °C before adding, via syringe, the solution containing triethylaluminum. The resulting reaction mixture was warmed to room temperature and stirred for 2 h before being heated to 80 °C and stirred overnight. The reaction mixture was cooled to room temperature and slowly quenched with 1 M aq. HCl until the pH was acidic (pH 1–2). The mixture was diluted with water (20 mL) and then extracted with DCM (3 × 50 mL). The combined organic layers were concentrated under reduced pressure with silica. The crude mixture was purified by flash column chromatography (silica, 12 g, 1:0 petrol/EtOAc to 1:3 petrol/EtOAc over 25 CV’s). The appropriate fractions containing product were combined and concentrated under reduced pressure. The residue was further purified by trituration with petroleum ether, followed by filtration and then trituration in diethyl ether followed by filtration. The isolated solid was dried thoroughly overnight to afford *N*^1^-benzyl-*N*^1^-butyl-*N*^4^-phenylterephthalamide (95 mg, 0.24 mmol, 62% yield) as a colorless solid. ^1^H NMR (500 MHz, CDCl_3_): δ 8.21 (s, 0.4H), 8.14 (s, 0.5H), 7.89 (d, *J* = 7.8 Hz, 1H), 7.82 (d, *J* = 7.9 Hz, 1H), 7.68 (dd, *J* = 17.1, 8.0 Hz, 2H), 7.46 (t, *J* = 7.9 Hz, 2H), 7.42–7.27 (m, 6H), 7.20–7.10 (m, 2H), 4.79 (s, 1H), 4.46 (s, 1H), 3.48 (t, *J* = 7.7 Hz, 1H), 3.10 (t, *J* = 7.7 Hz, 1H), 1.65 (p, *J* = 7.8 Hz, 1H), 1.47 (p, *J* = 7.8 Hz, 1H), 1.36 (h, *J* = 7.4 Hz, 1H), 1.08 (h, *J* = 7.4 Hz, 1H), 0.95 (t, *J* = 7.3 Hz, 1.4H), 0.75 (t, *J* = 7.3 Hz, 1.5H). Rotamers observed in approximately 1:1 ratio. ACQUITY UPLC BEH C18 1.7 μm: *R*_t_ = 1.88 min; *m*/*z* 387.2 [M + H]^+^.

#### *N*-Benzyl-4-bromo-*N*-butyl-benzamide (**26a**)

Synthesized according to general procedure C. 4-Bromobenzoic acid (500 mg, 2.49 mmol), HOBt hydrate (419 mg, 2.74 mmol), EDC.HCl (572 mg, 2.98 mmol), *N*-benzylbutylamine (0.67 mL, 3.73 mmol). Purified by flash column chromatography (silica, 12 g, 1:0 petrol/EtOAc to 1:1 petrol/EtOAc over 25 CV’s). Yield: 798 mg, 2.19 mmol, 88%. Colorless oil. ^1^H NMR (500 MHz, CDCl_3_): δ 7.60–7.43 (m, 2H), 7.39–7.24 (m, 6H), 7.20–7.08 (m, 1H), 4.75 (s, 1H), 4.48 (s, 1H), 3.44 (t, *J* = 8.5 Hz, 1H), 3.12 (t, *J* = 7.3 Hz, 1H), 1.68–1.55 (m, 1H), 1.48–1.40 (m, 1H), 1.38–1.27 (m, 1H), 1.15–1.03 (m, 1H), 0.98–0.86 (m, 1.4H), 0.77 (t, *J* = 7.7 Hz, 1.4H). Rotamers observed in approximately 1:1 ratio. ACQUITY UPLC BEH C18 1.7 μm: *R*_t_ = 1.93 min; *m*/*z* 346.0 [M + H, ^79^Br]^+^, 348.0 [M + H, ^81^Br]^+^.

#### *N*-Benzyl-4-benzylsulfonyl-*N*-butyl-benzamide (**29**)

Using a previously described procedure,^27^*N*-benzyl-4-bromo-*N*-butyl-benzamide (300 mg, 0.87 mmol) was added to a solution of potassium disulfite (385 mg,1.73 mmol), tetrabutylammonium bromide (308 mg, 0.95 mmol), sodium formate (132 mg, 1.91 mmol), Pd(OAc)_2_ (10 mg, 0.04 mmol), PPh_3_ (34 mg, 0.13 mmol) and 1,10-phenanthroline (23 mg, 0.13 mmol) in anhydrous DMSO (4 mL). The reaction mixture was degassed (bubbling N_2_) for 10 min, then heated to 70 °C while stirring for 3 h. After cooling the reaction mixture to room temperature, benzyl bromide (0.15 mL, 1.3 mmol) was added. The reaction mixture was stirred at room temperature overnight. The reaction mixture was diluted with EtOAc (20 mL) and washed with water (2 × 20 mL) and brine (20 mL). The organic layer was dried over MgSO_4_, filtered and concentrated under reduced pressure with silica. The crude mixture was purified by flash column chromatography (silica, 12 g, 1:0 petrol/EtOAc +1% Et_3_N to 1:1 petrol/EtOAc +1% Et_3_N over 30 CV’s). Fractions containing product were combined and concentrated under reduced pressure. The solid was triturated in hot MeOH (3 mL) and washed with additional MeOH (3 × 5 mL). The precipitate was then dried thoroughly to afford *N*-benzyl-4-benzylsulfonyl-*N*-butyl-benzamide (238 mg, 0.54 mmol, 62% yield) as a colorless solid. ^1^H NMR (500 MHz, CDCl_3_): δ 7.67 (d, *J* = 7.9 Hz, 1H), 7.60 (d, *J* = 7.9 Hz, 1H), 7.52–7.41 (m, 2H), 7.40–7.17 (m, 7H), 7.14–7.03 (m, 3H), 4.76 (s, 1H), 4.37 (s, 1H), 4.33 (s, 1H), 4.29 (s, 1H), 3.48 (t, *J* = 7.8 Hz, 1H), 3.02 (t, *J* = 7.7 Hz, 1H), 1.69–1.58 (m, 1H), 1.44 (t, *J* = 7.6 Hz, 1H), 1.39–1.31 (m, 1H), 1.07 (h, *J* = 7.5 Hz, 1H), 0.95 (t, *J* = 7.4 Hz, 1.3H), 0.76 (t, *J* = 7.4 Hz, 1.5H). Rotamers observed in approximately 1:1 ratio. ACQUITY UPLC BEH C18 1.7 μm: *R*_t_ = 1.84 min; *m*/*z* 422.2 [M + H]^+^.

#### *N*-Benzyl-4-bromo-*N*-butyl-3-fluoro-benzamide (**26b**)

Synthesized according to general procedure C. 4-Bromo-3-fluoro-benzoic acid (500 mg, 2.28 mmol), HOBt hydrate (385 mg, 2.51 mmol), EDC.HCl (525 mg, 2.74 mmol), *N*-benzylbutylamine (0.61 mL, 3.42 mmol). Purified by flash column chromatography (silica, 12 g, 1:0 petrol/EtOAc to 1:1 petrol/EtOAc over 25 CV’s). Yield: 735 mg, 1.92 mmol, 84% yield. Colorless oil. ^1^H NMR (400 MHz, CDCl_3_): δ 7.57 (d, *J* = 25.4 Hz, 1H), 7.41–7.27 (m, 4H), 7.19 (d, *J* = 8.5 Hz, 1H), 7.16–7.04 (m, 2H), 4.74 (s, 1H), 4.48 (s, 1H), 3.43 (d, *J* = 8.9 Hz, 1H), 3.21–3.03 (m, 1H), 1.54–1.41 (m, 1H), 1.41–1.27 (m, 1H), 1.17–1.05 (m, 1H), 0.92 (t, *J* = 7.8 Hz, 1.5H), 0.78 (t, *J* = 7.4 Hz, 1.5H). Rotamers observed in approximately 1:1 ratio. CH_2_ for one rotamer not observed as overlapping with HDO peak ACQUITY UPLC BEH C18 1.7 μm: *R*_t_ = 1.93 min; *m*/*z* 364.1 [M + H, ^79^Br]^+^, 366.1 [M + H, ^81^Br]^+^.

#### *N*-Benzyl-*N*-butyl-3-fluoro-4-(phenylsulfamoyl)benzamide (**19**)

Synthesized according to general procedure E. *N*-Benzyl-4-bromo-*N*-butyl-3-fluoro-benzamide (300 mg, 0.82 mmol), potassium disulfite (366 mg, 1.65 mmol), tetrabutylammonium bromide (293 mg, 0.91 mmol), sodium formate (125 mg, 1.81 mmol), Pd(OAc)_2_ (9.3 mg, 0.04 mmol), PPh_3_ (32 mg, 0.12 mmol), 1,10-phenanthroline (22 mg, 0.12 mmol), aniline (0.75 mL, 8.24 mmol), NCS (220 mg, 1.65 mmol). Purified by flash column chromatography (silica, 12 g, 1:0 petrol/EtOAc to 1:1 petrol/EtOAc over 30 CV’s). Yield: 83 mg, 0.18 mmol, 22%. Colorless solid. ^1^H NMR (500 MHz, CDCl_3_): δ 7.85 (t, *J* = 7.6 Hz, 0.5H), 7.77 (t, *J* = 7.5 Hz, 0.4H), 7.41–7.28 (m, 4H), 7.25–7.15 (m, 3.7H), 7.14–7.03 (m, 3.7H), 6.77 (br s, 1H), 4.72 (s, 1H), 4.37 (s, 0.8H), 3.46 (t, *J* = 7.6 Hz, 0.8H), 3.01 (t, *J* = 7.8 Hz, 1H), 1.66–1.56 (m, 0.7H), 1.47–1.38 (m, 0.8H), 1.34 (h, *J* = 7.5 Hz, 0.6H), 1.06 (h, *J* = 7.5 Hz, 1H), 0.93 (t, *J* = 7.4 Hz, 1.2H), 0.73 (t, *J* = 7.3 Hz, 1.5H). Rotamers observed in approximately 3:2 ratio. ^19^F NMR (376 MHz, CDCl_3_): δ −109.2 (s). ACQUITY UPLC BEH C18 1.7 μm: *R*_t_ = 1.86 min; *m*/*z* 441.1 [M + H]^+^.

#### 3-Chloro-4-iodo-benzoic Acid (**25c**)

Methyl-3-chloro-4-iodobenzoate (250 mg, 0.840 mmol) was dissolved in 1:1:3 MeOH/THF/H_2_O (1.5, 1.5, 4.5 mL, respectively), to which lithium hydroxide monohydrate (126 mg, 1.69 mmol) was added. The reaction mixture was stirred at room temperature for 2 h. The reaction mixture was concentrated under reduced pressure. 1 M HCl (5 mL) was added and a solid immediately precipitated. The precipitate was filtered, washed with water (10 mL) and dried to afford 3-chloro-4-iodo-benzoic acid (129 mg, 0.44 mmol, 91% yield) as a colorless solid. ^1^H NMR (500 MHz, DMSO-*d*_6_): δ 13.44 (br s, 1H), 8.10 (dd, *J* = 8.2, 1.8 Hz, 1H), 7.97 (d, *J* = 2.1 Hz, 1H), 7.60–7.55 (m, 1H). ACQUITY UPLC BEH C18 1.7 μm: *R*_t_ = 2.01 min; *m*/*z* 280.8 [M-H]^−^.

#### *N*-Benzyl-*N*-butyl-3-chloro-4-iodo-benzamide (**26c**)

Synthesized according to general procedure C. 3-Chloro-4-iodo-benzoic acid (220 mg, 0.74 mmol), HOBt hydrate (125 mg, 0.810 mmol), EDC.HCl (170 mg, 0.89 mmol), *N*-benzylbutylamine (0.20 mL, 1.11 mmol). Purified by flash column chromatography (silica, 12 g, petrol/EtOAc 0–100% over 25 CV’s). Yield: 292 mg, 0.65 mmol, 88%. Colorless oil. ^1^H NMR (500 MHz, CDCl_3_): δ 7.90 (d, *J* = 8.0 Hz, 0.4H), 7.82 (d, *J* = 8.3 Hz, 0.5H), 7.49 (s, 1H), 7.39–7.27 (m, 4H), 7.16–7.08 (m, 1H), 7.04–6.94 (m, 1H), 4.74 (s, 1H), 4.47 (s, 1H), 3.43 (t, *J* = 8.2 Hz, 1H), 3.11 (t, *J* = 7.7 Hz, 1H), 1.67–1.56 (m, 1H), 1.53–1.43 (m, 1H), 1.41–1.29 (m, 0.9H), 1.13 (h, *J* = 7.6 Hz, 1H), 0.93 (t, *J* = 7.4 Hz, 1.3H), 0.79 (t, *J* = 7.7 Hz, 1.5H). Rotamers observed in approximately 1:1 ratio. ACQUITY UPLC BEH C18 1.7 μm: *R*_t_ = 2.01 min; *m*/*z* 428.2 [M + H, ^35^Cl]^+^, 430.1 [M + H, ^37^Cl]^+^.

#### *N*-Benzyl-*N*-butyl-3-chloro-4-(phenylsulfamoyl)benzamide (**20**)

Synthesized according to general procedure E. *N*-Benzyl-*N*-butyl-3-chloro-4-iodo-benzamide (278 mg, 0.65 mmol), potassium disulfite (289 mg, 1.30 mmol), tetrabutylammonium bromide (231 mg, 0.72 mmol), sodium formate (99 mg, 1.43 mmol), Pd(OAc)_2_ (7.4 mg, 0.03 mmol), PPh_3_ (26 mg, 0.10 mmol), 1,10-phenanthroline (18 mg, 0.10 mmol), aniline (0.59 mL, 6.50 mmol), NCS (174 mg, 1.30 mmol). Purified by flash column chromatography (silica, 12 g, 1:0 petrol/EtOAc to 1:1 petrol/EtOAc over 25 CV’s). Additionally purified by reverse-phase chromatography (9:1 H_2_O/MeOH to 0:1 H_2_O/MeOH over 20 min). Yield: 41 mg, 0.09 mmol, 13%. Cream solid. ^1^H NMR (500 MHz, CDCl_3_): δ 8.02 (d, *J* = 8.1 Hz, 0.5H), 7.93 (d, *J* = 8.1 Hz, 0.4H), 7.52 (s, 0.4H), 7.49 (s, 0.4H), 7.39–7.24 (m, 6H), 7.24–7.17 (m, 2H), 7.14–7.02 (m, 4H), 4.72 (s, 1H), 4.36 (s, 0.8H), 3.46 (t, *J* = 7.8 Hz, 0.8H), 3.00 (t, *J* = 7.8 Hz, 1H), 1.43 (p, *J* = 7.6 Hz, 0.8H),1.35 (h, *J* = 7.4 Hz, 0.6H), 1.05 (h, *J* = 7.4 Hz, 1H), 0.97–0.90 (m, 1.2H), 0.73 (t, *J* = 7.5 Hz, 1.4H). Rotamers observed in approximately 3:2 ratio. CH_2_ for one rotamer not observed as overlapping with HDO peak. ACQUITY UPLC BEH C18 1.7 μm: *R*_t_ = 1.88 min; *m*/*z* 457.2 [M + H, ^35^Cl]^+^, 459.2 [M + H, ^37^Cl]^+^.

#### *N*-Benzyl-4-bromo-*N*-butyl-2,3-difluoro-benzamide (**26d**)

Synthesized according to general procedure C. 4-Bromo-2,3-difluorobenzoic acid (200 mg, 0.84 mmol), HOBt hydrate (142 mg, 0.93 mmol), EDC.HCl (194 mg, 1.01 mmol), *N*-benzylbutylamine (0.23 mL, 1.27 mmol). Purified by flash column chromatography (silica, 12 g, 1:0 petrol/EtOAc to 0:1 petrol/EtOAc over 25 CV’s). Additionally purified by reverse-phase chromatography (9:1H_2_O/MeOH to 0:1H_2_O/MeOH over 25 min). Yield: 265 mg, 0.66 mmol, 78%. Orange oil. ^1^H NMR (500 MHz, CDCl_3_): δ 7.43–7.27 (m, 5H), 7.11 (d, *J* = 7.4 Hz, 1H), 7.07–7.00 (m, 1H), 4.79 (br s, 1.2H), 4.41 (s, 1H), 3.46 (br s, 1H), 3.08 (t, *J* = 7.6 Hz, 1.2H), 1.66–1.54 (m, 1H), 1.45 (p, *J* = 7.6 Hz, 1.3H), 1.36 (h, *J* = 7.4 Hz, 1H), 1.11 (h, *J* = 7.4 Hz, 1.2H), 0.93 (t, *J* = 7.4 Hz, 1.5H), 0.76 (t, *J* = 7.4 Hz, 1.8H). Rotamers observed in approximately 3:2 ratio. ACQUITY UPLCBEH C18 1.7 μm: *R*_t_ = 1.97 min; *m*/*z* 382.0 [M + H, ^79^Br]^+^, 384.0 [M + H, ^81^Br]^+^.

#### *N*-Benzyl-*N*-butyl-2-fluoro-4-(phenylsulfamoyl)benzamide (**21**)

Synthesized according to general procedure E. *N*-Benzyl-4-bromo-*N*-butyl-2,3-difluoro-benzamide (252 mg, 0.63 mmol), potassium disulfite (279 mg, 1.25 mmol), tetrabutylammonium bromide (223 mg, 0.69 mmol), sodium formate (95 mg, 1.38 mmol), Pd(OAc)_2_ (7.1 mg, 0.03 mmol), PPh_3_ (25 mg, 0.09 mmol), 1,10-phenanthroline (17 mg, 0.09 mmol), aniline (0.57 mL, 6.27 mmol), NCS (168 mg, 1.25 mmol). Purified by flash column chromatography (silica, 12 g, 1:0 petrol/EtOAc to 1:1 petrol/EtOAc over 30 CV’s). Additionally purified by reverse-phase chromatography (9:1 H_2_O/MeOH to 0:1 H_2_O/MeOH over 20 min). Yield: 52 mg, 0.11 mmol, 18%. Colorless glass. ^1^H NMR (500 MHz, CDCl_3_): δ 7.57 (d, *J* = 8.1 Hz, 0.7H), 7.53–7.40 (m, 2H), 7.40–7.21 (m, 5H), 7.20–7.12 (m, 1.2H), 7.09–7.01 (m, 3.6H), 4.77 (br s, 1.4H), 4.31 (s, 1H), 3.66–3.26 (m, 0.8H), 2.98 (t, *J* = 7.8 Hz, 1.4H), 1.60 (p, *J* = 8.0 Hz, 1.5H), 1.44–1.30 (m, 2.4H), 1.04 (h, *J* = 7.6 Hz, 1.4H), 0.93 (t, *J* = 7.4 Hz, 1.5H), 0.70 (t, *J* = 7.6 Hz, 2H). Rotamers observed in approximately 1:1 ratio. NH not observed. ^19^F NMR (470 MHz, CDCl_3_): δ −111.9 (s). ACQUITY UPLC CORTECS C18 1.7 μm: *R*_t_ = 1.79 min; *m*/*z* 441.2 [M + H]^+^.

#### *N*-Benzyl-4-bromo-*N*-butyl-2-methyl-benzamide (**26e**)

Synthesized according to general procedure C. 4-Bromo-2-methyl-benzoic acid (200 mg, 0.93 mmol), HOBt hydrate (157 mg, 1.02 mmol), EDC·HCl (214 mg, 1.12 mmol), *N*-benzylbutylamine (0.25 mL, 1.4 mmol). Purified by flash column chromatography (silica, 12 g, 1:0 petrol/EtOAc to 1:1 petrol/EtOAc over 25 CV’s). Yield: 318 mg, 0.84 mmol, 90%. Colorless oil. ^1^H NMR (500 MHz, CDCl_3_): δ 7.41–7.24 (m, 6H), 7.12–7.04 (m, 2H), 4.33 (s, 2H), 2.96 (t, *J* = 7.8 Hz, 2H), 2.31 (s, 1.4H), 2.27 (s, 1.6H), 1.66–1.60 (m, 1H), 1.44–1.34 (m, 2H), 1.07 (h, *J* = 7.5 Hz, 1H), 0.94 (t, *J* = 7.3 Hz, 1.5H), 0.74 (t, *J* = 7.3 Hz, 1.7H). Rotamers observed in approximately 1:1 ratio. CH_2_ α-protons to amide N for each rotamer appear to relax significantly differently. One is showing slow *T*_2_ relaxation (at 2.96 and 4.33 ppm) while the other has very fast *T*_2_ relaxation and is impossible to detect. This atropisomerism effect is due to effect of CH_3_ group as not observed in other analogues. 2.96 and 4.33 ppm peak integrations were set to 1H each. ACQUITY UPLCBEH C18 1.7 μm: *R*_t_ = 1.97 min; *m*/*z* 360.1 [M + H, ^79^Br]^+^, 362.1 [M + H, ^81^Br]^+^.

#### *N*-Benzyl-*N*-butyl-2-methyl-4-(phenylsulfamoyl)benzamide (**22**)

Synthesized according to general procedure E. *N*-Benzyl-4-bromo-*N*-butyl-2-methyl-benzamide (318 mg, 0.84 mmol), potassium disulfite (373 mg, 1.68 mmol), tetrabutylammonium bromide (299 mg, 0.92 mmol), sodium formate (127 mg, 1.85 mmol), Pd(OAc)_2_ (9.5 mg, 0.04 mmol), PPh_3_ (33 mg, 0.13 mmol), 1,10-phenanthroline (23 mg, 0.13 mmol), aniline (0.76 mL, 8.39 mmol), NCS (224 mg, 1.68 mmol). Purified by flash column chromatography (silica, 12 g, 1:0 petrol/EtOAc to 1:1 petrol/EtOAc over 30 CV’s). Additionally purified by reverse-phase chromatography (9:1 H_2_O/MeOH to 0:1 H_2_O/MeOH over 25 min). Yield: 68 mg, 0.15 mmol, 18%. Colorless glass. ^1^H NMR (500 MHz, CDCl_3_): δ 7.62–7.54 (m, 1.6H), 7.49 (dd, *J* = 8.0, 1.9 Hz, 0.5H), 7.37–7.34 (m, 2.4H), 7.33–7.27 (m, 1.4H), 7.25–7.20 (m, 3H), 7.16–7.10 (m, 1H), 7.07–6.99 (m, 3.3H), 6.51 (br s, 0.2H), 5.02 (br s, 0.5H), 4.50 (br s, 0.5H), 4.23 (s, 1H), 3.78 (br s, 0.4H), 3.20 (br s, 0.4H), 3.02–2.73 (m, 1.4H), 2.30 (s, 1.3H), 2.27 (s, 1.9H), 1.69–1.49 (m, 1H), 1.44–1.31 (m, 2.5H), 1.01 (h, *J* = 7.5 Hz, 1H), 0.94 (t, *J* = 7.3 Hz, 1.4H), 0.70 (t, *J* = 7.3 Hz, 1.8H). Rotamers and atropisomers observed in approximately 1:1:1:1 ratio. NH not observed. One rotamer effecting butyl chain protons (except for terminal CH_3_ group), causing these protons to exist in different chemical environments. ACQUITY UPLC BEH C18 1.7 μm: *R*_t_ = 1.86 min; *m*/*z* 437.2 [M + H]^+^.

#### *N*-Benzyl-5-bromo-*N*-butyl-pyridine-2-carboxamide (**28a**)

Synthesized according to general procedure C. 5-Bromo-2-pyridinecarboxylic acid (500 mg, 2.48 mmol), HOBt hydrate (417 mg, 2.72 mmol), EDC·HCl (569 mg, 2.97 mmol), *N*-benzylbutylamine (0.67 mL, 3.71 mmol). Purified by flash column chromatography (silica, 12 g, 1:0 petrol/EtOAc to 0:1 petrol/EtOAc over 25 CV’s). Yield: 242 mg, 0.86 mmol, 97% yield. Colorless oil. ^1^H NMR (500 MHz, CDCl_3_): δ 8.55 (d, *J* = 2.3 Hz, 0.5H), 8.50 (d, *J* = 2.3 Hz, 0.5H), 7.83 (dd, *J* = 8.4, 2.3 Hz, 0.5H), 7.77 (dd, *J* = 8.4, 2.3 Hz, 0.5H), 7.51–7.44 (m, 1H), 7.29–7.10 (m, 5H), 4.68 (s, 1H), 4.60 (s, 1H), 3.38–3.32 (m, 1H), 3.25–3.19 (m, 1H), 1.57–1.49 (m, 1H), 1.49–1.42 (m, 1H), 1.26 (h, *J* = 7.4 Hz, 1H), 1.03 (h, *J* = 7.4 Hz, 1H), 0.82 (t, *J* = 7.4 Hz, 1.5H), 0.68 (t, *J* = 7.4 Hz, 1.5H). Rotamers observed in approximately 1:1 ratio. ACQUITY UPLCBEH C18 1.7 μm: *R*_t_ = 1.89 min; *m*/*z* 347.0 [M + H, ^79^Br]^+^, 349.0 [M + H, ^81^Br]^+^.

#### *N*-Benzyl-*N*-butyl-5-(phenylsulfamoyl)pyridine-2-carboxamide (**23**)

Synthesized according to general procedure E. *N*-Benzyl-5-bromo-*N*-butyl-pyridine-2-carboxamide (100 mg, 0.29 mmol), potassium disulfite (128 mg, 0.58 mmol), tetrabutylammonium bromide (102 mg, 0.32 mmol), sodium formate (44 mg, 0.63 mmol), Pd(OAc)_2_ (3.3 mg, 0.01 mmol), PPh_3_ (11.3 mg, 0.04 mmol), 1,10-phenanthroline (7.8 mg, 0.04 mmol), aniline (0.26 mL, 2.88 mmol), NCS (77 mg, 0.58 mmol). Purified by automated column chromatography (silica, 4 g, 1:0 petrol/EtOAc +1% Et_3_N to 1:1 petrol/EtOAc +1% Et_3_N over 30 CVs). Yield: 31 mg, 0.07 mmol, 24%. Beige glass. ^1^H NMR (500 MHz, CDCl_3_): δ 8.88 (d, *J* = 2.3 Hz, 0.5H), 8.85 (d, *J* = 2.3 Hz, 0.5H), 8.07 (dd, *J* = 8.2, 2.3 Hz, 0.5H), 7.99 (dd, *J* = 8.2, 2.3 Hz, 0.5H), 7.68 (d, *J* = 8.2 Hz, 0.5H), 7.61 (d, *J* = 8.2 Hz, 0.5H), 7.38–7.12 (m, 8H), 7.10–7.03 (m, 2.2H), 6.79 (br s, 1H), 4.76 (s, 1H), 4.56 (s, 1H), 3.51–3.42 (m, 1H), 3.24–3.15 (m, 1H), 1.66–1.58 (m, 1H), 1.49 (p, *J* = 7.7 Hz, 1H), 1.35 (h, *J* = 7.4 Hz, 1H), 1.07 (h, *J* = 7.4 Hz, 1H), 0.92 (t, *J* = 7.4 Hz, 1.7H), 0.73 (t, *J* = 7.4 Hz, 1.6H). Rotamers observed in approximately 1:1 ratio. ACQUITY UPLC BEH C18 1.7 μm: *R*_t_ = 1.82 min; *m*/*z* 424.2 [M + H]^+^.

#### *N*-Benzyl-*N*-butyl-2-chloro-pyrimidine-5-carboxamide (**28b**)

A solution of 2-chloropyrimidine-5-carboxylic acid (200 mg, 1.26 mmol), propylphosphonic anhydride (1.13 mL, 1.89 mmol) and Et_3_N (0.53 mL, 3.78 mmol) in DMF (3 mL) was stirred at room temperature for 30 min. *N*-Benzylbutylamine (0.23 mL, 1.26 mmol) was then added and the reaction mixture stirred at room temperature for 1 h. The reaction mixture was diluted with DCM (20 mL) and washed with water (2 × 20 mL) and brine (20 mL). The organic layer was dried over MgSO_4_, filtered, concentrated under reduced pressure and the residue purified by automated column chromatography (silica, 12 g, 1:0 petrol/EtOAc to 1:1 petrol/EtOAc over 25 CV’s). Fractions containing product were combined and concentrated under reduced pressure to afford *N*-benzyl-*N*-butyl-2-chloro-pyrimidine-5-carboxamide (133 mg, 0.42 mmol, 33% yield) as a light orange oil. ^1^H NMR (500 MHz, CDCl_3_): δ 8.71 (s, 1H), 8.64 (s, 1H), 7.42–7.28 (m, 4H), 7.18–7.08 (m, 1H), 4.76 (s, 1H), 4.51 (s, 1H), 3.51 (t, *J* = 8.2 Hz, 1H), 3.15 (t, *J* = 8.0 Hz, 1H), 1.71–1.60 (m, 1H), 1.57–1.47 (m, 1H), 1.43–1.30 (m, 1H), 1.22–1.10 (m, 1H), 0.95 (t, *J* = 7.4 Hz, 1.5H), 0.81 (t, *J* = 7.4 Hz, 1.5H). Rotamers observed in approximately 1:1 ratio. ACQUITY UPLCBEH C18 1.7 μm: *R*_t_ = 1.77 min; *m*/*z* 304.1 [M + H, ^35^Cl]^+^, 306.1 [M + H, ^37^Cl]^+^.

#### *N*-Benzyl-*N*-butyl-2-(phenylsulfamoyl)pyrimidine-5-carboxamide (**24**)

Synthesized according to general procedure E. *N*-Benzyl-*N*-butyl-2-chloro-pyrimidine-5-carboxamide (133 mg, 0.44 mmol), potassium disulfite (195 mg, 0.88 mmol), tetrabutylammonium bromide (156 mg, 0.48 mmol), sodium formate (67 mg, 0.97 mmol), Pd(OAc)_2_ (5 mg, 0.02 mmol), PPh_3_ (17 mg, 0.07 mmol), 1,10-phenanthroline (12 mg, 0.07 mmol), aniline (0.40 mL, 4.39 mmol), NCS (117 mg, 0.88 mmol). Purified by flash column chromatography (silica, 12 g, 1:0 petrol/EtOAc to 1:1 petrol/EtOAc over 25 CV’s). Additionally purified by reverse-phase chromatography (9:1 H_2_O/MeOH to 0:1 H_2_O/MeOH over 25 min). Yield: 14 mg, 0.03 mmol, 7%. Light yellow solid. ^1^H NMR (500 MHz, CDCl_3_): δ 8.90 (s, 1H), 8.81 (s, 1H), 7.41–7.29 (m, 4.4H), 7.27–7.17 (m, 5H), 7.16–7.10 (m, 0.8H), 7.10–7.03 (m, 1H), 4.75 (s, 1H), 4.45 (s, 1H), 3.59–3.49 (m, 1H), 3.13–3.03 (m, 1H), 1.71–1.62 (m, 1H), 1.37 (h, *J* = 8.3 Hz, 1H), 1.10 (h, *J* = 7.7 Hz, 1H), 0.95 (t, *J* = 7.3 Hz, 1.5H), 0.77 (t, *J* = 7.4 Hz, 1.5H). Rotamers observed in approximately 1:1 ratio. CH_2_ for one rotamer not observed as overlapping with HDO peak. ACQUITY UPLC BEH C18 1.7 μm: *R*_t_ = 1.78 min; *m*/*z* 425.1 [M + H]^+^.

#### Methyl 5-(Phenylsulfamoyl)furan-3-carboxylate (**32**)

Synthesized according to general procedure A. 5-(Chlorosulfonyl)furan-3-carboxylate (250 mg, 1.11 mmol), aniline (0.51 mL, 5.56 mmol). Purified by flash column chromatography (silica, 12 g, 1:0 petrol/EtOAc to 1:1 petrol/EtOAc). Yield: 217 mg, 0.73 mmol, 66% yield. Cream solid. ^1^H NMR (400 MHz, CDCl_3_): δ 8.05 (s, 1H), 7.34–7.27 (m, 3H), 7.21–7.16 (m, 1H), 7.15–7.11 (m, 2H), 6.83 (br s, 1H), 3.83 (s, 3H). ACQUITY UPLC BEH C18 1.7 μm: *R*_t_ = 1.59 min; *m*/*z* 280.0 [M – H]^−^.

#### 5-(Phenylsulfamoyl)furan-3-carboxylic Acid (**33**)

Methyl 5-(phenylsulfamoyl)furan-3-carboxylate (194 mg, 0.690 mmol) was dissolved in 1:1:3 MeOH/THF/H_2_O (1.5, 1.5, 4.5 mL, respectively), to which lithium hydroxide monohydrate (103 mg, 1.38 mmol) was added. The reaction mixture was stirred at room temperature overnight. The reaction mixture was concentrated under reduced pressure. 1 M HCl (5 mL) was added and a solid immediately precipitated. The precipitate was filtered, washed with water (10 mL) and dried to afford 5-(phenylsulfamoyl)furan-3-carboxylic acid (183 mg, 0.65 mmol, 95% yield) as a cream solid. ^1^H NMR (500 MHz, DMSO-*d*_6_): δ 13.20 (br s, 1H), 10.81 (br s, 1H), 8.57 (s, 1H), 7.29 (t, *J* = 7.8 Hz, 2H), 7.24 (s, 1H), 7.08–7.16 (m, 3H). ACQUITY UPLC BEH C18 1.7 μm: *R*_t_ = 1.44 min; *m*/*z* 265.9 [M – H]^−^.

#### *N*-Benzyl-*N*-butyl-5-(phenylsulfamoyl)furan-3-carboxamide (**30**)

Synthesized according to general procedure C. 5-(Phenylsulfamoyl)furan-3-carboxylic acid (171 mg, 0.61 mmol), HOBt hydrate (103 mg, 0.67 mmol), EDC.HCl (140 mg, 0.73 mmol), *N*-benzylbutylamine (0.17 mL, 0.98 mmol). Purified by flash column chromatography (silica, 12 g, 1:0 petrol/EtOAc to 0:1 petrol/EtOAc over 25 CV’s). Yield: 236 mg, 0.54 mmol, 89%. Colorless glass. ^1^H NMR (500 MHz, CDCl_3_): δ 7.81 (br s, 0.4H), 7.61 (s, 0.5H), 7.42–7.02 (m, 11H), 6.95 (br s, 1H), 4.68 (s, 0.7H), 4.55 (s, 1H), 3.51–3.32 (m, 1H), 3.28–3.09 (m, 0.8H), 1.69–1.42 (m, 2H), 1.36–1.23 (m, 1.2H), 1.21–1.08 (m, 0.8H), 0.98–0.75 (m, 3H). Rotamers observed in approximately 3:2 ratio. ACQUITY UPLC BEH C18 1.7 μm: *R*_t_ = 1.82 min; *m*/*z* 413.1 [M + H]^+^.

#### *N*-Benzyl-*N*-butyl-4-(*N*-methylsulfamoyl)benzamide (**34**)

Synthesized according to general procedure A. 4-[Benzyl(butyl)carbamoyl]benzenesulfonyl chloride (100 mg, 0.27 mmol, Key Intermediate C), methylamine (2 M in THF, 0.15 mL, 0.33 mmol, 1.2 equiv used). Et_3_N (46 μL, 0.33 mmol) was also added. The reaction was conducted in DCM (5 mL) and stirred at room temperature for 1 h. Saturated NaHCO_3_ (2 mL) was added, mixed vigorously and organic phase separated using a phase separator. Purified by flash column chromatography (silica, 12 g, 1:0 petrol/EtOAc to 0:1 petrol/EtOAc over 25 CV’s). Yield: 81 mg, 0.21 mmol, 78%. Colorless glass. ^1^H NMR (500 MHz, CDCl_3_): δ 7.90 (d, *J* = 7.9 Hz, 1H), 7.82 (d, *J* = 7.9 Hz, 1H), 7.57–7.47 (m, 2H), 7.40–7.27 (m, 4H), 7.12 (d, *J* = 7.3 Hz, 1H), 4.77 (s, 1H), 4.44 (s, 1H), 3.47 (t, *J* = 7.9 Hz, 1H), 3.08 (t, *J* = 7.7 Hz, 1H), 2.62 (d, *J* = 8.6 Hz, 3H), 1.69–1.56 (m, 1H), 1.53–1.37 (m, 2H), 1.37–1.27 (m, 1H), 1.17–1.01 (m, 1H), 0.93 (t, *J* = 7.9 Hz, 1.5H), 0.74 (t, *J* = 7.7 Hz, 1.5H). Rotamers observed in approximately 1:1 ratio. ACQUITY UPLC BEH C18 1.7 μm: *R*_t_ = 1.72 min; *m*/*z* 361.1 [M + H]^+^.

#### *N*-Benzyl-*N*-butyl-4-(*N*-(pyridin-4-yl)sulfamoyl)benzamide (**35**)

Synthesized according to general procedure A. 4-[Benzyl(butyl)carbamoyl]benzenesulfonyl chloride (100 mg, 0.27 mmol, Key Intermediate C), 4-aminopyridine (28 mg, 0.30 mmol), 1.1 equiv used). Et_3_N (46 μL, 0.33 mmol) was also added. The reaction mixture was diluted with EtOAc (30 mL), washed with saturated NaHCO_3_ (3 × 20 mL) and water (20 mL). The organic phase was dried over MgSO_4_, filtered and concentrated under reduced pressure. Purified by flash column chromatography (silica, 12 g, 1:0 petrol/EtOAc to 0:1 petrol/EtOAc over 25 CV’s), followed by additional purification by flash column chromatography (silica, 12 g, 1:0 DCM/MeOH to 9:1 DCM/MeOH). Yield: 47 mg, 0.11 mmol, 39%. Colorless solid. ^1^H NMR (500 MHz, MeOD-*d*_4_): δ 8.02 (d, *J* = 7.9 Hz, 1H), 7.99–7.92 (m, 3H), 7.56 (d, *J* = 8.0 Hz, 1H), 7.52 (d, *J* = 8.0 Hz, 1H), 7.40–7.34 (m, 2H), 7.34–7.24 (m, 2H), 7.16–7.05 (m, 3H), 4.77 (s, 1.1H), 4.47 (s, 0.9H), 3.46 (t, *J* = 7.7 Hz, 0.8H), 3.13 (t, *J* = 7.8 Hz, 1.1H), 1.62 (p, *J* = 7.7 Hz, 1H), 1.45 (p, *J* = 7.6 Hz, 1H), 1.36 (h, *J* = 7.5 Hz, 1H), 1.03 (h, *J* = 7.4 Hz, 1H), 0.94 (t, *J* = 7.4 Hz, 1.3H), 0.65 (t, *J* = 7.4 Hz, 1.7H). Rotamers observed in 1:1 ratio. ACQUITY UPLC CORTECS C18 1.7 μm: *R*_t_ = 1.54 min; *m*/*z* 424.3 [M + H]^+^.

#### *N*-Benzyl-*N*-butyl-4-(isoxazol-4-ylsulfamoyl)benzamide (**36**)

Synthesized according to general procedure A. 4-[Benzyl(butyl)carbamoyl]benzenesulfonyl chloride (100 mg, 0.27 mmol, Key Intermediate C), 4-aminoisoxazole (30 mg, 0.36 mmol, 1.3 equiv used). Et_3_N (46 μL, 0.33 mmol) was also added. Reaction conducted in DCM (5 mL) and at 40 °C for 4 h. Saturated NaHCO_3_ (3 mL) was added, mixed vigorously and organic phase separated using a phase separator. Purified by flash column chromatography (silica, 4 g, 1:0 petrol/EtOAc to 1:1 petrol/EtOAc), followed by additional purification by flash column chromatography (silica, 4 g, 3:2 petrol/EtOAc to 0:1 petrol/EtOAc). Yield: 21 mg, 0.05 mmol, 17%. Yellow glass. ^1^H NMR (500 MHz, CDCl_3_): δ 8.32 (d, *J* = 10.5 Hz, 1H), 8.14 (d, *J* = 9.8 Hz, 1H), 7.77 (d, *J* = 7.9 Hz, 1H), 7.69 (d, *J* = 8.0 Hz, 1H), 7.53–7.45 (m, 2H), 7.40–7.28 (m, 4H), 7.15–7.07 (m, 1H), 6.75 (br s, 1H), 4.76 (s, 1H), 4.41 (s, 1H), 3.48 (t, *J* = 7.5 Hz, 1H), 3.06 (t, *J* = 7.8 Hz, 1H), 1.63 (p, *J* = 7.5 Hz, 1H), 1.46 (p, *J* = 7.4 Hz, 1H), 1.40–1.32 (m, 1H), 1.08 (h, *J* = 7.5 Hz, 1H), 0.94 (t, *J* = 7.4 Hz, 1.5H), 0.74 (t, *J* = 7.4 Hz, 1.5H). Rotamers observed in 1:1 ratio. ACQUITY UPLC BEH C18 1.7 μm: *R*_t_ = 1.74 min; *m*/*z* 414.1 [M + H]^+^.

#### *N*-Benzyl-*N*-butyl-4-(*N*-cyclobutylsulfamoyl)benzamide (**37**)

Synthesized according to general procedure A. 4-[Benzyl(butyl)carbamoyl]benzenesulfonyl chloride (100 mg, 0.27 mmol, Key Intermediate C), cyclobutylamine (19 mg, 0.27 mmol, 1 equiv used). Et_3_N (46 μL, 0.33 mmol) was also added. The reaction was conducted in DCM (5 mL) and stirred at room temperature for 2 h. Saturated NaHCO_3_ (2 mL) was added, mixed vigorously and organic phase separated using a phase separator. Purified by flash column chromatography (silica, 4 g, 1:0 petrol/EtOAc to 0:1 petrol/EtOAc over 25 CV’s). Yield: 72 mg, 0.17 mmol, 63%. Colorless glass. ^1^H NMR (500 MHz, CDCl_3_): δ 7.91 (d, *J* = 7.9 Hz, 1.1H), 7.83 (d, *J* = 8.0 Hz, 0.9H), 7.52 (dd, *J* = 10.9, 7.9 Hz, 2H), 7.40–7.27 (m, 4H), 7.12 (d, *J* = 7.4 Hz, 1H), 4.88 (d, *J* = 8.8 Hz, 0.5H), 4.84 (d, *J* = 8.7 Hz, 0.3H), 4.77 (s, 1.1H), 4.43 (s, 0.9H), 3.85–3.71 (m, 1H), 3.49 (t, *J* = 7.4 Hz, 0.9H), 3.07 (t, *J* = 7.5 Hz, 1H), 2.16–2.07 (m, 2H), 1.83–1.69 (m, 2H), 1.68–1.50 (m, 1H), 1.46 (p, *J* = 7.5 Hz, 1H), 1.37 (h, *J* = 7.4 Hz, 0.7H), 1.07 (h, *J* = 7.5 Hz, 1H), 0.95 (t, *J* = 7.4 Hz, 1.4H), 0.74 (t, *J* = 7.3 Hz, 1.6H). Rotamers observed in approximately 1:1 ratio. ACQUITY UPLC BEH C18 1.7 μm: *R*_t_ = 1.82 min; *m*/*z* 401.3 [M + H]^+^.

#### *N*-Benzyl-*N*-butyl-4-(*N*-(oxetan-3-yl)sulfamoyl)benzamide (**38**)

Synthesized according to general procedure A. 4-[Benzyl(butyl)carbamoyl]benzenesulfonyl chloride (100 mg, 0.27 mmol, Key Intermediate C), 3-oxetanamine (20 mg, 0.27 mmol, 1 equiv used). Et_3_N (57 μL, 0.41 mmol) was also added. The reaction was conducted in DCM (3 mL) and stirred at room temperature for 1 h. Saturated NaHCO_3_ (2 mL) was added, mixed vigorously and organic phase separated using a phase separator. Purified by flash column chromatography (silica, 12 g, 1:0 petrol/EtOAc to 0:1 petrol/EtOAc over 25 CV’s). Yield: 66 mg, 0.16 mmol, 57%. Colorless glass, which produced a solid upon scratching. ^1^H NMR (500 MHz, CDCl_3_): δ 7.86 (d, *J* = 7.9 Hz, 1H), 7.78 (d, *J* = 8.0 Hz, 1H), 7.56–7.47 (m, 2H), 7.41–7.28 (m, 4H), 7.12 (d, *J* = 7.4 Hz, 1H), 5.74 (d, *J* = 8.9 Hz, 0.5H), 5.67 (d, *J* = 9.0 Hz, 0.5H), 4.77 (s, 1.1H), 4.73–4.64 (m, 2H), 4.57–4.45 (m, 1H), 4.43 (s, 0.9H), 4.38–4.30 (m, 2H), 3.50 (t, *J* = 7.8 Hz, 1H), 3.08 (t, *J* = 7.7 Hz, 1H), 1.69–1.63 (m, 1H), 1.52–1.42 (m, 1H), 1.42–1.32 (m, 1H), 1.09 (h, *J* = 7.6 Hz, 1H), 0.95 (t, *J* = 7.3 Hz, 1.4H), 0.75 (t, *J* = 7.4 Hz, 1.6H). Rotamers observed in approximately 1:1 ratio. ACQUITY UPLC BEH C18 1.7 μm: *R*_t_ = 1.70 min; *m*/*z* 403.2 [M + H]^+^.

#### *N*-(4-Fluorobenzyl)butan-1-amine

Synthesized according to general procedure B. 4-Fluorobenzylamine (91 μL, 0.80 mmol), butyraldehyde (58 μL, 0.64 mmol), NaHCO_3_ (100 mg, 1.20 mmol), NaBH_4_ (36 mg, 0.96 mmol, 1.5 equiv used). The reduction was conducted at 0 °C for 4 h, allowing to warm to room temperature. Crude product taken to next step without further purification.

#### *N*-Butyl-*N*-(4-fluorobenzyl)-4-(*N*-phenylsulfamoyl)benzamide (**39**)

Synthesized according to general procedure C. 4-(*N*-Phenylsulfamoyl)benzoic acid (110 mg, 0.40 mmol), HOBt hydrate (86 mg, 0.56 mmol, 1.4 equiv used), EDC.HCl (122 mg, 0.64 mmol, 1.6 equiv used), *N*-(4-fluorobenzyl)butan-1-amine (crude as above), DCM (5 mL). The reaction mixture was directly concentrated under reduced pressure and purified by flash column chromatography (silica, 24 g, 1:0 petrol/EtOAc to 0:1 petrol/EtOAc). Yield: 104 mg, 0.24 mmol, 28%. Colorless glass. IR (neat) ν_max_/cm^–1^: 3144, 2959, 2932, 2874, 1614, 1155. ^1^H NMR (500 MHz, MeOD-*d*_4_): δ 7.84 (d, *J* = 8.0 Hz, 1.3H), 7.77 (d, *J* = 8.0 Hz, 0.9H), 7.52 (d, *J* = 8.0 Hz, 1.2H), 7.46 (d, *J* = 8.0 Hz, 0.8H), 7.39 (dd, *J* = 8.3, 5.5 Hz, 1H), 7.25–7.14 (m, 2H), 7.14–6.95 (m, 6H), 4.72 (s, 1.4H), 4.39 (s, 0.9H), 3.57–3.38 (m, 0.9H), 3.07 (t, *J* = 7.8 Hz, 1.4H), 1.73–1.53 (m, 1H), 1.54–1.21 (m, 1.6H), 1.01 (h, *J* = 7.5 Hz, 1.1H), 0.94 (t, *J* = 7.3 Hz, 1.2H), 0.67 (t, *J* = 7.4 Hz, 2H). NH not observed. Rotamers observed in approximately 3:2 ratio. ^13^C NMR (151 MHz, CDCl_3_): δ 170.5, 163.1, 161.5, 140.7, 140.3, 140.2, 136.3, 132.7, 131.9, 129.9, 129.4, 128.4, 127.5, 127.1, 125.6, 122.1, 116.0, 115.9, 115.8, 115.6, 51.8, 48.0, 47.0, 44.9, 30.2, 29.1, 20.2, 19.6, 13.8, 13.5. ACQUITY UPLC BEH C18 1.7 μm: *R*_t_ = 1.85 min; *m*/*z* 441.1 [M + H]^+^. HRMS (EI) calcd for C_24_H_25_O_3_N_2_FS, 440.1564; found, 440.1559.

#### *N*-(4-Methoxybenzyl)butan-1-amine

Synthesized according to general procedure B. 4-Methoxybenzylamine (95 μL, 0.73 mmol), butyraldehyde (58 μL, 0.64 mmol), NaHCO_3_ (100 mg, 1.20 mmol), NaBH_4_ (36 mg, 0.96 mmol, 1.5 equiv used). The reduction was conducted at 0 °C for 4 h, allowing to warm to room temperature. Crude product taken to next step without further purification.

#### *N*-Butyl-*N*-(4-methoxybenzyl)-4-(*N*-phenylsulfamoyl)benzamide (**40**)

Synthesized according to general procedure C. 4-(*N*-Phenylsulfamoyl)benzoic acid (110 mg, 0.40 mmol), HOBt hydrate (86 mg, 0.56 mmol, 1.4 equiv used), EDC.HCl (122 mg, 0.64 mmol, 1.6 equiv used), *N*-(4-methoxybenzyl)butan-1-amine (crude as above), DCM (5 mL). The reaction mixture was directly concentrated under reduced pressure and purified by flash column chromatography (silica, 24 g, 1:0 petrol/EtOAc to 0:1 petrol/EtOAc). Yield: 110 mg, 0.24 mmol, 29%. Colorless glass. IR (neat) ν_max_/cm^–1^: 3156, 2957, 2932, 2874, 1611, 1163. ^1^H NMR (500 MHz, MeOD-*d*_4_): δ 7.84 (d, *J* = 8.0 Hz, 1H), 7.78 (dd, *J* = 8.2, 5.2 Hz, 1H), 7.53–7.42 (m, 2H), 7.29 (d, *J* = 8.2 Hz, 1H), 7.23–7.13 (m, 2H), 7.10–7.01 (m, 3H), 6.99 (d, *J* = 8.3 Hz, 1H), 6.91 (d, *J* = 8.3 Hz, 1H), 6.85 (d, *J* = 8.2 Hz, 1H), 4.68 (s, 1.3H), 4.33 (s, 0.9H), 3.78 (d, *J* = 8.8 Hz, 3H), 3.55–3.38 (m, 1H), 3.13–2.86 (m, 1.2H), 1.60 (p, *J* = 7.7 Hz, 1H), 1.46–1.30 (m, 2H), 1.00 (h, *J* = 7.4 Hz, 1H), 0.94 (t, *J* = 7.3 Hz, 1.2H), 0.67 (t, *J* = 7.4 Hz, 1.7H). NH not observed. Rotamers observed in approximately 3:2 ratio. ^13^C NMR (151 MHz, CDCl_3_): δ 170.3, 159.2, 159.2, 141.1, 140.1, 139.9, 136.2, 129.6, 129.4, 128.9, 128.0, 127.5, 127.4, 127.2, 127.1, 125.8, 125.7, 122.2, 122.0, 114.3, 114.2, 55.3, 51.9, 47.6, 46.9, 44.7, 30.2, 29.1, 20.2, 19.6, 13.9, 13.5. ACQUITY UPLC BEH C18 1.7 μm: *R*_t_ = 1.75 min; *m*/*z* 453.1 [M + H]^+^. HRMS (EI) calcd for C_25_H_28_O_4_N_2_S, 452.1764; found, 452.1762.

#### *N*-(3-Methoxybenzyl)butan-1-amine

Synthesized according to general procedure B. 3-Methoxybenzylamine (93 μL, 0.73 mmol), butyraldehyde (58 μL, 0.64 mmol), NaHCO_3_ (100 mg, 1.20 mmol), NaBH_4_ (36 mg, 0.96 mmol, 1.5 equiv used). The reduction was conducted at 0 °C for 4 h, allowing to warm to room temperature. Crude product taken to next step without further purification.

#### *N*-Butyl-*N*-(3-methoxybenzyl)-4-(*N*-phenylsulfamoyl)benzamide (**41**)

Synthesized according to general procedure C. 4-(*N*-Phenylsulfamoyl)benzoic acid (110 mg, 0.40 mmol), HOBt hydrate (86 mg, 0.56 mmol, 1.4 equiv used), EDC·HCl (122 mg, 0.64 mmol, 1.6 equiv used), *N*-(3-methoxybenzyl)butan-1-amine (crude as above), DCM (5 mL). The reaction mixture was directly concentrated under reduced pressure and purified by flash column chromatography (silica, 24 g, 1:0 DCM/1% NH_3_ in MeOH to 9:1 DCM/1% NH_3_ in MeOH). Yield: 91 mg, 0.20 mmol, 24%. Colorless glass. IR (neat) ν_max_/cm^–1^: 3146, 2959, 2934, 2874, 1599, 1163. ^1^H NMR (500 MHz, MeOD-*d*_4_): δ 7.85 (d, *J* = 8.3 Hz, 1.1H), 7.81–7.72 (m, 0.9H), 7.57–7.49 (m, 1.2H), 7.47 (d, *J* = 8.1 Hz, 0.8H), 7.32–7.12 (m, 3H), 7.11–6.99 (m, 3H), 6.93 (d, *J* = 8.2 Hz, 1H), 6.90–6.80 (m, 1H), 6.71–6.58 (m, 1H), 4.72 (s, 1.2H), 4.38 (s, 1H), 3.82–3.74 (m, 3H), 3.60–3.39 (m, 1H), 3.20–2.99 (m, 1.2H), 1.62 (p, *J* = 7.7 Hz, 1H), 1.54–1.26 (m, 2H), 1.02 (h, *J* = 7.4 Hz, 1H), 0.94 (t, *J* = 7.4 Hz, 1.3H), 0.67 (t, *J* = 7.4 Hz, 1.7H). NH not observed. Rotamers observed in approximately 3:2 ratio. ^13^C NMR (151 MHz, CDCl_3_): δ 170.4, 170.3, 160.1, 160.0, 141.0, 140.9, 140.2, 140.0, 138.4, 137.9, 136.2, 130.1, 129.8, 129.4, 127.5, 127.4, 127.2, 127.1, 125.8, 125.7, 122.1, 122.0, 120.3, 118.8, 113.9, 112.9, 112.8, 112.6, 55.3, 52.3, 48.0, 47.4, 45.0, 30.2, 29.1, 20.2, 19.6, 13.9, 13.5. ACQUITY UPLC BEH C18 1.7 μm: *R*_t_ = 1.85 min; *m*/*z* 453.1 [M + H]^+^. HRMS (EI) calcd for C_25_H_28_O_4_N_2_S, 452.1764; found, 452.1760.

#### *N*-(2-Methoxybenzyl)butan-1-amine

Synthesized according to general procedure B. 2-Methoxybenzylamine (95 μL, 0.73 mmol), butyraldehyde (58 μL, 0.64 mmol), NaHCO_3_ (100 mg, 1.20 mmol), NaBH_4_ (36 mg, 0.96 mmol, 1.5 equiv used). The reduction was conducted at 0 °C for 4 h, allowing to warm to room temperature. Crude product taken to next step without further purification.

#### *N*-Butyl-*N*-(2-methoxybenzyl)-4-(*N*-phenylsulfamoyl)benzamide (**42**)

Synthesized according to general procedure C. 4-(*N*-Phenylsulfamoyl)benzoic acid (110 mg, 0.40 mmol), HOBt hydrate (86 mg, 0.56 mmol, 1.4 equiv used), EDC·HCl (122 mg, 0.64 mmol, 1.6 equiv used), *N*-(2-methoxybenzyl)butan-1-amine (crude as above), DCM (5 mL). The reaction mixture was directly concentrated under reduced pressure and purified by flash column chromatography (silica, 24 g, 1:0 petrol/EtOAc to 0:1 petrol/EtOAc). Yield: 73 mg, 0.16 mmol, 19%. Colorless glass. mp 150–152 °C; IR (neat) ν_max_/cm^–1^ 3154, 2959, 2932, 2874, 1616, 1165. ^1^H NMR (500 MHz, MeOD-*d*_4_): δ 7.84 (d, *J* = 8.3 Hz, 0.8H), 7.77 (d, *J* = 8.1 Hz, 1.1H), 7.49 (t, *J* = 8.4 Hz, 2H), 7.34–7.23 (m, 1H), 7.23–7.12 (m, 2H), 7.11–6.87 (m, 6H), 4.75 (s, 0.9H), 4.38 (s, 1.2H), 3.86 (s, 1.2H), 3.68 (s, 1.6H), 3.51–3.36 (m, 1.1H), 3.13–2.90 (m, 0.9H), 1.60–1.49 (m, 1.1H), 1.48–1.38 (m, 0.9H), 1.33 (h, *J* = 7.4 Hz, 1.2H), 1.01 (h, *J* = 7.4 Hz, 0.8H), 0.91 (t, *J* = 7.4 Hz, 1.8H), 0.67 (t, *J* = 7.4 Hz, 1.2H). NH not observed. Rotamers observed in approximately 3:2 ratio. ^13^C NMR (151 MHz, CDCl_3_): δ 170.5, 170.2, 157.6, 157.1, 141.4, 141.2, 139.9, 139.8, 136.2, 129.5, 129.4, 129.0, 128.8), 127.6, 127.4, 127.2, 127.1, 125.8, 125.7, 124.8, 124.3, 122.2, 122.1, 120.8, 120.6, 110.4, 110.4, 55.3, 55.1, 48.2, 48.1, 45.0, 42.2, 30.3, 29.2, 20.2, 19.7, 13.9, 13.6. ACQUITY UPLC BEH C18 1.7 μm: *R*_t_ = 1.86 min; *m*/*z* 453.1 [M + H]^+^. HRMS (EI) calcd for C_25_H_28_O_4_N_2_S, 452.1764; found, 452.1766.

#### *N*-(Pyridin-4-ylmethyl)butan-1-amine

Synthesized according to general procedure B. 4-(Aminomethyl)pyridine (94 μL, 0.73 mmol), butyraldehyde (58 μL, 0.64 mmol), NaHCO_3_ (100 mg, 1.20 mmol), NaBH_4_ (36 mg, 0.96 mmol, 1.5 equiv used). The reduction was conducted at 0 °C for 4 h, allowing to warm to room temperature. Crude product taken to next step without further purification.

#### *N*-Butyl-4-(*N*-phenylsulfamoyl)-*N*-(pyridin-4-ylmethyl)benzamide (**43**)

Synthesized according to general procedure C. 4-(*N*-Phenylsulfamoyl)benzoic acid (110 mg, 0.40 mmol), HOBt hydrate (86 mg, 0.56 mmol, 1.4 equiv used), EDC·HCl (122 mg, 0.64 mmol, 1.6 equiv used), *N*-(pyridin-4-ylmethyl)butan-1-amine (crude as above), DCM (5 mL). The reaction mixture was directly concentrated under reduced pressure and purified by flash column chromatography (silica, 24 g, 1:0 DCM/1% NH_3_ in MeOH to 9:1 DCM/1% NH_3_ in MeOH). Additionally purified by reverse-phase chromatography (9:1 H_2_O/MeOH to 0:1 H_2_O/MeOH). Yield: 87 mg, 0.21 mmol, 24%. Colorless glass. mp 91–94 °C; IR (neat) ν_max_/cm^–1^: 3057, 2959, 2932, 2874, 1622, 1161. ^1^H NMR (500 MHz, MeOD-*d*_4_): δ 8.51 (d, *J* = 5.2 Hz, 1.2H), 8.48–8.41 (m, 0.7H), 7.89–7.80 (m, 1.4H), 7.74 (d, *J* = 8.0 Hz, 0.6H), 7.59 (d, *J* = 8.0 Hz, 1H), 7.47–7.36 (m, 2H), 7.24–7.13 (m, 3H), 7.13–6.97 (m, 3H), 4.79 (s, 1.4H), 4.49 (s, 0.6H), 3.52 (t, *J* = 7.7 Hz, 0.6H), 3.23–3.04 (m, 1.4H), 1.73–1.62 (m, 1H), 1.46 (p, *J* = 7.7 Hz, 1H), 1.42–1.32 (m, 1H), 1.05 (h, *J* = 7.3 Hz, 1H), 1.01–0.88 (m, 1H), 0.69 (t, *J* = 7.3 Hz, 2H). NH not observed. Rotamers observed in approximately 2:1 ratio. ^13^C NMR (151 MHz, CDCl_3_): δ 170.6, 150.3, 150.1, 146.2, 140.4, 140.4, 136.2, 129.4, 127.6, 127.2, 127.0, 125.8, 125.7, 122.7, 122.1, 121.9, 121.5, 51.5, 49.0, 47.2, 45.5, 30.5, 29.1, 20.1, 19.6, 13.8, 13.5. ACQUITY UPLC BEH C18 1.7 μm: *R*_t_ = 1.48 min; *m*/*z* 424.1 [M + H]^+^. HRMS (EI) calcd for C_23_H_25_O_3_N_3_S, 423.1611; found, 423.1617.

#### *N*-(Pyridin-3-ylmethyl)butan-1-amine

Synthesized according to general procedure B. 3-Picolylamine (94 μL, 0.73 mmol), butyraldehyde (58 μL, 0.64 mmol), NaHCO_3_ (100 mg, 1.20 mmol), NaBH_4_ (36 mg, 0.96 mmol, 1.5 equiv used). The reduction was conducted at 0 °C for 4 h, allowing to warm to room temperature. Crude product taken to next step without further purification.

#### *N*-Butyl-4-(*N*-phenylsulfamoyl)-*N*-(pyridin-3-ylmethyl)benzamide (**44**)

Synthesized according to general procedure C. 4-(*N*-Phenylsulfamoyl)benzoic acid (110 mg, 0.40 mmol), HOBt hydrate (86 mg, 0.56 mmol, 1.4 equiv used), EDC.HCl (122 mg, 0.64 mmol, 1.6 equiv used), *N*-(pyridin-3-ylmethyl)butan-1-amine (crude as above), DCM (5 mL). The reaction mixture was directly concentrated under reduced pressure and purified by flash column chromatography (silica, 24 g, 1:0 DCM/1% NH_3_ in MeOH to 9:1 DCM/1% NH_3_ in MeOH). Additionally purified by reverse-phase chromatography (9:1 H_2_O/MeOH to 0:1 H_2_O/MeOH). Yield: 99 mg, 0.23 mmol, 28%. Colorless glass. IR (neat) ν_max_/cm^–1^: 3055, 2959, 2932, 2874, 1620, 1161. ^1^H NMR (500 MHz, MeOD-*d*_4_): δ 8.65–8.53 (m, 0.6H), 8.53–8.38 (m, 1H), 8.29 (s, 0.3H), 7.92–7.74 (m, 3H), 7.61–7.34 (m, 3H), 7.24–7.11 (m, 2H), 7.13–6.94 (m, 3H), 4.78 (s, 1.7H), 4.49 (s, 0.6H), 3.57–3.39 (m, 0.6H), 3.22–3.05 (m, 1.5H), 1.77–1.54 (m, 0.6H), 1.46 (h, *J* = 7.2 Hz, 1.4H), 1.42–1.32 (m, 0.6H), 1.03 (h, *J* = 7.4 Hz, 1.3H), 0.99–0.89 (m, 0.8H), 0.68 (t, *J* = 7.3 Hz, 2.1H). NH not observed. Rotamers observed in approximately 3:1 ratio. ^13^C NMR (151 MHz, CDCl_3_): δ 170.8, 170.4, 148.0, 147.7, 140.6, 140.1, 137.4, 136.8, 134.9, 133.7), 129.3, 127.6, 127.1, 125.3, 124.3, 124.1, 121.7, 121.6, 50.1, 48.8, 45.7, 45.0, 30.4, 29.1, 20.1, 19.5, 13.8, 13.5. ACQUITY UPLC BEH C18 1.7 μm: *R*_t_ = 1.51 min; *m*/*z* 424.1 [M + H]^+^. HRMS (EI) calcd for C_23_H_25_O_3_N_3_S, 423.1611; found, 423.1612.

#### *N*-(Pyridin-2-ylmethyl)butan-1-amine

Synthesized according to general procedure B. 2-Picolylamine (95 μL, 0.73 mmol), butyraldehyde (58 μL, 0.64 mmol), NaHCO_3_ (100 mg, 1.20 mmol), NaBH_4_ (36 mg, 0.96 mmol, 1.5 equiv used). The reduction was conducted at 0 °C for 4 h, allowing to warm to room temperature. Crude product taken to next step without further purification.

#### *N*-Butyl-4-(*N*-phenylsulfamoyl)-*N*-(pyridin-2-ylmethyl)benzamide (**45**)

Synthesized according to general procedure C. 4-(*N*-Phenylsulfamoyl)benzoic acid (110 mg, 0.40 mmol), HOBt hydrate (86 mg, 0.56 mmol, 1.4 equiv used), EDC.HCl (122 mg, 0.64 mmol, 1.6 equiv used), *N*-(pyridin-2-ylmethyl)butan-1-amine (crude as above), DCM (5 mL). The reaction mixture was directly concentrated under reduced pressure and purified by flash column chromatography (silica, 24 g, 1:0 petrol/EtOAc to 0:1 petrol/EtOAc). Yield: 27 mg, 0.06 mmol, 17%. Colorless solid. IR (neat) ν_max_/cm^–1^: 3082, 2959, 2932, 2874, 1616, 1163. ^1^H NMR (500 MHz, CDCl_3_): δ 8.64–8.45 (m, 1H), 7.77 (d, *J* = 8.0 Hz, 1H), 7.71–7.60 (m, 2H), 7.50 (d, *J* = 8.0 Hz, 1H), 7.46 (d, *J* = 8.1 Hz, 1H), 7.38 (d, *J* = 7.8 Hz, 1H), 7.23–7.16 (m, 3H), 7.14–7.07 (m, 1H), 7.07–6.98 (m, 2H), 4.84 (s, 1.1H), 4.45 (s, 0.9H), 3.48 (t, *J* = 7.6 Hz, 0.9H), 3.27–3.05 (m, 1.2H), 1.67–1.52 (m, 0.9H), 1.52–1.40 (m, 1.1H), 1.40–1.27 (m, 0.9H), 1.05 (h, *J* = 7.4 Hz, 1.1H), 0.96–0.83 (m, 1.4H), 0.70 (t, *J* = 7.4 Hz, 1.3H). NH not observed. Rotamers observed in approximately 1:1 ratio. ^13^C NMR (151 MHz, CDCl_3_): δ 170.8, 170.6, 156.1, 155.8, 149.6, 147.1, 140.3, 140.3, 140.2, 139.3, 137.3, 136.4, 129.3, 127.5, 127.4, 127.3, 125.6, 123.7, 123.3, 122.9, 122.0, 121.9, 121.3, 53.9, 49.9, 49.3, 45.5, 30.6, 29.1, 20.2, 19.6, 13.8, 13.5. ACQUITY UPLC BEH C18 1.7 μm: *R*_t_ = 1.65 min; *m*/*z* 424.1 [M + H]^+^. HRMS (EI) calcd for C_23_H_25_O_3_N_3_S, 423.1611; found, 423.1608.

#### *N*-(Furan-2-ylmethyl)butan-1-amine

Synthesized according to general procedure B. Furfurylamine (91 μL, 0.73 mmol), butyraldehyde (58 μL, 0.64 mmol), NaHCO_3_ (100 mg, 1.20 mmol), NaBH_4_ (36 mg, 0.96 mmol, 1.5 equiv used). The reduction was conducted at 0 °C for 4 h, allowing to warm to room temperature. Crude product taken to next step without further purification.

#### *N*-Butyl-*N*-(furan-2-ylmethyl)-4-(*N*-phenylsulfamoyl)benzamide (**46**)

Synthesized according to general procedure C. 4-(*N*-Phenylsulfamoyl)benzoic acid (110 mg, 0.40 mmol), HOBt hydrate (86 mg, 0.56 mmol, 1.4 equiv used), EDC.HCl (122 mg, 0.64 mmol, 1.6 equiv used), *N*-(furan-2-ylmethyl)butan-1-amine (crude as above), DCM (5 mL). The reaction mixture was directly concentrated under reduced pressure and purified by flash column chromatography (silica, 24 g, 1:0 petrol/EtOAc to 0:1 petrol/EtOAc) Additionally purified by reverse-phase chromatography (9:1 H_2_O/MeOH to 0:1 H_2_O/MeOH). Yield: 49 mg, 0.12 mmol, 14%. Colorless glass. IR (neat) ν_max_/cm^–1^: 3146, 2961, 2934, 2874, 1616, 1165. ^1^H NMR (500 MHz, MeOD-*d*_4_): δ 7.92–7.69 (m, 2H), 7.55 (d, *J* = 8.0 Hz, 1H), 7.52–7.43 (m, 2H), 7.19 (t, *J* = 7.8 Hz, 2H), 7.12–7.00 (m, 3H), 6.39 (s, 1H), 6.35–6.31 (m, 0.5H), 6.15 (d, *J* = 3.2 Hz, 0.5H), 4.72 (s, 0.9H), 4.32 (s, 1.1H), 3.55–3.37 (m, 1.1H), 3.18–3.04 (m, 1H), 1.52 (p, *J* = 7.7 Hz, 1H), 1.45–1.37 (m, 1H), 1.33 (h, *J* = 7.8 Hz, 1H), 1.02 (h, *J* = 7.4 Hz, 1H), 0.93 (t, *J* = 7.4 Hz, 1.6H), 0.69 (t, *J* = 7.4 Hz, 1.3H). NH not observed. Rotamers observed in approximately 1:1 ratio. ^13^C NMR (151 MHz, CDCl_3_): δ 170.1, 170.1, 150.3, 149.4, 142.9, 142.4, 140.8, 140.1, 140.0, 136.3, 129.4, 129.4, 127.6, 127.4, 127.2, 125.7, 125.7, 122.1, 122.0, 110.6, 110.4, 109.1, 108.9, 48.4, 46.1, 44.9, 40.8, 30.2, 29.1, 20.1, 19.6, 13.8, 13.5. ACQUITY UPLC BEH C18 1.7 μm: *R*_t_ = 1.80 min; *m*/*z* 413.1 [M + H]^+^. HRMS (EI) calcd for C_22_H_24_O_4_N_2_S, 412.1451; found, 412.1451.

#### *N*-((Tetrahydrofuran-2-yl)methyl)butan-1-amine

Synthesized according to general procedure B. Tetrahydrofurfurylamine (0.10 mL, 0.73 mmol), butyraldehyde (58 μL, 0.64 mmol), NaHCO_3_ (100 mg, 1.20 mmol), NaBH_4_ (36 mg, 0.96 mmol, 1.5 equiv used). The reduction was conducted at 0 °C for 4 h, allowing to warm to room temperature. Crude product taken to next step without further purification.

#### *N*-Butyl-4-(*N*-phenylsulfamoyl)-*N*-((tetrahydrofuran-2-yl)methyl)benzamide (**47**)

Synthesized according to general procedure C. 4-(*N*-Phenylsulfamoyl)benzoic acid (110 mg, 0.40 mmol), HOBt hydrate (86 mg, 0.56 mmol, 1.4 equiv used), EDC·HCl (122 mg, 0.64 mmol, 1.6 equiv used), *N*-((tetrahydrofuran-2-yl)methyl)butan-1-amine (crude as above), DCM (5 mL). The reaction mixture was directly concentrated under reduced pressure and purified by flash column chromatography (silica, 24 g, 1:0 petrol/EtOAc to 0:1 petrol/EtOAc) Additionally purified by reverse-phase chromatography (9:1 H_2_O/MeOH to 0:1 H_2_O/MeOH). Yield: 88 mg, 0.21 mmol, 25%. Colorless glass. mp 177–179 °C; IR (neat) ν_max_/cm^–1^ 3140, 2957, 2932, 2872, 1616, 1159. ^1^H NMR (500 MHz, MeOD-*d*_4_): δ 7.81 (t, *J* = 7.3 Hz, 2H), 7.59–7.41 (m, 2H), 7.30–7.14 (m, 2H), 7.11–7.02 (m, 3H), 4.30–4.19 (m, 0.5H), 4.03 (p, *J* = 7.3 Hz, 0.5H), 3.95–3.84 (m, 0.5H), 3.84–3.66 (m, 1.5H), 3.62–3.48 (m, 0.5H), 3.48–3.36 (m, 1H), 3.26–3.10 (m, 1.5H), 2.12–2.02 (m, 0.5H), 2.02–1.89 (m, 1.5H), 1.89–1.78 (m, 0.5H), 1.78–1.58 (m, 3.5H), 1.59–1.50 (m, 0.4H), 1.50–1.32 (m, 1.6H), 1.29–1.14 (m, 0.5H), 1.11–1.02 (m, 0.7H), 0.98 (t, *J* = 7.4 Hz, 1.5H), 0.70 (t, *J* = 7.4 Hz, 1.5H). NH not observed. Rotamers observed in approximately 1:1 ratio. ^13^C NMR (151 MHz, CDCl_3_): δ 170.3, 141.7, 141.4, 139.8, 139.5, 136.2, 129.4, 127.7, 127.4, 127.3, 127.1, 125.8, 125.8, 122.2, 122.1, 77.7, 76.4, 68.0, 67.8, 53.1, 49.7, 48.5, 45.2, 30.5, 29.5, 29.4, 29.2, 25.5, 20.2, 19.6, 13.9, 13.6. ACQUITY UPLC BEH C18 1.7 μm: *R*_t_ = 1.74 min; *m*/*z* 417.1 [M + H]^+^. HRMS (EI) calcd for C_22_H_28_O_4_N_2_S, 416.1764; found, 416.1766.

#### Methyl (*E*)-3-(4-((Butylamino)methyl)phenyl)acrylate

Synthesized according to general procedure B. Methyl (2*E*)-3-(4-formylphenyl)prop-2-enoate (400 mg, 2.1 mmol), *N*-butylamine (185 mg, 2.52 mmol), NaHCO_3_ (353 mg, 4.21 mmol), NaBH_4_ (398 mg, 10.52 mmol, 5 equiv used). The reaction was heated at 80 °C for 4 h prior to NaBH_4_ reduction. After NaBH_4_ addition, MgSO_4_ was directly added, filtered and organic solvent concentrated under reduced pressure. Purified by flash column chromatography (1:0 DCM/MeOH to 9:1 DCM/MeOH over 20 CV’s). Yield: 202 mg, 0.73 mmol, 35%. Colorless oil. Product taken forward as crude to the next step.

#### Methyl 3-(4-((Butylamino)methyl)phenyl)propanoate

In a 2.5 mL microwave vial containing 10% palladium on carbon (5.8 mg, 0.05 mmol), methyl 3-[4-(butylaminomethyl)phenyl]acrylate (100 mg, 0.36 mmol) and MeOH (1 mL) was added and the reaction mixture degassed with N_2_ for 10 min. Triethylsilane (0.09 mL, 0.54 mmol) was added and the mixture stirred at room temperature for 1 h. The mixture was filtered through Celite under reduced pressure using additional quantities of MeOH to wash the filter cake. The filtrate was concentrated under reduced pressure to give methyl 3-[4-(butylaminomethyl)phenyl]propanoate (65 mg, 0.25 mmol, 69% yield) as a colorless oil. ^1^H NMR (500 MHz, CDCl_3_): δ 7.23 (dd, *J* = 8.1, 2.2 Hz, 2H), 7.17–7.12 (m, 2H), 3.74 (s, 2H), 3.66 (s, 3H), 2.93 (t, *J* = 8.0 Hz, 2H), 2.62 (app t, 4H), 1.53–1.43 (m, 2H), 1.39–1.29 (m, 2H), 0.90 (t, *J* = 7.5 Hz, 3H). NH not observed. ACQUITY UPLC BEH C18 1.7 μm: *R*_t_ = 1.29 min; *m*/*z* 250.2 [M + H]^+^.

#### Methyl 3-(4-((*N*-Butyl-4-(*N*-phenylsulfamoyl)benzamido)methyl)phenyl)propanoate

Synthesized according to general procedure C. 4-(Phenylsulfamoyl)benzoic acid (72 mg, 0.26 mmol), HOBt hydrate (44 mg, 0.29 mmol), EDC·HCl (60 mg, 0.31 mmol), methyl 3-[4-(butylaminomethyl)phenyl]propanoate (65 mg, 0.26 mmol), DCM (4 mL). Saturated NaHCO_3_ (4 mL) was used. Purified by flash column chromatography (silica, 12 g, 1:0 petrol/EtOAc to 4:1 petrol/EtOAc, then rapid gradient to 0:1 petrol/EtOAc over 30 CV’s). Yield: 114 mg, 0.21 mmol, 82%. Colorless oil. ^1^H NMR (500 MHz, CDCl_3_): δ 7.80 (d, *J* = 7.9 Hz, 1.1H), 7.73 (d, *J* = 8.0 Hz, 0.9H), 7.56 (s, 1.1H), 7.44 (dd, *J* = 9.7 Hz, 2.3H), 7.31–7.27 (m, 0.7H), 7.25–7.15 (m, 4.8H), 7.15–7.04 (m, 3.3H), 7.01 (d, *J* = 7.6 Hz, 1H), 4.73 (s, 1.1H), 4.36 (s, 0.9H), 3.70 (s, 3H), 3.48 (t, *J* = 7.7 Hz, 1H), 3.03 (t, *J* = 7.6 Hz, 1H), 3.00–2.91 (m, 2H), 2.65 (t, *J* = 7.9 Hz, 2H), 1.63 (p, *J* = 7.8 Hz, 1H), 1.44 (p, *J* = 7.5 Hz., 1H), 1.36 (h, *J* = 7.0 Hz, 0.6H), 1.06 (h, *J* = 7.5 Hz, 1.2H), 0.95 (t, *J* = 7.6 Hz, 1.5H), 0.73 (t, *J* = 7.4 Hz, 1.6H). Rotamers observed in approximately 1:1 ratio. ACQUITY UPLC BEH C18 1.7 μm: *R*_t_ = 1.84 min; *m*/*z* 509.3 [M + H]^+^.

#### 3-(4-((*N*-Butyl-4-(*N*-phenylsulfamoyl)benzamido)methyl)phenyl)propanoic acid (**48**)

To a solution of methyl 3-[4-[[butyl-[4-phenylsulfamoyl)benzoyl]amino]methyl]phenyl]propanoate (85 mg, 0.16 mmol) in 1:1:1 MeOH/THF/H_2_O (0.9 mL each, respectively) was added lithium hydroxide monohydrate (40 mg, 0.95 mmol). The mixture was stirred at room temperature for 2 days. The reaction mixture was concentrated under reduced pressure and 1 M HCl solution (3 mL) added. Following stirring for 5 min, the supernatant was removed by pipet and the white solid washed with water (5 mL). The solid was dried thoroughly to afford 3-[4-[[butyl-[4-(phenylsulfamoyl)benzoyl]amino]methyl]phenyl]propanoic acid (82 mg, 0.158 mmol, 99% yield) as a colorless solid. ^1^H NMR (500 MHz, CDCl_3_): δ 7.76 (d, *J* = 7.9 Hz, 1H), 7.64 (d, *J* = 7.9 Hz, 1H), 7.43 (d, *J* = 7.9 Hz, 1H), 7.32 (d, *J* = 7.9 Hz, 1H), 7.25–7.21 (m, 1H), 7.19 (d, *J* = 7.4 Hz, 4H), 7.15–7.08 (m, 2H), 7.04 (d, *J* = 7.8 Hz, 2H), 6.92 (d, *J* = 7.6 Hz, 1H), 4.70 (s, 1H), 4.32 (s, 1H), 3.49 (t, 1.1H), 3.00 (t, *J* = 7.6 Hz, 0.9H), 2.97–2.88 (m, 2H), 2.66 (t, *J* = 7.5 Hz, 2H), 1.62 (p, *J* = 7.6 Hz, 1.1H), 1.46–1.38 (m, 0.8H), 1.38–1.30 (m, 0.7H), 1.08–0.98 (m, 1.1H), 0.93 (t, *J* = 7.4 Hz, 1.5H), 0.70 (t, *J* = 7.4 Hz, 1.5H). COOH not observed. Rotamers observed in approximately 1:1 ratio. ACQUITY UPLC BEH C18 1.7 μm: *R*_t_ = 1.73 min; *m*/*z* 495.2 [M + H]^+^.

#### *tert*-Butyl 4-(4-((Butylamino)methyl)phenyl)piperazine-1-carboxylate

Synthesized according to general procedure B. *N*-Butylamine (40 μL, 0.36 mmol, 1 eq used), *tert*-butyl 4-(4-formylphenyl)piperazine-1-carboxylate (220 mg, 0.76 mmol, 2.1 equiv used). NaBH_4_ (40 mg, 1.06 mmol, 2.9 equiv used). The reaction mixture was left for 4 h after NaBH_4_ addition and quenched with 2 M NaOH (5 mL). Product was taken forward as crude to the next step.

#### *tert*-Butyl 4-(4-((*N*-Butyl-4-(*N*-phenylsulfamoyl)benzamido)methyl)phenyl)piperazine-1-carboxylate

Synthesized according to general procedure D. 4-(Phenylsulfamoyl)benzoyl chloride (107 mg, 0.36 mmol, Key Intermediate A), Et_3_N (76 μL, 0.55 mmol). Washed with saturated NH_4_Cl (3 mL) instead of 1 M HCl. Purified by flash column chromatography (silica, 4 g, 4:1 petrol/EtOAc to 1:1 petrol/EtOAc) followed by additional purification by reverse-phase chromatography (9:1H_2_O/MeOH to 0:1H_2_O/MeOH). Yield: 94 mg, 0.15 mmol, 41%. Colorless solid. ^1^H NMR (500 MHz, CDCl_3_): δ 7.78 (d, *J* = 7.9 Hz, 1H), 7.73 (d, *J* = 7.9 Hz, 1H), 7.44 (d, *J* = 8.0 Hz, 2H), 7.29–7.22 (m, 3H), 7.16 (d, *J* = 8.1 Hz, 1H), 7.06 (d, *J* = 7.1 Hz, 2H), 6.97 (d, *J* = 8.0 Hz, 1H), 6.93–6.84 (m, 2H), 6.47 (s, 1H), 4.66 (s, 1H), 4.29 (s, 1H), 3.59 (s, 4H), 3.45 (s, 1H), 3.14 (s, 4H), 2.98 (t, *J* = 8.9 Hz, 1H), 1.49 (s, 9H), 1.45–1.25 (m, 2H), 1.05 (p, *J* = 7.5 Hz, 1H), 0.93 (t, *J* = 7.9 Hz, 1.5H), 0.90–0.80 (m, 1H), 0.73 (t, *J* = 7.5 Hz, 1.5H). Rotamers observed in approximately 1:1 ratio. ACQUITY UPLC BEH C18 1.7 μm: *R*_t_ = 1.87 min; *m*/*z* 607.4 [M + H]^+^.

#### *N*-Butyl-4-(*N*-phenylsulfamoyl)-*N*-(4-(piperazin-1-yl)benzyl)benzamide (**49**)

*tert*-Butyl 4-(4-((*N*-butyl-4-(*N*-phenylsulfamoyl)benzamido)methyl)phenyl)piperazine-1-carboxylate (100 mg, 0.17 mmol) was suspended in 4 N HCl in 1,4-dioxane (2 mL, 8 mmol). The reaction mixture was stirred at room temperature for 30 min. The reaction mixture was concentrated under reduced pressure. The mixture was resuspended in MeOH (1 mL) and TBME (4 mL), followed by concentration under reduced pressure. The precipitate was dried thoroughly to afford *N*-butyl-4-(*N*-phenylsulfamoyl)-*N*-(4-(piperazin-1-yl)benzyl)benzamide dihydrochloride (90 mg, 0.14 mmol, 93% yield) as a yellow solid. ^1^H NMR (500 MHz, DMSO-*d*_6_): δ 10.35 (s, 1H), 9.17–8.94 (m, 2H), 7.83–7.73 (m, 2H), 7.55–7.49 (m, 2H), 7.26–7.17 (m, 3H), 7.10–6.85 (m, 6H), 4.57 (s, 1.2H), 4.24 (s, 0.7H), 3.37–3.26 (m, 5H), 3.21 (s, 4H), 2.92 (t, *J* = 7.6 Hz, 1H), 1.56–1.44 (m, 1H), 1.39–1.20 (m, 2H), 0.96–0.90 (m, 1H), 0.87 (t, *J* = 7.5 Hz, 1H), 0.57 (t, *J* = 7.4 Hz, 1.6H). Rotamers observed in approximately 3:2 ratio. Residual TBME observed but product purity >95% as determined by ^1^H NMR. ACQUITY UPLC BEH C18 1.7 μm: *R*_t_ = 1.46 min; *m*/*z* 507.3 [M + H, free base]^+^.

#### *N*-(4-(4-Benzylpiperazin-1-yl)benzyl)butan-1-amine

Synthesized according to general procedure B. *N*-Butylamine (40 μL, 0.36 mmol, 1 equiv used), 4-(4-benzylpiperazin-1-yl)benzaldehyde (212 mg, 0.76 mmol, 2.1 equiv used). NaBH_4_ (40 mg, 1.06 mmol, 2.9 equiv used). The reaction mixture was left for 4 h after NaBH_4_ addition and quenched with 2 M NaOH (5 mL). Product was taken forward as crude to the next step.

#### *N*-[[4-(4-Benzylpiperazin-1-yl)phenyl]methyl]-*N*-butyl-4-(phenylsulfamoyl)benzamide (**50**)

Synthesized according to general procedure D. 4-(Phenylsulfamoyl)benzoyl chloride (107 mg, 0.36 mmol, Key Intermediate A), Et_3_N (76 μL, 0.55 mmol). Washed with saturated NH_4_Cl (3 mL) instead of 1 M HCl. Purified by flash column chromatography (silica, 4 g, 4:1 petrol/EtOAc to 1:1 petrol/EtOAc) followed by additional purification by reverse-phase chromatography (9:1 H_2_O/MeOH to 0:1 H_2_O/MeOH). Yield: 103 mg, 0.16 mmol, 45%. Colorless glass. ^1^H NMR (500 MHz, CDCl_3_): δ 7.80–7.68 (m, 2H), 7.43 (d, *J* = 8.0 Hz, 2H), 7.38–7.30 (m, 5H), 7.30–7.17 (m, 3H), 7.17–7.10 (m, 1H), 7.08–6.99 (m, 2H), 6.97–6.80 (m, 3H), 6.49 (br s, 1H), 4.64 (s, 1H), 4.27 (s, 1H), 3.61–3.54 (m, 2H), 3.47–3.40 (m, 1H), 3.23–3.15 (m, 4H), 3.00–2.93 (m, 1H), 2.64–2.58 (m, 4H), 1.45–1.38 (m, 1H), 1.36–1.30 (m, 1H), 1.30–1.23 (m, 1H), 1.08–0.98 (m, 1H), 0.93 (t, *J* = 7.8 Hz, 1.4H), 0.71 (t, *J* = 7.5 Hz, 1.4H). Rotamers observed in approximately 1:1 ratio. ACQUITY UPLC BEH C18 1.7 μm: *R*_t_ = 1.48 min; *m*/*z* 597.3 [M + H]^+^.

#### *N*-((1*H*-Indol-5-yl)methyl)-butan-1-amine

Synthesized according to general procedure B. *N*-Butylamine (0.45 mL, 0.45 mmol), 5-formylindole (589 mg, 4.06 mmol), NaHCO_3_ (909 mg, 10.82 mmol), MeOH (8 mL). NaBH_4_ (174 mg, 4.60 mmol). Yield: 841 mg, 4.20 mmol, 96.7%. Orange oil. ACQUITY UPLC BEH C18 1.7 μm: *R*_t_ = 0.55 min; *m*/*z* 203.1 [M + H]^+^.

#### *N*-Butyl-*N*-(1*H*-indol-5-ylmethyl)-4-(phenylsulfamoyl)benzamide (**51**)

Synthesized according to general procedure C. 4-(Phenylsulfamoyl)benzoic acid (750 mg, 2.70 mmol), HOBt hydrate (456 mg, 2.98 mmol), EDC.HCl (622 mg, 3.25 mmol), *N*-((1*H*-indol-5-yl)methyl)-1-cyclopropylmethanamine (841 mg, 4.20 mmol), DCM (8 mL). Purified by flash column chromatography (silica, 24 g, 1:0 petrol/EtOAc to 1:4 petrol/EtOAc over 25 CV’s). Yield: 838 mg,1.73 mmol, 64%. Colorless solid. ^1^H NMR (500 MHz, CDCl_3_): δ 8.25–8.18 (m, 0.7H), 7.76 (d, *J* = 8.0 Hz, 1H), 7.71 (d, *J* = 8.1 Hz, 1H), 7.59 (s, 0.5H), 7.49 (d, *J* = 8.2 Hz, 1H), 7.45 (d, *J* = 8.2 Hz, 1H), 7.40–7.33 (m, 1.5H), 7.29–7.17 (m, 4H), 7.15–7.08 (m, 1H), 7.07–7.00 (m, 2H), 6.86 (d, *J* = 8.4 Hz, 0.5H), 6.56–6.49 (m, 2H), 4.84 (s, 1H), 4.47 (s, 1H), 3.49 (t, *J* = 7.6 Hz, 1H), 2.99 (t, *J* = 7.7 Hz, 1H), 1.63 (p, *J* = 7.5 Hz, 1H), 1.44 (p, *J* = 7.7 Hz, 1H), 1.34 (h, *J* = 7.4 Hz, 1H), 1.03 (h, *J* = 7.3 Hz, 1H), 0.93 (t, *J* = 7.3 Hz, 1.7H), 0.71 (t, *J* = 7.3 Hz, 1.6H). Rotamers observed in approximately 1:1 ratio. ACQUITY UPLC BEH C18 1.7 μm: *R*_t_ = 1.79 min; *m*/*z* 462.2 [M + H]^+^.

#### *N*-Benzyl-*N*-ethyl-4-(*N*-phenylsulfamoyl)benzamide (**52**)

Synthesized according to general procedure C. 4-(*N*-Phenylsulfamoyl)benzoic acid (100 mg, 0.36 mmol), HOBt hydrate (64 mg, 0.41 mmol), EDC.HCl (88 mg, 0.46 mmol), *N*-benzylethanamine (79 μL, 0.54 mmol). Purified by purified by flash column chromatography (silica, 12 g, 1:0 petrol/EtOAc to 0:1 petrol/EtOAc over 25 CVs), followed by additional purified by flash column chromatography (silica, 12 g, 1:0 DCM/MeOH to 99:1 DCM/MeOH over 30 CV’s) and reverse-phase chromatography (9:1 H_2_O/MeCN to 1:4 H_2_O/MeCN over 25 min). Yield: 99 mg, 0.25 mmol, 69%. Colorless glass. ^1^H NMR (500 MHz, MeOD-*d*_4_): δ 7.80 (dd, *J* = 44.9, 8.2 Hz, 2H), 7.51 (dd, *J* = 31.7, 8.2 Hz, 2H), 7.39–7.24 (m, 5H), 7.24–7.13 (m, 2H), 7.13–6.97 (m, 3H), 4.76 (s, 1.2H), 4.41 (s, 1H), 3.51 (q, *J* = 7.1 Hz, 0.9H), 3.14 (q, *J* = 7.1 Hz, 1.2H), 1.19 (t, *J* = 7.1 Hz, 1.3H), 1.02 (t, *J* = 7.1 Hz, 1.7H). Rotamers observed in approximately 1:1 ratio. ACQUITY UPLC BEH C18 1.7 μm: *R*_t_ = 1.76 min; *m*/*z* 395.1 [M + H]^+^.

2-(Benzylamino)ethan-1-ol. Synthesized according to general procedure B. 2-Aminoethanol (37 μL, 0.60 mmol, 1 equiv used), benzaldehyde (77 μL, 0.60 mmol). Reaction conducted in DCM (1 mL). NaBH_4_ (40 mg, 1.06 mmol, 1.8 equiv used). The reaction mixture was left for 2 h and quenched with 2 M NaOH (5 mL). Product was taken forward as crude to the next step.

#### *N*-Benzyl-*N*-(2-hydroxyethyl)-4-(*N*-phenylsulfamoyl)benzamide (**53**)

Synthesized according to general procedure D. 4-(Phenylsulfamoyl)benzoyl chloride (107 mg, 0.36 mmol, Key Intermediate A), Et_3_N (76 μL, 0.55 mmol). Purified by flash column chromatography (silica, 4 g, 3:2 petrol/EtOAc to 0:1 petrol/EtOAc). Yield: 160 mg, 0.35 mmol, 97%. Off-white foam. ^1^H NMR (500 MHz, CDCl_3_): δ 7.80–7.70 (m, 2H), 7.56–7.47 (m, 2H), 7.40–7.19 (m, 7H), 7.16–7.09 (m, 2H), 7.08–7.01 (m, 1H), 6.79 (br s, 0.2H), 6.72 (br s, 0.6H), 4.83 (s, 0.6H), 4.49 (s, 1.4H), 3.87–3.81 (m, 1.4H), 3.73–3.67 (m, 0.8H), 3.62–3.58 (m, 0.5H), 3.29–3.25 (m, 0.6H). OH not observed. Rotamers observed in approximately 7:3 ratio. ^13^C NMR (125 MHz, CDCl_3_): δ 172.3, 140.6, 140.2, 136.0, 135.8, 129.6, 129.3, 128.2, 127.7, 127.6, 126.8, 126.1, 122.2, 61.6, 54.0, 48.9. ACQUITY UPLC BEH C18 1.7 μm: *R*_t_ = 1.61 min; *m*/*z* 411.3 [M + H]^+^.

#### *N*-Benzyl-*N*-(2-cyanoethyl)-4-(*N*-phenylsulfamoyl)benzamide (**54**)

Synthesized according to general procedure C. 4-(*N*-Phenylsulfamoyl)benzoic acid (100 mg, 0.36 mmol), HOBt hydrate (64 mg, 0.41 mmol), EDC·HCl (83 mg, 0.43 mmol), 3-(benzylamino)propionitrile (0.85 μL, 0.54 mmol). Purified by automated column chromatography (silica, 12 g, 1:0 petrol/EtOAc to 0:1 petrol/EtOAc over 25 CV’s), followed by additional purification by automated column chromatography (silica, 12 g, 1:0 DCM/MeOH to 99:1 DCM/MeOH over 30 CVs) and reverse-phase chromatography (9:1 H_2_O/MeCN to 4:1 H_2_O/MeCN over 25 min. Yield: 53 mg, 0.13 mmol, 36%. Colorless glass. ^1^H NMR (500 MHz, CDCl_3_): δ 7.77 (d, *J* = 8.0 Hz, 2H), 7.56–7.48 (m, 2H), 7.42–7.29 (m, 3H), 7.23 (t, *J* = 7.8 Hz, 2H), 7.18–6.99 (m, 5H), 6.56 (s, 1H), 4.83 (s, 0.2H), 4.56 (s, 1.8H), 3.69 (t, *J* = 6.5 Hz, 1.8H), 3.45–3.37 (m, 0.3H), 2.78 (t, *J* = 6.4 Hz, 1.8H), 2.42–2.33 (m, 0.3H). Rotamers observed in approximately 9:1 ratio. ACQUITY UPLC BEH C18 1.7 μm: *R*_t_ = 1.69 min; *m*/*z* 420.1 [M + H]^+^.

#### 3-(4-Pyridylmethylamino)propanenitrile

4-Picolinylamine (0.28 mL, 2.77 mmol) was added to a solution of 3-bromopropionitrile (0.25 mL, 3.05 mmol) and K_2_CO_3_ (1.17 g, 8.32 mmol) in MeCN (3 mL). The reaction mixture was heated to 80 °C and stirred overnight. The crude mixture was filtered and the filtrate concentrated under reduced pressure with silica. The crude mixture was purified by flash column chromatography (1:0 DCM/10% MeOH in DCM +1% Et_3_N to 0:1 DCM/10% MeOH in DCM +1% Et_3_N over 25 CV’s). Fractions containing product were combined and concentrated under reduced pressure to afford 3-(4-pyridylmethylamino)propanenitrile (193 mg, 1.13 mmol, 41% yield) as an orange oil. ^1^H NMR (500 MHz, CDCl_3_): δ 8.55–8.53 (m, 2H), 7.28–7.25 (m, 2H), 3.85 (s, 2H), 2.92 (t, *J* = 6.5 Hz, 2H), 2.52 (t, *J* = 6.5 Hz, 2H). NH not observed. ACQUITY UPLC BEH C18 1.7 μm: *R*_t_ = 0.19 min; *m*/*z* 162.1 [M + H]^+^.

#### *N*-(2-Cyanoethyl)-4-(phenylsulfamoyl)-*N*-(4-pyridylmethyl)benzamide (**55**)

Synthesized according to general procedure C. 4-(Phenylsulfamoyl)benzoic acid (200 mg, 0.72 mmol), HOBt hydrate (122 mg, 0.79 mmol), EDC.HCl (166 mg, 0.87 mmol), 3-(4-pyridylmethylamino)propanenitrile (184 mg, 1.08 mmol). Purified by automated column chromatography (silica, 12 g, 1:0 DCM/10% MeOH in DCM +1% Et_3_N to 1:1 DCM/10% MeOH in DCM +1% Et_3_N over 25 CV’s), followed by additional purification by reverse-phase chromatography (1:9 MeOH/H_2_O to 1:0 MeOH/H_2_O for 25 min). Yield: 54 mg, 0.12 mmol, 16.8%. Off-white solid. ^1^H NMR (500 MHz, CDCl_3_): δ 8.65–8.59 (m, 2H), 7.76 (d, *J* = 7.7 Hz, 2H), 7.46 (d, *J* = 7.8 Hz, 2H), 7.24 (t, *J* = 7.8 Hz, 2H), 7.14 (t, *J* = 7.4 Hz, 1H), 7.09–6.99 (m, 4H), 4.81 (s, 0.5H), 4.61 (s, 1.7H), 3.80–3.65 (m, 1.8H), 3.60–3.41 (m, 0.4H), 2.96–2.78 (m, 1.7H), 2.60–2.33 (m, 0.3H). Rotamers observed in approximately 4:1 ratio. NH not observed. ACQUITY UPLC BEH C18 1.7 μm: *R*_t_ = 0.42 min; *m*/*z* 421.2 [M + H]^+^.

#### *N*-Benzyl-3-(1*H*-imidazol-1-yl)propan-1-amine

Synthesized according to general procedure B. 3-Imidazol-1-ylpropan-1-amine (75 mg, 0.60 mmol, 1 equiv used), benzaldehyde (77 μL, 0.60 mmol). Reaction conducted in DCM (1 mL). NaBH_4_ (40 mg, 1.06 mmol, 1.8 equiv used). The reaction mixture was left for 2 h and quenched with 2 M NaOH (5 mL). Product was taken forward as crude to the next step.

#### *N*-Benzyl-*N*-(3-imidazol-1-ylpropyl)-4-(phenylsulfamoyl)benzamide (**56**)

Synthesized according to general procedure D. 4-(Phenylsulfamoyl)benzoyl chloride (107 mg, 0.36 mmol, Key Intermediate A), Et_3_N (76 μL, 0.55 mmol). Purified by flash column chromatography (silica, 4 g, 3:2 petrol/EtOAc to 0:1 petrol/EtOAc, followed by gradient of 1:0 EtOAc/MeOH to 4:1 EtOAc/MeOH). Yield: 70 mg, 0.14 mmol, 39%. Colorless gum. ^1^H NMR (500 MHz, CDCl_3_): δ 7.84 (d, *J* = 3.2 Hz, 0.6H), 7.75 (d, *J* = 8.0 Hz, 2.2H), 7.44 (d, *J* = 8.0 Hz, 1H), 7.39–7.26 (m, 5.5H), 7.26–7.14 (m, 3.2H), 7.12–6.91 (m, 5.2H), 6.61 (s, 0.4H), 4.73 (s, 0.8H), 4.35 (s, 1.1H), 4.08–3.99 (m, 1.1H), 3.76–3.67 (m, 0.8H), 3.53–3.44 (m, 1.1H), 3.06–2.96 (m, 0.8H), 2.16–2.05 (m, 1.3H), 1.95–1.81 (m, 0.8H). Rotamers observed in approximately 11:9 ratio. ACQUITY UPLC BEH C18 1.7 μm: *R*_t_ = 1.42 min; *m*/*z* 475.3 [M + H]^+^.

#### *N*-Benzyl-1-cyclopropylmethanamine (**88a**)

Synthesized according to general procedure B. Cyclopropylmethanamine (52 μL, 0.60 mmol, 1 equiv used), benzaldehyde (77 μL, 0.60 mmol). Reaction conducted in DCM (1 mL). NaBH_4_ (40 mg, 1.06 mmol, 1.8 equiv used). The reaction mixture was left for 2 h and quenched with 2 M NaOH (5 mL). Product was taken forward as crude to the next step.

#### *N*-Benzyl-*N*-(cyclopropylmethyl)-4-(phenylsulfamoyl)benzamide (**57**)

Synthesized according to general procedure D. 4-(Phenylsulfamoyl)benzoyl chloride (107 mg, 0.36 mmol, Key Intermediate A), Et_3_N (76 μL, 0.55 mmol). Purified by flash column chromatography (silica, 4 g, 3:2 petrol/EtOAc to 0:1 petrol/EtOAc). Yield: 105 mg, 0.22 mmol, 62%. Off-white foam. ^1^H NMR (400 MHz, CDCl_3_): δ 7.83–7.67 (m, 3H), 7.47 (d, *J* = 7.9 Hz, 2H), 7.39–7.19 (m, 7H), 7.17–6.98 (m, 2H), 6.79 (br s, 1H), 4.90 (s, 1.1H), 4.51 (s, 1H), 3.40 (d, *J* = 6.9 Hz, 1H), 2.92 (d, *J* = 6.5 Hz, 1.2H), 1.13–1.01 (m, 0.4H), 0.86–0.74 (m, 0.7H), 0.57–0.40 (m, 2H), 0.28–0.14 (m, 0.9H), −0.03 – −0.19 (m, 1.2H). Rotamers observed in approximately 1:1 ratio. ACQUITY UPLC CORTECS C18 1.7 μm: *R*_t_ = 1.73 min; *m*/*z* 421.2 [M + H]^+^.

#### *N*-Benzyl-*N*-cyclopropyl-4-(phenylsulfamoyl)benzamide (**58**)

Synthesized according to general procedure C. 4-(Phenylsulfamoyl)benzoic acid (100 mg, 0.35 mmol), HOBt hydrate (65 mg, 0.42 mmol), EDC·HCl (81 mg, 0.42 mmol), *N*-cyclopropylbenzylamine (52 mg, 0.35 mmol). Purified by automated column chromatography (silica, 12 g, 1:0 petrol/EtOAc to 1:1 petrol/EtOAc over 20 CV’s). Yield: 80 mg, 0.19 mmol, 53%. Colorless solid. ^1^H NMR (500 MHz, CDCl_3_): δ 7.69 (dd, *J* = 8.3, 2.4 Hz, 2H), 7.46 (d, *J* = 7.9 Hz, 2H), 7.27 (d, *J* = 27.0 Hz, 5H), 7.21–7.14 (m, 2H), 7.10–7.04 (m, 1H), 6.99 (d, *J* = 7.8 Hz, 2H), 6.79 (s, 1H), 4.69 (s, 2H), 2.45 (s, 1H), 0.49–0.26 (m, 4H). ACQUITY UPLC CORTECS C18 1.7 μm: *R*_t_ = 1.70 min; *m*/*z* 407.2 [M + H]^+^.

#### *N*-Benzyl-*N*-isobutyl-4-(phenylsulfamoyl)benzamide (**59**)

Synthesized according to general procedure C. 4-(Phenylsulfamoyl)benzoic acid (100 mg, 0.36 mmol, HOBt hydrate (61 mg, 0.40 mmol), EDC·HCl (83 mg, 0.43 mmol), *N*-benzyl-2-methylpropan-1-amine (0.08 mL, 0.43 mmol), DCM (5 mL). Purified by flash column chromatography (silica, 12 g, 1:0 petrol/EtOAc to 1:4 petrol/EtOAc over 25 CV’s). Yield: 49 mg, 0.11 mmol, 31%. Colorless solid. ^1^H NMR (500 MHz, CDCl_3_): δ 7.76 (d, *J* = 8.0 Hz, 1H), 7.71 (d, *J* = 8.1 Hz, 1H), 7.44–7.40 (m, 2H), 7.39–7.27 (m, 3.8H), 7.24–7.18 (m, 1.8H), 7.16–7.09 (m, 1.1H), 7.07–7.00 (m, 3.3H), 6.69 (s, 1H), 4.76 (s, 1H), 4.39 (s, 1H), 3.32 (d, *J* = 7.6 Hz, 1H), 2.87 (d, *J* = 7.5 Hz, 1H), 2.16–2.07 (m, 0.5H), 1.95–1.80 (m, 0.5H), 0.96 (d, *J* = 6.7 Hz, 3H), 0.69 (d, *J* = 6.6 Hz, 3H). Rotamers observed in approximately 1:1 ratio. ACQUITY UPLC BEH C18 1.7 μm: *R*_t_ = 1.84 min; *m*/*z* 423.3 [M + H]^+^.

#### *N*-[[1-(Imidazol-1-ylmethyl)cyclopropyl]methyl]-1-phenyl-methanamine

Synthesized according to general procedure B but reversed reagents for reductive amination and imine formation was carried out at 65 °C overnight. 1-[1-(1*H*-Imidazol-1-ylmethyl)cyclopropyl]methanamine (0.15 mL, 0.99 mmol, 1 equiv used), benzaldehyde (0.12 mL, 1.19 mmol, 1.2 equiv used), NaHCO_3_ (250 mg, 2.98 mmol), NaBH_4_ (45 mg, 1.19 mmol). Yield: 257 mg, 0.80 mmol, 81% (75% purity). Colorless oil. ^1^H NMR (500 MHz, CDCl_3_): δ 7.37 (t, *J* = 1.2 Hz, 1H), 7.29–7.13 (m, 5H), 6.90 (t, *J* = 1.1 Hz, 1H), 6.82 (t, *J* = 1.3 Hz, 1H), 3.83 (s, 2H), 3.62 (s, 2H), 2.20 (s, 2H), 0.47–0.43 (m, 2H), 0.37–0.33 (m, 2H). NH not observed. ACQUITY UPLC BEH C18 1.7 μm: *R*_t_ = 0.24 min; *m*/*z* 242.1 [M + H]^+^.

#### *N*-Benzyl-*N*-[[1-(imidazol-1-ylmethyl)cyclopropyl]methyl]-4-(phenylsulfamoyl)benzamide (**60**)

Synthesized according to general procedure D. 4-(Phenylsulfamoyl)benzoyl chloride (590 mg, 2.00 mmol, Key Intermediate A), *N*-[[1-(imidazol-1-ylmethyl)cyclopropyl]methyl]-1-phenyl-methanamine (257 mg, 0.80 mmol), Et_3_N (0.28 mL, 2.00 mmol). A mixture of the desired product and imidazole dimer formed that were not separable. To cleave the imidazole *N* side product, the crude mixture was dissolved in THF (15 mL) and 2 M NaOH (10 mL) was added. The reaction mixture was stirred at room temperature for 1 h. The desired product was extracted with EtOAc (3 × 50 mL). The combined organic extracts were dried over MgSO_4_, filtered and concentrated under reduced pressure with silica. The crude mixture was then purified by flash column chromatography (silica, 12 g, 1:0 DCM/20% MeOH in DCM to 1:1 DCM/20% MeOH in DCM over 25 CV’s), followed by additional purification of the relevant concentrated fractions by reverse-phase chromatography (1:9 MeOH/H_2_O to 1:0 MeOH/H_2_O over 20 min). Fractions containing product were combined and concentrated under reduced pressure to afford *N*-benzyl-*N*-[[1-(imidazol-1-ylmethyl)cyclopropyl]methyl]-4-(phenylsulfamoyl)benzamide (44 mg, 0.08 mmol, 10% yield) as a light yellow solid. ^1^H NMR (500 MHz, DMSO-*d*_6_): δ 10.35 (br s, 1H), 7.82–6.61 (m, 17H), 4.75 (s, 0.7H), 4.42 (s, 1.4H), 3.98 (s, 1.5H), 3.76 (s, 0.7H), 3.03 (s, 0.7H), 0.71–0.61 (m, 1.4H), 0.56–0.39 (m, 2.2H), 0.31–0.17 (m, 0.7H). CH_2_ for major rotamer not observed as overlapping with HDO peak. Rotamers observed in approximately 2:1 ratio. ACQUITY UPLC BEH C18 1.7 μm: *R*_t_ = 1.42 min; *m*/*z* 501.2 [M + H]^+^.

#### *N*-(Cyclopropylmethyl)-1-(1*H*-indol-5-yl)methanamine

Synthesized according to general procedure B but reversed reagents for reductive amination. (1*H*-Indol-5-yl)methanamine (300 mg, 2.05 mmol, 1 equiv used), cyclopropanecarbaldehyde (0.19 mL, 2.48 mmol, 1.2 equiv used). NaBH_4_ (120 mg, 3.17 mmol, 1.5 equiv used). Yield: 450 mg, 2.02 mmol, 98%. Product was taken forward as crude to the next step.

#### *N*-(Cyclopropylmethyl)-*N*-(1*H*-indol-5-ylmethyl)-4-(phenylsulfamoyl)benzamide (**61**)

Synthesized according to general procedure D. 4-(Phenylsulfamoyl)benzoyl chloride (80 mg, 0.27 mmol, Key Intermediate A), *N*-(cyclopropylmethyl)-1-(1*H*-indol-5-yl)methanamine (65 mg, 0.32 mmol, 1.2 equiv used), Et_3_N (56 μL, 0.39 mmol). Washed with 0.6 M citric acid (3 mL) instead of 1 M HCl. Purified by flash column chromatography (silica, 4 g, 1:0 petrol/EtOAc to 1:1 petrol/EtOAc) followed by additional purification by reverse-phase chromatography (4:1 H_2_O/MeOH to 1:9 H_2_O/MeOH). Yield: 5 mg, 0.01 mmol, 5%. Colorless solid. ^1^H NMR (500 MHz, CDCl_3_): δ 8.26–8.18 (m, 1H), 7.83–7.67 (m, 2H), 7.60–7.44 (m, 3H), 7.41–7.31 (m, 2H), 7.25–7.08 (m, 3H), 7.08–6.99 (m, 2H), 6.88 (d, *J* = 8.2 Hz, 1H), 6.65 (s, 1H), 6.53 (s, 1H), 5.00 (s, 1H), 4.60 (s, 1H), 3.42 (d, *J* = 7.0 Hz, 1H), 2.90 (d, *J* = 6.7 Hz, 1H), 1.16–1.03 (m, 1H), 0.90–0.77 (m, 1H), 0.55–0.42 (m, 2H), 0.25–0.18 (m, 1H), −0.03 – −0.13 (m, 1H). Rotamers observed in approximately 1:1 ratio. ACQUITY UPLC BEH C18 1.7 μm: *R*_t_ = 1.73 min; *m*/*z* 460.2 [M + H]^+^.

#### 1-(1,3-Benzoxazol-6-yl)-*N*-(cyclopropylmethyl)methanamine

Cyclopropylmethylamine (49.1 μL, 0.57 mmol) was added to a solution of 6-(bromomethyl)benzo[*d*]oxazole (100 mg, 0.47 mmol) and Et_3_N (0.13 mL, 0.94 mmol) in THF (2 mL). The reaction mixture was stirred at 50 °C overnight. The reaction mixture was diluted with DCM (10 mL) and washed with water (2 × 10 mL) and brine (10 mL). The organic layer was separated using a phase separator and the filtrate concentrated under reduced pressure to afford 1-(1,3-benzoxazol-6-yl)-*N*-(cyclopropylmethyl)methanamine (111 mg, 0.27 mmol, 58% yield) as an orange solid. ACQUITY UPLC BEH C18 1.7 μm: *R*_t_ = 0.34 min; *m*/*z* 203.1 [M + H]^+^. The product was taken forward as crude to the next step.

#### *N*-(1,3-Benzoxazol-6-ylmethyl)-*N*-(cyclopropylmethyl)-4-(phenylsulfamoyl)benzamide (**62**)

Synthesized according to general procedure C. 4-(Phenylsulfamoyl)benzoic acid (152 mg, 0.55 mmol), HOBt hydrate (93 mg, 0.60 mmol), EDC.HCl (126 mg, 0.66 mmol), 1-(1,3-benzoxazol-6-yl)-*N*-(cyclopropylmethyl)methanamine (111 mg, 0.55 mmol), DCM (5 mL). Purified by flash column chromatography (silica, 12 g, 1:0 petrol/EtOAc to 0:1 petrol/EtOAc over 25 CV’s). Yield: 13 mg, 1.03 mmol, 5%. Colorless solid. ^1^H NMR (400 MHz, CDCl_3_): δ 8.11 (s, 1H), 7.85–7.69 (m, 2.7H), 7.63–7.55 (m, 0.5H), 7.49 (d, *J* = 8.0 Hz, 2H), 7.40–7.31 (m, 0.4H), 7.31–7.19 (m, 4H), 7.18–7.10 (m, 0.4H), 7.09–6.97 (m, 2.4H), 6.60 (br s, 1H), 5.02 (s, 1.3H), 4.66 (s, 0.7H), 3.58–3.29 (m, 0.7H), 3.09–2.83 (m, 1.3H), 1.17–1.01 (m, 0.3H), 0.95–0.69 (m, 0.6H), 0.61–0.39 (m, 2H), 0.30–0.11 (m, 0.7H), 0.04 – −0.19 (m, 1.3H). Rotamers observed in approximately 2:1 ratio. ACQUITY UPLC BEH C18 1.7 μm: *R*_t_ = 1.70 min; *m*/*z* 462.1 [M + H]^+^.

#### 1-(5-Bromo-2-pyridyl)-*N*-(cyclopropylmethyl)methanamine (**90a**)

Synthesized according to general procedure B. Cyclopropylmethylamine (0.37 mL, 4.22 mmol, 1 equiv used), 5-bromo-pyridine-2-carbaldehyde (942 mg, 5.06 mmol, 1.2 equiv used), NaHCO_3_ (1.06 g, 12.7 mmol), MeOH (8 mL), NaBH_4_ (192 mg, 5.06 mmol). Yield: 1.21 g, 4.02 mmol, 95.3% (80% purity). Yellow oil. ACQUITY UPLC BEH C18 1.7 μm: *R*_t_ = 0.52 min, *m*/*z* 241.1 [M + H, ^79^Br]^+^, 243.1 [M + H, ^81^Br]^+^. Product was taken forward directly as crude without further characterization.

#### *N*-[(5-Bromo-2-pyridyl)methyl]-*N*-(cyclopropylmethyl)-4-(phenylsulfamoyl)benzamide (**63**)

Synthesized according to general procedure D. 4-(Phenylsulfamoyl)benzoyl chloride (800 mg, 2.71 mmol, Key Intermediate A), 1-(5-bromo-2-pyridyl)-*N*-(cyclopropylmethyl)methanamine (1.18 g, 4.06 mmol), Et_3_N (0.57 mL, 4.06 mmol). Purified by flash column chromatography (silica, 12 g, 0:1 DCM/10% MeOH in DCM to 1:1 DCM/10% MeOH in DCM over 25 CV’s). Yield: 1.16 g, 2.09 mmol, 77% yield (90% purity). Colorless solid. ^1^H NMR (500 MHz, CDCl_3_): δ 8.67–8.55 (m, 1H), 7.82–7.75 (m, 2H), 7.73–7.68 (m, 1H), 7.53 (d, *J* = 8.0 Hz, 0.8H), 7.47 (d, *J* = 8.1 Hz, 1.2H), 7.29 (d, *J* = 8.3 Hz, 0.5H), 7.25–7.18 (m, 2H), 7.13 (t, *J* = 7.2 Hz, 1H), 7.07–6.96 (m, 2.5H), 6.96–6.87 (m, 1H), 4.93 (s, 1.2H), 4.54 (s, 0.8H), 3.40 (d, *J* = 7.0 Hz, 0.7H), 3.07 (d, *J* = 6.7 Hz, 1.2H), 1.10–0.96 (m, 0.4H), 0.88–0.74 (m, 0.6H), 0.55–0.39 (m, 2H), 0.25–0.12 (m, 0.8H), 0.02– −0.07 (m, 1.2H). Rotamers observed in approximately 3:2 ratio. ACQUITY UPLC BEH C18 1.7 μm: *R*_t_ = 1.79 min; *m*/*z* 500.1 [M + H, ^79^Br]^+^, 502.1 [M + H, ^81^Br]^+^.

#### (*S*)-*N*-(Cyclopropylmethyl)-1-phenylethan-1-amine

Synthesized according to general procedure B. (*S*)-1-Phenylethan-1-amine (0.11 mL, 0.83 mmol), cyclopropanecarbaldehyde (0.07 mL, 0.99 mmol), NaHCO_3_ (208 mg, 2.48 mmol), MeOH (4 mL), NaBH_4_ (38 mg, 0.99 mmol). Yield: 33 mg, 0.18 mmol, 22%. Colorless oil. ^1^H NMR (500 MHz, CDCl_3_): δ 7.34–7.28 (m, 4H), 7.25–7.20 (m, 1H), 3.78 (q, *J* = 6.6 Hz, 1H), 2.39 (dd, *J* = 11.9, 6.7 Hz, 1H), 2.23 (dd, *J* = 11.9, 7.1 Hz, 1H), 1.36 (d, *J* = 6.6 Hz, 3H), 1.00–0.87 (m, 1H), 0.49–0.38 (m, 2H), 0.10 – −0.03 (m, 2H). NH not observed. ACQUITY UPLC BEH C18 1.7 μm: *R*_t_ = 0.42 min; *m*/*z* 176.2 [M + H]^+^.

#### (*S*)-*N*-(Cyclopropylmethyl)-*N*-(1-phenylethyl)-4-(*N*-phenylsulfamoyl)benzamide (**64**)

Synthesized according to general procedure C. 4-(Phenylsulfamoyl)benzoic acid (50 mg, 0.18 mmol), HOBt hydrate (30 mg, 0.20 mmol), EDC.HCl (41 mg, 0.21 mmol), (*S*)-*N*-(cyclopropylmethyl)-1-phenylethan-1-amine (33 mg, 0.18 mmol), DCM (2 mL). Purified by flash column chromatography (silica, 12 g, 0:1 EtOAc/petrol to 1:1 EtOAc/petrol). Yield: 7.2 mg, 0.02 mmol, 9%. Colorless solid. ^1^H NMR (500 MHz, CDCl_3_): δ 7.78 (d, *J* = 8.1 Hz, 2H), 7.56–7.47 (m, 2H), 7.36–7.31 (m, 2H), 7.30–7.21 (m, 5.4H), 7.16–7.11 (m, 1H), 7.07–7.03 (m, 1H), 6.52 (br s, 1H), 6.18–5.80 (m, 0.3H), 4.97–4.71 (m, 0.5H), 3.53–3.22 (m, 0.5H), 2.85–2.73 (m, 1H), 1.55 (s, 3H), 1.33–1.19 (m, 0.5H), 1.11–0.92 (m, 0.5H), 0.55 – −0.07 (m, 4H). Rotamers observed in approximately 1:1 ratio. ACQUITY UPLC BEH C18 1.7 μm: *R*_t_ = 1.86 min; *m*/*z* 435.2 [M + H]^+^.

#### (*R*)-*N*-(Cyclopropylmethyl)-1-phenylethan-1-amine

Synthesized according to general procedure B. (*R*)-1-Phenylethan-1-amine (0.11 mL, 0.83 mmol), cyclopropanecarbaldehyde (0.07 mL, 0.99 mmol), NaHCO_3_ (208 mg, 2.48 mmol), MeOH (4 mL). NaBH_4_ (38 mg, 0.99 mmol). Yield: 121 mg, 0.66 mmol, 80%. Colorless oil. ACQUITY UPLC BEH C18 1.7 μm: *R*_t_ = 0.44 min; *m*/*z* 176.2 [M + H]^+^.

#### (*R*)-*N*-(Cyclopropylmethyl)-*N*-(1-phenylethyl)-4-(*N*-phenylsulfamoyl)benzamide (**65**)

Synthesized according to general procedure C. 4-(Phenylsulfamoyl)benzoic acid (182 mg, 0.66 mmol), HOBt hydrate (111 mg, 0.72 mmol), EDC.HCl (151 mg, 0.79 mmol), (*R*)-*N*-(cyclopropylmethyl)-1-phenylethan-1-amine (121 mg, 0.66 mmol), DCM (5 mL). Purified by flash column chromatography (silica, 12 g, 0:1 EtOAc/petrol to 1:1 EtOAc/petrol). Yield: 19 mg, 0.04 mmol, 6%. Colorless solid. ^1^H NMR (500 MHz, CDCl_3_): δ 7.78 (d, *J* = 7.9 Hz, 2H), 7.54–7.47 (m, 2H), 7.37–7.31 (m, 2H), 7.31–7.21 (m, 5.4H), 7.16–7.10 (m, 1H), 7.08–7.03 (m, 2H), 6.56 (br s, 1H), 6.15–5.82 (m, 0.4H), 5.02–4.64 (m, 0.7H), 3.47–3.22 (m, 0.6H), 2.79 (dd, *J* = 14.4, 6.6 Hz, 1H), 1.63 (s, 3H), 1.32–1.20 (m, 0.3H), 1.12–0.93 (m, 0.5H), 0.58 – −0.08 (m, 4H). Rotamers observed in approximately 1:1 ratio. ACQUITY UPLC BEH C18 1.7 μm: *R*_t_ = 1.86 min; *m*/*z* 435.2 [M + H]^+^.

#### 1-Cyclopropyl-*N*-((5-fluoropyridin-2-yl)methyl)methanamine (**88b**)

Synthesized according to general procedure B. Cyclopropylmethylamine (35 μL, 0.40 mmol, 1 equiv used), 5-fluoro-2-formylpyridine (61 mg, 0.49 mmol, 1.2 equiv used). NaBH_4_ (22 mg, 0.60 mmol, 1.5 equiv used). The reaction mixture was left for 2 days after NaBH_4_ addition and quenched with 2 M NaOH (1 mL). Product was taken forward as crude to the next step.

#### *N*-(Cyclopropylmethyl)-*N*-((5-fluoropyridin-2-yl)methyl)-4-(*N*-phenylsulfamoyl)benzamide (**71**)

Synthesized according to general procedure D. 4-(Phenylsulfamoyl)benzoyl chloride (80 mg, 0.27 mmol, Key Intermediate A), Et_3_N (55 μL, 0.39 mmol). Washed with 0.6 M citric acid (3 mL) instead of 1 M HCl. Purified by flash column chromatography (silica, 4 g, 1:0 petrol/EtOAc to 3:7 petrol/EtOAc). The residue was suspended in a mixture of TBME/petroleum ether and filtered followed by additional purification by trituration (TBME and petroleum ether). Yield: 63 mg, 0.13 mmol, 48%. Colorless glass. ^1^H NMR (500 MHz, CDCl_3_): δ 8.39 (s, 1H), 7.76 (d, *J* = 8.0 Hz, 1.2H), 7.70 (d, *J* = 8.0 Hz, 0.6H), 7.56–7.30 (m, 5H), 7.24–7.16 (m, 2H), 7.13–7.01 (m, 3H), 4.97 (s, 1.3H), 4.57 (s, 0.8H), 3.40 (d, *J* = 6.9 Hz, 0.7H), 3.08 (d, *J* = 6.8 Hz, 1.3H), 1.09–0.97 (m, 0.4H), 0.87–0.76 (m, 0.7H), 0.56–0.38 (m, 2H), 0.25–0.15 (m, 0.8H), 0.01 – −0.09 (m, 1.2H). Rotamers observed in approximately 3:2 ratio. ACQUITY UPLC BEH C18 1.7 μm: *R*_t_ = 1.71 min; *m*/*z* 440.2 [M + H]^+^

#### *N*-[(5-Amino-2-pyridyl)methyl]-*N*-(cyclopropylmethyl)-4-(phenylsulfamoyl)benzamide (**72**)

Synthesized according to general procedure F. *N*-[(5-Bromo-2-pyridyl)methyl)-*N*-(cyclopropylmethyl)-4-(phenylsulfamoyl)benzamide (100 mg, 0.18 mmol), 7 N NH_3_ in MeOH (7 μL, 0.36 mmol), CuI (3.4 mg, 0.02 mmol), l-proline (4.1 mg, 0.04 mmol), K_2_CO_3_ (50 mg, 0.36 mmol). Purified by flash column chromatography (12 g, silica, 0:1 EtOAc/petrol +1% Et_3_N to 1:0 EtOAc/petrol +1% Et_3_N over 25 CV’s, following by gradient of 1:0 DCM/10% MeOH in DCM +1% Et_3_N to 9:1 DCM/10% MeOH in DCM +1% Et_3_N over 10 CV’s). Yield: 12 mg, 0.02 mmol, 13%. Light yellow solid. ^1^H NMR (500 MHz, CDCl_3_): δ 8.04–8.00 (m, 1H), 7.77 (d, *J* = 8.0 Hz, 1H), 7.71 (d, *J* = 8.1 Hz, 1H), 7.57 (d, *J* = 8.0 Hz, 1H), 7.46 (d, *J* = 7.9 Hz, 1H), 7.28–7.09 (m, 4H), 7.09–7.01 (m, 1.5H), 7.00–6.90 (m, 1H), 6.84 (d, *J* = 8.3 Hz, 0.5H), 4.88 (s, 1H), 4.46 (s, 1H), 3.78–3.61 (m, 2H), 3.38 (d, *J* = 7.0 Hz, 1H), 3.00 (d, *J* = 6.7 Hz, 1H), 2.93 (br s, 1H), 1.10–0.99 (m, 0.5H), 0.87–0.75 (m, 0.6H), 0.55–0.47 (m, 1H), 0.45–0.38 (m, 1H), 0.25–0.16 (m, 1H), −0.01––0.10 (m, 1H). Rotamers observed in approximately 1:1 ratio. ACQUITY UPLC BEH C18 1.7 μm: *R*_t_ = 1.40 min; *m*/*z* 437.3 [M + H]^+^.

#### *N*-(Cyclopropylmethyl)-*N*-[(5-morpholino-2-pyridyl)methyl]-4-(phenylsulfamoyl)benzamide (**73**)

Synthesized according to general procedure F. *N*-[(5-Bromo-2-pyridyl)methyl)-*N*-(cyclopropylmethyl)-4-(phenylsulfamoyl)benzamide (100 mg, 0.18 mmol), morpholine (31.1 μL, 0.36 mmol), CuI (3.4 mg, 0.02 mmol), l-proline (4.1 mg, 0.04 mmol), K_2_CO_3_ (50 mg, 0.36 mmol). Purified by flash column chromatography (silica, 12 g, 0:1 EtOAc/petrol to 1:0 EtOAc/petrol over 25 CV’s). Yield: 25 mg, 0.05 mmol, 26%. Colorless solid. ^1^H NMR (500 MHz, CDCl_3_): δ 8.27–8.16 (m, 1H), 7.76 (d, *J* = 7.9 Hz, 1H), 7.71 (d, *J* = 8.1 Hz, 1H), 7.58 (d, *J* = 8.0 Hz, 1H), 7.47 (d, *J* = 8.0 Hz, 1H), 7.31–7.09 (m, 4H), 7.07–7.00 (m, 2.5H), 6.98–6.90 (m, 0.5H), 6.74–6.63 (m, 1H), 4.92 (s, 1H), 4.50 (s, 1H), 3.93–3.81 (m, 4H), 3.39 (d, *J* = 7.0 Hz, 1H), 3.25–3.12 (m, 4H), 3.02 (d, *J* = 6.8 Hz, 1H), 1.11–0.98 (m, 0.5H), 0.90–0.75 (m, 0.6H), 0.54–0.48 (m, 1H), 0.46–0.41 (m, 1H), 0.30–0.12 (m, 1H), 0.05 – −0.12 (m, 1H). Rotamers observed in approximately 1:1 ratio. ACQUITY UPLC BEH C18 1.7 μm: *R*_t_ = 1.53 min; *m*/*z* 507.3 [M + H]^+^.

#### *N*-(Cyclopropylmethyl)-*N*-[[5-(2-hydroxyethylamino)-2-pyridyl]methyl]-4-(phenylsulfamoyl)benzamide (**74**)

Synthesized according to general procedure F. *N*-[(5-Bromo-2-pyridyl)methyl)-*N*-(cyclopropylmethyl)-4-(phenylsulfamoyl)benzamide (100 mg, 0.18 mmol), ethanolamine (21.7 μL, 0.36 mmol), CuI (3.4 mg, 0.02 mmol), l-proline (4.1 mg, 0.04 mmol), K_2_CO_3_ (50 mg, 0.36 mmol). Purified by flash column chromatography (silica, 12 g, 0:1 DCM/10% MeOH in DCM to 1:1 DCM/10% MeOH in DCM over 25 CV’s). Yield: 42 mg, 0.08 mmol, 46%. Colorless solid. ^1^H NMR (500 MHz, CDCl_3_): δ 8.02–7.93 (m, 1H), 7.75 (d, *J* = 8.0 Hz, 1H), 7.70 (d, *J* = 8.1 Hz, 1H), 7.57 (d, *J* = 8.0 Hz, 1H), 7.49–7.44 (m, 1H), 7.30–7.17 (m, 2.5H), 7.16–7.09 (m, 1H), 7.07–7.01 (m, 2H), 6.95–6.89 (m, 0.5H), 6.86 (s, 1H), 6.80–6.67 (m, 1H), 4.88 (s, 1H), 4.46 (s, 1H), 4.19–4.13 (m, 0.6H), 4.12–4.07 (m, 0.7H), 3.86 (t, *J* = 5.0 Hz, 2H), 3.39 (d, *J* = 7.0 Hz, 1H), 3.30 (q, *J* = 5.2 Hz, 2H), 3.01 (d, *J* = 6.7 Hz, 1H), 1.87 (br s, 1H), 1.11–1.00 (m, 0.6H), 0.86–0.77 (m, 0.6H), 0.54–0.47 (m, 1H), 0.46–0.40 (m, 1H), 0.25–0.17 (m, 1H), 0.01––0.10 (m, 1H). Rotamers observed in approximately 1:1 ratio. ACQUITY UPLC BEH C18 1.7 μm: *R*_t_ = 1.38 min; *m*/*z* 481.3 [M + H]^+^.

#### *N*-(Cyclopropylmethyl)-*N*-[[5-(2-methoxyethylamino)-2-pyridyl]methyl]-4-(phenylsulfamoyl)benzamide (**75**)

Synthesized according to general procedure F. *N*-[(5-Bromo-2-pyridyl)methyl)-*N*-(cyclopropylmethyl)-4-(phenylsulfamoyl)benzamide (263 mg, 0.47 mmol), 2-methoxyethanamine (0.12 mL, 1.42 mmol), CuI (9.1 mg, 0.05 mmol), l-proline (11 mg, 0.09 mmol), K_2_CO_3_ (199 mg, 1.42 mmol). Reaction performed at 100 °C. Purified by flash column chromatography (silica, 12 g, 1:0 DCM/10% MeOH in DCM to 1:1 DCM/10% MeOH in DCM over 25 CV’s) followed by reverse-phase chromatography (9:1 H_2_O/MeOH to 0:1 H_2_O/MeOH for 20 min). Yield: 97 mg, 0.19 mmol, 39%. Light yellow solid. ^1^H NMR (500 MHz, DMSO-*d*_6_): δ 10.34 (br s, 1H), 7.95 (s, 0.5H), 7.92 (d, *J* = 3.1 Hz, 0.5H), 7.78 (d, *J* = 8.1 Hz, 1H), 7.74 (d, *J* = 8.3 Hz, 1H), 7.62 (d, *J* = 8.1 Hz, 1H), 7.55 (d, *J* = 8.1 Hz, 1H), 7.25–7.17 (m, 2H), 7.11–6.99 (m, 3.5H), 6.95 (dd, *J* = 8.5, 2.8 Hz, 0.5H), 6.87–6.84 (m, 1H), 5.91 (t, *J* = 5.6 Hz, 0.5H), 5.85 (t, *J* = 5.7 Hz, 0.4H), 4.69 (s, 1H), 4.31 (s, 1H), 3.47 (m, 2H), 3.27 (s, 3H), 3.22–3.15 (m, 3H), 2.89 (d, *J* = 6.7 Hz, 1H), 1.05–0.95 (m, 0.5H), 0.89–0.79 (m, 0.2H), 0.44–0.38 (m, 1H), 0.36–0.29 (m, 1H), 0.19–0.12 (m, 1H), −0.08 – −0.16 (m, 1H). Rotamers observed in approximately 1:1 ratio. ACQUITY UPLC BEH C18 1.7 μm: *R*_t_ = 1.45 min; *m*/*z* 495.2 [M + H]^+^.

#### *N*-(Cyclopropylmethyl)-*N*-[[5-(oxetan-3-ylamino)-2-pyridyl]methyl]-4-(phenylsulfamoyl)benzamide (**76**)

Synthesized according to general procedure F. *N*-[(5-Bromo-2-pyridyl)methyl)-*N*-(cyclopropylmethyl)-4-(phenylsulfamoyl)benzamide (100 mg, 0.18 mmol), 3-oxetanamine (33.6 μL, 0.48 mmol), CuI (3.4 mg, 0.02 mmol), l-proline (4.1 mg, 0.04 mmol), K_2_CO_3_ (76 mg, 0.54 mmol). Reaction performed at 100 °C. Purified by flash column chromatography (silica, 12 g, 1:0 DCM/10% MeOH in DCM to 1:1 DCM/10% MeOH in DCM over 25 CV’s). Yield: 12.8 mg, 0.02 mmol, 15%. Colorless solid. ^1^H NMR (400 MHz, CDCl_3_): δ 7.87 (d, *J* = 8.8 Hz, 1H), 7.80–7.67 (m, 2H), 7.58 (d, *J* = 8.1 Hz, 1H), 7.46 (d, *J* = 8.0 Hz, 1H), 7.32–7.18 (m, 2.5H), 7.17–7.09 (m, 1H), 7.07–7.01 (m, 2H), 6.86 (d, *J* = 8.3 Hz, 0.4H), 6.81–6.67 (m, 1H), 6.56 (br s, 1H), 5.01 (t, *J* = 6.5 Hz, 2H), 4.88 (s, 1H), 4.67–4.57 (m, 1H), 4.53 (t, *J* = 6.1 Hz, 2H), 4.47 (s, 1H), 4.26–4.16 (m, 1H), 3.37 (d, *J* = 7.0 Hz, 1H), 3.02 (d, *J* = 6.8 Hz, 1H), 1.11–0.96 (m, 0.5H), 0.90–0.76 (m, 0.7H), 0.55–0.39 (m, 2H), 0.26–0.16 (m, 1H), 0.02 – −0.08 (m, 1H). Rotamers observed in approximately 1:1 ratio. ACQUITY UPLC BEH C18 1.7 μm: *R*_t_ = 1.46 min; *m*/*z* 493.3 [M + H]^+^.

#### *N*-(Cyclopropylmethyl)-*N*-[[5-[2-hydroxyethyl(methyl)amino]-2-pyridyl]methyl]-4-(phenylsulfamoyl)benzamide (**77**)

Synthesized according to general procedure F. *N*-[(5-Bromo-2-pyridyl)methyl)-*N*-(cyclopropylmethyl)-4-(phenylsulfamoyl)benzamide (100 mg, 0.18 mmol), 2-(methylamino)ethanol (38.3 μL, 0.48 mmol), CuI (3.4 mg, 0.02 mmol), l-proline (4.1 mg, 0.04 mmol), K_2_CO_3_ (76 mg, 0.54 mmol). Reaction performed at 100 °C. Purified by flash column chromatography (silica, 12 g, 1:0 DCM/10% MeOH in DCM to 1:1 DCM/10% MeOH in DCM over 25 CV’s) followed by reverse-phase chromatography (9:1 H_2_O/MeOH to 0:1 H_2_O/MeOH for 20 min). Yield: 7.9 mg, 0.02 mmol, 10%. Colorless solid. ^1^H NMR (500 MHz, CDCl_3_): δ 8.11–8.05 (m, 1H), 7.74 (d, *J* = 8.0 Hz, 1H), 7.69 (d, *J* = 8.1 Hz, 1H), 7.56 (d, *J* = 8.0 Hz, 1H), 7.44 (d, *J* = 8.0 Hz, 1H), 7.24–7.17 (m, 3H), 7.13–7.07 (m, 1H), 7.06–7.01 (m, 3H), 7.00–6.94 (m, 0.5H), 6.87 (d, *J* = 8.6 Hz, 0.5H), 4.88 (s, 1H), 4.47 (s, 1H), 3.80 (q, *J* = 5.5 Hz, 2H), 3.48 (q, *J* = 5.1 Hz, 2H), 3.38 (d, *J* = 7.0 Hz, 1H), 3.03–2.95 (m, 4H), 1.12–0.99 (m, 0.50H), 0.87–0.77 (m, 0.50H), 0.54–0.47 (m, 1H), 0.46–0.38 (m, 1H), 0.26–0.17 (m, 1H), −0.01––0.09 (m, 1H). OH not observed. Rotamers observed in approximately 1:1 ratio. ACQUITY UPLC BEH C18 1.7 μm: *R*_t_ = 1.40 min; *m*/*z* 495.3 [M + H]^+^.

#### *N*-(Cyclopropylmethyl)-*N*-[[5-[2-(dimethylamino)ethylamino]-2-pyridyl]methyl]-4-(phenylsulfamoyl)benzamide (**78**)

Synthesized according to general procedure F. *N*-[(5-Bromo-2-pyridyl)methyl)-*N*-(cyclopropylmethyl)-4-(phenylsulfamoyl)benzamide (100 mg, 0.18 mmol), *N*,*N*-dimethylethylenediamine (58.9 μL, 0.54 mmol), CuI (3.4 mg, 0.02 mmol), l-proline (4.1 mg, 0.04 mmol), K_2_CO_3_ (76 mg, 0.54 mmol). Purified by flash column chromatography (silica, 12 g, 1:0 DCM/10% MeOH in DCM to 0:1 DCM/10% MeOH in DCM over 25 CV’s). Yield: 16 mg, 0.03 mmol, 17%. Colorless solid. ^1^H NMR (500 MHz, CDCl_3_): δ 7.99–7.93 (m, 1H), 7.75 (d, *J* = 8.0 Hz, 1H), 7.70 (d, *J* = 8.1 Hz, 1H), 7.58 (d, *J* = 8.0 Hz, 1H), 7.45 (d, *J* = 7.9 Hz, 1H), 7.25–7.16 (m, 2.5H), 7.15–7.07 (m, 1H), 7.06–7.00 (m, 2H), 6.91–6.79 (m, 1.5H), 4.88 (s, 1H), 4.46 (s, 1H), 4.44–4.41 (m, 0.5H), 4.40–4.36 (m, 0.4H), 3.38 (d, *J* = 6.9 Hz, 1H), 3.12 (q, *J* = 5.7 Hz, 2H), 2.99 (d, *J* = 6.7 Hz, 1H), 2.56 (dd, *J* = 6.6, 5.1 Hz, 2H), 2.25 (s, 6H), 1.12–0.99 (m, 0.50H), 0.86–0.77 (m, 0.50H), 0.52–0.47 (m, 1H), 0.45–0.39 (m, 1H), 0.25–0.17 (m, 1H), −0.01––0.10 (m, 1H). NH not observed. Rotamers observed in approximately 1:1 ratio. ACQUITY UPLC BEH C18 1.7 μm: *R*_t_ = 1.31 min; *m*/*z* 508.3 [M + H]^+^.

#### *N*-[[5-(2-Cyanoethyl)-2-pyridyl]methyl]-*N*-(cyclopropylmethyl)-4-(phenylsulfamoyl)benzamide (**92**)

*N*-[(5-Bromo-2-pyridyl)methyl]-*N*-(cyclopropylmethyl)-4-(phenylsulfamoyl)benzamide (300 mg, 0.54 mmol) was added to a solution of acrylonitrile (0.06 mL, 1.62 mmol), tetrabutylammonium bromide (174 mg, 0.54 mmol) and NaHCO_3_ (136 mg, 1.62 mmol) in DMF (3 mL). The reaction mixture was degassed with N_2_ for 5 min, before Pd(OAc)_2_ (6.1 mg, 0.03 mmol) was added. The reaction mixture was further degassed with N_2_ for another 5 min before heating to 110 °C for 4 h. The reaction mixture was concentrated under reduced pressure and the crude redissolved in DCM (20 mL). The organic layer was washed with water (2 × 20 mL) and brine (20 mL). The organic phase was separated using a phase separator and the filtrated was concentrated under reduced pressure to dryness. The crude mixture was dissolved in MeOH (10 mL). Palladium on carbon (5.7 mg, 0.05 mmol) was then added and the reaction mixture degassed with N_2_ for 5 min, followed by addition of triethylsilane (0.43 mL, 2.70 mmol). The reaction mixture was stirred at room temperature for 24 h. The reaction mixture was filtered through Celite and washed with MeOH (∼20 mL). The filtrate was concentrated under reduced pressure to dryness. The crude mixture was purified by reverse-phase column chromatography (1:9 MeOH/H_2_O to 1:0 MeOH/H_2_O for 25 min), followed by additional purification of the relevant concentrated fractions by flash column chromatography (silica, 12 g, 1:0 petrol/EtOAc +1% Et_3_N to 0:1 petrol/EtOAc +1% Et_3_N over 30 CV’s). Fractions containing product were combined and concentrated under reduced pressure to afford *N*-[[5-(2-cyanoethyl)-2-pyridyl]methyl]-*N*-(cyclopropylmethyl)-4-(phenylsulfamoyl)benzamide (111 mg, 0.21 mmol, 39% yield over two steps, 90% purity) as a colorless solid. ^1^H NMR (500 MHz, CDCl_3_): δ 8.44 (dd, *J* = 2.4, 0.8 Hz, 1H), 7.78 (d, *J* = 7.9 Hz, 1.1H), 7.69 (d, *J* = 8.0 Hz, 0.9H), 7.61–7.48 (m, 3.2H), 7.36 (d, *J* = 8.0 Hz, 0.7H), 7.25–7.21 (m, 2.4H), 7.17–7.10 (m, 1.1H), 7.09–6.99 (m, 2.3H), 5.05–4.92 (m, 1.1H), 4.59 (s, 0.9H), 3.43 (d, *J* = 7.0 Hz, 0.9H), 3.07 (d, *J* = 6.7 Hz, 1.1H), 2.97 (t, *J* = 7.2 Hz, 2H), 2.68–2.63 (m, 2H), 1.10–1.00 (m, 0.6H), 0.87–0.77 (m, 0.6H), 0.55–0.49 (m, 0.9H), 0.47–0.40 (m, 1.1H), 0.26–0.18 (m, 0.9H), 0.01 – −0.07 (m, 1.1H). Rotamers observed in approximately 3:2 ratio. ACQUITY UPLC BEH C18 1.7 μm: *R*_t_ = 1.63 min; *m*/*z* 475.3 [M + H]^+^.

#### *N*-(Cyclopropylmethyl)-4-(phenylsulfamoyl)-*N*-[[5-[2-(1*H*-tetrazol-5-yl)ethyl]-2-pyridyl]methyl]benzamide (**79**)

NaN_3_ (41 mg, 0.63 mmol) was added to a solution of *N*-[[5-(2-cyanoethyl)-2-pyridyl]methyl]-*N*-(cyclopropylmethyl)-4-(phenylsulfamoyl)benzamide (110 mg, 0.21 mmol), NH_4_Cl (33 mg, 0.63 mmol) and DMF (3 mL). The reaction mixture was heated to 120 °C and stirred overnight. The reaction mixture was diluted with water (1 mL) and cooled to 0 °C. A solution of aq NaNO_2_ (2.9 M, 1 mL) was added in one portion while stirring, followed by dropwise addition of aq H_2_SO_4_ (2 M, 1 mL) until no more gas evolution and solution was acidic (pH 1.5). The reaction mixture was then adjusted to pH 6–7 and the crude mixture purified by reverse-phase column chromatography (1:9 MeOH/H_2_O to 1:0 MeOH/H_2_O over 20 min). Fractions containing product were combined and concentrated under reduced pressure to afford *N*-(cyclopropylmethyl)-4-(phenylsulfamoyl)-*N*-[[5-[2-(1*H*-tetrazol-5-yl)ethyl]-2-pyridyl]methyl]benzamide (72 mg, 0.13 mmol, 63% yield) as a colorless solid. ^1^H NMR (500 MHz, DMSO-*d*_6_): δ 10.33 (br s, 1H), 8.37 (d, *J* = 2.2 Hz, 1H), 7.80 (d, *J* = 8.0 Hz, 1.1H), 7.71 (d, *J* = 7.9 Hz, 0.9H), 7.64–7.58 (m, 1.6H), 7.57–7.53 (m, 1.4H), 7.29 (d, *J* = 8.0 Hz, 0.6H), 7.26–7.18 (m, 2.3H), 7.12–6.98 (m, 4H), 4.80 (s, 1.2H), 4.46 (s, 0.9H), 3.27–3.17 (m, 2.3H), 3.09–3.01 (m, 2.2H), 2.97 (d, *J* = 6.8 Hz, 1.1H), 1.04–0.91 (m, 0.5H), 0.89–0.73 (m, 0.6H), 0.43–0.34 (m, 0.9H), 0.33–0.24 (m, 1.2H), 0.17–0.09 (m, 1H), −0.09 – −0.18 (m, 1.2H). Rotamers observed in approximately 3:2 ratio. ACQUITY UPLC BEH C18 1.7 μm: *R*_t_ = 1.53 min; *m*/*z* 518.3 [M + H]^+^.

#### *N*-[(5-Bromo-2-pyridyl)methyl]ethanamine

Synthesized according to general procedure B. Ethylamine solution in THF (2M, 1.61 mL, 3.23 mmol). 5-Bromo-pyridine-2-carbaldehyde (500 mg, 2.69 mmol), NaHCO_3_ (677 mg, 8.06 mmol), NaBH_4_ (122 mg, 3.23 mmol). Yield: 578 mg, 2.69 mmol, 100% yield. Pink liquid. ACQUITY UPLC BEH C18 1.7 μm: *R*_t_ = 0.33 min; *m*/*z* 215.0 [M + H, ^79^Br]^+^, 217.0 [M + H, ^81^Br]^+^.

#### *N*-[(5-Bromo-2-pyridyl)methyl]-*N*-ethyl-4-(phenylsulfamoyl)benzamide

Synthesized according to general procedure C. 4-(Phenylsulfamoyl)benzoic acid (400 mg, 1.44 mmol), HOBt hydrate (243 mg, 1.59 mmol), EDC.HCl (332 mg, 1.73 mmol), *N*-[(5-bromo-2-pyridyl)methyl]ethanamine (574 mg, 2.67 mmol). Purified by flash column chromatography (silica, 12 g, 1:0 petrol/EtOAc to 0:1 petrol/EtOAc over 25 CV’s). Yield: 561 mg, 1.12 mmol, 78%. Off-white solid. ^1^H NMR (500 MHz, CDCl_3_): δ 8.66–8.57 (m, 1H), 7.84–7.76 (m, 2.3H), 7.71 (d, *J* = 8.1 Hz, 0.8H), 7.52 (d, *J* = 8.0 Hz, 0.8H), 7.47 (d, *J* = 7.8 Hz, 1.3H), 7.32 (d, *J* = 8.3 Hz, 0.6H), 7.28–7.20 (m, 2.3H), 7.17–7.10 (m, 0.3H), 7.08–6.95 (m, 2.5H), 6.61–6.49 (m, 1H), 4.77 (s, 1.3H), 4.40 (s, 0.7H), 3.53 (q, *J* = 7.8 Hz, 0.8H), 3.25 (q, *J* = 7.5 Hz, 1.3H), 1.22–1.15 (m, 1.4H), 1.07 (t, *J* = 7.2 Hz, 1.9H). Rotamers observed in approximately 3:2 ratio. ACQUITY UPLC BEH C18 1.7 μm: *R*_t_ = 1.74 min; *m*/*z* 474.0 [M + H, ^79^Br]^+^, 476.0 [M + H, ^81^Br]^+^.

#### *N*-Ethyl-*N*-[[5-(2-hydroxyethylamino)-2-pyridyl]methyl]-4-(phenylsulfamoyl)benzamide (**80**)

Synthesized according to general procedure F. *N*-[(5-Bromo-2-pyridyl)methyl]-*N*-ethyl-4-(phenylsulfamoyl)benzamide (125 mg, 0.25 mmol), ethanolamine (45.3 μL, 0.75 mmol), CuI (4.8 mg, 0.03 mmol), l-proline (5.8 mg, 0.05 mmol), K_2_CO_3_ (105 mg, 0.75 mmol). Purified by flash column chromatography (silica, 12 g, 1:0 DCM/10% MeOH in DCM to 1:4 DCM/10% MeOH in DCM over 25 CV’s). Additionally purified by reverse-phase chromatography (9:1H_2_O/MeOH to 0:1H_2_O/MeOH for 20 min). Yield: 63 mg, 0.13 mmol, 53%. Off-white solid. ^1^H NMR (500 MHz, CDCl_3_): δ 7.99–7.95 (m, 0.5H), 7.95–7.91 (m, 0.5H), 7.75 (d, *J* = 8.0 Hz, 1H), 7.69 (d, *J* = 8.0 Hz, 1H), 7.55 (d, *J* = 8.0 Hz, 1H), 7.44 (d, *J* = 8.0 Hz, 1H), 7.25–7.18 (m, 3H), 7.16–7.08 (m, 1H), 7.07–7.00 (m, 2.3H), 6.94–6.89 (m, 0.5H), 6.87–6.82 (m, 1H), 4.72 (s, 1H), 4.33 (s, 1H), 4.20 (t, *J* = 6.0 Hz, 0.5H), 4.17–4.12 (m, 0.2H), 3.88–3.82 (m, 2H), 3.53 (q, *J* = 7.1 Hz, 1H), 3.28 (q, *J* = 5.5 Hz, 2H), 3.19 (q, *J* = 7.1 Hz, 1H), 1.17 (t, *J* = 7.1 Hz, 1.5H), 1.03 (t, *J* = 7.0 Hz, 1.6H). NH not observed. Rotamers observed in approximately 1:1 ratio. ACQUITY UPLC BEH C18 1.7 μm: *R*_t_ = 1.33 min; *m*/*z* 455.2 [M + H]^+^.

#### *N*-(Cyclopropylmethyl)-*N*-[[5-[(2-hydroxy-1,1-dimethyl-ethyl)amino]-2-pyridyl]methyl]-4-(phenylsulfamoyl)benzamide (**81**)

Synthesized according to general procedure F. *N*-[(5-Bromo-2-pyridyl)methyl)-*N*-(cyclopropylmethyl)-4-(phenylsulfamoyl)benzamide (125 mg, 0.24 mmol), 2-amino-2-methyl-1-propanol (67.9 μL, 0.71 mmol), CuI (4.5 mg, 0.03 mmol), l-proline (5.5 mg, 0.05 mmol), K_2_CO_3_ (100 mg, 0.71 mmol). Purified by flash column chromatography (silica, 12 g, 1:0 DCM/10% MeOH in DCM to 1:4 DCM/10% MeOH in DCM over 25 CV’s) followed by reverse-phase chromatography (9:1H_2_O/MeOH to 0:1H_2_O/MeOH for 20 min). Yield: 16 mg, 0.03 mmol, 12%. Colorless solid. ^1^H NMR (500 MHz, CDCl_3_): δ 8.03 (s, 1H), 7.74 (d, *J* = 8.0 Hz, 1H), 7.69 (d, *J* = 8.0 Hz, 1H), 7.51 (d, *J* = 7.9 Hz, 1H), 7.45–7.38 (m, 1H), 7.22–7.11 (m, 3H), 7.10–6.98 (m, 4.5H), 6.84–6.78 (m, 0.5H), 4.86 (s, 1H), 4.46 (s, 1H), 3.82 (s, 0.5H), 3.74 (s, 0.5H), 3.57–3.49 (m, 2H), 3.39 (d, *J* = 7.0 Hz, 1H), 3.00 (d, *J* = 6.7 Hz, 1H), 1.26 (s, 6H), 1.11–0.99 (m, 0.5H), 0.85–0.73 (m, 0.7H), 0.54–0.46 (m, 1H), 0.44–0.36 (m, 1H), 0.26–0.17 (m, 1H), −0.02 – −0.13 (m, 1H). NH not observed. Rotamers observed in approximately 1:1 ratio. ACQUITY UPLC BEH C18 1.7 μm: *R*_t_ = 1.43 min; *m*/*z* 509.2 [M + H]^+^.

#### 1-(5-Bromo-6-methyl-2-pyridyl)-*N*-(cyclopropylmethyl)methanamine (**90c**)

Synthesized according to general procedure B. Cyclopropylmethylamine (0.18 mL, 2.11 mmol, 1.7 equiv was used), 5-bromo-6-methylpicolinaldehyde (250 mg, 1.25 mmol, 1 equiv used), NaHCO_3_ (315 mg, 3.75 mmol), MeOH (5 mL), NaBH_4_ (57 mg, 1.50 mmol). Yield: 464 mg, 1.18 mmol, 95% (80% purity). Orange oil. ^1^H NMR (500 MHz, CDCl_3_): δ 7.73 (d, *J* = 8.1 Hz, 1H), 7.03 (dq, *J* = 8.1, 0.6 Hz, 1H), 3.85 (s, 2H), 2.64 (s, 3H), 2.50 (d, *J* = 6.9 Hz, 2H), 2.07 (br s, 1H), 1.03–0.93 (m, 1H), 0.51–0.45 (m, 2H), 0.14–0.09 (m, 2H). ACQUITY UPLC BEH C18 1.7 μm: *R*_t_ = 0.47 min; *m*/*z* 255.0 [M + H, ^79^Br]^+^, 257.0 [M + H, ^81^Br]^+^.

#### *N*-[(5-Bromo-6-methyl-2-pyridyl)methyl]-*N*-(cyclopropylmethyl)-4-(phenylsulfamoyl)benzamide (**91b**)

Synthesized according to general procedure D. 4-(Phenylsulfamoyl)benzoyl chloride (300 mg, 1.01 mmol, Key Intermediate A), 1-(5-bromo-6-methyl-2-pyridyl)-*N*-(cyclopropylmethyl)methanamine (478 mg, 1.22 mmol, 65% purity), Et_3_N (0.21 mL, 1.52 mmol). Purified by flash column chromatography (silica, 12 g, 0:1 EtOAc/petrol to 4:1 EtOAc/petrol over 25 CV’s). Yield: 395 mg, 0.69 mmol, 68% yield (90% purity). Colorless solid. ^1^H NMR (500 MHz, CDCl_3_): δ 7.85–7.66 (m, 3H), 7.54 (d, *J* = 7.9 Hz, 0.6H), 7.49 (d, *J* = 8.1 Hz, 1.2H), 7.27–7.19 (m, 2H), 7.17–7.08 (m, 2H), 7.07–6.99 (m, 2H), 6.80 (d, *J* = 8.0 Hz, 0.4H), 6.62 (s, 0.4H), 6.56 (s, 0.4H), 4.94 (s, 1.2H), 4.50 (s, 0.8H), 3.40 (d, *J* = 6.7 Hz, 1H), 3.12–3.04 (m, 1H), 2.74–2.54 (m, 3H), 1.09–0.96 (m, 0.5H), 0.86–0.75 (m, 0.5H), 0.55–0.47 (m, 0.9H), 0.47–0.38 (m, 1.3H), 0.26–0.13 (m, 1H), 0.04 – −0.09 (m, 1H). Rotamers observed in approximately 1:1 ratio. ACQUITY UPLC BEH C18 1.7 μm: *R*_t_ = 1.86 min; *m*/*z* 514.0 [M + H, ^79^Br]^+^, 516.0 [M + H, ^81^Br]^+^.

#### *N*-(Cyclopropylmethyl)-*N*-[[5-(2-hydroxyethylamino)-6-methyl-2-pyridyl]methyl]-4-(phenylsulfamoyl)benzamide (**82**)

Synthesized according to general procedure F. *N*-[(5-Bromo-6-methyl-2-pyridyl)methyl]-*N*-(cyclopropylmethyl)-4-(phenylsulfamoyl)benzamide (150 mg, 0.26 mmol), ethanolamine (47.5 μL, 0.79 mmol), CuI (5.0 mg, 0.03 mmol), l-proline (6.0 mg, 0.05 mmol), K_2_CO_3_ (110 mg, 0.79 mmol). Reaction performed at 100 °C. Purified by flash column chromatography (silica, 12 g, 0:1 EtOAc/petrol to 1:0 EtOAc/petrol over 20 CV’s, followed by gradient of 1:0 DCM/10% MeOH in DCM to 0:1 DCM/10% MeOH in DCM over 10 CV’s). Additionally purified by reverse-phase chromatography (9:1H_2_O/MeOH to 0:1H_2_O/MeOH for 20 min). Yield: 35 mg, 0.07 mmol, 26%. Colorless solid. ^1^H NMR (500 MHz, CDCl_3_): δ 7.82–7.78 (m, 0.3H), 7.75 (d, *J* = 7.9 Hz, 0.7H), 7.70 (d, *J* = 8.0 Hz, 1H), 7.59 (d, *J* = 8.0 Hz, 1H), 7.55–7.42 (m, 1.3H), 7.25–7.18 (m, 3.5H), 7.16–7.09 (m, 0.7H), 7.08–7.01 (m, 3H), 7.01–6.94 (m, 0.2H), 6.83 (br s, 1H), 4.95 (s, 1H), 4.47 (s, 1H), 3.90 (t, *J* = 5.1 Hz, 2H), 3.42–3.37 (m, 1H), 3.34–3.27 (m, 2H), 3.16–3.04 (m, 1H), 2.56–2.45 (m, 1.5H), 2.41–2.35 (m, 1.5H), 1.57 (br s, 1H), 1.13–0.99 (m, 0.7H), 0.88–0.76 (m, 0.7H), 0.55–0.46 (m, 1H), 0.46–0.37 (m, 1H), 0.29–0.15 (m, 1H), 0.03 – −0.09 (m, 1H). Rotamers observed in approximately 1:1 ratio. ACQUITY UPLC BEH C18 1.7 μm: *R*_t_ = 1.36 min; *m*/*z* 495.2 [M + H]^+^.

#### 1-(5-Bromo-4-methyl-2-pyridyl)-*N*-(cyclopropylmethyl)methanamine (**90d**)

Synthesized according to general procedure B. Cyclopropylmethylamine (0.18 mL, 2.11 mmol, 1.7 equiv was used), 5-bromo-4-methylpicolinaldehyde (250 mg, 1.25 mmol, 1 equiv used), NaHCO_3_ (315 mg, 3.75 mmol), MeOH (5 mL), NaBH_4_ (57 mg, 1.50 mmol). Yield: 351 mg, 1.24 mmol, 99% (90% purity). Orange oil. ^1^H NMR (500 MHz, CDCl_3_): δ 8.56 (s, 1H), 7.22 (s, 1H), 3.86 (s, 2H), 2.54–2.48 (m, 2H), 2.40–2.34 (s, 3H), 2.12–1.96 (m, 1H), 1.04–0.95 (m, 1H), 0.51–0.45 (m, 2H), 0.15–0.08 (m, 2H). ACQUITY UPLC BEH C18 1.7 μm: *R*_t_ = 0.48 min; *m*/*z* 255.0 [M + H, ^79^Br]^+^, 257.0 [M + H, ^81^Br]^+^.

#### *N*-[(5-Bromo-4-methyl-2-pyridyl)methyl]-*N*-(cyclopropylmethyl)-4-(phenylsulfamoyl)benzamide (**91c**)

Synthesized according to general procedure D. 4-(Phenylsulfamoyl)benzoyl chloride (300 mg, 1.01 mmol, Key Intermediate A), 1-(5-bromo-4-methyl-2-pyridyl)-*N*-(cyclopropylmethyl)methanamine (345 mg, 1.22 mmol, 90% purity), Et_3_N (0.21 mL, 1.52 mmol). Purified by flash column chromatography (silica, 12 g, 0:1 EtOAc/petrol to 4:1 EtOAc/petrol over 25 CV’s). Yield: 333 mg, 0.55 mmol, 54% yield (85% purity). Colorless solid. ^1^H NMR (500 MHz, CDCl_3_): δ 8.62–8.57 (m, 1H), 7.78 (d, *J* = 7.9 Hz, 1.2H), 7.71 (d, *J* = 7.8 Hz, 1H), 7.58–7.48 (m, 2H), 7.38–7.32 (m, 0.6H), 7.28–7.21 (m, 2H), 7.16–7.10 (m, 1H), 7.08–7.01 (m, 2H), 6.97–6.92 (m, 0.3H), 6.59 (s, 0.5H), 6.51 (s, 0.2H), 4.92 (s, 1.2H), 4.52 (s, 0.7H), 3.40 (d, *J* = 7.0 Hz, 0.7H), 3.16 (d, *J* = 6.9 Hz, 1.3H), 2.49–2.36 (m, 3H), 1.10–0.97 (m, 0.3H), 0.87–0.78 (m, 0.7H), 0.55–0.49 (m, 1H), 0.48–0.42 (m, 1H), 0.23–0.19 (m, 0.9H), 0.03 – −0.03 (m, 1.3H). Rotamers observed in approximately 2:1 ratio. ACQUITY UPLC BEH C18 1.7 μm: *R*_t_ = 1.84 min; *m*/*z* 514.0 [M + H, ^79^Br]^+^, 516.0 [M + H, ^81^Br]^+^.

#### *N*-(Cyclopropylmethyl)-*N*-[[5-(2-hydroxyethylamino)-4-methyl-2-pyridyl]methyl]-4-(phenylsulfamoyl)benzamide (**83**)

Synthesized according to general procedure F. *N*-[(5-Bromo-4-methyl-2-pyridyl)methyl]-*N*-(cyclopropylmethyl)-4-(phenylsulfamoyl)benzamide (150 mg, 0.25 mmol), ethanolamine (44.9 μL, 0.74 mmol), CuI (4.7 mg, 0.02 mmol), l-proline (5.7 mg, 0.05 mmol), K_2_CO_3_ (104 mg, 0.74 mmol). Reaction performed at 100 °C. Purified by flash column chromatography (silica, 12 g, 0:1 EtOAc/petrol to 1:0 EtOAc/petrol over 20 CV’s, followed by gradient of 1:0 DCM/10% MeOH in DCM to 0:1 DCM/10% MeOH in DCM over 10 CV’s). Additionally purified by reverse-phase chromatography (9:1H_2_O/MeOH to 0:1H_2_O/MeOH for 20 min). Yield: 29 mg, 0.06 mmol, 23%. Colorless solid. ^1^H NMR (500 MHz, CDCl_3_): δ 7.89 (s, 1H), 7.75 (d, *J* = 7.9 Hz, 1H), 7.71 (d, *J* = 8.0 Hz, 1H), 7.56 (d, *J* = 7.9 Hz, 1H), 7.50 (d, *J* = 7.9 Hz, 1H), 7.25–7.17 (m, 3H), 7.14–7.08 (m, 1H), 7.08–7.01 (m, 2.5H), 6.75 (br s, 0.5H), 4.90 (s, 1H), 4.48 (s, 1H), 3.95–3.84 (m, 2H), 3.42–3.30 (m, 3H), 3.18–3.09 (m, 1H), 2.19 (s, 1.6H), 2.14 (s, 1.3H), 1.12–0.99 (m, 0.4H), 0.87–0.77 (m, 0.7H), 0.54–0.48 (m, 1H), 0.46–0.38 (m, 1H), 0.28–0.18 (m, 1H), 0.03––0.08 (m, 1H). NH and OH not observed. Rotamers observed in approximately 1:1 ratio. ACQUITY UPLC BEH C18 1.7 μm: *R*_t_ = 1.37 min; *m*/*z* 495.2 [M + H]^+^.

#### 4-[Methyl(phenyl)sulfamoyl]benzoic Acid (**94**)

Synthesized according to general procedure A. 4-(Chlorosulfonyl)benzoic acid (1.00 g, 4.53 mmol), *N*-methylaniline (2.46 mL, 22.7 mmol). Yield: 785 mg, 2.56 mmol, 57%. Off-white solid. ^1^H NMR (500 MHz, DMSO-*d*_6_): δ 13.51 (s, 1H), 8.12–8.06 (m, 2H), 7.64–7.60 (m, 2H), 7.39–7.28 (m, 3H), 7.13–7.08 (m, 2H), 3.17 (s, 3H). ACQUITY UPLC BEH C18 1.7 μm: *R*_t_ = 1.68 min; *m*/*z* 289.9 [M + H]^+^.

#### *N*-[(5-Bromo-2-pyridyl)methyl]-*N*-(cyclopropylmethyl)-4-[methyl(phenyl)sulfamoyl]benzamide (**95**)

To a solution of 4-[methyl(phenyl)sulfamoyl]benzoic acid (780 mg, 2.54 mmol) in DCM (5 mL) was added oxalyl chloride (0.26 mL, 3.05 mmol) and DMF (30 μL). The reaction mixture was stirred at 0 °C overnight, allowing to warm to room temperature. The reaction mixture was concentrated under reduced pressure and used directly in the next step.

Synthesized according to general procedure D. 4-[Methyl(phenyl)sulfamoyl]benzoyl chloride (787 mg, 2.54 mmol), 1-(5-bromo-2-pyridyl)-*N*-(cyclopropylmethyl)methanamine (1.01 g, 4.19, 1.65 eq used), Et_3_N (0.53 mL, 3.81 mmol). Purified by flash column chromatography (silica, 12 g, 0:1 EtOAc/petrol to 1:0 EtOAc/petrol over 25 CV’s). Yield: 303 mg, 0.53 mmol, 21% (90% purity). Colorless glass. ^1^H NMR (500 MHz, CDCl_3_): δ 8.62 (s, 1H), 7.84–7.74 (m, 1H), 7.64–7.48 (m, 4H), 7.35–7.23 (m, 3.4H), 7.14–7.06 (m, 2.2H), 7.04–6.98 (m, 0.4H), 4.96 (s, 1.2H), 4.57 (s, 0.8H), 3.43 (d, *J* = 7.0 Hz, 0.7H), 3.23–3.15 (m, 3H), 3.10 (d, *J* = 6.8 Hz, 1.3H), 1.09–0.98 (m, 0.4H), 0.87–0.76 (m, 0.7H), 0.56–0.49 (m, 0.8H), 0.48–0.41 (m, 1.3H), 0.25–0.17 (m, 0.7H), 0.05––0.06 (m, 1.3H). Rotamers observed in approximately 2:1 ratio. ACQUITY UPLC BEH C18 1.7 μm: *R*_t_ = 1.86 min; *m*/*z* 514.0 [M + H, ^79^Br]^+^, 516.0 [M + H, ^81^Br]^+^.

#### *N*-(Cyclopropylmethyl)-*N*-[[5-(2-hydroxyethylamino)-2-pyridyl]methyl]-4-[methyl(phenyl)sulfamoyl]benzamide (**84**)

Synthesized according to general procedure F. *N*-[(5-Bromo-2-pyridyl)methyl]-*N*-(cyclopropylmethyl)-4-[methyl(phenyl)sulfamoyl]benzamide (99 mg, 0.17 mmol), ethanolamine (31.3 μL, 0.52 mmol), CuI (3.3 mg, 0.02 mmol), l-proline (4.0 mg, 0.03 mmol), K_2_CO_3_ (73 mg, 0.52 mmol). Purified by flash column chromatography (silica, 12 g, 1:0 DCM/10% MeOH in DCM to 0:1 DCM/10% MeOH in DCM over 25 CV’s). Additionally purified by reverse-phase chromatography (9:1 H_2_O/MeOH to 0:1 H_2_O/MeOH for 20 min). Yield: 46 mg, 0.09 mmol, 51%. Colorless solid. ^1^H NMR (500 MHz, CDCl_3_): δ 8.06 (s, 0.5H), 8.01 (s, 0.4H), 7.64–7.47 (m, 5H), 7.36 (d, *J* = 8.5 Hz, 0.5H), 7.32–7.21 (m, 2H), 7.13–7.03 (m, 2.5H), 6.89 (br s, 1H), 4.95 (s, 1.1H), 4.50 (s, 0.8H), 3.92–3.82 (m, 3H), 3.41 (d, *J* = 7.0 Hz, 1H), 3.35–3.26 (m, 2H), 3.23–3.07 (m, 5H), 1.14–1.00 (m, 0.3H), 0.92–0.77 (m, 0.5H), 0.55–0.49 (m, 1H), 0.48–0.41 (m, 1.4H), 0.28–0.19 (m, 0.7H), 0.07––0.04 (m, 1.3H). Rotamers observed in approximately 3:2 ratio. ACQUITY UPLC BEH C18 1.7 μm: *R*_t_ = 1.43 min; *m*/*z* 495.2 [M + H]^+^.

#### 1-(4-Bromophenyl)-*N*-(cyclopropylmethyl)methanamine (**90b**)

Synthesized according to general procedure B but reversed reagents for reductive amination. Cyclopropanecarbaldehyde (0.87 mL, 11.61 mmol), 4-bromobenzylamine (1.22 mL, 9.67 mmol). NaHCO_3_ (2.44 g, 29.02 mmol), MeOH (15 mL), NaBH_4_ (439 mg, 11.61 mmol). Yield: 1.64 g, 5.46 mmol, 56% (80% purity). Orange oil. ACQUITY UPLC BEH C18 1.7 μm: *R*_t_ = 1.21 min; *m*/*z* 240.0 [M + H, ^79^Br]^+^, 242.0 [M + H, ^81^Br]^+^.

#### *N*-[(4-Bromophenyl)methyl]-*N*-(cyclopropylmethyl)-4-(phenylsulfamoyl)benzamide (**91a**)

Synthesized according to general procedure D. 4-(Phenylsulfamoyl)benzoyl chloride (887 mg, 3.00 mmol, Key Intermediate A), 1-(4-bromophenyl)-*N*-(cyclopropylmethyl)methanamine (1.64 g, 5.46 mmol, 80% purity), Et_3_N (0.38 mL, 2.70 mmol). Purified by flash column chromatography (silica, 24 g, 0:1 EtOAc/petrol to 3:2 EtOAc/petrol over 40 CV’s). Yield: 1.03 g, 1.96 mmol, 65%. Colorless solid. ^1^H NMR (500 MHz, CDCl_3_): δ 7.81–7.70 (m, 2H), 7.49–7.43 (m, 4H), 7.26–7.18 (m, 2H), 7.17–7.12 (m, 1H), 7.08–7.02 (m, 2H), 6.97 (d, *J* = 7.9 Hz, 1H), 6.59 (s, 1H), 4.83 (s, 1.3H), 4.46 (s, 0.8H), 3.38 (d, *J* = 7.0 Hz, 0.8H), 2.92 (d, *J* = 6.6 Hz, 1H), 1.13–0.97 (m, 0.6H), 0.87–0.74 (m, 1.6H), 0.56–0.43 (m, 2H), 0.24–0.17 (m, 0.7H), −0.08 (d, *J* = 5.1 Hz, 1.4H). Rotamers observed in approximately 3:2 ratio. ACQUITY UPLC BEH C18 1.7 μm: *R*_t_ = 1.85 min; *m*/*z* 498.9 [M + H, ^79^Br]^+^, 500.9 [M + H, ^81^Br]^+^.

#### *N*-(Cyclopropylmethyl)-*N*-[[4-(2-hydroxyethylamino)phenyl]methyl]-4-(phenylsulfamoyl)benzamide (MDI-114215, **85**)

Synthesized according to general procedure F. *N*-[(5-Bromo-4-methyl-2-pyridyl)methyl]-*N*-(cyclopropylmethyl)-4-(phenylsulfamoyl)benzamide (1.32 g, 2.64 mmol), ethanolamine (0.48 mL, 7.91 mmol, 3 equiv used), CuI (51 mg, 0.26 mmol), l-proline (61 mg, 0.53 mmol), K_2_CO_3_ (1.11 g, 7.91 mmol, 3 equiv used), DMSO (27 mL). Reaction performed at 100 °C. Purified by flash column chromatography (silica, 12 g, 1:0 DCM/EtOAc to 0:1 DCM/EtOAc over 42 CV’s). Yield: 542 mg, 1.07 mmol, 41%. Colorless solid. ^1^H NMR (500 MHz, DMSO-*d*_6_): δ 10.32 (s, 1H), 7.77 (d, *J* = 8.0 Hz, 2H), 7.55–7.51 (m, 2H), 7.24–7.19 (m, 2H), 7.11–6.99 (m, 4H), 6.80 (d, *J* = 8.0 Hz, 1H), 6.59–6.46 (m, 2H), 5.54–5.45 (m, 1H), 4.66 (t, *J* = 5.5 Hz, 1H), 4.61 (s, 1.1H), 4.24 (s, 0.9H), 3.57–3.50 (m, 2H), 3.19 (d, *J* = 6.9 Hz, 0.9H), 3.09–3.01 (m, 2H), 2.80 (d, *J* = 6.7 Hz, 1.1H), 1.03 (s, 0.5H), 0.82 (s, 0.6H), 0.43 (d, *J* = 7.8 Hz, 0.8H), 0.34 (d, *J* = 7.8 Hz, 1.2H), 0.17 (s, 0.8H), −0.16 (d, *J* = 5.4 Hz, 1.1H). Rotamers observed in a 1:1 ratio. ACQUITY UPLC BEH C18 1.7 μm: *R*_t_ = 1.57 min; *m*/*z* 480.2 [M + H]^+^.

#### *N*-(Cyclopropylmethyl)-*N*-[[4-(2-hydroxyethoxy)phenyl]methyl]-4-(phenylsulfamoyl)benzamide (**86**)

Lithium *tert*-butoxide (69 mg, 0.86 mmol) was added to ethylene glycol (1.00 mL, 18.21 mmol) and the resulting suspension was stirred at room temperature for 5 min to form a clear solution. *N*-[(5-Bromo-4-methyl-2-pyridyl)methyl]-*N*-(cyclopropylmethyl)-4-(phenylsulfamoyl)benzamide (150 mg, 0.29 mmol) and CuI (5.4 mg, 0.03 mmol) were then added and the mixture stirred for a further 5 min, before heating to 110 °C and stirring overnight. The reaction mixture was cooled to room temperature and quenched with AcOH (until pH = 7–8). The reaction mixture was diluted with DCM (10 mL), washed with saturated NaHCO_3_ (10 mL) and separated using a phase separator. The filtrate was concentrated under reduced pressure with silica. The crude mixture was purified by flash column chromatography (silica, 12 g, 1:0 petrol/EtOAc to 0:1 petrol/EtOAc over 25 CV’s), followed by additional purification by reverse-phase chromatography (9:1H_2_O/MeOH to 0:1H_2_O/MeOH over 20 min). Fractions containing product were combined and concentrated under reduced pressure to afford *N*-(cyclopropylmethyl)-*N*-[[4-(2-hydroxyethoxy)phenyl]methyl]-4-(phenylsulfamoyl)benzamide (72 mg, 0.14 mmol, 50% yield) as a colorless solid. ^1^H NMR (500 MHz, CDCl_3_): δ 7.80–7.68 (m, 2H), 7.49–7.43 (m, 2H), 7.28–7.19 (m, 3H), 7.17–7.09 (m, 1H), 7.08–6.97 (m, 3H), 6.92–6.83 (m, 2H), 6.63 (s, 1H), 4.83 (s, 1.1H), 4.44 (s, 1H), 4.11–4.04 (m, 2H), 3.98–3.94 (m, 2H), 3.38 (d, *J* = 6.2 Hz, 0.9H), 2.90 (d, *J* = 6.6 Hz, 1.2H), 1.12–0.99 (m, 0.5H), 0.86–0.72 (m, 0.6H), 0.56–0.42 (m, 2H), 0.26–0.18 (m, 0.9H), −0.05 – −0.14 (m, 1H). OH not observed. Rotamers observed in approximately 1:1 ratio. ACQUITY UPLC BEH C18 1.7 μm: *R*_t_ = 1.65 min; *m*/*z* 481.2 [M + H]^+^.

### In Silico Property Predictions

All physicochemical property calculations (*c* log *D*, TPSA) were performed using MarvinSketch v23.2, Chemaxon (https://www.chemaxon.com).

### Crystallography

LIMK1 protein was expressed for crystallization from a pFastBac-derived plasmid containing DNA for residues 330–637 of human LIMK1 isoform 1 (NCBI reference NP_002305) fused to a tobacco etch virus (TEV) protease cleavable hexahistidine tag (extension MHHHHHHSSGVDLGTENLYFQ*SM where * represents the position of digestion by TEV protease). The plasmid was transformed into DH10Bac vector and was expressed in insect cells. One liter of *Spodoptera frugiperda* (sf9) cells (2 million cells/mL) was infected with 15 mL LIMK1 P2 virus.

For purification, the cell pellet was resuspended and lysed by sonication in 100 mL buffer containing 50 mM HEPES 7.5, 500 mM NaCl, 20 mM Imidazole, 0.5 mM TCEP, 5% glycerol and protease inhibitor cocktail tablet (Sigma). The cell lysate was then centrifuged at 35,000*g* for 1 h at 4 °C in the presence of 0.15% PEI. The supernatant was incubated with nickel beads for 1 h at 4 °C. The beads were then washed 4 times with 50 bed volumes of wash buffer (50 mM HEPES, pH 7.5, 500 mM NaCl, 40 mM imidazole and 5% Glycerol) and eluted with 50 mM HEPES, pH 7.5, 500 mM NaCl and 250 mM Imidazole and 5% glycerol. The eluate was incubated at 4 °C overnight with TEV protease to remove the purification tag while being dialyzed against GF Buffer (50 mM HEPES pH 7.5, 150 mM NaCl, 5% glycerol, 0.5 mM TCEP). After dialysis the sample was passed through a column of nickel beads (2.5 mL) and the flow-through and washes with buffer collected. The sample was concentrated to <5 mL and injected on an S200 16/600 gel filtration column (GE Healthcare) pre-equilibrated into GF Buffer. Fractions containing LIMK1 were concentrated to 10 mg/mL, and stored at −80 °C.

For crystallization, concentrated LIMK1 protein was incubated with inhibitors at 1.2 mM concentration (from 50 mM inhibitor stock solutions in 100% DMSO) on ice before setting up crystallization plates. Crystals were obtained using the sitting drop vapor diffusion method at 4 °C from total drop volumes of 200 nL and ratios of protein to well solution of 2:1, 1:1 or 1:2, equilibrated against 25 μL of a reservoir solution 20% PEG3350, 0.2 M ammonium citrate. Crystals appeared after 4 days and were cryo-protected by addition of 25% ethylene glycol and flash frozen in liquid nitrogen. Data was collected at 100 K at the Diamond Synchrotron. Data collection statistics can be found in Table S9. The diffraction data was indexed and integrated using XDS,^44^ scaled using AIMLESS^45^ and structures solved by molecular replacement using PHASER^46^ with a previous structure of human LIMK1 as a search model. The model was built using using Coot^47^ and refined using REFMAC5.^48^

### Production of LIMK1 Homology Model

A homology model of LIMK1 bound to TH-300 (**7**) was derived from the homologous kinase LIMK2, generated from protein structure 5NXD using Schrodinger 2018-3 homology modeling tool (Schrodinger; Inc., New York NY 10036) using physics-based methods, with loop refinement and postmodeling minimization using the standard settings. Sequence alignment between LIMK1 and LIMK2 was compared using Uniprot P53667 and P53671, respectively.

### RapidFire Mass Spectrometry Kinase Assays

Inhibition of cofilin phosphorylation by LIMK1, LIMK2, PAK1-phosphorylated LIMK1 or PAK1-phosphorylated LIMK2 was assessed by kinase enzymatic assay with inhibitors added in dose–response and simultaneous quantification of cofilin and phospho-cofilin performed on a RapidFire-Quadrupole-Time-of-Flight LC–MS instrument (Agilent) as previously described.^14^ The resulting data were analyzed using RapidFire integrator software (Agilent), and GraphPad Prism 7 was used to calculate IC_50_ values. For nonphosphorylated IC_50_ measurements, LIMK enzyme assay concentrations of 40 nM and 15 nM means IC_50_ < 20 nM and IC_50_ < 7.5 nM for LIMK1 and LIMK2, respectively, should be treated with caution.

### Cell Lines and Growth Conditions

HEK293 and SH-SY5Y cells (Sigma/Merck, Dorset, U.K.) were cultured in Dulbecco’s modified Eagle’s medium (DMEM)/F12 (#11320033, Thermo Fisher Scientific, U.K.) supplemented with 10% fetal calf serum, 1% penicillin and 1% streptomycin (all sourced from Sigma-Aldrich, Dorset, U.K.). Cells were cultured in a standard T75 tissue-culture treated flask under standard conditions (37 °C, 5% CO_2_) in a sterile incubator.

### Transient Transfection of HEK293 Cells

The transfection reagent mix was prepared and composed of 1.25 mL of assay media (Opti-MEM without phenol red, Fisher Life Technologies, U.K.), 1.25 μg of NanoLuc LIMK1 or LIMK2 kinase fusion vector (Promega, Hampshire, U.K.), 11.25 μg of transfection carrier DNA (Promega, Hampshire, U.K.), and 37.5 μL of FuGENE HD transfection reagent (Promega, Hampshire, U.K.). Following routine trypsinization, neutralization, and sedimentation, HEK293 cells were resuspended in 5 mL growth media. Cell density was then calculated and adjusted to 1 × 10^5^ cells/mL for each transfection (LIMK1/2) in of 25 mL of growth media. The transfection mix was added directly to the cells and mixed gently via inversion. The HEK293 cells-transfection mix solution was then plated into T75 tissue culture flasks and incubated for 20 h.

### Cellular NanoBRET LIMK1/2 Assay

The NanoBRET cellular target engagement assay was performed as previously described.^14^ Briefly, white 96-well plates containing LIMK1/2 transfected HEK293 cells and extracellular NanoLuc inhibitor was added with either positive control (NanoBRET Tracer #10), negative control (DMSO) or test compound (8-point dose–response curve in DMSO in duplicate, final concentration of 0.5% for control wells). Following incubation of plates under standard conditions (37 °C and 5% CO_2_) for 2 h, plates were removed and allowed to reach RT for 15 min. Freshly prepared Nano-Glo substrate was then added to each well and luminescence measured using dual emission for the donor at 450 nm and the acceptor 610 nm on a BMG Pherastar plate reader. Kit components were purchased from Promega (Hampshire, UK).

### AlphaLISA SureFire Assay

The AlphaLISA assay for detection of p-cofilin Ser3 levels was followed as previously described.^14^ Briefly, 96-well plates containing SH-SY5Y cells was added either positive control (LIMKi3, 10 μM), negative control (0.5% DMSO) or test compound (8-point, 3-fold serial dilution in DMSO from 10 μM to 3 nM in duplicate). Cells were placed in the incubator for 2 h, after which the media was removed and the cells were lysed using 50 μL AlphaLISA 1× lysis buffer (PerkinElmer, USA) containing protease inhibitor cocktail (Sigma/Merck, Dorset, UK) and Pierce phosphatase inhibitor cocktail (Thermo Fisher Scientific, UK). The cell lysate was then transferred to a clean, flat-bottom, white 384-well plate, to which 5 μL/well of acceptor bead solution consisting of Reaction buffer 1, Reaction buffer 2, Activation Buffer and Acceptor Beads from pCofilin SureFire Ultra assay kit (PerkinElmer, USA, cat# ALSU-PCOF-A500) was added under dim light. After incubation at RT for 1 h, 5 μL/well of donor solution consisting of Dilution Buffer and Donor beads was added. The plate was read on a Pherastar reader (BMD Labtech Ltd., Aylesbury, UK) using an AlphaLISA cartridge and AlphaLISA plate settings. The AlphaLISA assay was robust and reproducible (*Z*′ = 0.7).

### Microsomal Stability

Five μL microsomes (20 mg/mL, Corning BV) diluted in 95 μL PBS (pH 7.4 with 0.6% MeCN) containing 0.04% DMSO and 4 μM compound were incubated with 100 μL of prewarmed 4 mM of NADPH in PBS (final concentrations: 0.5 mg/mL microsomes, 2 μM compound, 0.02% DMSO, 0.3% MeCN, and 2 mM NADPH). After mixing thoroughly, the *T* = 0 sample (40 μL) was immediately quenched into an 80 μL ice-cold MeOH containing a 4 μM internal standard (carbamazepine). Three further samples were quenched in the same way at *T* = 3, 9, and 30 min. Samples were incubated on ice for 30 min before centrifugation at 4700 rpm for 20 min. The supernatant was analyzed via LCMS/MS, and compound/carbamazepine peak area ratios were calculated to determine the rate of substrate depletion.

### Thermodynamic Solubility

1–2 mg of accurately weighed compound was suspended in 1 mL PBS (pH 7.0) and incubated (rotating end over end) at room temperature for 24 h. The samples were then centrifuged at >10,000 rpm for 10 min to pellet any remaining solid. The supernatant was then diluted sequentially (1:5, 1:50, 1:500, and 1:5000) in acetonitrile and mixed 1:1 with MeCN containing 4 μM carbamazepine. To prepare the standard, an 8-point, 1:3 dilution curve was prepared in DMSO with a top concentration of 1 mM, which was then diluted to 1:100 in MeCN containing 2 μM carbamazepine. Standards and samples were analyzed via LCMS/MS. The compound carbamazepine peak area ratios were calculated, and the test article solubility was determined by interpolation from the standard curve.

### In Vivo DMPK and CNS Penetration Studies

i.v., p.o. and i.p. PK data were generated at Pharmidex (Hatfield, U.K.), Sygnature Discovery (Nottingham, U.K.) or internally (vide infra).

For Pharmidex and Sygnature Discovery studies, Male Sprague–Dawley rats were either administered: (1) intravenously (i.v.) dosed at 1 mg/kg with a single test compound in a formulation of 10% DMSO/20% Cremophor/70% saline (**51**, **74**, **75**), (2) i.v. dosed at 0.2 mg/kg with five test compounds as a cassette in a formulation of 10% DMSO/20% Kolliphor EL/70% (0.9%) saline (**55**, **85**), or (3) oral gavage (p.o.) dosed at 3 mg/kg with a single test compound in a formulation of 17% solutol/18% glycerol/65% citric buffer (pH 3, **74**). Plasma samples were taken at 2, 5, 15, 30 min, 1, 2, 4, 8, and 24 h periods for bioanalysis. A satellite group of three animals were also administered i.v. test compound (dosed at 1 mg/kg in 10% DMSO/20% Cremophor/70% saline) and after 1 h, the animals culled and brain samples immediately prepared by homogenization in H_2_O and protein precipitation in MeCN. Bioanalysis on plasma and brain samples were performed using a UHPLC-tandem mass spectrometry using electrospray ionization.

For internal studies, Male Sprague–Dawley rats were purchased from Charles River UK. All compounds were prepared in a vehicle containing 20% (w/v) of 2-hydroxypropyl-β-cyclodextrin (Sigma/Merck) in dH_2_O and compound concentrations adjusted accordingly to be dosed at an equal volume/bodyweight ratio (5 mL/kg) into animals. Compounds were either dosed via p.o. or i.p. as stated individually. At 1 h, animals were culled and plasma and brain samples collected immediately. Analyte standard curve samples (matrix matched in citrated rat plasma) and collected plasma samples were precipitated in MeOH containing internal standard (I.S.—Carbemazapine). The supernatant was subjected to HybridSPE filtration to remove lipids, followed by vacuum evaporation. The dried samples were resuspended in 50% MeCN and analyzed via LC/MS–MS. Brain samples were homogenized in 10 mM potassium phosphate buffer (pH 7.0) using an IKA T 10 basic ULTRA-TURRAX disperser (VWR, cat # 431-0188) fitted with a S12N-12S dispersing element (VWR, cat # 431-0112) using Fisherbrand prefilled bead mill tubes (cat: 15,555,799) and protein precipitation procedure in MeOH followed as described above. Analyte peak areas were normalized to I.S. and concentrations interpolated from the matrix matched standard curve.

### Tolerability Study

Non-GLP 28 day intraperitoneal toxicology study with 2 weeks recovery in mice was performed at Charles River Laboratories (Veszprém, Hungary) test facility. **85** was freshly formulated at 12 mg/mL in vehicle containing 40% (v/v) of propylene glycol (Thermo Fisher) in dH_2_O on the day of administration. Twenty male mice (five mouse/group) were treated via intraperitoneal injection (i.p.) at a dose volume of 2.5 mL/kg. A test group of five mice was injected with **85** formulation (at 30 mg/kg dose) and a control group of five mice was injected with the corresponding vehicle control. The main animals received a single daily dose of **85** for 29 days, the recovery animals received a single daily dose of **85** for 28 days with 14 days recovery period. The day of dosing of each animal was regarded as Day 1. Animals were inspected for signs of morbidity and mortality once daily and clinical observations were recorded at the time seen (note: one animal from the control group was euthanized for humane reasons on Day 14). Body weights and food consumption were recorded on Day 1 and twice weekly from the day of dosing.

At the end of the treatment period, prior to scheduled euthanasia by sodium pentobarbital (Euthanimal 40%) terminal anesthesia and necropsy on Day 29 (all main animals), blood samples were collected from retro orbital plexus (0.5 mL in tubes with sodium citrate as anticoagulant) for clinical chemistry. Immediately after collection, samples were gently inverted several times to ensure complete mixing with the anticoagulant. Afterward, the samples were kept in an ice-cooled water bath and were centrifuged (within 1 h after collection) at 3000*g* for 10 min at 4 °C. The supernatant plasma were immediately snap frozen on dry ice and stored frozen below −80 ± 10 °C for bioanalysis. Brain samples from all nonrecovery animals were placed in a freezer tube immediately after dissection and were snap frozen in liquid nitrogen. The samples were stored at −80 °C before bioanalysis. Levels of compound in plasma and brain were analyzed according to the method outlined under “In Vivo DMPK and CNS Penetration Studies”.

### Mouse Hippocampal Slice Preparation and Western Blot Analysis

All procedures involving mice were performed in accordance with Schedule 1 of the UK Government Animals (Scientific Procedures) Act 1986 and under the auspices of an approved Home Office project license (PP320488). Hippocampal slices were obtained from neonatal (postnatal days 7–9) wild type (WT) and *Fmr1* KO mice originally supplied by Jackson Laboratories. The *Fmr1* KO mice were generated by breeding homozygous females with hemizygous males (see link https://www.jax.org/strain/004624) and were subsequently genotyped by Transnetyx. Mice for experimentation were killed by cervical dislocation. Following decapitation, the brain was rapidly dissected, with incisions made at the cerebellum and frontal lobes, and then placed in iced cold solution containing artificial cerebrospinal fluid (aCSF) of the following composition (in mM): NaCl, 126; KCl, 2.95; CaCl_2_, 2.5; NaHCO_3_, 26; NaH_2_PO_4_, 1.25; d-glucose, 10; MgSO_4_, 1.3; pH 7.4 with 95% O_2_/5% CO_2_. The temporal aspects of the brain were trimmed, bisected, then glued to a metal plate, such that the midline was uppermost and horizontal. In this orientation, the brain was submerged in oxygenated (95% O_2_/5% CO_2_) aCSF and a Vibratome (IntraCel, Royston, Herts, UK) was then used to cut 400 μm-thick brain slices. Sagittal hippocampal brain slices were cut from each bisected hemisphere, which were then placed on a submerged nylon mesh in an incubation chamber, filled with circulating oxygenated aCSF, at 32 °C, for at least 2 h.

Following a 2 h incubation in circulating aCSF at 32 °C the slices were then perfused for 30 min with either FRAX486 (**2**, 3 μM), SR7826 (**4**, 3 μM), MDI-114215 (**85**, 3 μM), or vehicle solution containing an equivalent concentration of DMSO. Following incubation, the brain slices were snap frozen and stored at −80 °C. Protein was extracted from the mouse tissue using cell extraction buffer (Thermo Fisher FNN0011) containing protease inhibitor (Sigma, S8820) and phosphatase inhibitor (Thermo Fisher, A32957). Each individual sample was suspended in 300 μL of buffer and lysed by pipetting on ice. Samples were centrifuged to pellet at 17,000*g* for 30 min at 4 °C. Protein was quantified using Biorad DC protein assay (Biorad, 5000112) and read on a PheraStar plate reader (BMG Labtech) and standardized to 2 mg/mL each sample.

Sample loading buffer was added to each sample at 1:4 concentrations and boiled for 5 min prior to use. Samples were then loaded onto Novex TRIS glycine 12% gels and assembled in XCell SureLock electrophoresis tanks (Thermo Fisher, EI0001) and topped up with SDS running buffer and run at 140 V, 200 mA and 50 W for 1.5 h for protein separation. Gels were transferred onto PVDF membrane using Electroblot semidry blotting unit (Invitrogen/Thermo Fisher, UK) at 20 V, 500 mA and 50 W for 50 min. Membranes were then blocked in 5% BSA TBST for 1 h at rt and then placed into LIMK1, p-cofilin, cofilin and actin primary antibody solutions at 4 °C overnight. The following day, membranes were washed 3× in TBST solution before being placed in appropriate secondary antibody solutions for 1 h at room temperature. Following a final 3 washes in TBST, the membranes were developed using ClarityMax ECL substrate (Biorad, UK) at a 1:1 ratio. The ECL solution was allowed to develop on the membrane for 5 min prior to visualizing using the CHEMIDOC imaging system (Biorad, UK).

### Ex Vivo Extracellular Recording from Hippocampal CA1 Pyramidal Neurons

Hippocampal slices were obtained from a minimum of four mice of either sex. For recording, a slice was transferred to a submersion recording chamber (Scientific Systems Design, Paris, France) and continuously perfused at 4–6 mL/min with aCSF utilizing a peristaltic pump (Gilson Minipuls Evolution, Paris, France). The perfusion medium was gassed with 95% O_2_/5% CO_2_ and maintained at 32 °C. For the accurate positioning by micromanipulators of the stimulating and recording electrodes, the hippocampal slice was viewed via a microscope (Olympus SZ30). To induce and monitor basal synaptic transmission, a concentric bipolar stimulating electrode (125 μm conical tip; inner pole 25 μm) was positioned in *Stratum radiatum*, allowing the afferent *Schaffer collateral*—commissural pathway from the CA3 area to the CA1 region to be stimulated. A Digitimer stimulator was utilized to excite the pathway at 30 s intervals (0.033 Hz, 100 μS duration). The glass (King Precision Glass; ID: 1.00 ± 0.05; OD: 1.55 ± 0.05) extracellular recording microelectrode was filled with aCSF (>5 MΩ) and positioned in the hippocampal *Stratum radiatum* of area CA1. The microelectrode was carefully lowered into the dendritic region of CA1 until clear field excitatory postsynaptic potentials (fEPSPs) were observed. Such fEPSPs were simultaneously displayed on a digital storage oscilloscope (Tektronix 2201) and via an A to D converter (National Instruments, Paris, France; BNC-2090) on a computer screen. The slope of each fEPSP (mV ms^–1^) was calculated online and the stimulus adjusted to produce a response 40% of the maximum. For long-term potentiation (LTP) experiments, maximal LTP was induced by a 4-pulse theta-burst stimulation (4-TBS) protocol (4 pulses, delivered at 100 Hz, repeated 10 times, each at an interval of 200 ms, i.e. total duration of 2 s). The stimulus parameters, the acquisition and the analysis of fEPSPs were under the control of LTP software (courtesy of Dr Anderson and Professor Collingridge, Bristol University, UK; http://www.ltp-program.com). The electrical signals were acquired at 10 kHz and filtered at 10 Hz to 3 kHz. Statistical analysis of LTP was performed using the IBM SPSS v29 statistical software and a comparison of the change in the fEPSP slope following the 4-TBS was determined by means of an independent *t*-test at 50–60 min post the high frequency stimulation.

**4** and **85** were prepared as stock solutions of 10 mM in 100% DMSO and subsequently diluted into the perfusion buffered saline to achieve the desired final bath concentration (3 μM). For all studies, stable baseline recordings of the field excitatory postsynaptic potentials (fEPSPs) were elicited by electrical stimulation (0.033 Hz) of the hippocampal *Schaffer collateral* pathway and monitored online as the slope of the fEPSP. Such stable fEPSPs were obtained for a minimum of 20 min prior to bath perfusion of the LIMK inhibitor. To investigate whether the LIMK inhibitors influenced LTP, they were applied for a further 30 min, prior to delivering the 4-TBS. Note neither drug influenced the control fEPSP during this 30 min application. Following the 4-TBS the drug was continually perfused throughout the remainder of the LTP experiment (a further 60 min). The fEPSPs (pre and post the 4-TBS protocol) were recorded in the continued presence of **4** and **85** and the magnitude of LTP determined between 50 and 60 min post the 4-TBS.

### Generation of Human iPSC-Derived Cortical Neuron

Method was adapted from our recent publication.^49^ Human cortical neurons were obtained from iPSCs culture. Briefly, iPSCs were cultivated in 10 cm dishes precoated with Matrigel (Corning) and were maintained in mTeSR1 media (STEMCELL Technologies) that was changed daily. Cortical progenitors (NPCs) were obtained from iPSCs as previously described^50^ and then banked. NPCs were grown in flasks precoated with poly-l-ornithine (PO) and laminin in NPC progenitor media composed by DMEM-F12 supplemented with N2, B27, NEAA, Antibiotic-Antimycotic, laminin (1 μg/mL), EGF (20 ng/mL) and FGFb (20 ng/mL). For cortical neuronal differentiation, cells were cultivated in PO and laminin precoated flasks and neuronal differentiation composed by Neurobasal media (Gibco) supplemented with N_2_, B27, compound E (0.1 μM), db-cAMP (500 μM), ascorbic acid (200 μM), BDNF (20 ng/mL), GDNF (20 ng/mL), TGF-β3 (1 ng/mL), laminin (1 μg/mL). Cells were kept in neuronal differentiation media until analysis, and half of the medium was changed twice a week.

### Statistical Analysis

A one-way ANOVA (with Brown–Forsythe test) with Dunnett’s multiple comparisons test was used to compare the effect of LIMK inhibitors on the p-cofilin/cofilin ratio in WT and *Fmr1* KO hippocampal brain slices. Details about the multiple comparisons test can be found in Table S10. Data analyses were performed using GraphPad Prism version 10.3.1.

## Supporting Information Available

Molecular formula strings containing SMILES string and associated biochemical and biological data (CSV)
Supporting Information figures and tables, including DiscoverX scanMAX panel CEREP selectivity panel data for compound **85**, Western blots of ex vivo treated brain slices and statistical data, data collection and refinement statistics for PDB: 7B8W; ^1^H, ^13^C, ^19^F NMR and UPLC-MS characterization data and details of chemical synthesis of all final compounds (PDF)

## Notes

PDB ID of New Crystal (X-ray) Structures/Homology Models: Authors will release the atomic coordinates upon article publication.

## Notes

Information on the data underpinning this publication, including access details, can be found in the Cardiff University Research Data Repository.

## Abbreviations

ADF: actin-depolymerizing factor
ASD: autism spectrum disorder
BMPR2: bone morphogenetic protein receptor type 2
CaMKIV: calcium/calmodulin-dependent protein kinase IV
FMRP: fragile X mental retardation protein
FXS: fragile X syndrome
LIMK: LIM domain kinase
MRCKα: myotonic dystrophy kinase-related Cdc42-binding kinase alpha
PAK: p21-activated kinase
ROCK: Rho-associated protein kinase
TESK: testis specific protein kinase
TKL: tyrosine kinase-like kinase
