# Supplementary material for: Discovery of MDI-114215: A Potent and Selective LIMK Inhibitor To Treat Fragile X Syndrome
Source: J Med Chem. 2024 Dec 23;68(1):719–52. doi: 10.1021/acs.jmedchem.4c02694 (PMC11726654; doi:10.1021/acs.jmedchem.4c02694)

Supporting Information

Discovery of MDI-114215: A potent and selective LIMK inhibitor to treat Fragile X syndrome

Alex G. Baldwin[1], David W. Foley[1†], Ross Collins[1‡], Hyunah Lee[2§], D. Heulyn Jones[1], Ben Wahab[1], Loren Waters[1], Josephine Pedder[1∥], Marie Paine[1], Gui Jie Feng[1⊥], Lucia Privitera[3⫛], Alexander Ashall-Kelly[1], Carys Thomas[1], Jason A. Gillespie[1‡], Lauramariú Schino[1], Delia Belelli[3], Cecilia Rocha[4], Gilles Maussion[4], Andrea I. Krahn[4], Thomas M. Durcan[4], Jonathan M. Elkins[2], Jeremy J. Lambert[3], John R. Atack[1], Simon E. Ward[1]*

[1]Medicines Discovery Institute, School of Biosciences, Cardiff University, Main Building, Park Place, Cardiff, CF10 3AT, United Kingdom. [2]Centre for Medicines Discovery, University of Oxford, Roosevelt Drive, Oxford, OX3 7DQ, United Kingdom. [3]Division of Neuroscience, School of Medicine, Medical Sciences Institute, Dundee University, Dow Street, Dundee, DD1 5HL, United Kingdom. [4]The Neuro's Early Drug Discovery Unit (EDDU), Department of Neurology and Neurosurgery, Montreal Neurological Institute-Hospital, McGill University, 3801 University Street, Montreal, Quebec, H3A 2B4, Canada.

Corresponding Author email address: WardS10@cardiff.ac.uk

Contents

Supplementary Figures and Tables 5

Table S1 | LIMK1/2 inhibitory activities of TH-257 (**6**) and amide analogues **9** and **10** 5

Table S2 | LIMK1/2 inhibitory activities of sulfonamide replacements **15**, **17** and **29** 6

Table S3 | LIMK1/2 inhibitory activities of *N*-phenylsulfonamide **34**-**38** 7

Table S4 | LIMK1/2 inhibitory activities of core phenyl analogues **19**-**24** and **30** 8

Table S5 | LIMK1/2 inhibitory activities of *N*-benzyl replacements **39**-**50** 9

Table S6 | LIMK1/2 inhibitory activities of *N*-butyl replacements and substituted benzyl analogues **51**-**56** and **58**-**65** 10

Figure S1 | Plot of microsomal CL_{int} (µL/min/mg) against cLogD for the TH-257 (**6**) and methyl cyclopropyl (**57**) series 11

Figure S2 | Dose escalation and linearity study of MDI-114215 (**85**) in CD-1 male mice after IP administration at 10, 30 and 50 mg/kg in 40% propylene glycol in dH_2O 12

Table S7 | KINOMEscan™ of MDI-114215 (**85**) tested against 468 kinases (Eurofins/DiscoverX scanMAX panel) at 300 nM 13

Table S8 | Selectivity profile of MDI-114215 (**85**) in the CEREP selectivity panel (Eurofins/CEREP, France) 24

Figure S3 | Full-size Western blots of treated brain slices isolated from young WT or *Fmr1* KO (P7-9) upon incubation with 3 µM of control (DMSO, P7 WT), FRAX486 (**2**) or MDI-114215 (**85**). 25

Figure S4 | Full-size Western blots of treated brain slices isolated from young WT or *Fmr1* KO (P7-9) upon incubation with 3 µM of control (DMSO, P7 WT) or SR7826 (**4**)....................................... 26

Table S9 | Data collection and refinement statistics for LIMK1 co-crystal structure with TH-470 (**8**) 27

Table S10 | Statistical data used to compare effect of LIMK inhibitors on WT or *Fmr1* KO hippocampal brain slices... 28

Description of rotamers for final compound characterisation 29

Figure S5 | Stacked VT-NMR spectra of TH-257 (**6**) aromatic region in DMSO-d_6 29

^{1}H, ^{13}C, ^{19}F NMR and UPLC data for final compounds 30

N-Butyl-*N*-(4-(*N*-phenylsulfamoyl)benzyl)benzamide (**10**) 32

N-Benzyl-*N*-butyl-4-(phenylsulfonamido)benzamide (**15**).. 34

N[1]-Benzyl-*N*[1]-butyl-*N*[4]-phenylterephthalamide (**17**) 35

N-Benzyl-*N*-butyl-3-fluoro-4-(phenylsulfamoyl)benzamide (**19**) 36

N-Benzyl-*N*-butyl-3-chloro-4-(phenylsulfamoyl)benzamide (**20**) 38

N-Benzyl-*N*-butyl-2-fluoro-4-(phenylsulfamoyl)benzamide (**21**) 39

N-Benzyl-*N*-butyl-2-methyl-4-(phenylsulfamoyl)benzamide (**22**)... 41

N-Benzyl-*N*-butyl-5-(phenylsulfamoyl)pyridine-2-carboxamide (**23**) 42

N-Benzyl-*N*-butyl-2-(phenylsulfamoyl)pyrimidine-5-carboxamide (**24**) 43

N-Benzyl-*N*-butyl-5-(phenylsulfamoyl)furan-3-carboxamide (**30**) 45

N-Benzyl-*N*-butyl-4-(*N*-methylsulfamoyl)benzamide (**34**).. 46

N-Benzyl-*N*-butyl-4-(*N*-(pyridin-4-yl)sulfamoyl)benzamide (**35**) 47

N-Benzyl-*N*-butyl-4-(isoxazol-4-ylsulfamoyl)benzamide (**36**).. 48

N-Benzyl-*N*-butyl-4-(*N*-cyclobutylsulfamoyl)benzamide (**37**) 49

N-Benzyl-*N*-butyl-4-(*N*-(oxetan-3-yl)sulfamoyl)benzamide (**38**).. 50

N-Butyl-*N*-(4-fluorobenzyl)-4-(*N*-phenylsulfamoyl)benzamide (**39**).. 51

N-Butyl-*N*-(4-methoxybenzyl)-4-(*N*-phenylsulfamoyl)benzamide (**40**)...................................... 53

N-Butyl-*N*-(3-methoxybenzyl)-4-(*N*-phenylsulfamoyl)benzamide (**41**)...................................... 55

N-Butyl-*N*-(2-methoxybenzyl)-4-(*N*-phenylsulfamoyl)benzamide (**42**)...................................... 57

N-Butyl-4-(*N*-phenylsulfamoyl)-*N*-(pyridin-4-ylmethyl)benzamide (**43**) 59

N-Butyl-4-(*N*-phenylsulfamoyl)-*N*-(pyridin-3-ylmethyl)benzamide (**44**) 61

N-Butyl-4-(*N*-phenylsulfamoyl)-*N*-(pyridin-2-ylmethyl)benzamide (**45**) 63

N-Butyl-*N*-(furan-2-ylmethyl)-4-(*N*-phenylsulfamoyl)benzamide (**46**) 65

N-Butyl-4-(*N*-phenylsulfamoyl)-*N*-((tetrahydrofuran-2-yl)methyl)benzamide (**47**)...................... 67

3-(4-((*N*-Butyl-4-(*N*-phenylsulfamoyl)benzamido)methyl)phenyl)propanoic acid (**48**)................. 69

N-Butyl-4-(*N*-phenylsulfamoyl)-*N*-(4-(piperazin-1-yl)benzyl)benzamide (**49**) 70
N-[[4-(4-Benzylpiperazin-1-yl)phenyl]methyl]-*N*-butyl-4-(phenylsulfamoyl)benzamide (**50**) 71
N-Butyl-*N*-(1*H*-indol-5-ylmethyl)-4-(phenylsulfamoyl)benzamide (**51**) 72
N-Benzyl-*N*-ethyl-4-(*N*-phenylsulfamoyl)benzamide (**52**) 73
N-Benzyl-*N*-(2-hydroxyethyl)-4-(*N*-phenylsulfamoyl)benzamide (**53**)....................................... 74
N-Benzyl-*N*-(2-cyanoethyl)-4-(*N*-phenylsulfamoyl)benzamide (**54**)... 76
N-(2-Cyanoethyl)-4-(phenylsulfamoyl)-*N*-(4-pyridylmethyl)benzamide (**55**) 77
N-Benzyl-*N*-(3-imidazol-1-ylpropyl)-4-(phenylsulfamoyl)benzamide (**56**) 78
N-Benzyl-*N*-(cyclopropylmethyl)-4-(phenylsulfamoyl)benzamide (**57**) 79
N-Benzyl-*N*-cyclopropyl-4-(phenylsulfamoyl)benzamide (**58**) 80
N-Benzyl-*N*-isobutyl-4-(phenylsulfamoyl)benzamide (**59**) 81
N-Benzyl-*N*-[[1-(imidazol-1-ylmethyl)cyclopropyl]methyl]-4-(phenylsulfamoyl)benzamide (**60**)... 82
N-(Cyclopropylmethyl)-*N*-(1*H*-indol-5-ylmethyl)-4-(phenylsulfamoyl)benzamide (**61**) 83
N-(1,3-Benzoxazol-6-ylmethyl)-*N*-(cyclopropylmethyl)-4-(phenylsulfamoyl)benzamide (**62**) 84
N-[(5-Bromo-2-pyridyl)methyl]-*N*-(cyclopropylmethyl)-4-(phenylsulfamoyl)benzamide (**63**) 85
(*S*)-*N*-(Cyclopropylmethyl)-*N*-(1-phenylethyl)-4-(*N*-phenylsulfamoyl)benzamide (**64**) 86
(*R*)-*N*-(Cyclopropylmethyl)-*N*-(1-phenylethyl)-4-(*N*-phenylsulfamoyl)benzamide (**65**)................ 87
N-(Cyclopropylmethyl)-*N*-((5-fluoropyridin-2-yl)methyl)-4-(*N*-phenylsulfamoyl)benzamide (**71**).. 88
N-[(5-Amino-2-pyridyl)methyl]-*N*-(cyclopropylmethyl)-4-(phenylsulfamoyl)benzamide (**72**) 89
N-(Cyclopropylmethyl)-*N*-[(5-morpholino-2-pyridyl)methyl]-4-(phenylsulfamoyl)benzamide (**73**) 90
N-(Cyclopropylmethyl)-*N*-[[5-(2-hydroxyethylamino)-2-pyridyl]methyl]-4-(phenylsulfamoyl)benzamide (**74**)... 91
N-(Cyclopropylmethyl)-*N*-[[5-(2-methoxyethylamino)-2-pyridyl]methyl]-4-(phenylsulfamoyl)benzamide (**75**)... 92
N-(Cyclopropylmethyl)-*N*-[[5-(oxetan-3-ylamino)-2-pyridyl]methyl]-4-(phenylsulfamoyl)benzamide (**76**) 93
N-(Cyclopropylmethyl)-*N*-[[5-[2-hydroxyethyl(methyl)amino]-2-pyridyl]methyl]-4-(phenylsulfamoyl)benzamide (**77**)... 94
N-(Cyclopropylmethyl)-*N*-[[5-[2-(dimethylamino)ethylamino]-2-pyridyl]methyl]-4-(phenylsulfamoyl)benzamide (**78**)... 95
N-(Cyclopropylmethyl)-4-(phenylsulfamoyl)-*N*-[[5-[2-(1*H*-tetrazol-5-yl)ethyl]-2-pyridyl]methyl]benzamide (**79**)... 96
N-Ethyl-*N*-[[5-(2-hydroxyethylamino)-2-pyridyl]methyl]-4-(phenylsulfamoyl)benzamide (**80**) 97
N-(Cyclopropylmethyl)-*N*-[[5-[(2-hydroxy-1,1-dimethyl-ethyl)amino]-2-pyridyl]methyl]-4-(phenylsulfamoyl)benzamide (**81**)... 98
N-(Cyclopropylmethyl)-*N*-[[5-(2-hydroxyethylamino)-6-methyl-2-pyridyl]methyl]-4-(phenylsulfamoyl)benzamide (**82**)... 99

N-(Cyclopropylmethyl)-*N*-[[5-(2-hydroxyethylamino)-4-methyl-2-pyridyl]methyl]-4-(phenylsulfamoyl)benzamide (**83**) 100

N-(Cyclopropylmethyl)-*N*-[[5-(2-hydroxyethylamino)-2-pyridyl]methyl]-4-[methyl(phenyl)sulfamoyl]benzamide (**84**) 101

N-(Cyclopropylmethyl)-*N*-[[4-(2-hydroxyethylamino)phenyl]methyl]-4-(phenylsulfamoyl)benzamide (MDI-114215, **85**) 102

N-(Cyclopropylmethyl)-*N*-[[4-(2-hydroxyethoxy)phenyl]methyl]-4-(phenylsulfamoyl)benzamide (**86**) 103

Supplementary Figures and Tables

Compound	Structure	RapidFire pIC_{50}	
		LIMK1	LIMK2
**6** (TH-257)	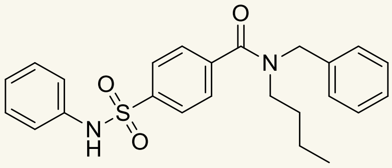	6.58 ± 0.11	7.82 ± 0.03
**9**	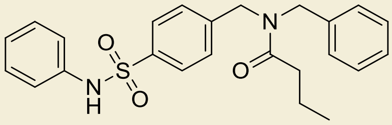	< 5	< 5
**10**	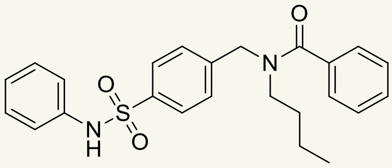	< 5	< 5

Table S1 | LIMK1/2 inhibitory activities of TH-257 (**6**) and amide analogues **9** and **10**. Data are reported as mean ± SEM of at least 3 independent experiments.

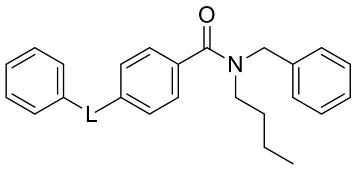

Compound	L	RapidFire pIC_{50}	
		LIMK1	LIMK2
**6** (TH-257)	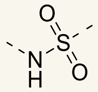	6.58 ± 0.11	7.82 ± 0.03
**15**	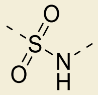	5.53 ± 0.18	6.55 ± 0.07
**17**	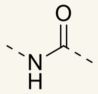	< 5	< 5
**29**	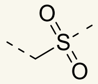	< 5	6.33 ± 0.00

Table S2 | LIMK1/2 inhibitory activities of sulfonamide replacements **15**, **17** and **29**. Data are reported as mean ± SEM of at least 3 independent experiments.

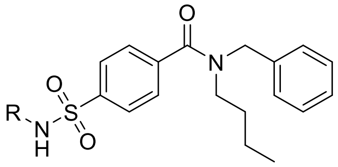

Compound	R	RapidFire pIC_{50}	
		LIMK1	LIMK2
**6** (TH-257)	Ph	6.58 ± 0.11	7.82 ± 0.03
**34**	Me	< 5	< 5
**35**	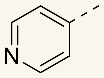	< 5	< 5
**36**	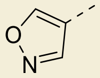	< 5	5.18 ± 0.15
**37**	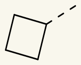	5.35 ± 0.16	6.43 ± 0.21
**38**	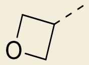	< 5	< 5

Table S3 | LIMK1/2 inhibitory activities of *N*-phenylsulfonamide **34-38**. Data are reported as mean ± SEM of at least 3 independent experiments.

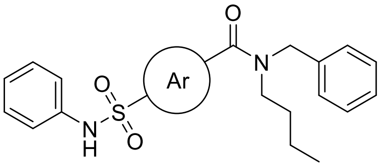

Compound	Ar	RapidFire pIC_{50}	
		LIMK1	LIMK2
**6** (TH-257)	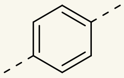	6.58 ± 0.11	7.82 ± 0.03
**19**	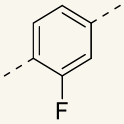	6.47 ± 0.08	7.59 ± 0.14
**20**	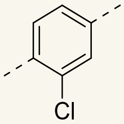	6.51 ± 0.18	7.42 ± 0.06
**21**	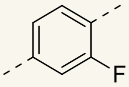	6.39 ± 0.07	7.52 ± 0.15
**22**	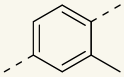	5.67 ± 0.16	6.83 ± 0.15
**23**	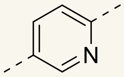	5.87 ± 0.11	6.78 ± 0.14
**24**	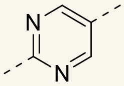	6.05 ± 0.04	5.76 ± 0.19
**30**	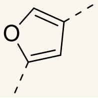	5.16 ± 0.05	6.03 ± 0.20

Table S4 | LIMK1/2 inhibitory activities of core phenyl analogues **19**-**24** and **30**. Data are reported as mean ± SEM of at least 3 independent experiments.

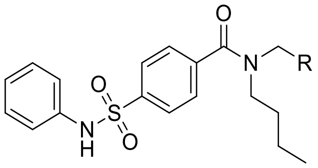

Compound	R	cLogD	RapidFire pIC_{50}		Microsomal CL_{int} (µL/min/mg)	
			LIMK1	LIMK2	Human	Rat
**6** (TH-257)	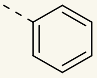	4.63	6.58 ± 0.11	7.82 ± 0.03	439	853
**39**	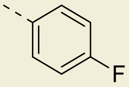	4.78	6.71 ± 0.04	7.57 ± 7.66	409	942
**40**	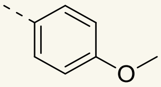	4.48	6.35 ± 0.07	7.40 ± 0.05	280	978
**41**	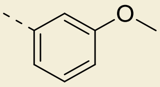	4.48	5.79 ± 0.04	6.65 ± 0.18	556	783
**42**	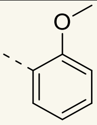	4.48	5.59 ± 0.07	5.93 ± 0.16	812	827
**43**	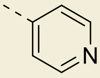	3.42	6.01 ± 0.15	7.72 ± 0.13	144	484
**44**	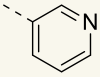	3.42	5.09 ± 0.12	5.82 ± 0.03	234	Rapid
**45**	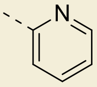	3.50	5.89 ± 0.03	7.29 ± 0.05[a]	256	Rapid
**46**	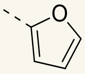	3.69	6.46 ± 0.14	7.66 ± 0.11	Rapid	Rapid
**47**	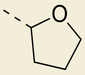	3.33	< 5	< 5	427	Rapid
**48**	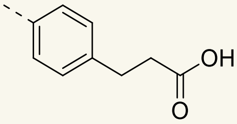	1.83	6.78 ± 0.28	7.68 ± 0.07	15	18
**49**	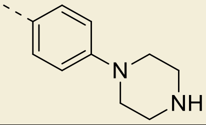	3.09	< 5	5.64 ± 0.06	50	56
**50**	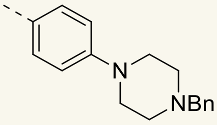	5.59	5.18 ± 0.09	6.10 ± 0.08	48	58

Table S5 | LIMK1/2 inhibitory activities of *N*-benzyl replacements **39**-**50**. Data are reported as mean ± SEM of at least 3 independent experiments. [a]Mean of two independent experiments.

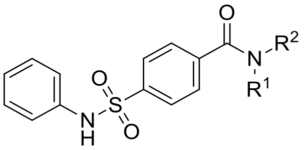

Compound	R[1]	R[2]	cLogD	RapidFire pIC_{50}		Microsomal CL_{int} (µL/min/mg)	
				LIMK1	LIMK2	Human	Rat
**6** (TH-257)	Bu	Bn	4.63	6.58 ± 0.11	7.82 ± 0.03	439	853
**51**	Bu	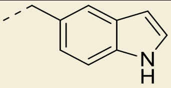	4.73	6.86 ± 0.13	7.68 ± 0.04	121	357
**52**	Et	Bn	3.67	6.66 ± 0.15	7.92 ± 0.04	720	Rapid
**53**	CH_2CH_2OH	Bn	2.62	6.68 ± 0.05[a]	7.30 ± 0.06	76	Rapid
**54**	CH_2CH_2CN	Bn	3.08	6.31 ± 0.10	8.10 ± 0.06	152	Rapid
**55**	CH_2CH_2CN	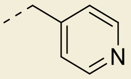	1.86	5.33 ± 0.11	7.07 ± 0.10	32	81
**56**	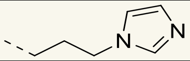	Bn	3.21	6.46 ± 0.14	7.74 ± 0.14	80	158
**58**	cPr	Bn	3.78	5.97 ± 0.08	7.33 ± 0.06	396	Rapid
**59**	$CH_2CH(CH_3)_2$	Bn	4.55	5.99 ± 0.01	7.68 ± 0.09	Rapid	Rapid
**60**	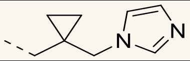	Bn	3.72	6.39 ± 0.08	7.72 ± 0.07	109	395
**61**	CH_2cPr	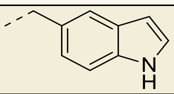	4.19	7.96 ± 0.09	7.96 ± 0.20	288	802
**62**	CH_2cPr	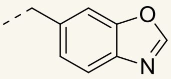	3.44	7.02 ± 0.21	8.19 ± 0.01	263	Rapid
**63**	CH_2cPr	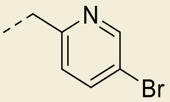	3.72	7.72 ± 0.13	7.59 ± 0.05	307	Rapid
**64**	CH_2cPr	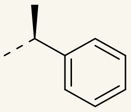	4.51	6.62 ± 0.11	7.00 ± 0.15	774	Rapid
**65**	CH_2cPr	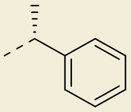	4.51	5.31 ± 0.05	5.97 ± 0.03	419	Rapid

Table S6 | LIMK1/2 inhibitory activities of *N*-butyl replacements and substituted benzyl analogues **51-56** and **58-65**. Data are reported as mean ± SEM of at least 3 independent experiments, unless otherwise stated. [a]Mean of two independent experiments.

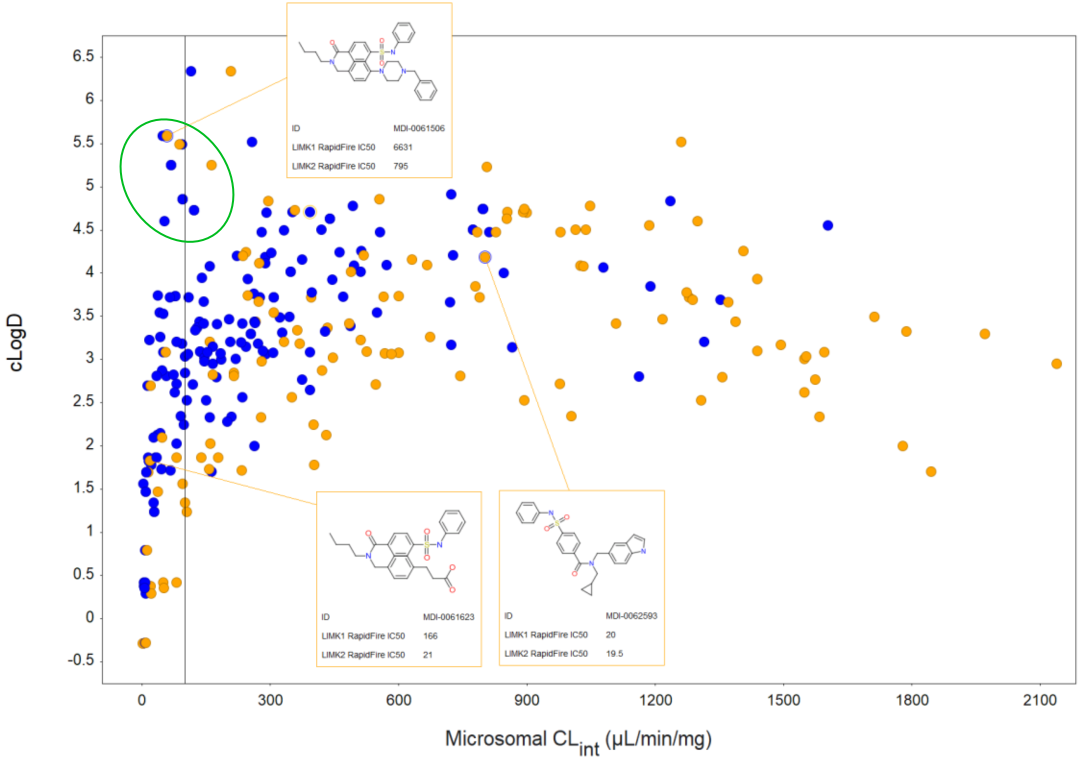

Figure S1 | Plot of microsomal CL_{int} (µL/min/mg) against cLogD for the TH-257 (**6**) and methyl cyclopropyl (**57**) series. CL_{int} data was generated in rat (orange, 117 compounds) and human (blue, 162 compounds). It should be noted that only ionisable compounds with measurable microsomal CL_{int} were included and sixty compounds did not generate a valid CL_{int} value (principally in rat), thus the analysis is likely underestimating the number of rapidly cleared molecules. The desired cutoff for high metabolic stability is shown by the black vertical line (≤ 100 µL/min/mg). cLogD was calculated using ChemAxon. Key molecules are highlighted in inset boxes based on RLM values.

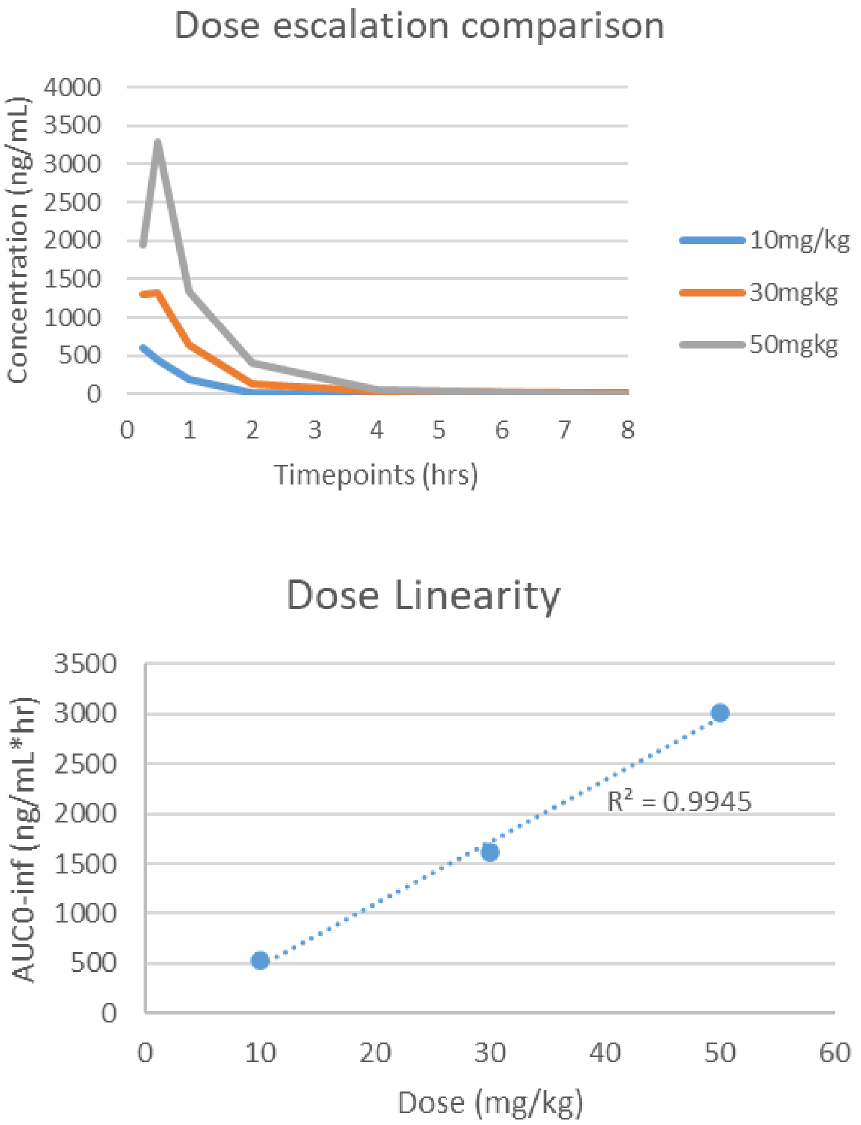

Figure S2 | Dose escalation and linearity study of MDI-114215 (**85**) in CD-1 male mice after IP administration at 10, 30 and 50 mg/kg in 40% propylene glycol in dH_2O. Performed at Pharmidex (Hatfield, U.K.).

Table S7 | KINOMEscan™ of MDI-114215 (**85**) tested against 468 kinases (Eurofins/DiscoverX scanMAX panel) at 300 nM. Kinases with binding interaction < 50% relative to control (% Ctrl) are highlighted.

DiscoveRx Gene Symbol	Entrez Gene Symbol	% Ctrl
AAK1	AAK1	95
ABL1(E255K)-phosphorylated	ABL1	96
ABL1(F317I)-nonphosphorylated	ABL1	93
ABL1(F317I)-phosphorylated	ABL1	91
ABL1(F317L)-nonphosphorylated	ABL1	98
ABL1(F317L)-phosphorylated	ABL1	86
ABL1(H396P)-nonphosphorylated	ABL1	88
ABL1(H396P)-phosphorylated	ABL1	94
ABL1(M351T)-phosphorylated	ABL1	89
ABL1(Q252H)-nonphosphorylated	ABL1	99
ABL1(Q252H)-phosphorylated	ABL1	41
ABL1(T315I)-nonphosphorylated	ABL1	94
ABL1(T315I)-phosphorylated	ABL1	96
ABL1(Y253F)-phosphorylated	ABL1	74
ABL1-nonphosphorylated	ABL1	85
ABL1-phosphorylated	ABL1	98
ABL2	ABL2	95
ACVR1	ACVR1	81
ACVR1B	ACVR1B	90
ACVR2A	ACVR2A	100
ACVR2B	ACVR2B	99
ACVRL1	ACVRL1	86
ADCK3	CABC1	100
ADCK4	ADCK4	96
AKT1	AKT1	73
AKT2	AKT2	90
AKT3	AKT3	91
ALK	ALK	83
ALK(C1156Y)	ALK	96
ALK(L1196M)	ALK	95
AMPK-alpha1	PRKAA1	88
AMPK-alpha2	PRKAA2	95
ANKK1	ANKK1	79
ARK5	NUAK1	92
ASK1	MAP3K5	100
ASK2	MAP3K6	90
AURKA	AURKA	84
AURKB	AURKB	80
AURKC	AURKC	86
AXL	AXL	96
BIKE	BMP2K	77

BLK	BLK	99
BMPR1A	BMPR1A	92
BMPR1B	BMPR1B	76
BMPR2	BMPR2	69
BMX	BMX	100
BRAF	BRAF	92
BRAF(V600E)	BRAF	91
BRK	PTK6	83
BRSK1	BRSK1	87
BRSK2	BRSK2	94
BTK	BTK	100
BUB1	BUB1	79
CAMK1	CAMK1	95
CAMK1B	PNCK	81
CAMK1D	CAMK1D	91
CAMK1G	CAMK1G	86
CAMK2A	CAMK2A	87
CAMK2B	CAMK2B	89
CAMK2D	CAMK2D	93
CAMK2G	CAMK2G	92
CAMK4	CAMK4	91
CAMKK1	CAMKK1	94
CAMKK2	CAMKK2	93
CASK	CASK	96
CDC2L1	CDK11B	87
CDC2L2	CDC2L2	93
CDC2L5	CDK13	91
CDK11	CDK19	94
CDK2	CDK2	96
CDK3	CDK3	91
CDK4	CDK4	87
CDK4-cyclinD1	CDK4	73
CDK4-cyclinD3	CDK4	61
CDK5	CDK5	86
CDK7	CDK7	41
CDK8	CDK8	100
CDK9	CDK9	91
CDKL1	CDKL1	81
CDKL2	CDKL2	88
CDKL3	CDKL3	100
CDKL5	CDKL5	68
CHEK1	CHEK1	88
CHEK2	CHEK2	95
CIT	CIT	90

CLK1	CLK1	91
CLK2	CLK2	92
CLK3	CLK3	86
CLK4	CLK4	88
CSF1R	CSF1R	98
CSF1R-autoinhibited	CSF1R	76
CSK	CSK	90
CSNK1A1	CSNK1A1	79
CSNK1A1L	CSNK1A1L	88
CSNK1D	CSNK1D	95
CSNK1E	CSNK1E	94
CSNK1G1	CSNK1G1	85
CSNK1G2	CSNK1G2	87
CSNK1G3	CSNK1G3	97
CSNK2A1	CSNK2A1	75
CSNK2A2	CSNK2A2	87
CTK	MATK	76
DAPK1	DAPK1	91
DAPK2	DAPK2	86
DAPK3	DAPK3	94
DCAMKL1	DCLK1	79
DCAMKL2	DCLK2	87
DCAMKL3	DCLK3	91
DDR1	DDR1	91
DDR2	DDR2	77
DLK	MAP3K12	82
DMPK	DMPK	96
DMPK2	CDC42BPG	89
DRAK1	STK17A	92
DRAK2	STK17B	92
DYRK1A	DYRK1A	69
DYRK1B	DYRK1B	95
DYRK2	DYRK2	80
EGFR	EGFR	75
EGFR(E746-A750del)	EGFR	73
EGFR(G719C)	EGFR	90
EGFR(G719S)	EGFR	90
EGFR(L747-E749del, A750P)	EGFR	71
EGFR(L747-S752del, P753S)	EGFR	77
EGFR(L747-T751del,Sins)	EGFR	88
EGFR(L858R)	EGFR	77
EGFR(L858R,T790M)	EGFR	58
EGFR(L861Q)	EGFR	97
EGFR(S752-I759del)	EGFR	78

EGFR(T790M)	EGFR	93
EIF2AK1	EIF2AK1	73
EPHA1	EPHA1	78
EPHA2	EPHA2	100
EPHA3	EPHA3	91
EPHA4	EPHA4	90
EPHA5	EPHA5	85
EPHA6	EPHA6	95
EPHA7	EPHA7	93
EPHA8	EPHA8	94
EPHB1	EPHB1	93
EPHB2	EPHB2	99
EPHB3	EPHB3	95
EPHB4	EPHB4	100
EPHB6	EPHB6	92
ERBB2	ERBB2	95
ERBB3	ERBB3	100
ERBB4	ERBB4	100
ERK1	MAPK3	94
ERK2	MAPK1	92
ERK3	MAPK6	90
ERK4	MAPK4	91
ERK5	MAPK7	90
ERK8	MAPK15	90
ERN1	ERN1	69
FAK	PTK2	100
FER	FER	98
FES	FES	97
FGFR1	FGFR1	94
FGFR2	FGFR2	100
FGFR3	FGFR3	100
FGFR3(G697C)	FGFR3	83
FGFR4	FGFR4	86
FGR	FGR	100
FLT1	FLT1	83
FLT3	FLT3	97
FLT3(D835H)	FLT3	92
FLT3(D835V)	FLT3	76
FLT3(D835Y)	FLT3	68
FLT3(ITD)	FLT3	80
FLT3(ITD,D835V)	FLT3	91
FLT3(ITD,F691L)	FLT3	80
FLT3(K663Q)	FLT3	71
FLT3(N841I)	FLT3	85

FLT3(R834Q)	FLT3	91
FLT3-autoinhibited	FLT3	87
FLT4	FLT4	92
FRK	FRK	89
FYN	FYN	81
GAK	GAK	87
GCN2(Kin.Dom.2,S808G)	EIF2AK4	89
GRK1	GRK1	94
GRK2	ADRBK1	56
GRK3	ADRBK2	74
GRK4	GRK4	84
GRK7	GRK7	71
GSK3A	GSK3A	96
GSK3B	GSK3B	71
HASPIN	GSG2	95
HCK	HCK	84
HIPK1	HIPK1	71
HIPK2	HIPK2	78
HIPK3	HIPK3	79
HIPK4	HIPK4	94
HPK1	MAP4K1	80
HUNK	HUNK	93
ICK	ICK	66
IGF1R	IGF1R	93
IKK-alpha	CHUK	81
IKK-beta	IKBKB	79
IKK-epsilon	IKBKE	90
INSR	INSR	78
INSRR	INSRR	99
IRAK1	IRAK1	82
IRAK3	IRAK3	83
IRAK4	IRAK4	93
ITK	ITK	96
JAK1(JH1domain-catalytic)	JAK1	91
JAK1(JH2domain-pseudokinase)	JAK1	88
JAK2(JH1domain-catalytic)	JAK2	97
JAK3(JH1domain-catalytic)	JAK3	72
JNK1	MAPK8	88
JNK2	MAPK9	84
JNK3	MAPK10	81
KIT	KIT	92
KIT(A829P)	KIT	95
KIT(D816H)	KIT	85
KIT(D816V)	KIT	100

KIT(L576P)	KIT	92
KIT(V559D)	KIT	95
KIT(V559D,T670I)	KIT	93
KIT(V559D,V654A)	KIT	94
KIT-autoinhibited	KIT	85
LATS1	LATS1	88
LATS2	LATS2	79
LCK	LCK	96
LIMK1	LIMK1	18
LIMK2	LIMK2	6.8
LKB1	STK11	100
LOK	STK10	96
LRRK2	LRRK2	97
LRRK2(G2019S)	LRRK2	80
LTK	LTK	91
LYN	LYN	78
LZK	MAP3K13	85
MAK	MAK	94
MAP3K1	MAP3K1	78
MAP3K15	MAP3K15	88
MAP3K2	MAP3K2	83
MAP3K3	MAP3K3	83
MAP3K4	MAP3K4	68
MAP4K2	MAP4K2	87
MAP4K3	MAP4K3	93
MAP4K4	MAP4K4	100
MAP4K5	MAP4K5	100
MAPKAPK2	MAPKAPK2	100
MAPKAPK5	MAPKAPK5	70
MARK1	MARK1	92
MARK2	MARK2	92
MARK3	MARK3	88
MARK4	MARK4	100
MAST1	MAST1	75
MEK1	MAP2K1	65
MEK2	MAP2K2	78
MEK3	MAP2K3	75
MEK4	MAP2K4	89
MEK5	MAP2K5	97
MEK6	MAP2K6	85
MELK	MELK	97
MERTK	MERTK	95
MET	MET	88
MET(M1250T)	MET	97

MET(Y1235D)	MET	94
MINK	MINK1	98
MKK7	MAP2K7	22
MKNK1	MKNK1	78
MKNK2	MKNK2	77
MLCK	MYLK3	99
MLK1	MAP3K9	96
MLK2	MAP3K10	79
MLK3	MAP3K11	93
MRCKA	CDC42BPA	94
MRCKB	CDC42BPB	97
MST1	STK4	90
MST1R	MST1R	88
MST2	STK3	92
MST3	STK24	100
MST4	MST4	91
MTOR	MTOR	98
MUSK	MUSK	100
MYLK	MYLK	76
MYLK2	MYLK2	82
MYLK4	MYLK4	91
MYO3A	MYO3A	80
MYO3B	MYO3B	100
NDR1	STK38	80
NDR2	STK38L	82
NEK1	NEK1	87
NEK10	NEK10	95
NEK11	NEK11	77
NEK2	NEK2	81
NEK3	NEK3	87
NEK4	NEK4	100
NEK5	NEK5	93
NEK6	NEK6	93
NEK7	NEK7	88
NEK9	NEK9	86
NIK	MAP3K14	66
NIM1	MGC42105	77
NLK	NLK	100
OSR1	OXSR1	90
p38-alpha	MAPK14	100
p38-beta	MAPK11	98
p38-delta	MAPK13	92
p38-gamma	MAPK12	80
PAK1	PAK1	89

PAK2	PAK2	51
PAK3	PAK3	69
PAK4	PAK4	89
PAK6	PAK6	89
PAK7	PAK7	89
PCTK1	CDK16	76
PCTK2	CDK17	92
PCTK3	CDK18	97
PDGFRA	PDGFRA	94
PDGFRB	PDGFRB	93
PDPK1	PDPK1	100
PFCDPK1(P.falciparum)	CDPK1	88
PFPK5(P.falciparum)	MAL13P1.279	89
PFTAIRE2	CDK15	85
PFTK1	CDK14	89
PHKG1	PHKG1	100
PHKG2	PHKG2	99
PIK3C2B	PIK3C2B	99
PIK3C2G	PIK3C2G	100
PIK3CA	PIK3CA	87
PIK3CA(C420R)	PIK3CA	76
PIK3CA(E542K)	PIK3CA	84
PIK3CA(E545A)	PIK3CA	99
PIK3CA(E545K)	PIK3CA	75
PIK3CA(H1047L)	PIK3CA	55
PIK3CA(H1047Y)	PIK3CA	68
PIK3CA(I800L)	PIK3CA	82
PIK3CA(M1043I)	PIK3CA	65
PIK3CA(Q546K)	PIK3CA	92
PIK3CB	PIK3CB	100
PIK3CD	PIK3CD	77
PIK3CG	PIK3CG	90
PIK4CB	PI4KB	66
PIKFYVE	PIKFYVE	90
PIM1	PIM1	88
PIM2	PIM2	91
PIM3	PIM3	80
PIP5K1A	PIP5K1A	91
PIP5K1C	PIP5K1C	96
PIP5K2B	PIP4K2B	89
PIP5K2C	PIP4K2C	99
PKAC-alpha	PRKACA	84
PKAC-beta	PRKACB	100
PKMYT1	PKMYT1	96

PKN1	PKN1	100
PKN2	PKN2	97
PKNB(M.tuberculosis)	pknB	97
PLK1	PLK1	92
PLK2	PLK2	84
PLK3	PLK3	78
PLK4	PLK4	88
PRKCD	PRKCD	100
PRKCE	PRKCE	100
PRKCH	PRKCH	92
PRKCI	PRKCI	88
PRKCQ	PRKCQ	74
PRKD1	PRKD1	90
PRKD2	PRKD2	96
PRKD3	PRKD3	100
PRKG1	PRKG1	96
PRKG2	PRKG2	87
PRKR	EIF2AK2	84
PRKX	PRKX	88
PRP4	PRPF4B	100
PYK2	PTK2B	89
QSK	KIAA0999	83
RAF1	RAF1	99
RET	RET	91
RET(M918T)	RET	94
RET(V804L)	RET	95
RET(V804M)	RET	70
RIOK1	RIOK1	96
RIOK2	RIOK2	81
RIOK3	RIOK3	89
RIPK1	RIPK1	93
RIPK2	RIPK2	83
RIPK4	RIPK4	70
RIPK5	DSTYK	82
ROCK1	ROCK1	73
ROCK2	ROCK2	76
ROS1	ROS1	83
RPS6KA4(Kin.Dom.1-N-terminal)	RPS6KA4	99
RPS6KA4(Kin.Dom.2-C-terminal)	RPS6KA4	75
RPS6KA5(Kin.Dom.1-N-terminal)	RPS6KA5	98
RPS6KA5(Kin.Dom.2-C-terminal)	RPS6KA5	76
RSK1(Kin.Dom.1-N-terminal)	RPS6KA1	96
RSK1(Kin.Dom.2-C-terminal)	RPS6KA1	94
RSK2(Kin.Dom.1-N-terminal)	RPS6KA3	79

RSK2(Kin.Dom.2-C-terminal)	RPS6KA3	58
RSK3(Kin.Dom.1-N-terminal)	RPS6KA2	97
RSK3(Kin.Dom.2-C-terminal)	RPS6KA2	96
RSK4(Kin.Dom.1-N-terminal)	RPS6KA6	94
RSK4(Kin.Dom.2-C-terminal)	RPS6KA6	96
S6K1	RPS6KB1	98
SBK1	SBK1	93
SGK	SGK1	81
SgK110	SgK110	86
SGK2	SGK2	96
SGK3	SGK3	100
SIK	SIK1	74
SIK2	SIK2	100
SLK	SLK	98
SNARK	NUAK2	92
SNRK	SNRK	90
SRC	SRC	87
SRMS	SRMS	73
SRPK1	SRPK1	100
SRPK2	SRPK2	89
SRPK3	SRPK3	100
STK16	STK16	75
STK33	STK33	100
STK35	STK35	95
STK36	STK36	96
STK39	STK39	91
SYK	SYK	100
TAK1	MAP3K7	100
TAOK1	TAOK1	88
TAOK2	TAOK2	80
TAOK3	TAOK3	100
TBK1	TBK1	100
TEC	TEC	93
TESK1	TESK1	82
TGFBR1	TGFBR1	100
TGFBR2	TGFBR2	89
TIE1	TIE1	99
TIE2	TEK	94
TLK1	TLK1	92
TLK2	TLK2	94
TNIK	TNIK	96
TNK1	TNK1	80
TNK2	TNK2	89
TNNI3K	TNNI3K	88

TRKA	NTRK1	85
TRKB	NTRK2	66
TRKC	NTRK3	67
TRPM6	TRPM6	95
TSSK1B	TSSK1B	97
TSSK3	TSSK3	89
TTK	TTK	93
TXK	TXK	72
TYK2(JH1domain-catalytic)	TYK2	93
TYK2(JH2domain-pseudokinase)	TYK2	90
TYRO3	TYRO3	79
ULK1	ULK1	85
ULK2	ULK2	75
ULK3	ULK3	89
VEGFR2	KDR	96
VPS34	PIK3C3	76
VRK2	VRK2	22
WEE1	WEE1	100
WEE2	WEE2	88
WNK1	WNK1	85
WNK2	WNK2	68
WNK3	WNK3	97
WNK4	WNK4	77
YANK1	STK32A	75
YANK2	STK32B	93
YANK3	STK32C	94
YES	YES1	86
YSK1	STK25	91
YSK4	MAP3K19	83
ZAK	ZAK	72
ZAP70	ZAP70	75

Binding assay	% inhibition at 10 μM
A_{2A} (agonist)[a]	15.5
α_{1A} (antagonist)[a]	7.0
$\alpha 2_A$ (antagonist)[a]	6.9
β_1 (agonist)[a]	-1.5
β_2 (antagonist)[a]	-2.7
BZD (central, agonist)[b]	48.5
CB_1 (agonist)[a]	88.3
CB_2 (agonist)[a]	19.2
CCK_1 (CCK_A, agonist)[a]	-1.7
D_1 (antagonist)[a]	4.4
D_{2S} (agonist)[a]	8.6
ET_A (agonist)[a]	-1.1
NMDA (antagonist)[b]	0.1
H_1 (antagonist)[a]	8.0
H_2 (antagonist)[a]	10.2
MAO-A (antagonist)[b]	12.0
M_1 (antagonist)[a]	6.5
M_2 (antagonist)[a]	12.8
M_3 (antagonist)[a]	-11.8
N neuronal α4β2 (agonist)[a]	-8.8
δ (DOP, agonist)[a]	39.4
kappa (KOP, agonist)[a]	48.8
μ (MOP, agonist)[a]	15.6
$5\text{-}HT_{1A}$ (agonist)[a]	-8.2
$5\text{-}HT_{1B}$ (antagonist)[a]	30.2
$5\text{-}HT_{2A}$ (agonist)[a]	36.7
$5\text{-}HT_{2B}$ (agonist)[a]	16.3
$5\text{-}HT_3$ (antagonist)[a]	-8.4
GR (agonist)[a]	25.1
AR (agonist)[a]	-5.2
V_{1A} (agonist)[a]	43.5
Ca^{2+} channel (L, dihydropyridine site, antagonist)[b]	22.8
Potassium Channel hERG ([3H]-Dofetilide)[a]	15.6
K_V channel (antagonist)[b]	6.3
Na^+ channel (site 2, antagonist)[b]	1.4
Norepinephrine transporter (antagonist)[a]	23.2
Dopamine transporter (antagonist)[a]	19.8
5-HT transporter (antagonist)[a]	4.1
COX1[a]	-4.5
COX2[a]	41.5
PDE3A[a]	13.3
PDE4D2[a]	5.0
Lck kinase[a]	1.7
Acetylcholinesterase[a]	-0.5

Table S8 | Selectivity profile of MDI-114215 (**85**) in the CEREP selectivity panel (Eurofins/CEREP, France). Compound binding was calculated as a % inhibition of the binding of a radioactively labelled ligand specific for each targets, whilst enzyme inhibition effect was calculated as a % inhibition of control enzyme activity. [a]Human recombinant; [b]Rat cerebral cortex. Binders with inhibition > 50% at 10 μM are highlighted.

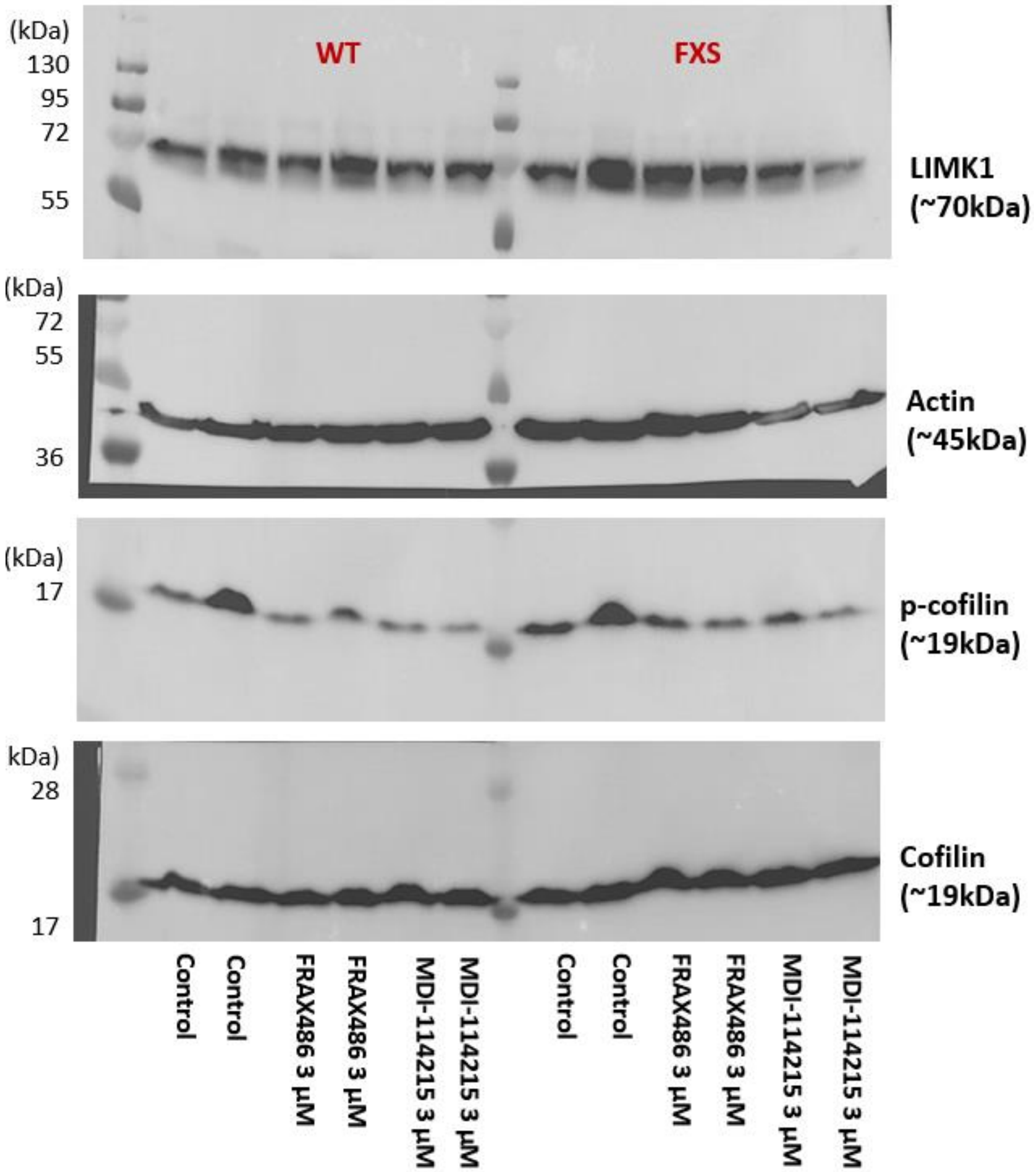

Figure S3 | Full-size Western blots of treated brain slices isolated from young WT or *Fmr1* KO (P7-9) upon incubation with 3 µM of control (DMSO, P7 WT), FRAX486 (**2**) or MDI-114215 (**85**).

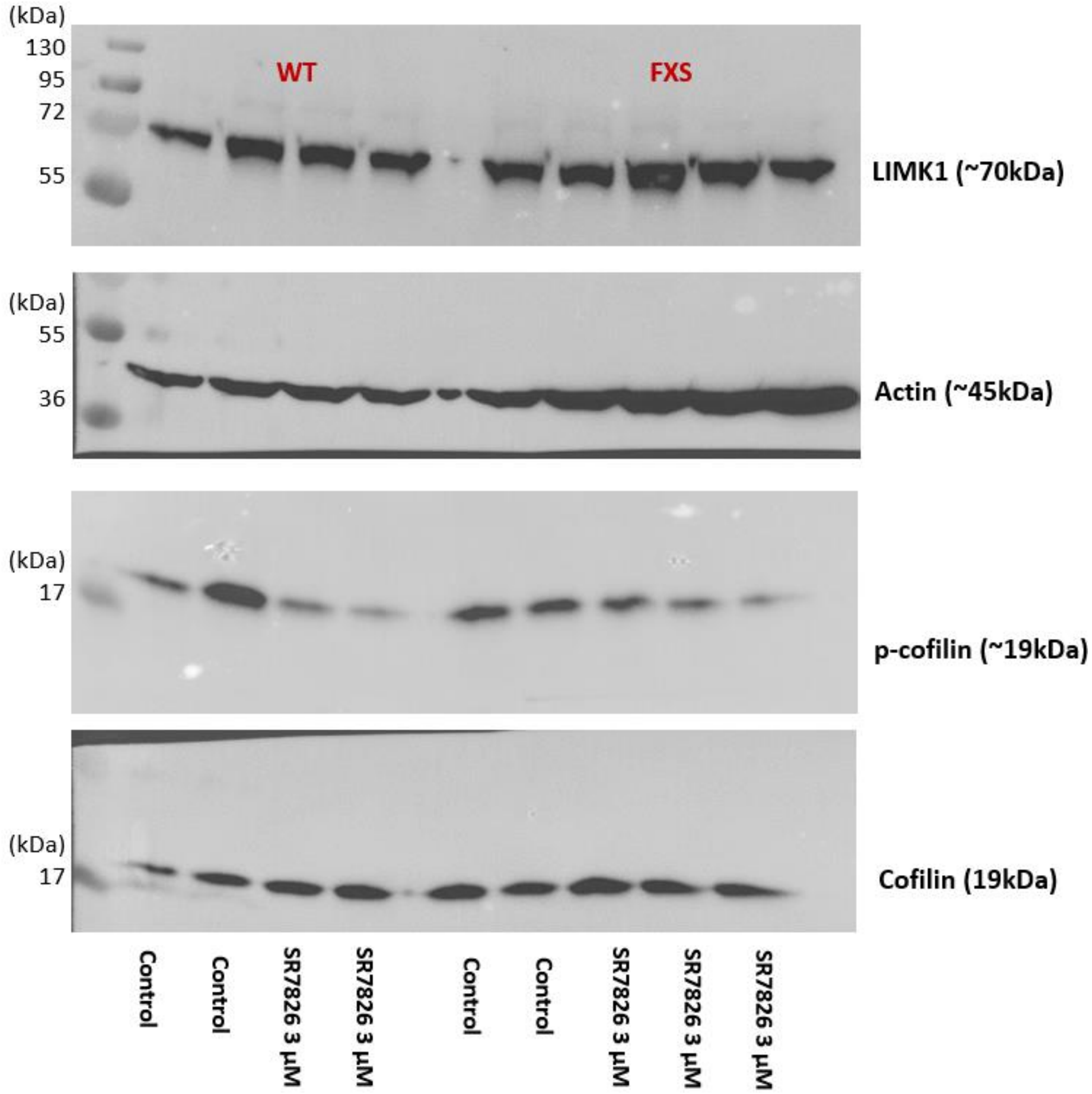

Figure S4 | Full-size Western blots of treated brain slices isolated from young WT or *Fmr1* KO (P7-9) upon incubation with 3 µM of control (DMSO, P7 WT) or SR7826 (**4**).

PDB ID	7B8W
Space group	$P2_1$
No. of molecules in the asymmetric unit	4
Unit cell dimensions a, b, c (Å), β (°)	84.7, 83.7, 96.5, 91.97
**Data collection**	
Resolution range (Å)[a]	51.20-2.80
Unique observations[a]	33406 (4392)
Average multiplicity[a]	6.8 (6.8)
Completeness (%)[a]	99.9 (99.9)
R_{merge}[a]	0.206 (1.34)
Mean ((I)/σ(I))[a]	7.0 (1.6)
Mean CC(1/2)	0.98 (0.97)
**Refinement**	
Resolution range (Å)	96.45-2.80
R-value, R_{free}	0.21, 0.29
r.m.s. deviation from ideal bond length (Å)	0.005
r.m.s. deviation from ideal bond angle (°)	1.43

Table S9 | Data collection and refinement statistics for LIMK1 co-crystal structure with TH-470 (**8**). [a]Values within parentheses refer to the highest resolution shell.

Brain slice	*Treatment*	*Mean difference*	*Degrees of freedom*	*F value*	*P value*	*R-squared*
One-way ANOVA summary	WT<br>*Fmr1* KO			30.27<br>10.76	0.0005***<br>0.0052**	0.94<br>0.82
Young WT	Control vs FRAX486	65.51	6		0.0012 **	
	Control vs MDI-114215	74.7	6		0.0006 ***	
	Control vs SR7826	58.33	6		0.0022 **	
Fmr1 KO	Control vs FRAX486	50.87	7		0.0142*	
	Control vs MDI-114215	58.7	7		0.0067 **	
	Control vs SR7826	48.82	7		0.0092 **	

Table S10 | Statistical data used to compare effect of LIMK inhibitors on WT or *Fmr1* KO hippocampal brain slices. WT brain slices were treated with control (DMSO, *n* = 4) or 3 µM FRAX486 (**2**, *n* = 2), MDI-114215 (**85**, *n* = 2) and SR7826 (**4**, *n* = 2). *Fmr1* KO brain slices were treated with control (DMSO, *n* = 4) or 3 µM FRAX486 (**2**, *n* = 2), MDI-114215 (**85**, *n* = 2) and SR7826 (**4**, *n* = 3). The test statistics are reported.

Description of rotamers for final compound characterisation

Amide rotamers were observed for asymmetric tertiary amides containing non-cyclised aliphatic/aromatic systems in various ratios. This was confirmed by VT-NMR for the parent literature TH-257 (**6**, Figure S1). For the purposes of NMR characterisation, both amide rotamers are reported but not distinguished.

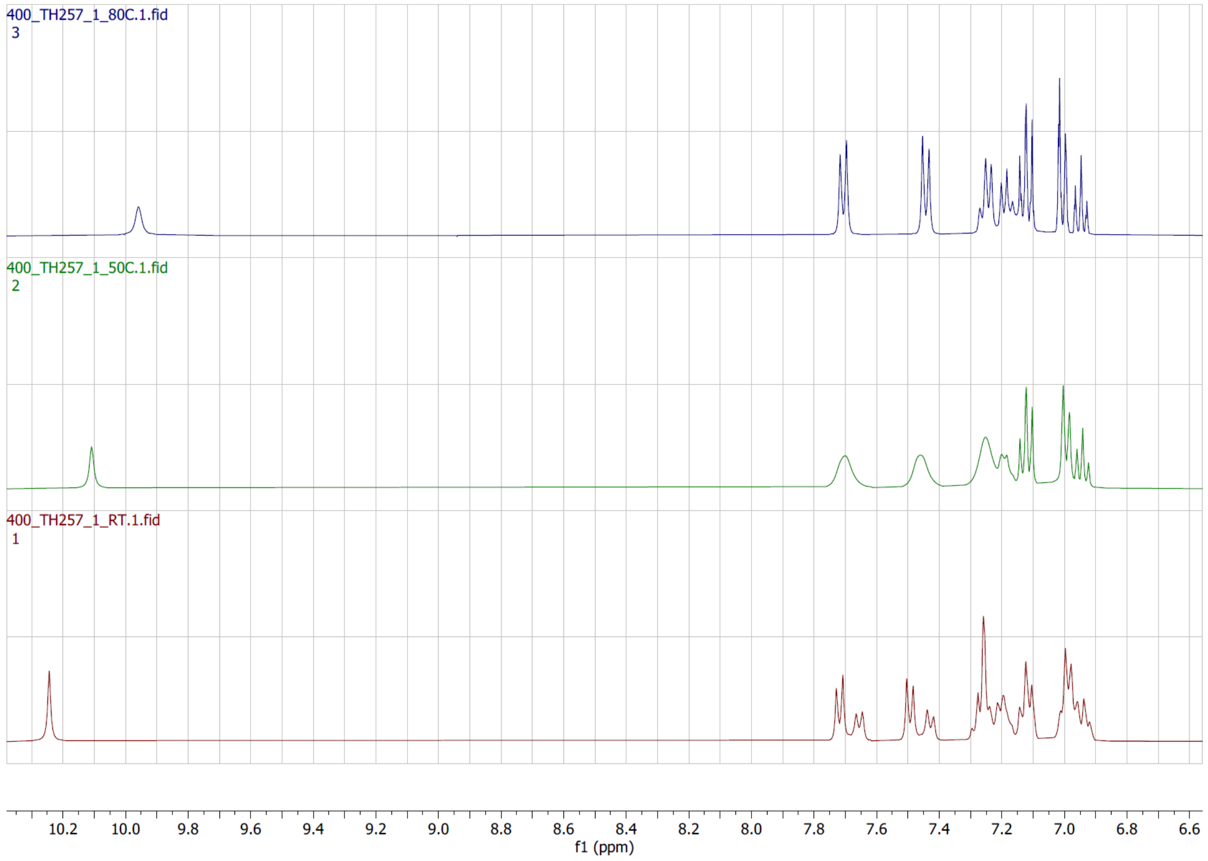

Figure S5 | Stacked VT-NMR spectra of TH-257 (**6**) aromatic region in DMSO-d_6. Rotamers are apparent when conducted at room temperature (red line) that disappears upon heating sample to 50 °C (green line) and 80 °C (blue line).

^{1}H, ^{13}C, ^{19}F NMR and UPLC data for final compounds

N-Benzyl-*N*-(4-(*N*-phenylsulfamoyl)benzyl)butyramide (**9**)

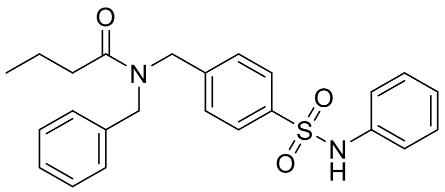

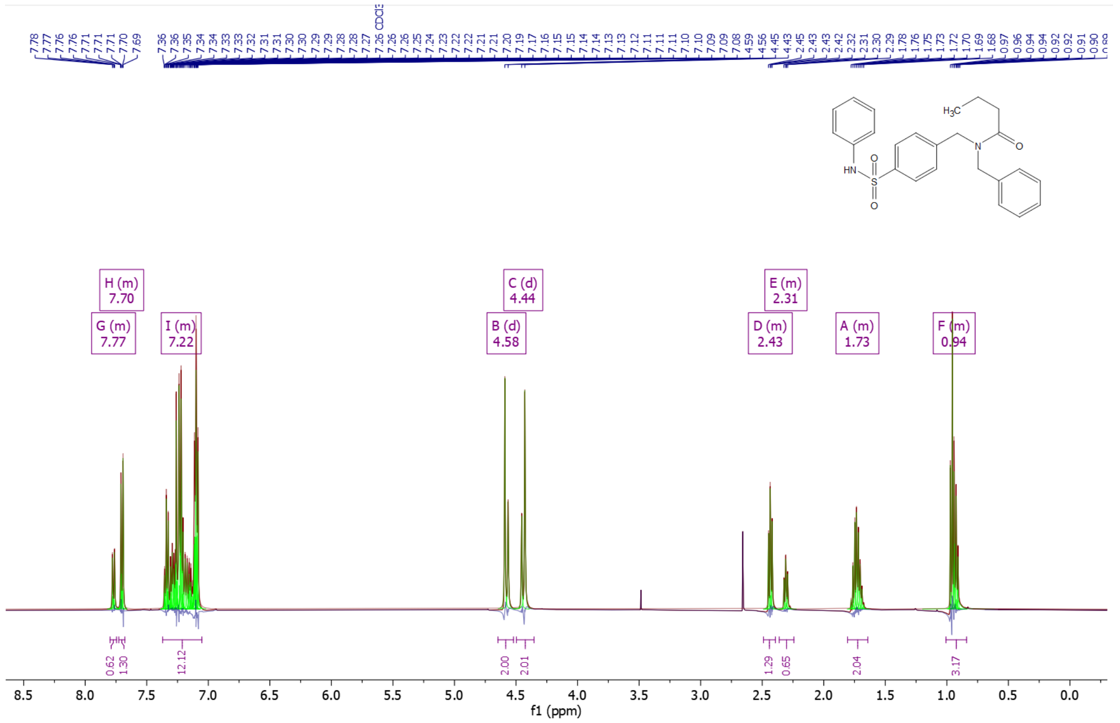

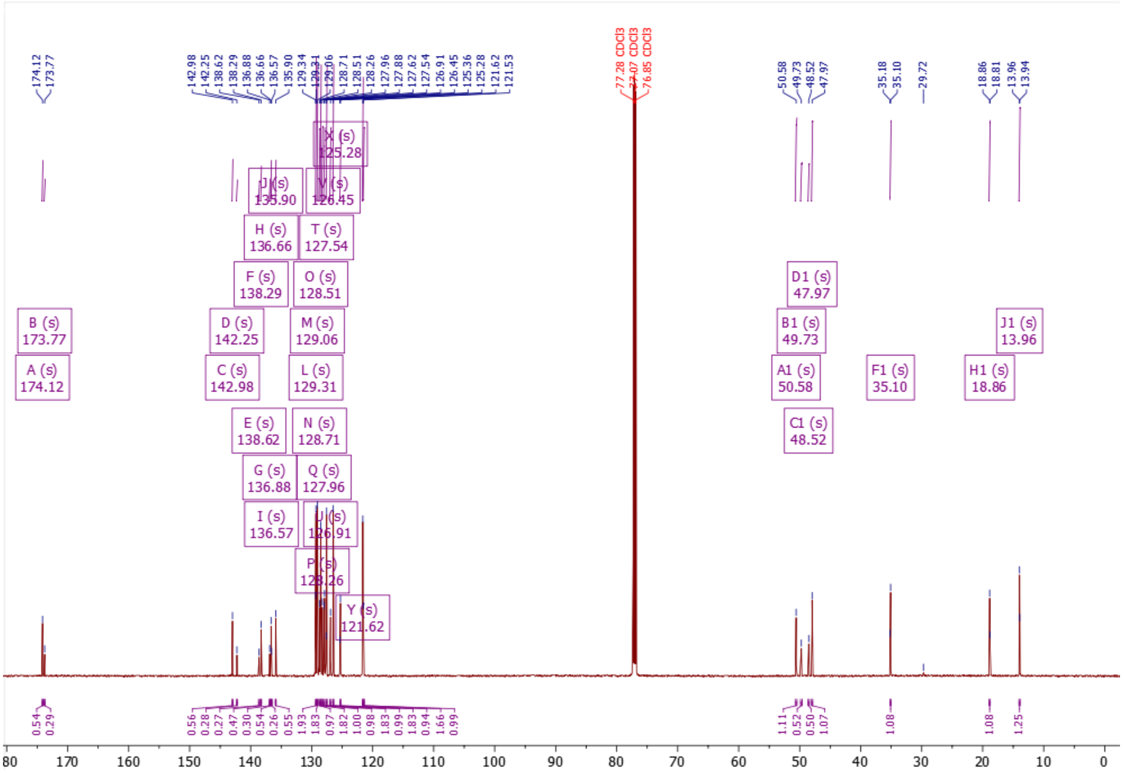

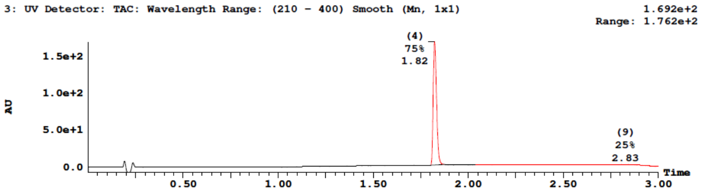

Peak Number	Compound	Time	Area %Total	Mass Found
4		1.82	74.63	Not Found
9		2.83	25.37	Not Found

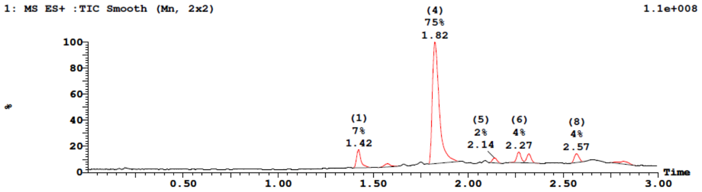

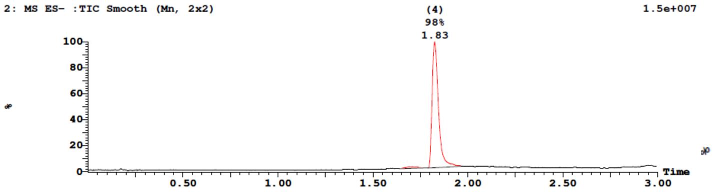

Peak ID	Compound	Time	Mass Found
4		1.82	Not Found

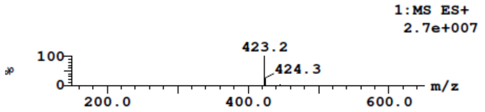

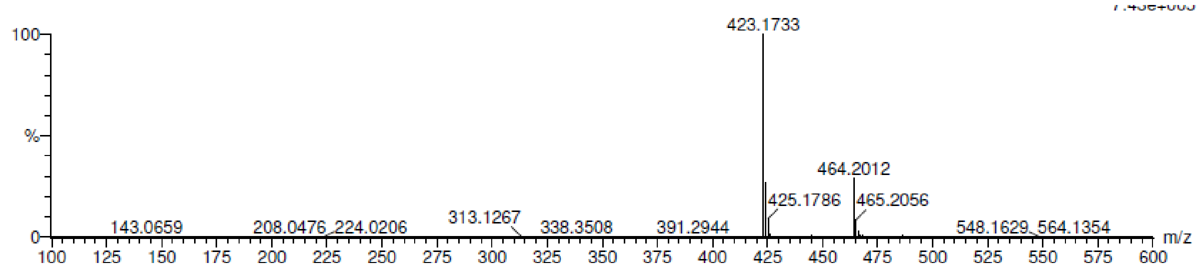

Minimum:				-1.5			
Maximum:		5.0	5.0	50.0			
Mass	Calc. Mass	mDa	PPM	DBE	i-FIT	i-FIT (Norm)	Formula
423.1733	423.1742	-0.9	-2.1	12.5	311.2	0.0	C24 H27 N2 O3 S

N-Butyl-*N*-(4-(*N*-phenylsulfamoyl)benzyl)benzamide (**10**)

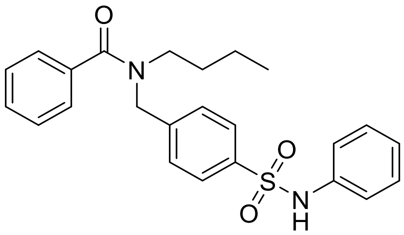

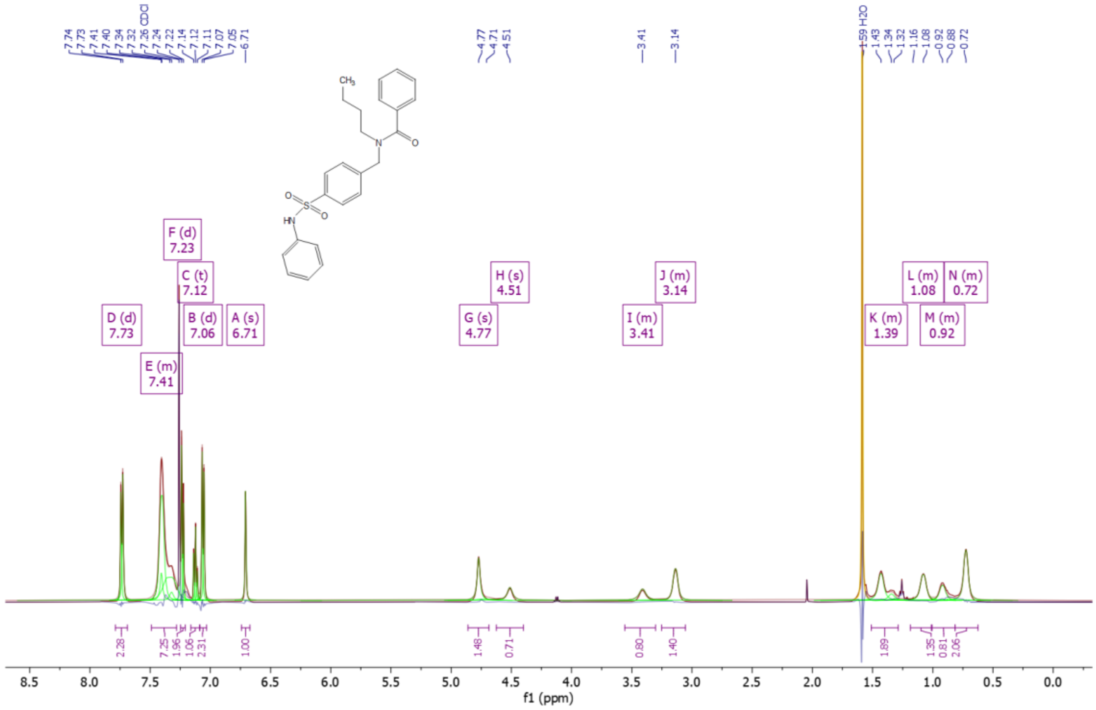

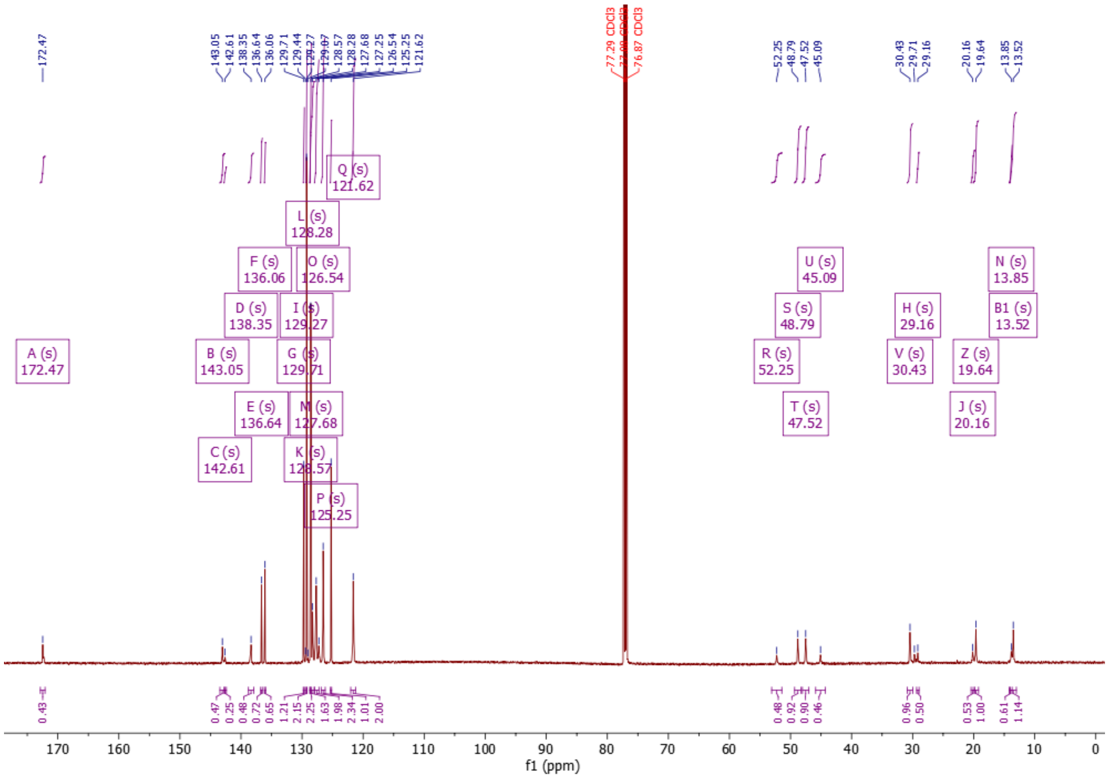

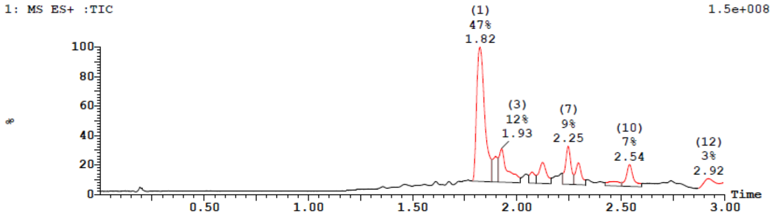

Peak Number	Compound	Time	AreaAbs	Area %Total	Width	Height	Mass Found
1		1.82	6e+006	46.52	0	1e+008	
2		1.90	7e+005	5.52	0	3e+007	
3		1.93	2e+006	12.16	0	3e+007	
4		2.07	3e+005	2.62	0	1e+007	
5		2.12	9e+005	6.75	0	2e+007	
7		2.25	1e+006	8.97	0	4e+007	
8		2.30	7e+005	5.54	0	2e+007	
9		2.47	3e+005	2.37	0	4e+006	
10		2.54	9e+005	6.64	0	2e+007	
12		2.92	4e+005	2.91	0	8e+006	

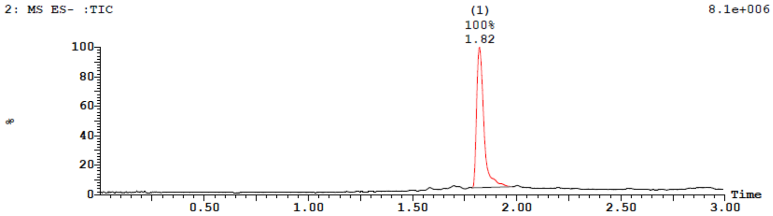

Peak Number	Compound	Time	AreaAbs	Area %Total	Width	Height	Mass Found
1		1.82	3e+005	100.00	0	8e+006	

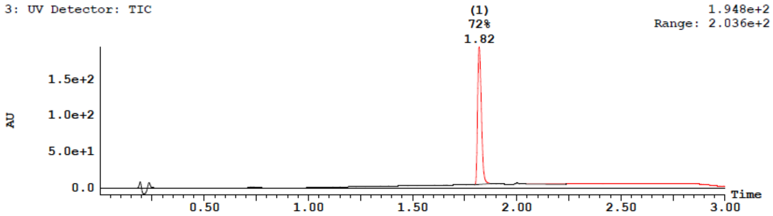

Peak ID	Compound	Time	Mass Found
1		1.82	

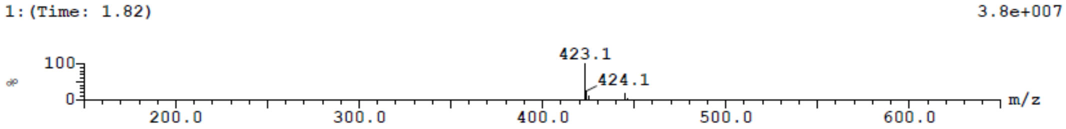

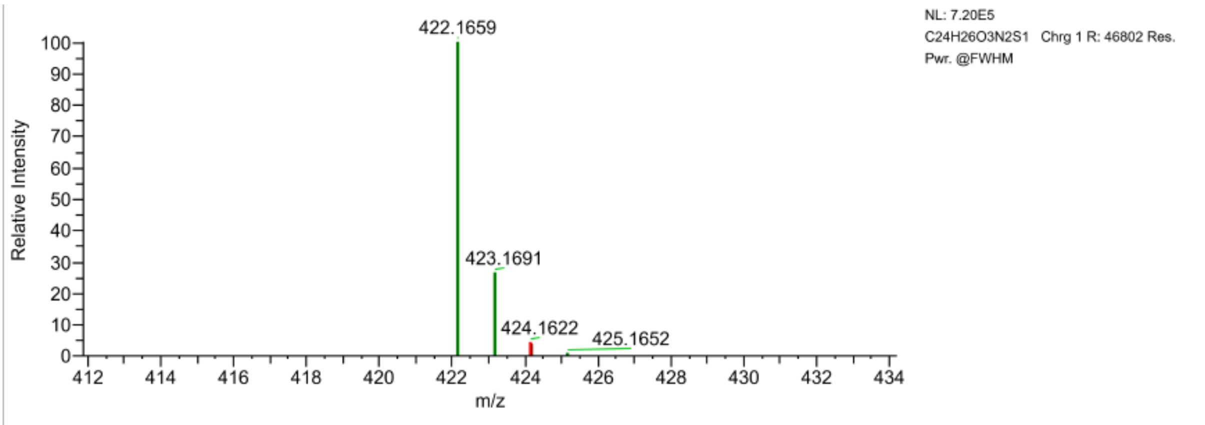

Peak Mass	Display Formula	Delta [ppm]	Theo. mass	Combined Score	MSMS Matched Frag...
422.1655	$C_{24}H_{26}O_3N_2{}^{32}S$	-0.80	422.16586	87.014709208968	(Collection)

N-Benzyl-*N*-butyl-4-(phenylsulfonamido)benzamide (**15**)

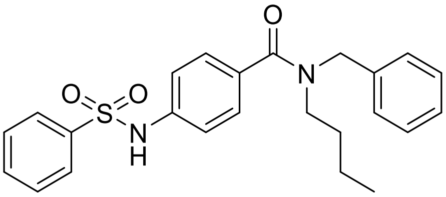

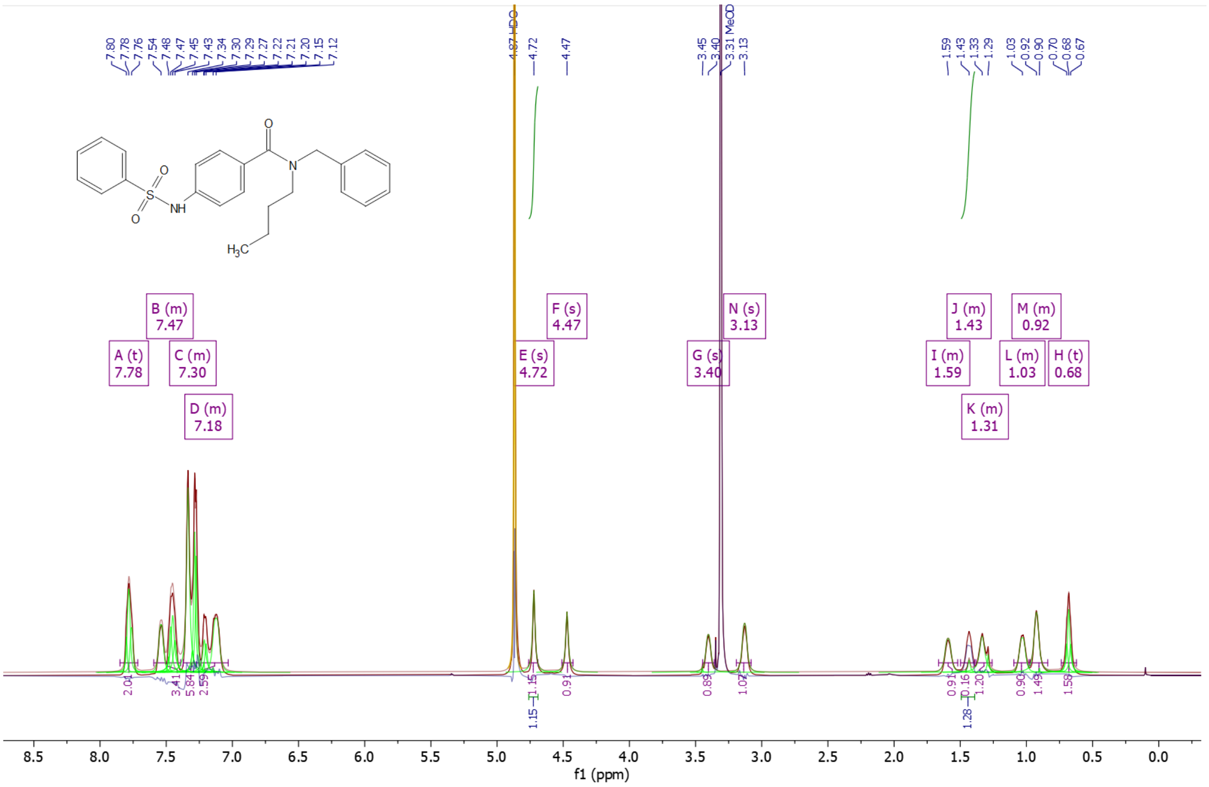

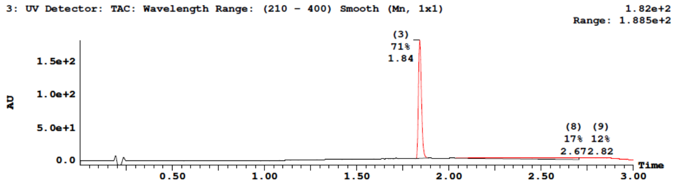

Peak Number	Compound	Time	Area %Total	Mass Found
3		1.84	70.67	Not Found
8		2.67	17.43	Not Found
9		2.82	11.91	Not Found

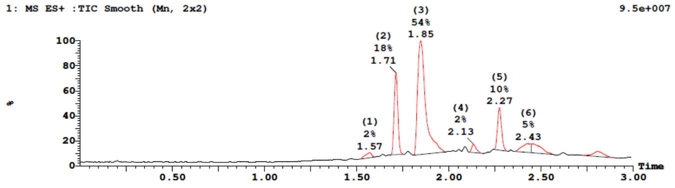

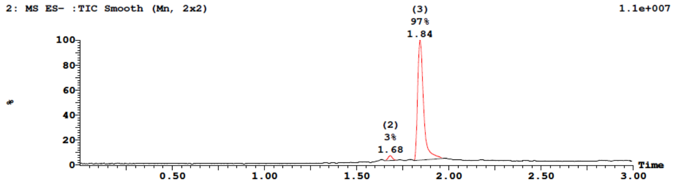

Peak ID	Compound	Time	Mass Found
3		1.85	Not Found

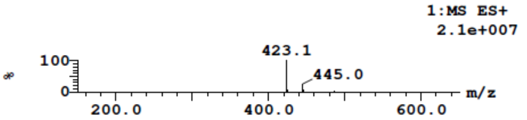

N^1-Benzyl-N^1-butyl-N^4-phenylterephthalamide (**17**)

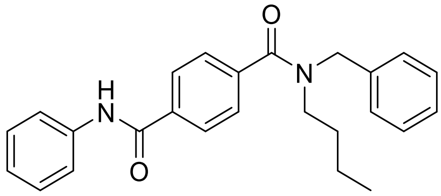

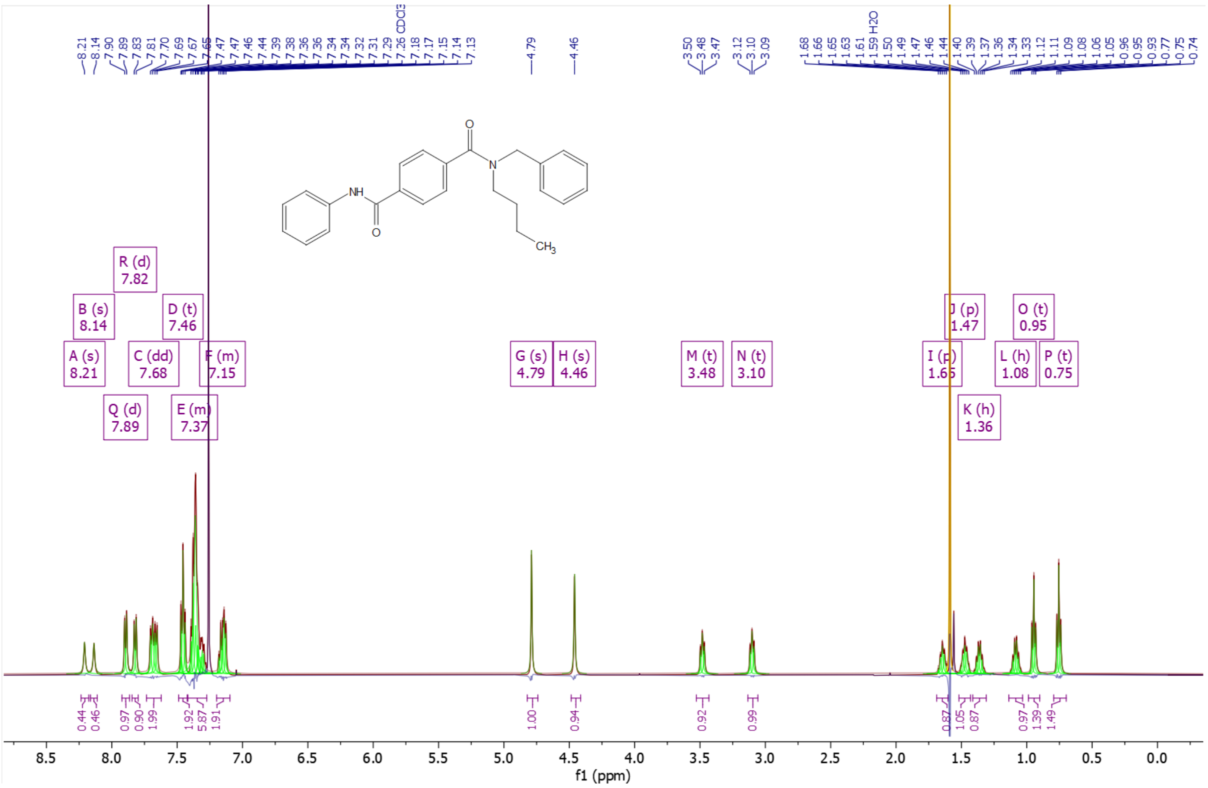

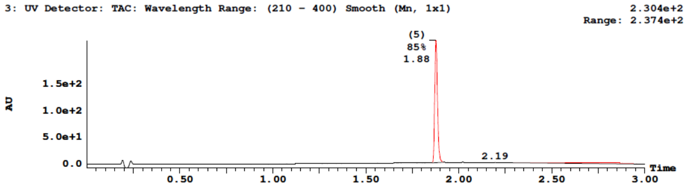

Peak Number	Compound	Time	Area %Total	Mass Found
5		1.88	85.09	Not Found
9		2.19	14.91	Not Found

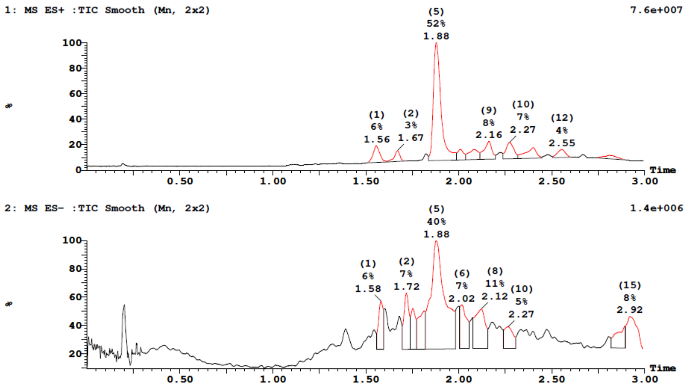

Peak ID	Compound	Time	Mass Found
5		1.88	Not Found

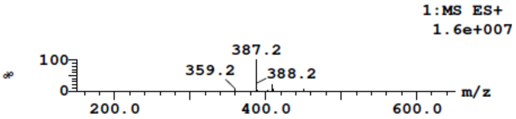

N-Benzyl-*N*-butyl-3-fluoro-4-(phenylsulfamoyl)benzamide (**19**)

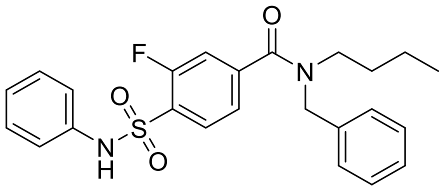

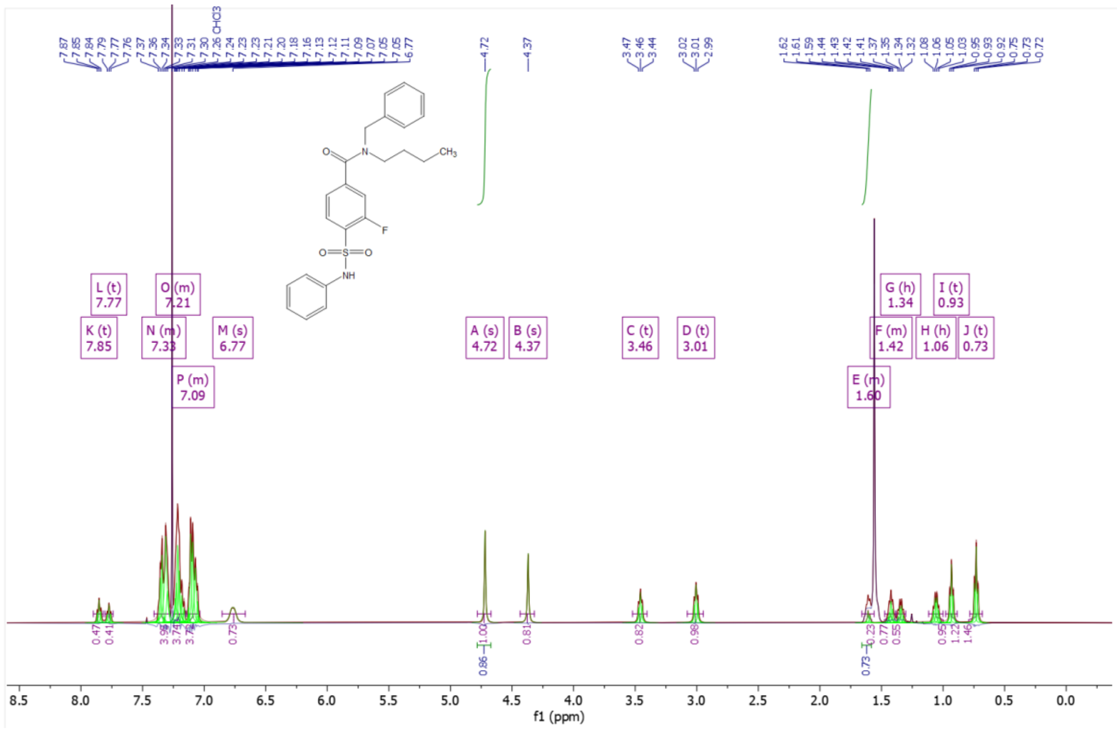

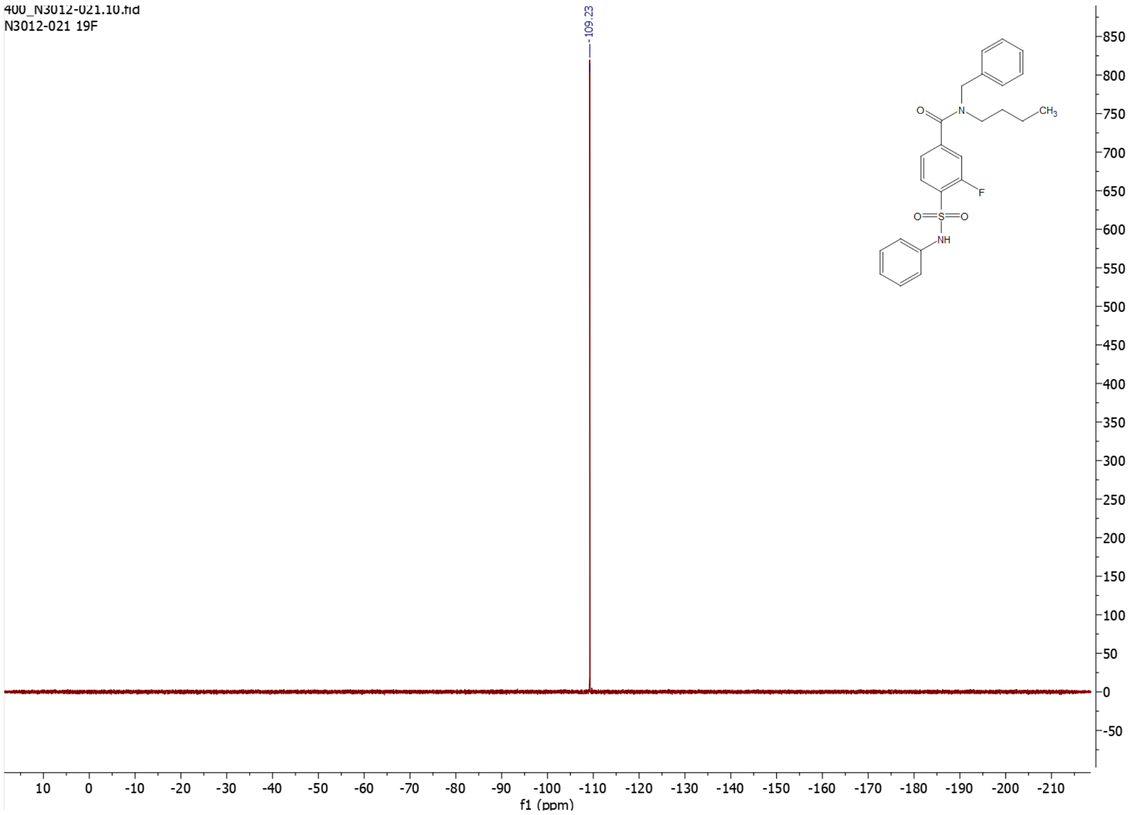

Openlynx Report - MDI UPLC MS Report - Alex B
eLN Number:N3012-021
Description:N3012-021 final
Column Name:ACQUITY UPLC® BEH C18 1.7µm
Time:18:06:47

File:Alex B718-1
Method:C:\MassLynx\Acidic BEH_Ver2.olp

Vial:1:C,7
Date:11-Jun-2019

Page 1

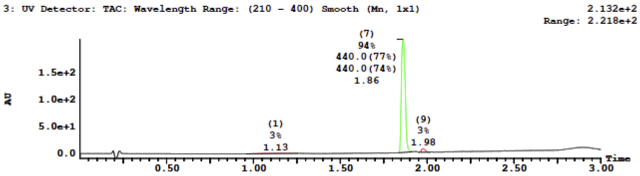

Peak Number	Compound	Time	Area %Total	Mass Found
1		1.13	2.58	Not Found
7	Found	1.86	94.42	)000, 440.0000
9		1.98	3.00	Not Found

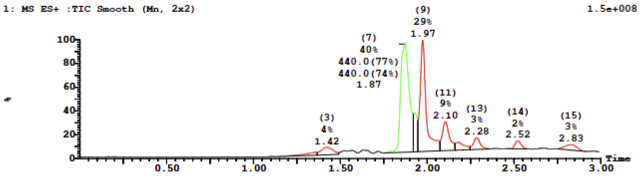

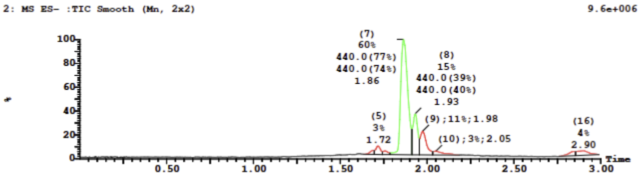

Peak ID	Compound	Time	Mass Found
7	Found	1.87	441

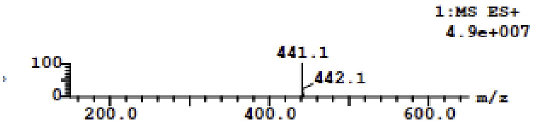

N-Benzyl-*N*-butyl-3-chloro-4-(phenylsulfamoyl)benzamide (**20**)

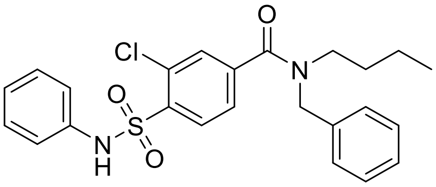

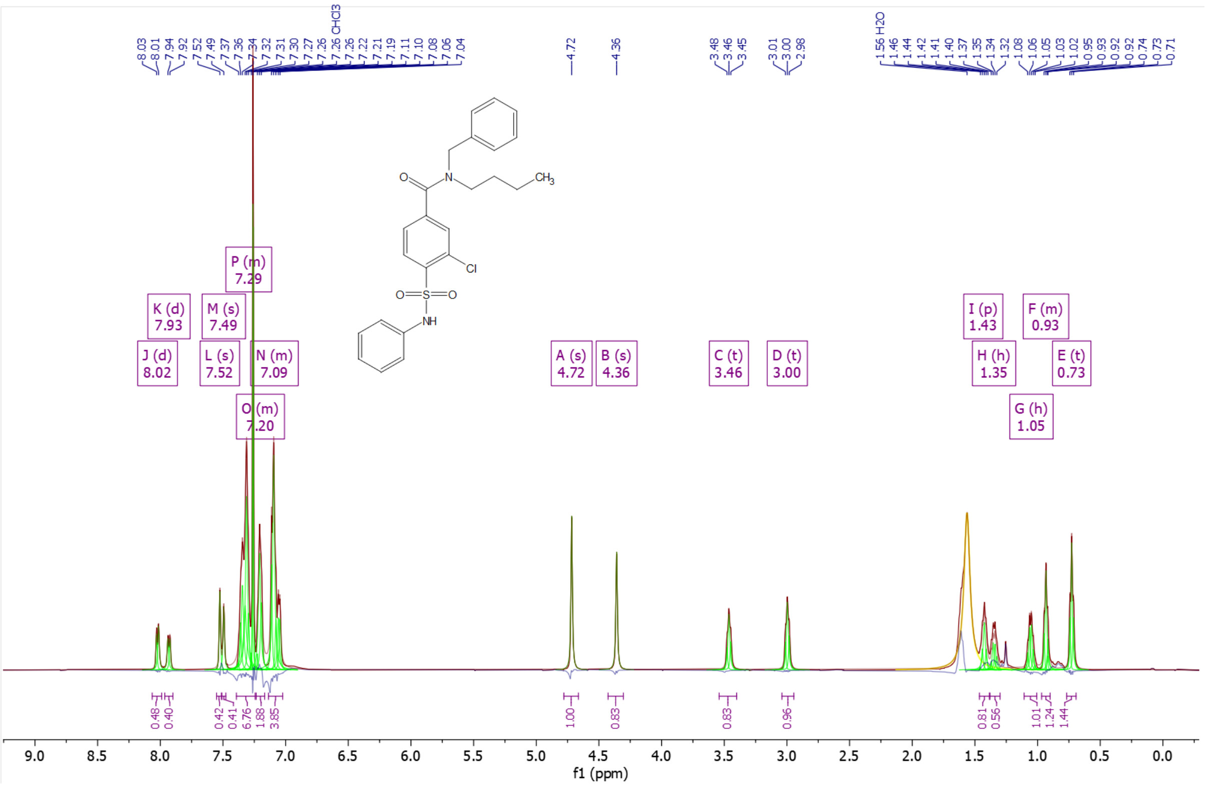

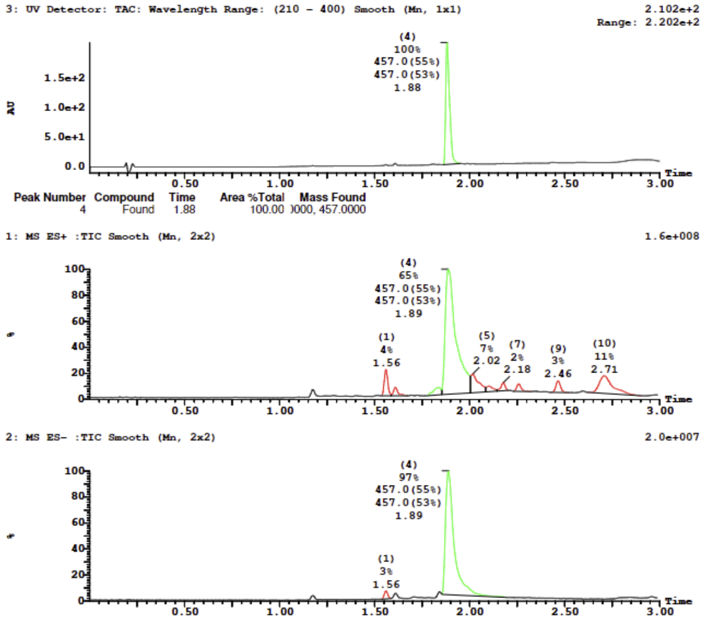

Peak ID	Compound	Time	Mass Found
4	Found	1.89	457

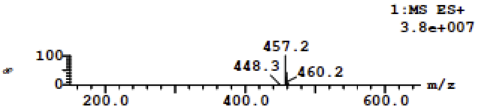

N-Benzyl-*N*-butyl-2-fluoro-4-(phenylsulfamoyl)benzamide (**21**)

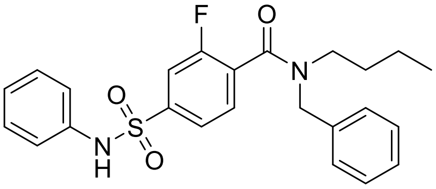

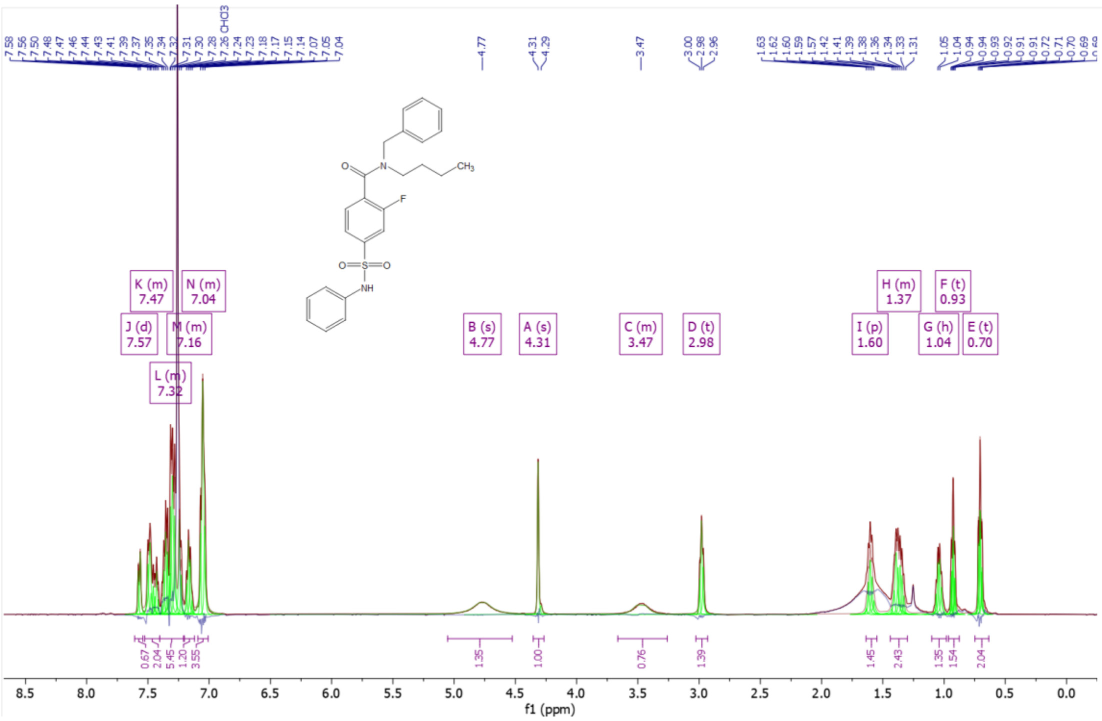

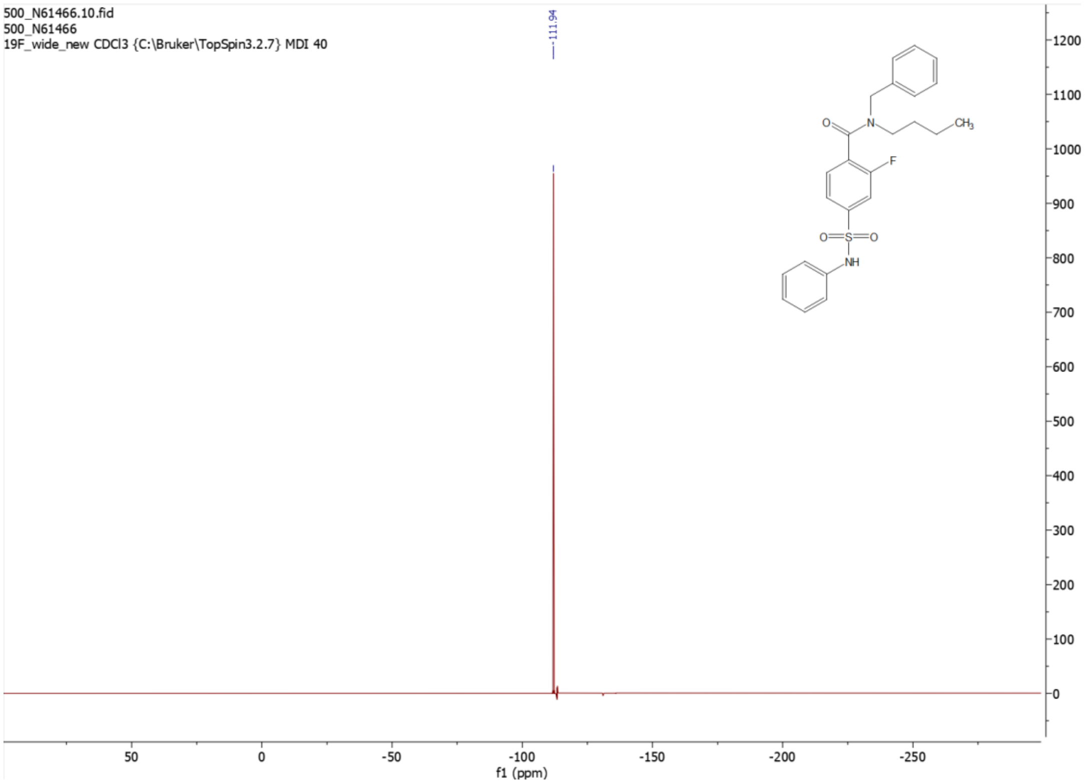

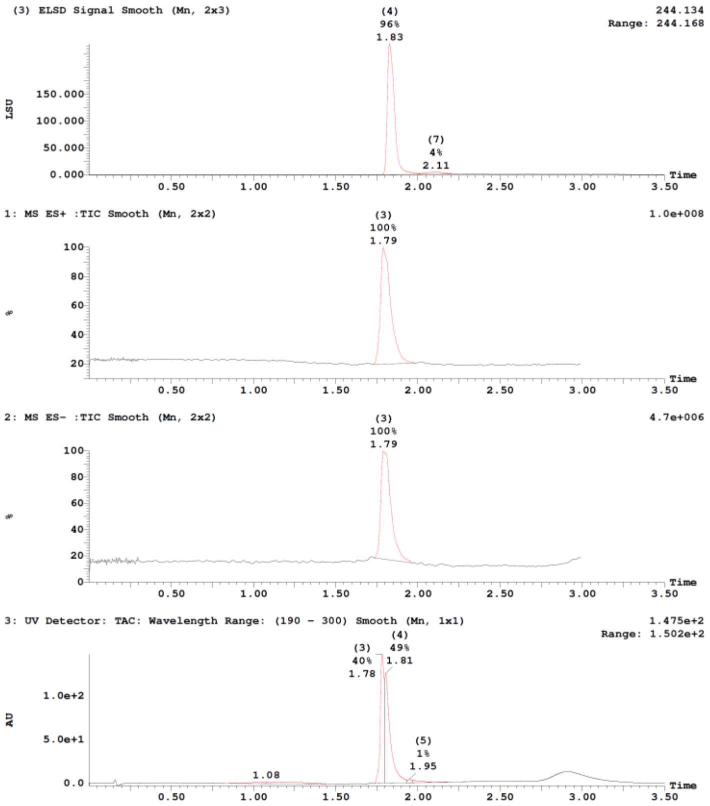

Peak ID	Compound	Time	Mass Found
3		1.79	Not Found

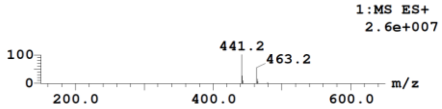

N-Benzyl-*N*-butyl-2-methyl-4-(phenylsulfamoyl)benzamide (**22**)

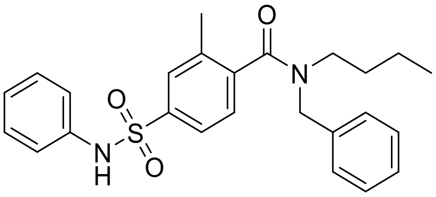

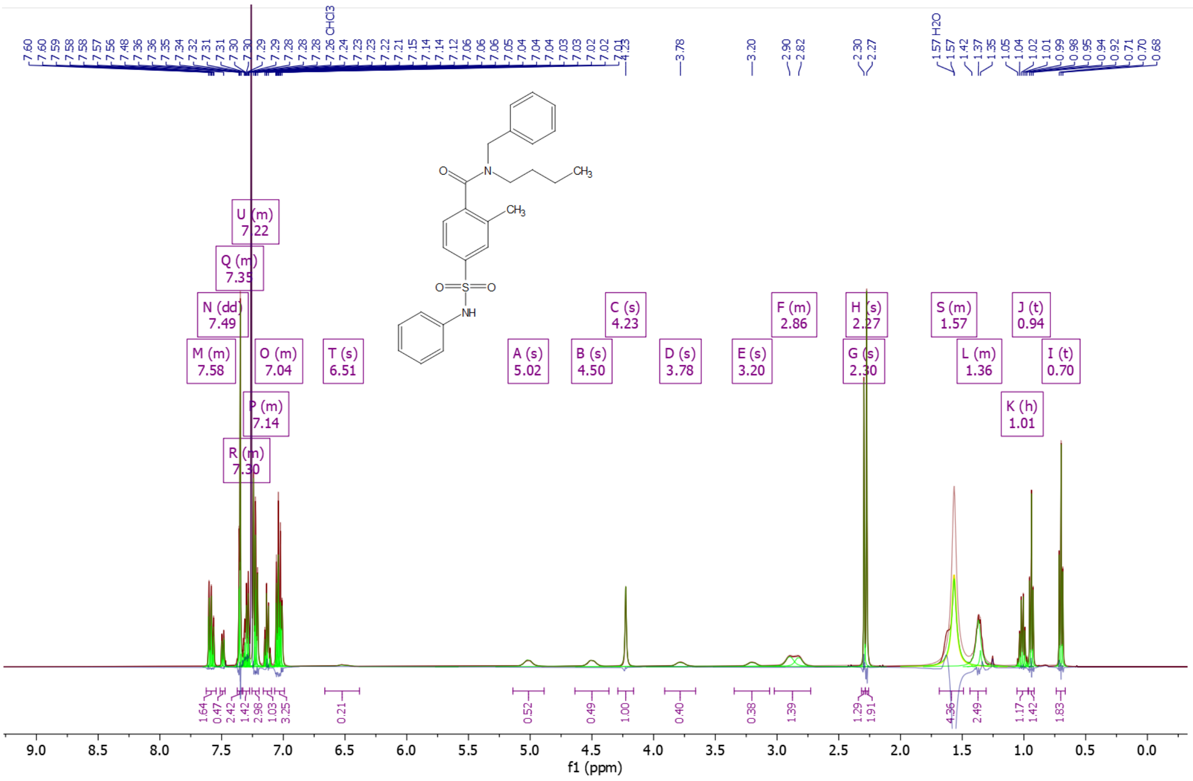

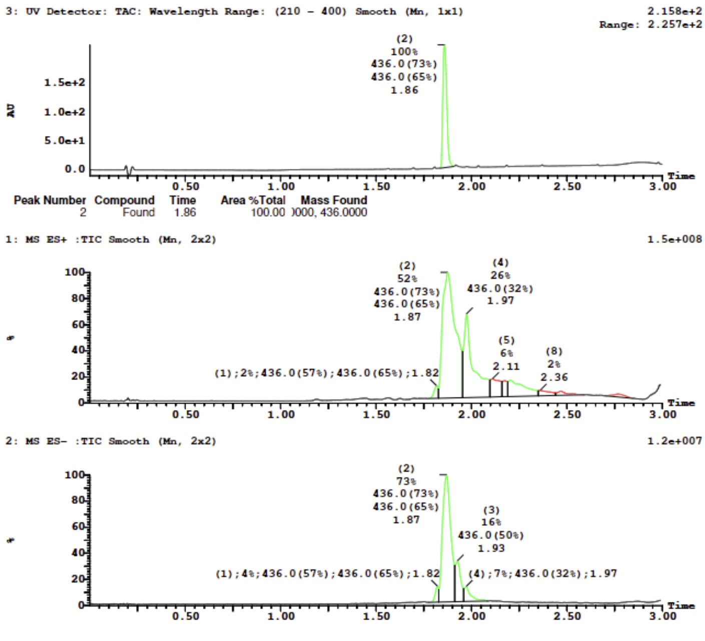

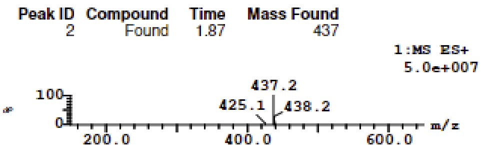

N-Benzyl-*N*-butyl-5-(phenylsulfamoyl)pyridine-2-carboxamide (**23**)

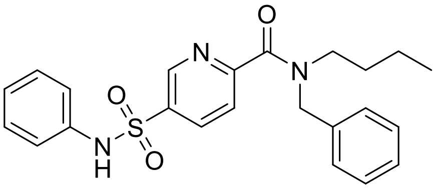

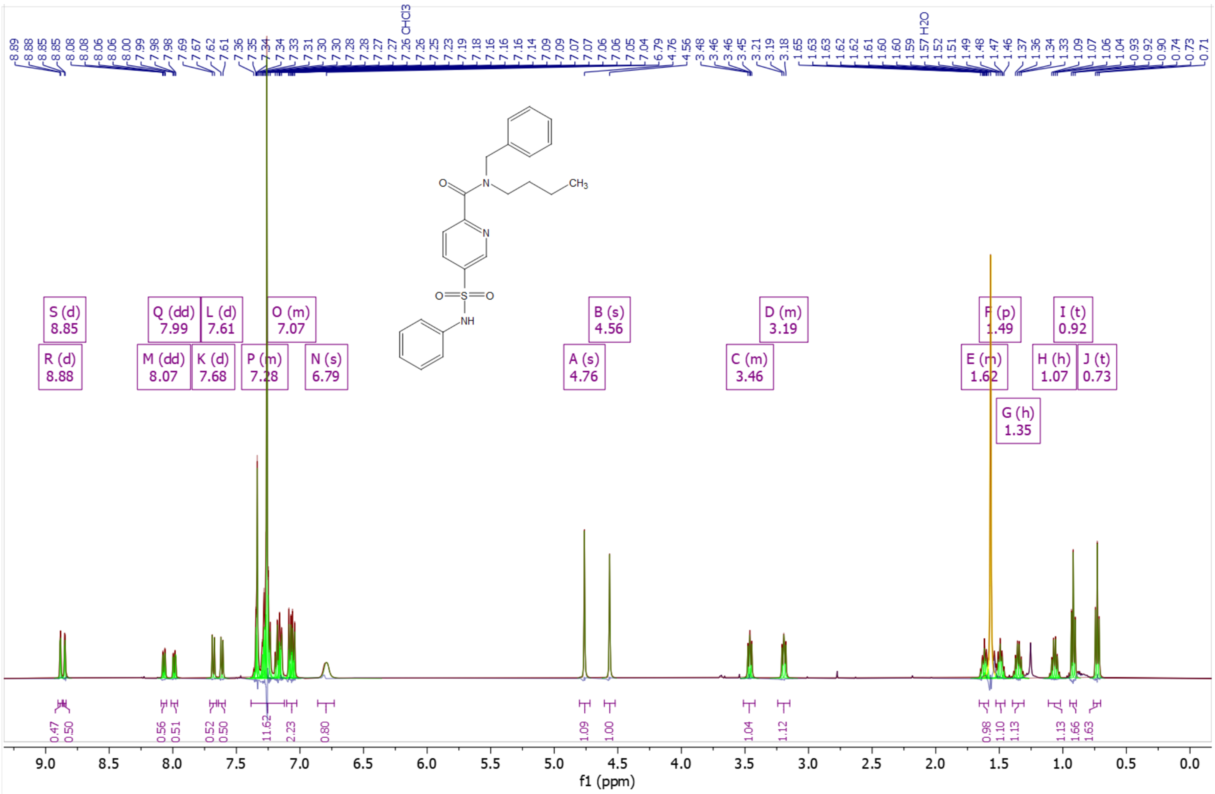

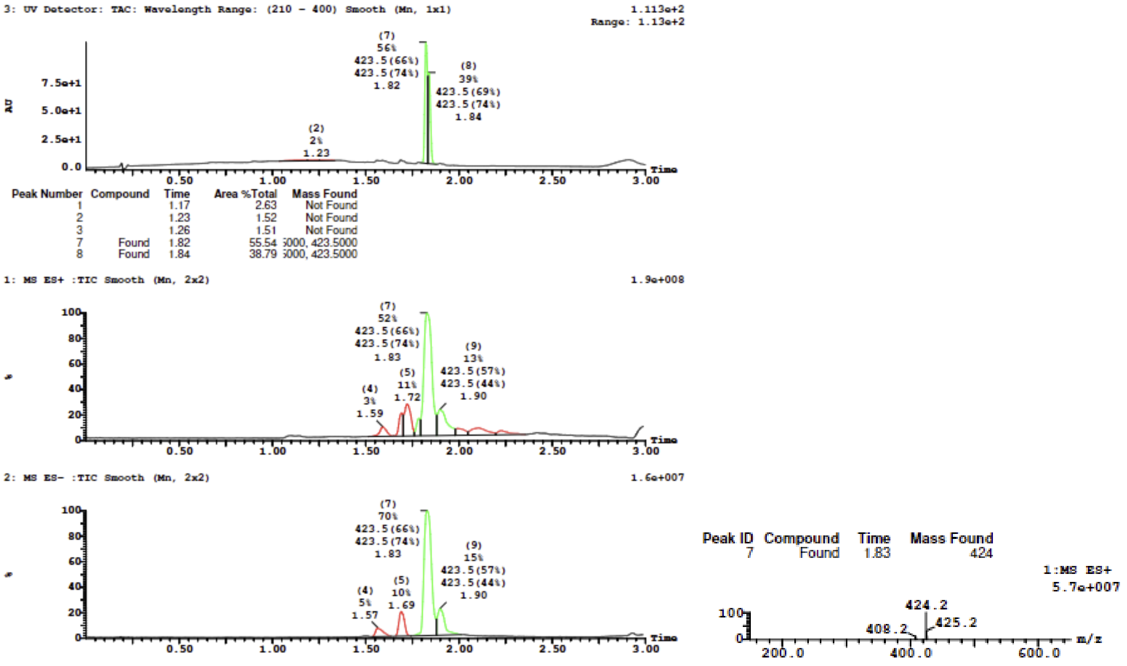

N-Benzyl-*N*-butyl-2-(phenylsulfamoyl)pyrimidine-5-carboxamide (**24**)

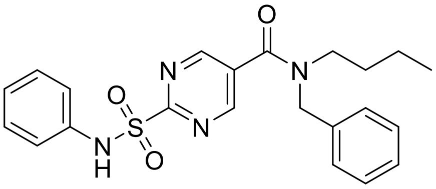

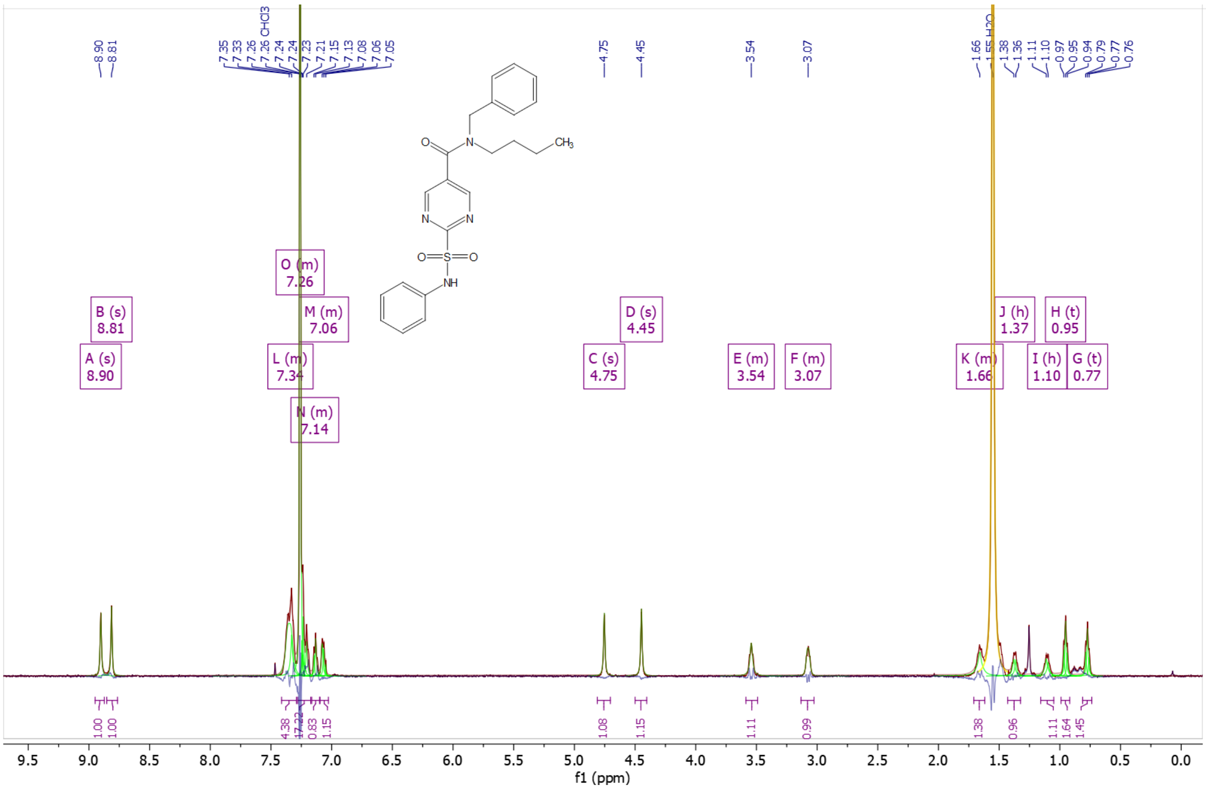

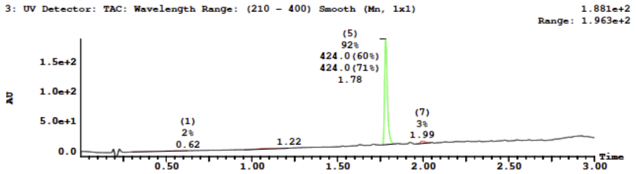

Peak Number	Compound	Time	Area %Total	Mass Found
1		0.62	1.94	Not Found
2		1.22	2.63	Not Found
5	Found	1.78	91.97	)000, 424.0000
7		1.99	3.46	Not Found

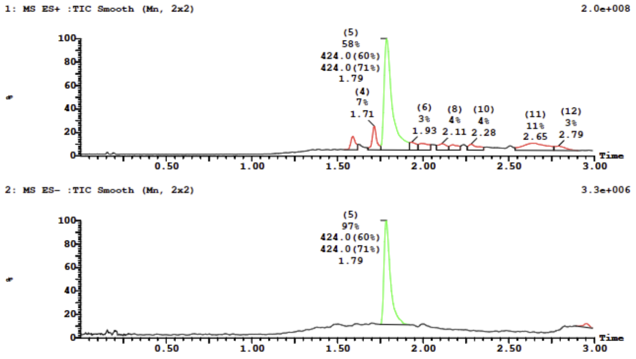

Peak ID	Compound	Time	Mass Found
5	Found	1.79	425

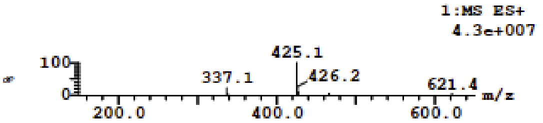

N-Benzyl-4-benzylsulfonyl-*N*-butyl-benzamide (**29**)

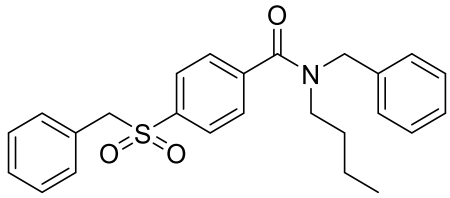

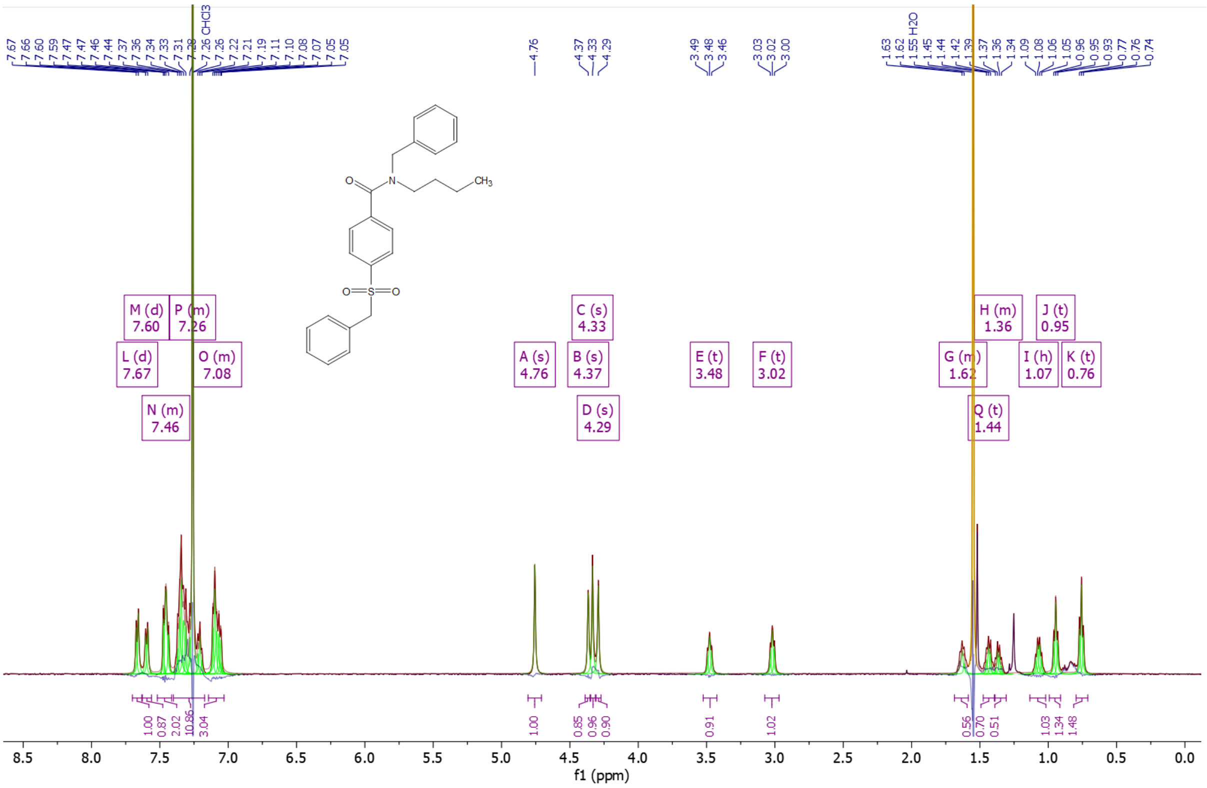

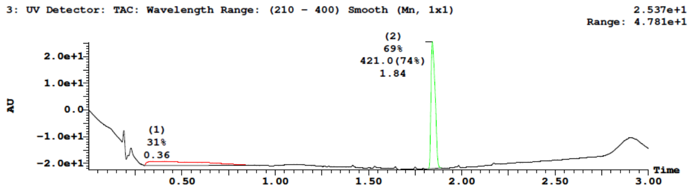

Peak Number	Compound	Time	Area %Total	Mass Found
1		0.36	31.01	Not Found
2	Found	1.84	68.99	421.0000

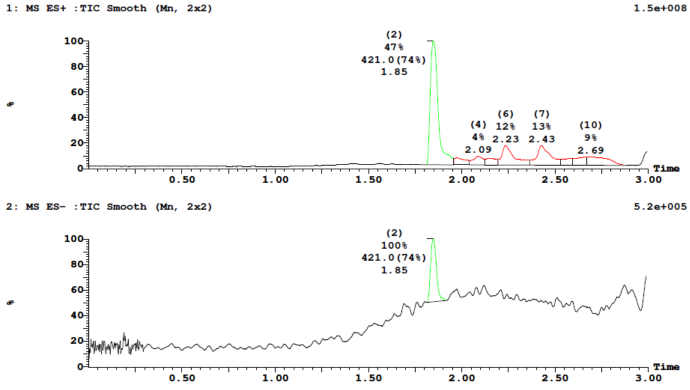

Peak ID	Compound	Time	Mass Found
2	Found	1.85	422

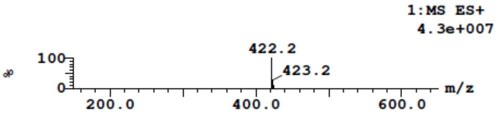

N-Benzyl-*N*-butyl-5-(phenylsulfamoyl)furan-3-carboxamide (**30**)

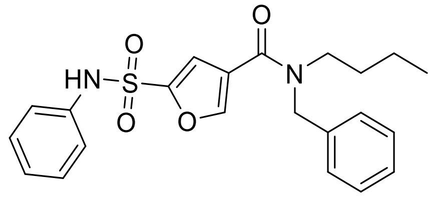

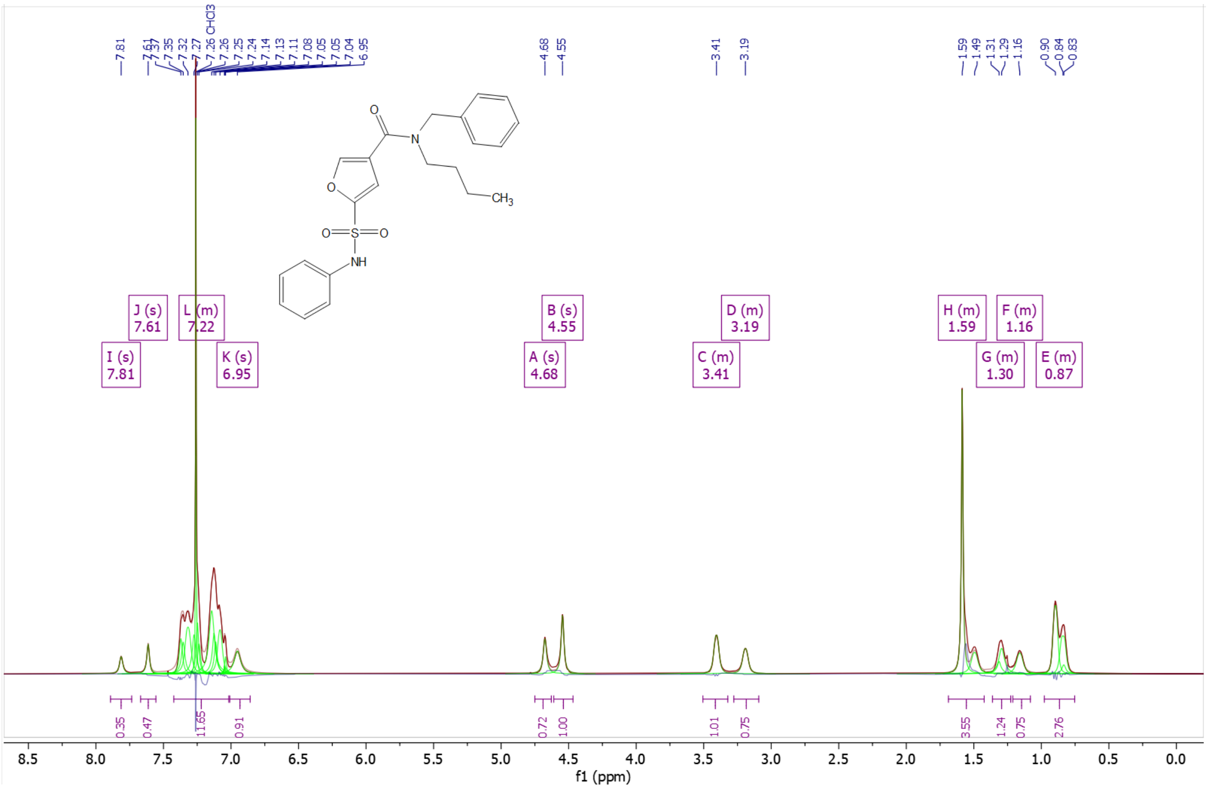

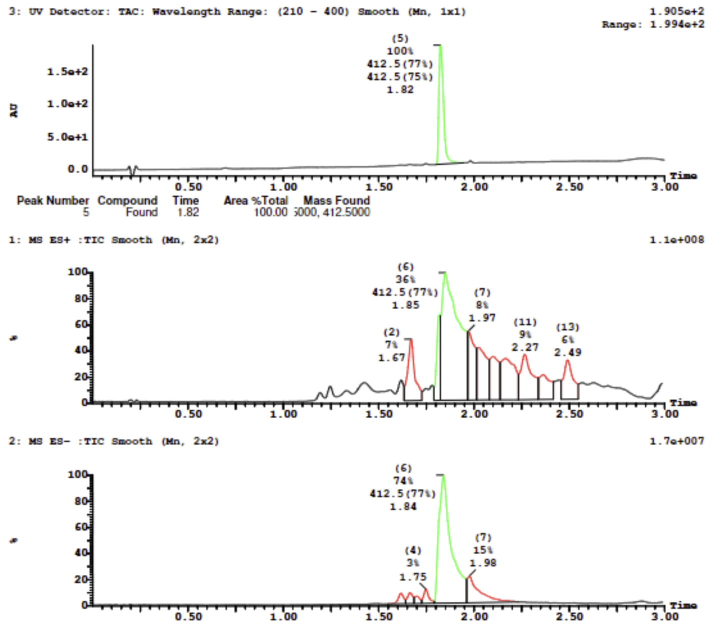

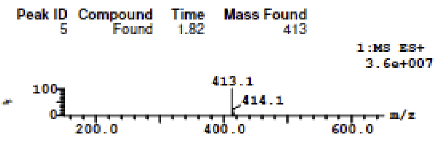

N-Benzyl-*N*-butyl-4-(*N*-methylsulfamoyl)benzamide (**34**)

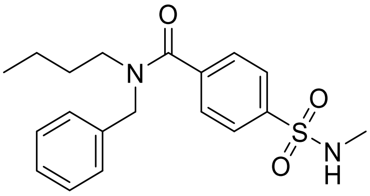

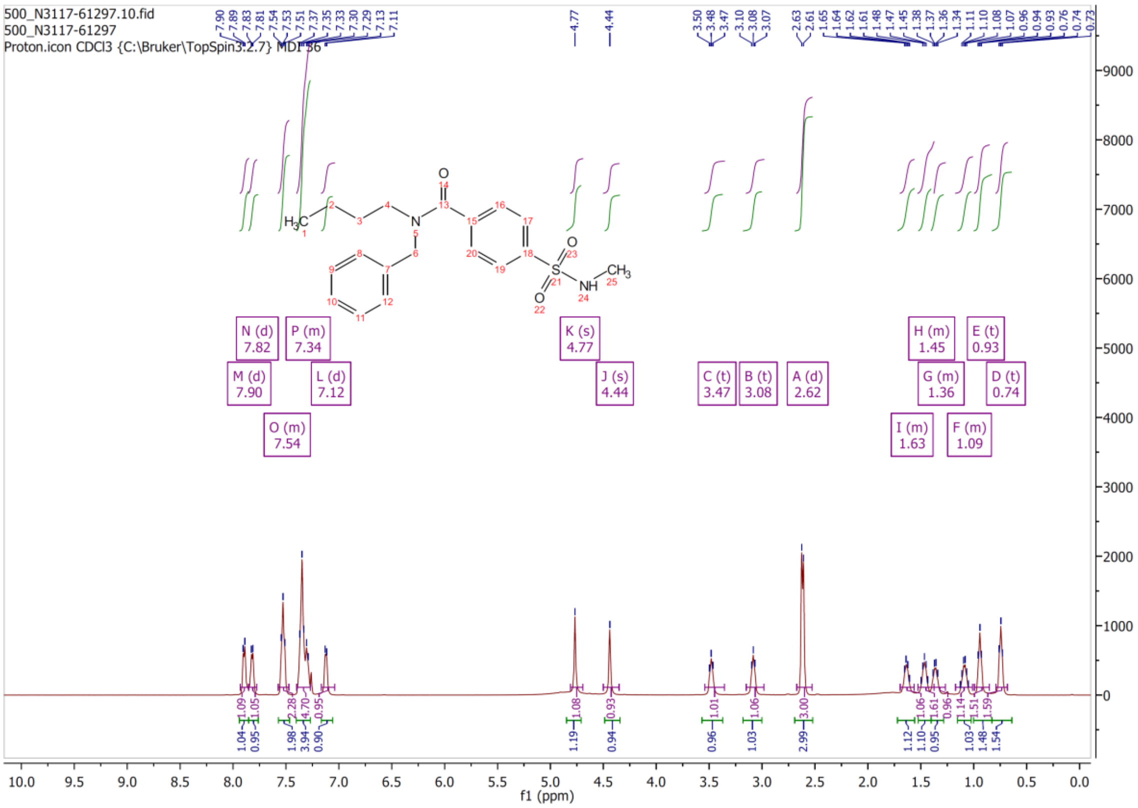

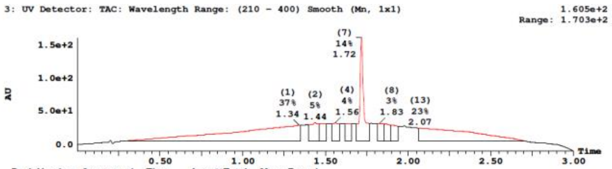

Peak Number	Compound	Time	Area %Total	Mass Found
1		1.34	36.71	Not Found
2		1.44	5.20	Not Found
3		1.48	3.84	Not Found
4		1.56	4.08	Not Found
5		1.63	3.17	Not Found
7		1.72	13.89	Not Found
8		1.83	3.31	Not Found
10		1.87	3.19	Not Found
11		1.90	3.22	Not Found
13		2.07	23.38	Not Found

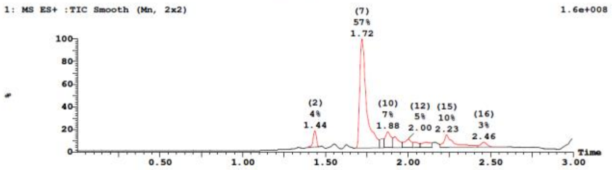

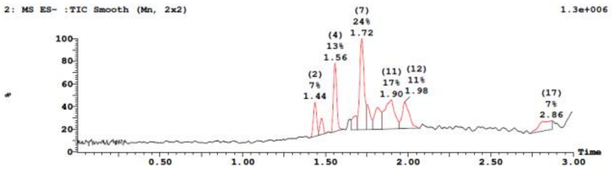

Peak ID	Compound	Time	Mass Found
7		1.72	Not Found

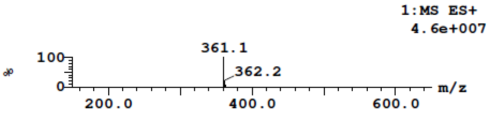

N-Benzyl-*N*-butyl-4-(*N*-(pyridin-4-yl)sulfamoyl)benzamide (**35**)

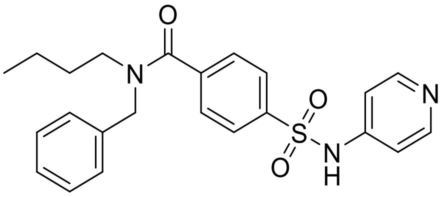

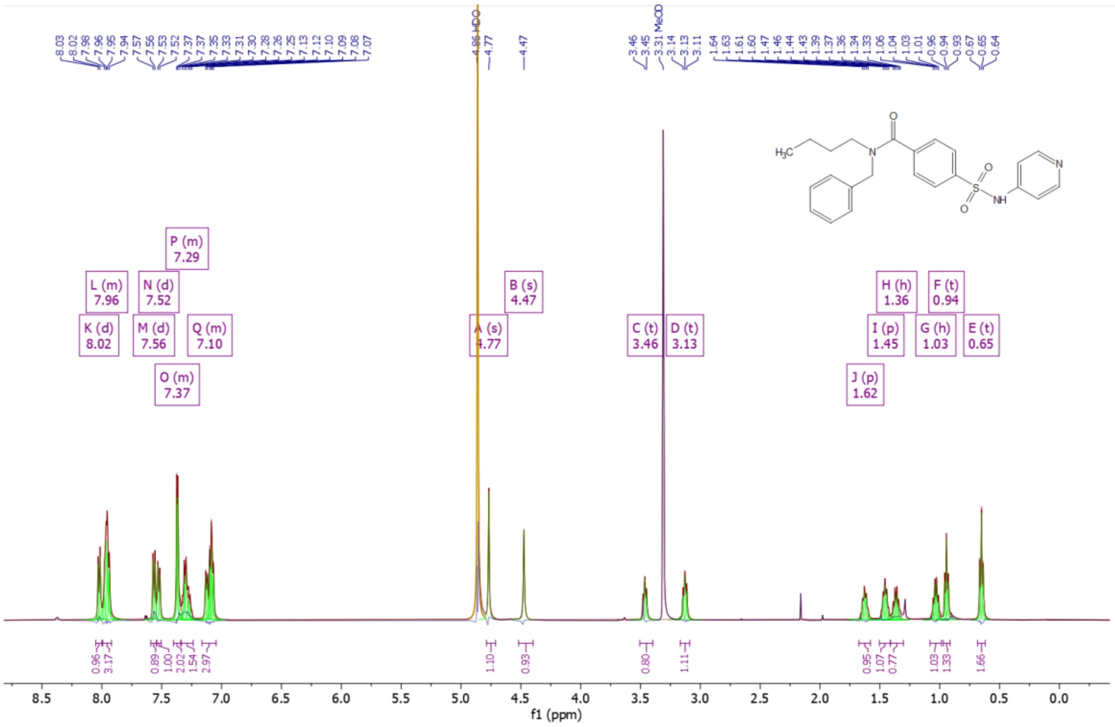

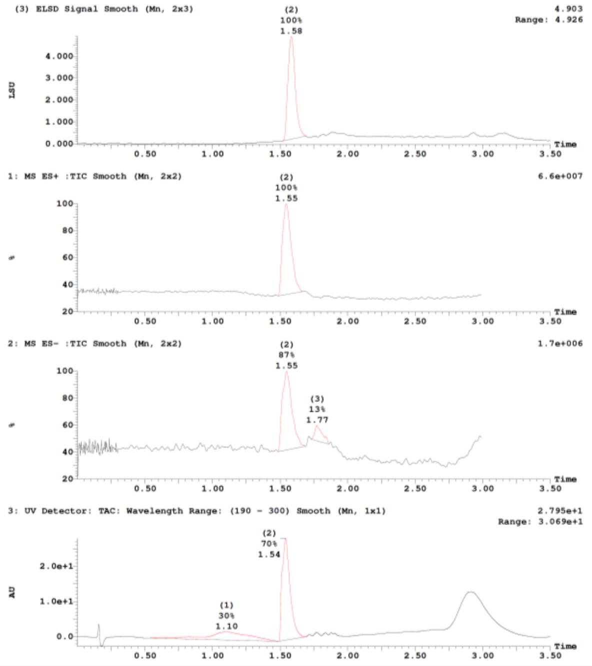

Peak ID	Compound	Time	Mass Found
2		1.55	Not Found

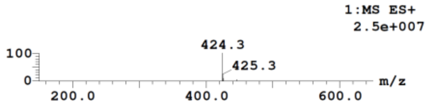

N-Benzyl-*N*-butyl-4-(isoxazol-4-ylsulfamoyl)benzamide (**36**)

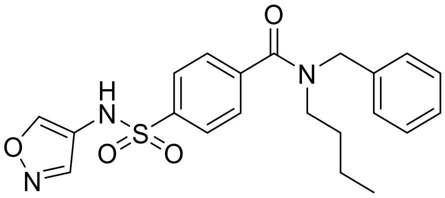

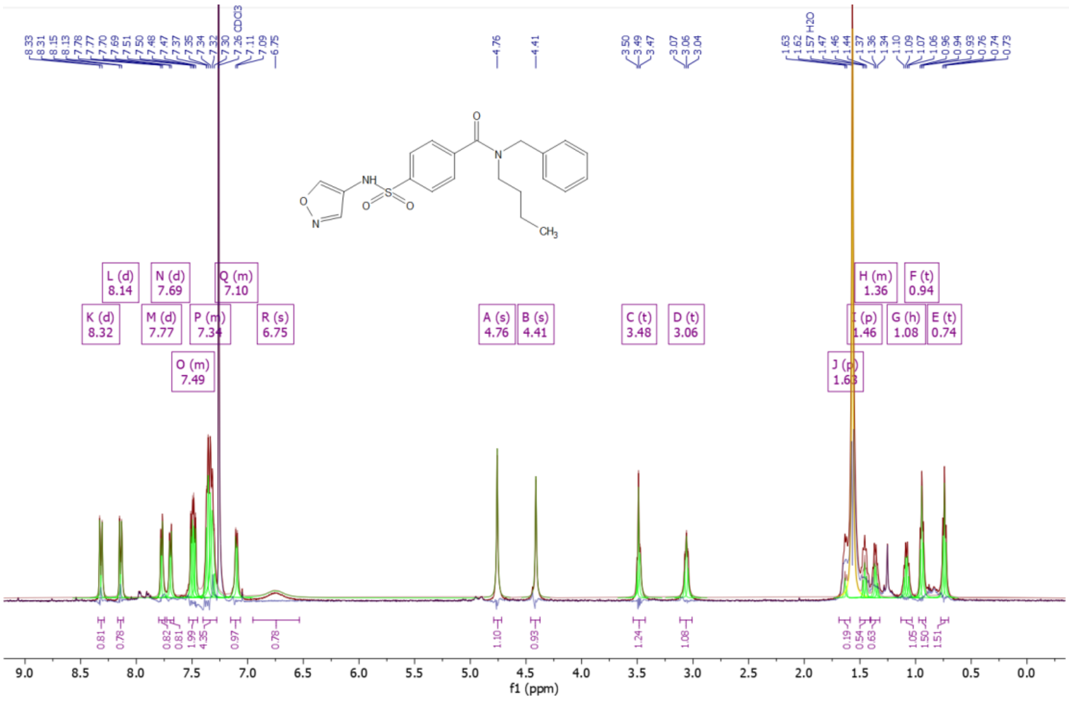

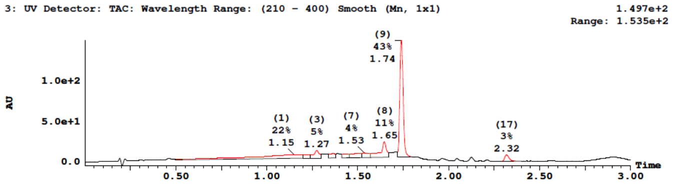

Peak Number	Compound	Time	Area %Total	Mass Found
1		1.15	21.76	Not Found
2		1.24	2.36	Not Found
3		1.27	4.74	Not Found
4		1.37	2.88	Not Found
5		1.43	2.68	Not Found
6		1.50	4.64	Not Found
7		1.53	3.55	Not Found
8		1.65	10.97	Not Found
9		1.74	43.23	Not Found
17		2.32	3.19	Not Found

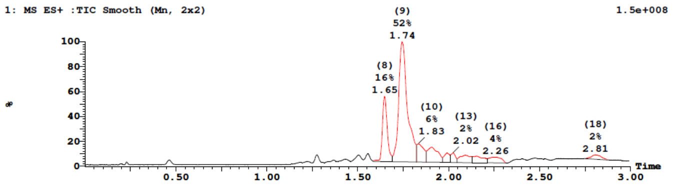

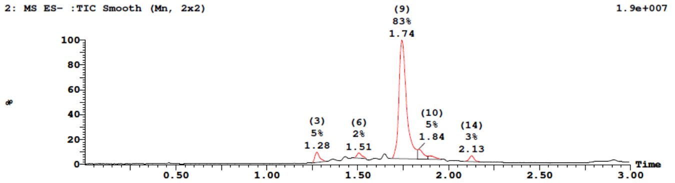

Peak ID	Compound	Time	Mass Found
9		1.74	Not Found

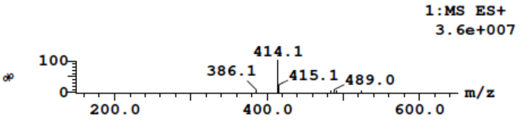

N-Benzyl-*N*-butyl-4-(*N*-cyclobutylsulfamoyl)benzamide (**37**)

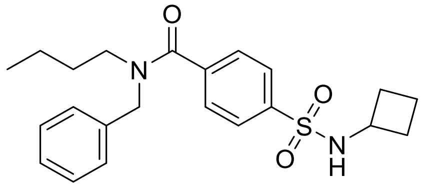

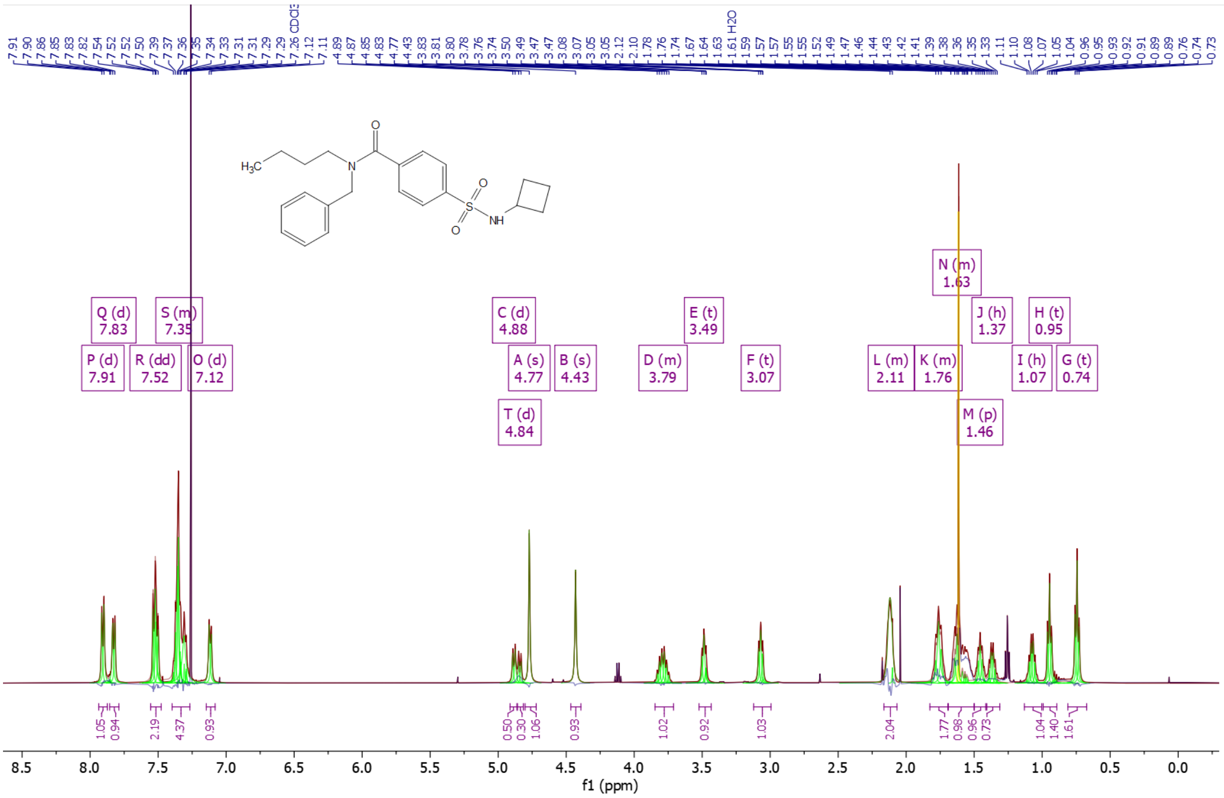

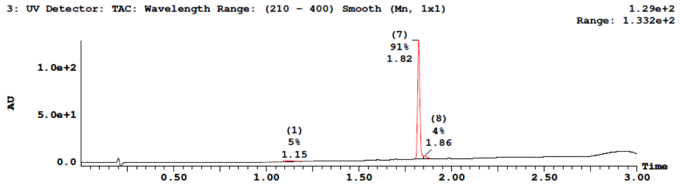

Peak Number	Compound	Time	Area %Total	Mass Found
1		1.15	4.59	Not Found
7		1.82	91.38	Not Found
8		1.86	4.02	Not Found

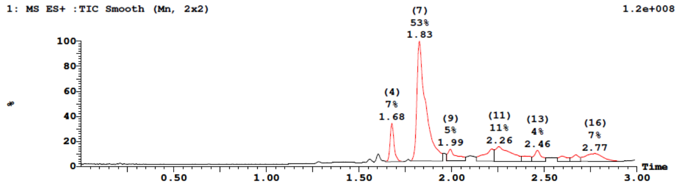

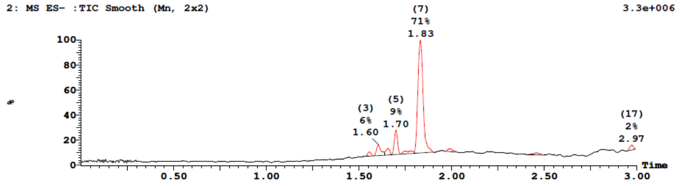

Peak ID	Compound	Time	Mass Found
7		1.83	Not Found

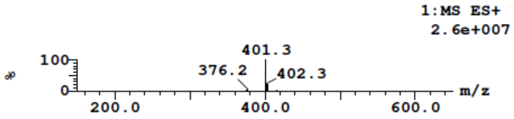

N-Benzyl-*N*-butyl-4-(*N*-(oxetan-3-yl)sulfamoyl)benzamide (**38**)

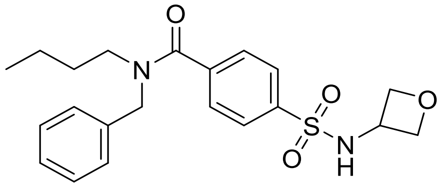

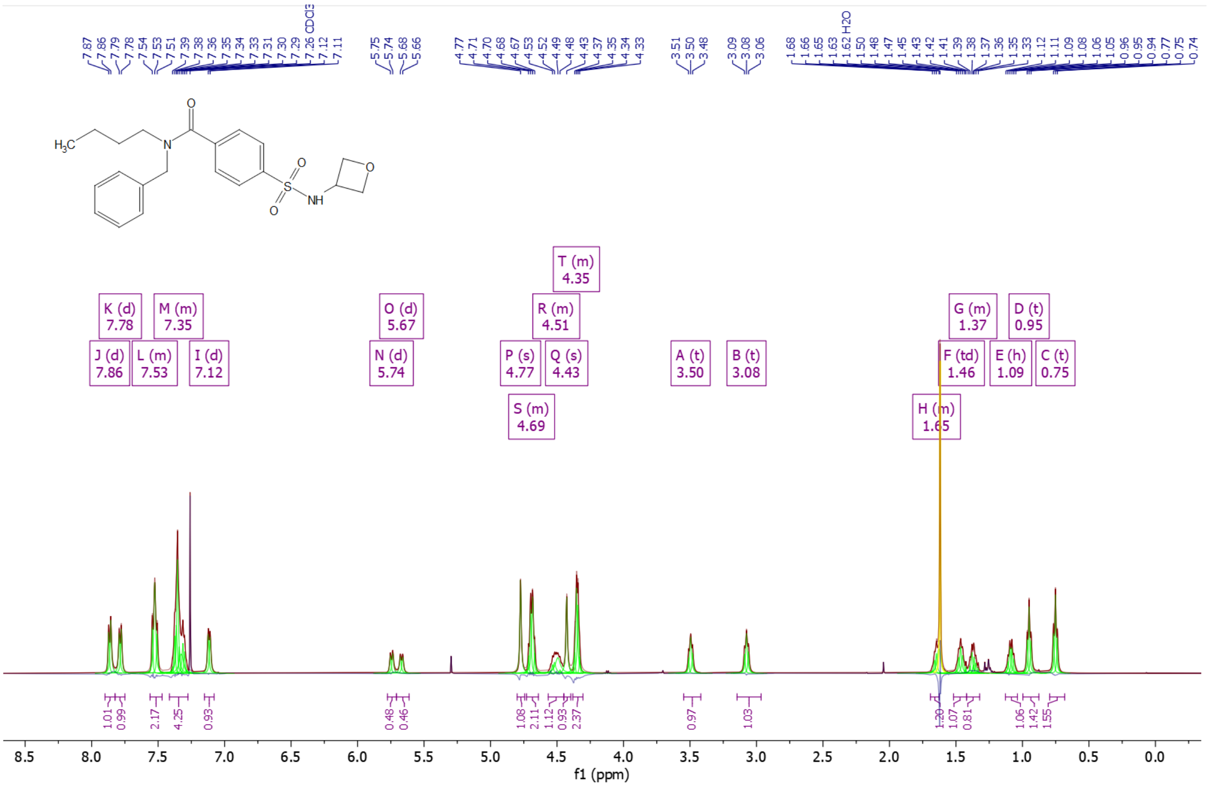

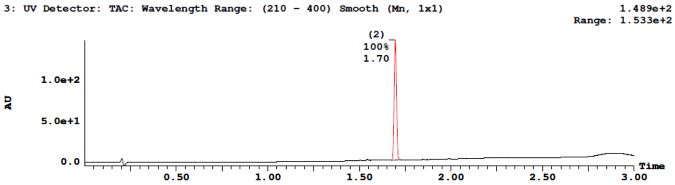

Peak Number	Compound	Time	Area %Total	Mass Found
2		1.70	100.00	Not Found

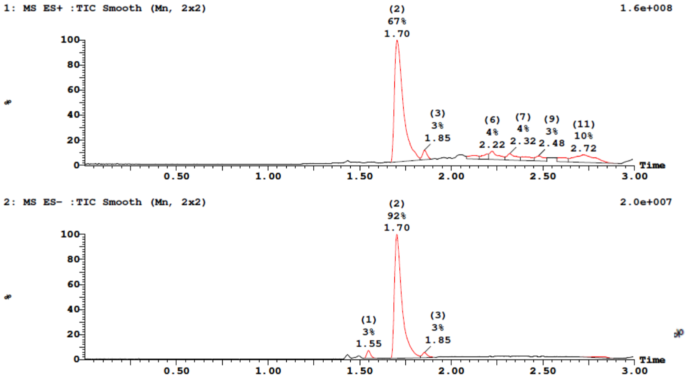

Peak ID	Compound	Time	Mass Found
2		1.70	Not Found

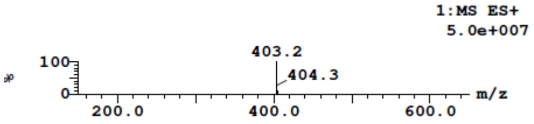

N-Butyl-*N*-(4-fluorobenzyl)-4-(*N*-phenylsulfamoyl)benzamide (**39**)

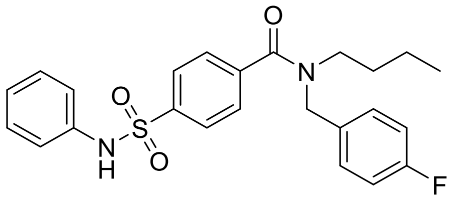

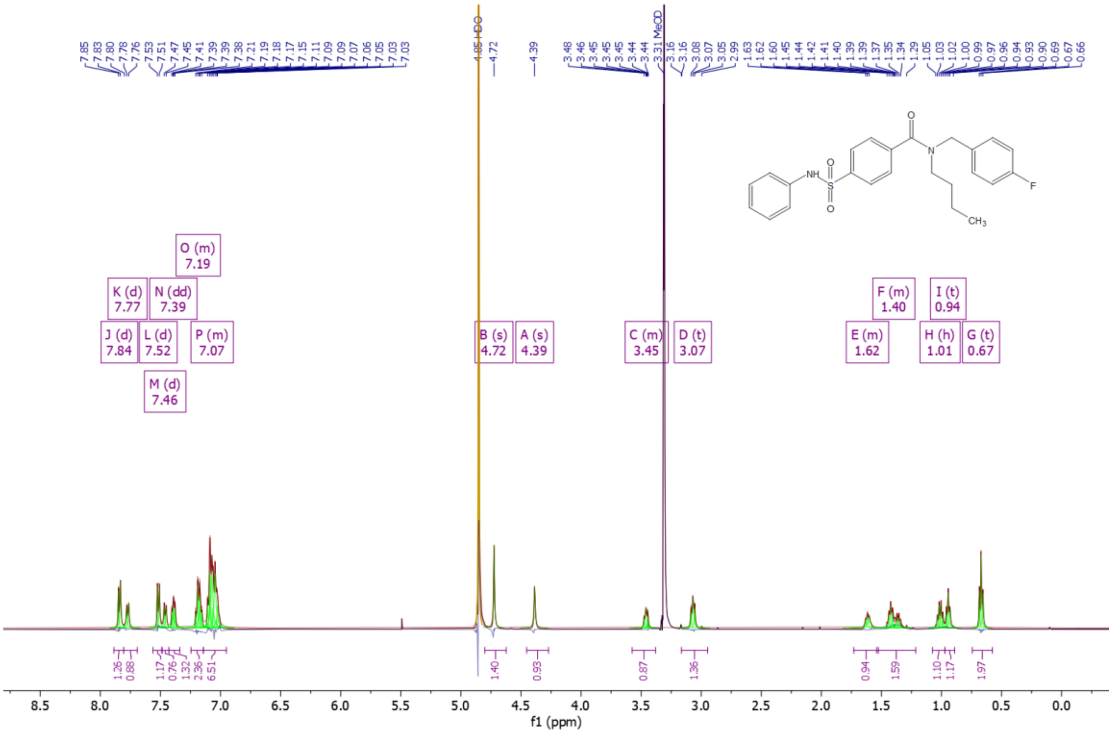

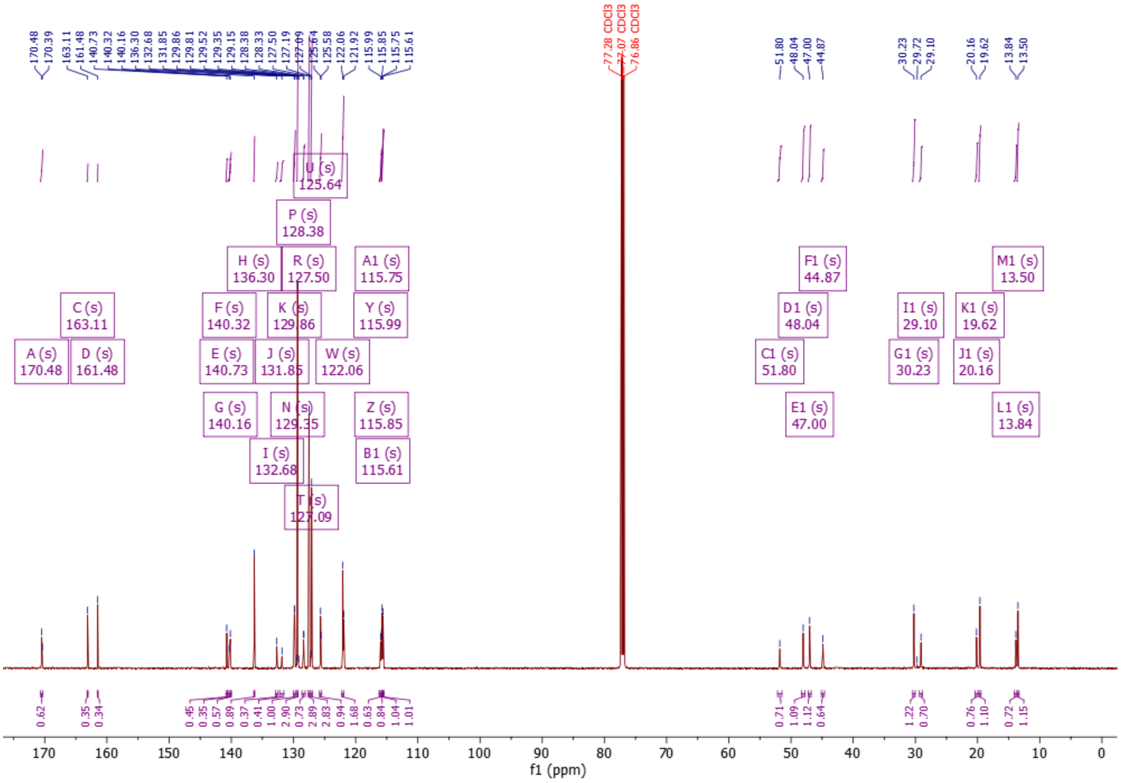

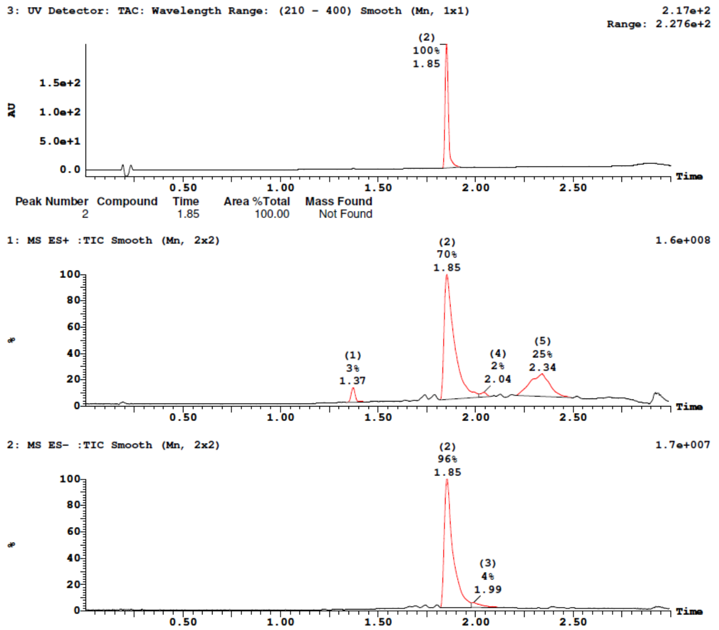

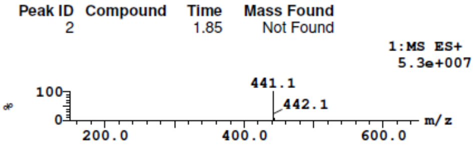

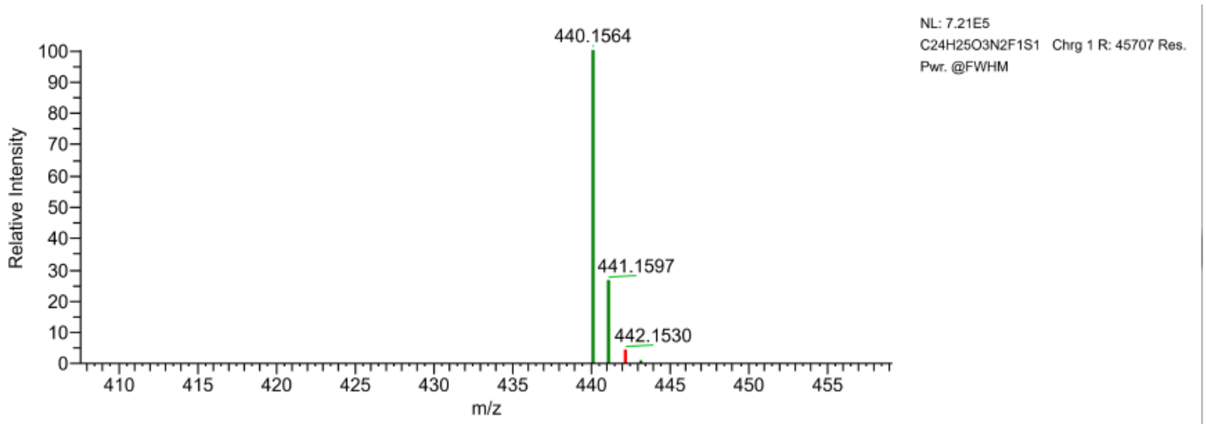

Peak Mass	Display Formula	Delta [ppm]	Theo. mass	Combined Score	MSMS Matched Frag...
440.1559	$C_{24}H_{25}O_3N_2F^{32}S$	-1.34	440.15644	90.7644958867867	(Collection)

N-Butyl-*N*-(4-methoxybenzyl)-4-(*N*-phenylsulfamoyl)benzamide (**40**)

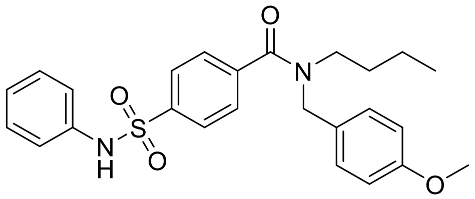

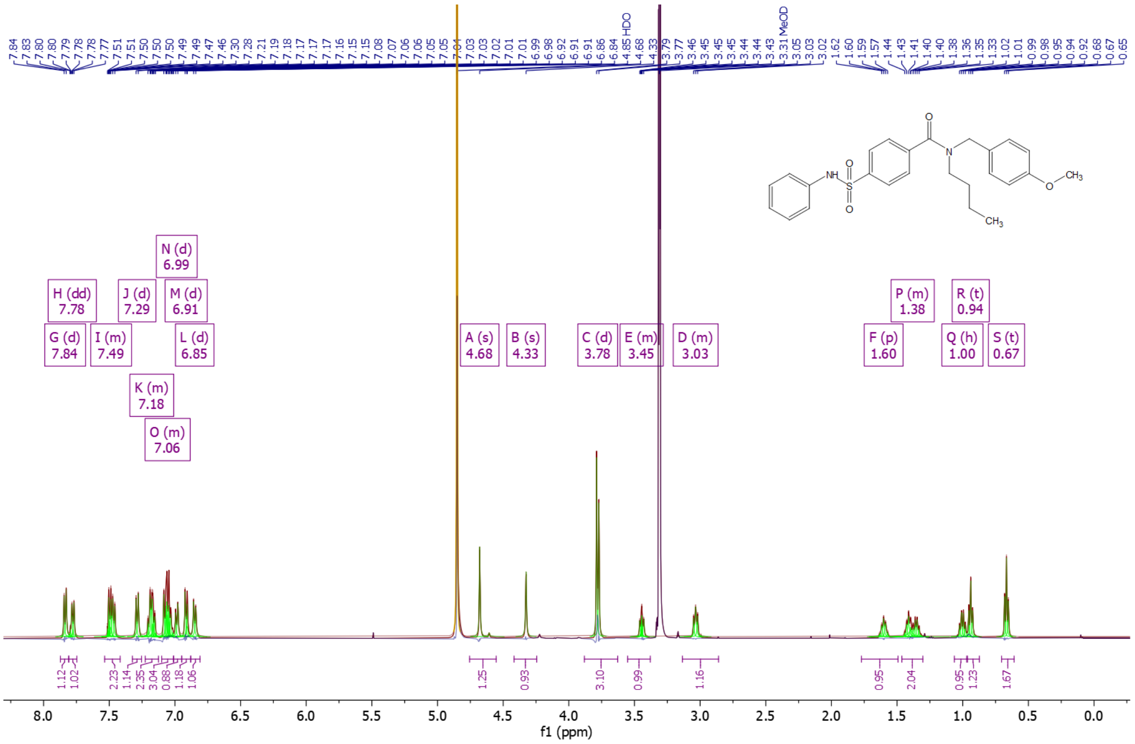

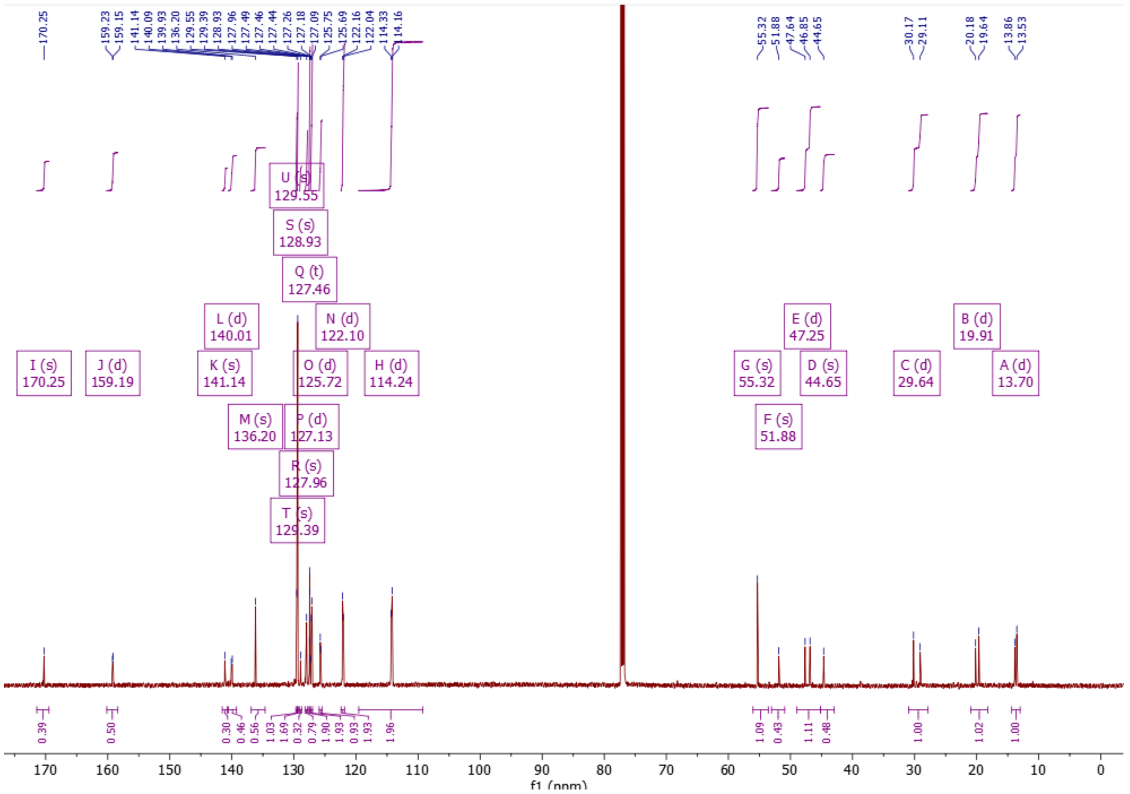

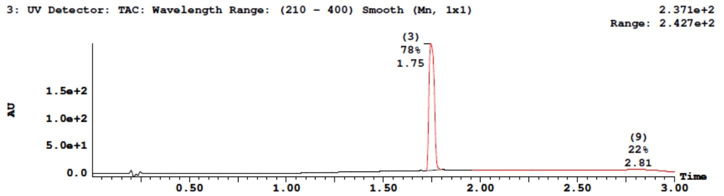

Peak Number	Compound	Time	Area %Total	Mass Found
3		1.75	77.99	Not Found
9		2.81	22.01	Not Found

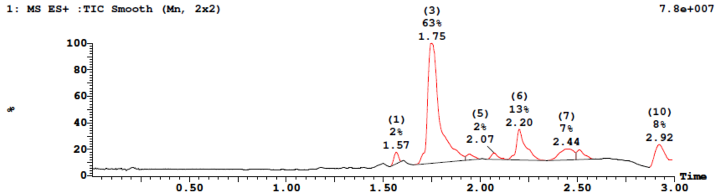

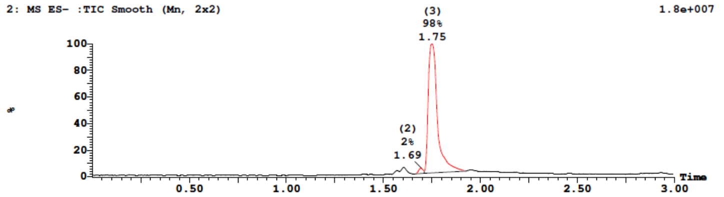

Peak ID	Compound	Time	Mass Found
3		1.75	Not Found

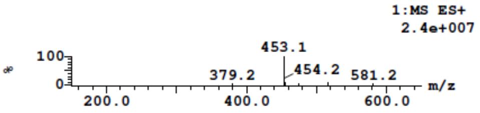

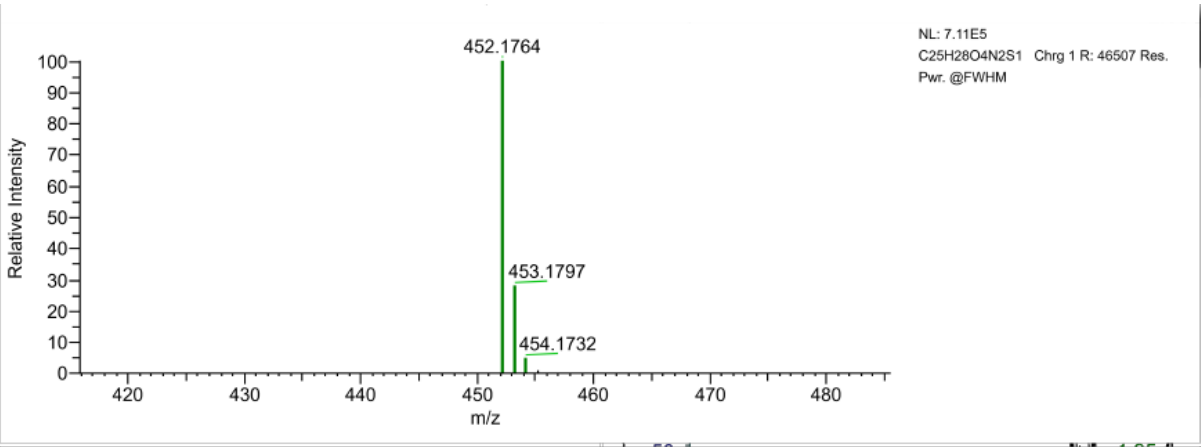

Peak Mass	Display Formula	Delta [ppm]	Theo. mass	Combined Score	MSMS Matched Frag...
452.1762	$C_{25}H_{28}O_4N_2{}^{32}S$	-0.49	452.17643	86.0937178109365	(Collection)

N-Butyl-*N*-(3-methoxybenzyl)-4-(*N*-phenylsulfamoyl)benzamide (**41**)

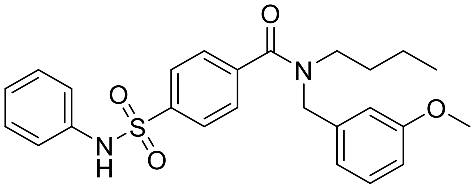

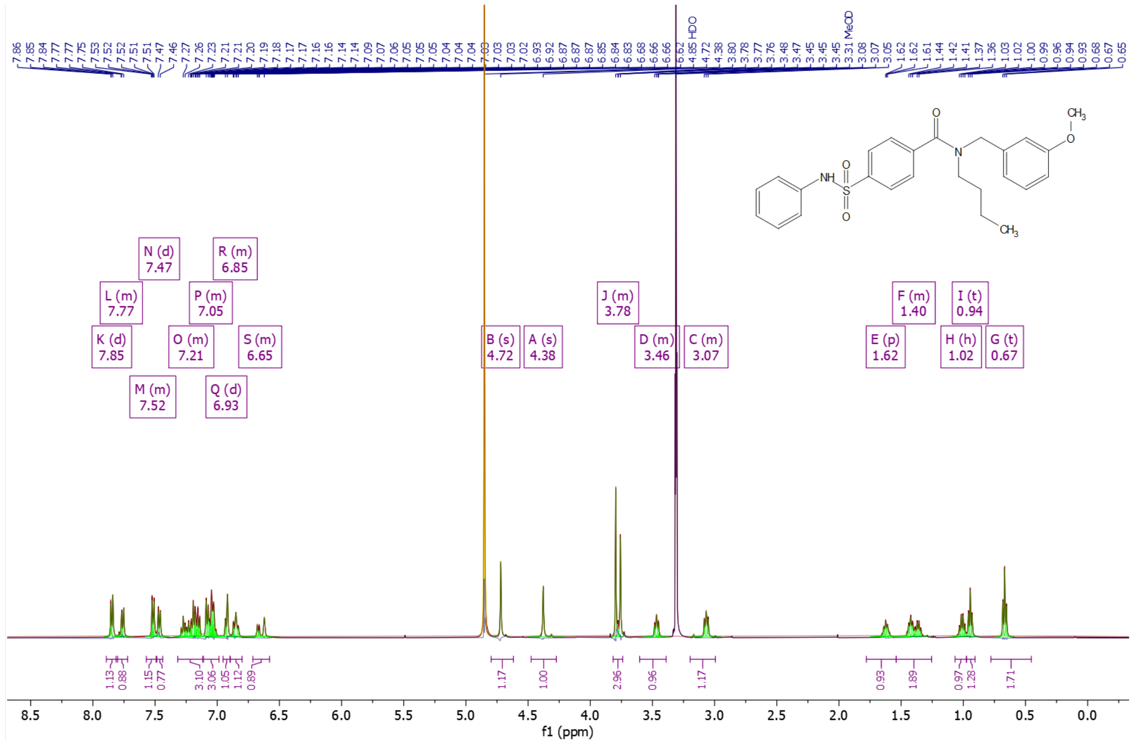

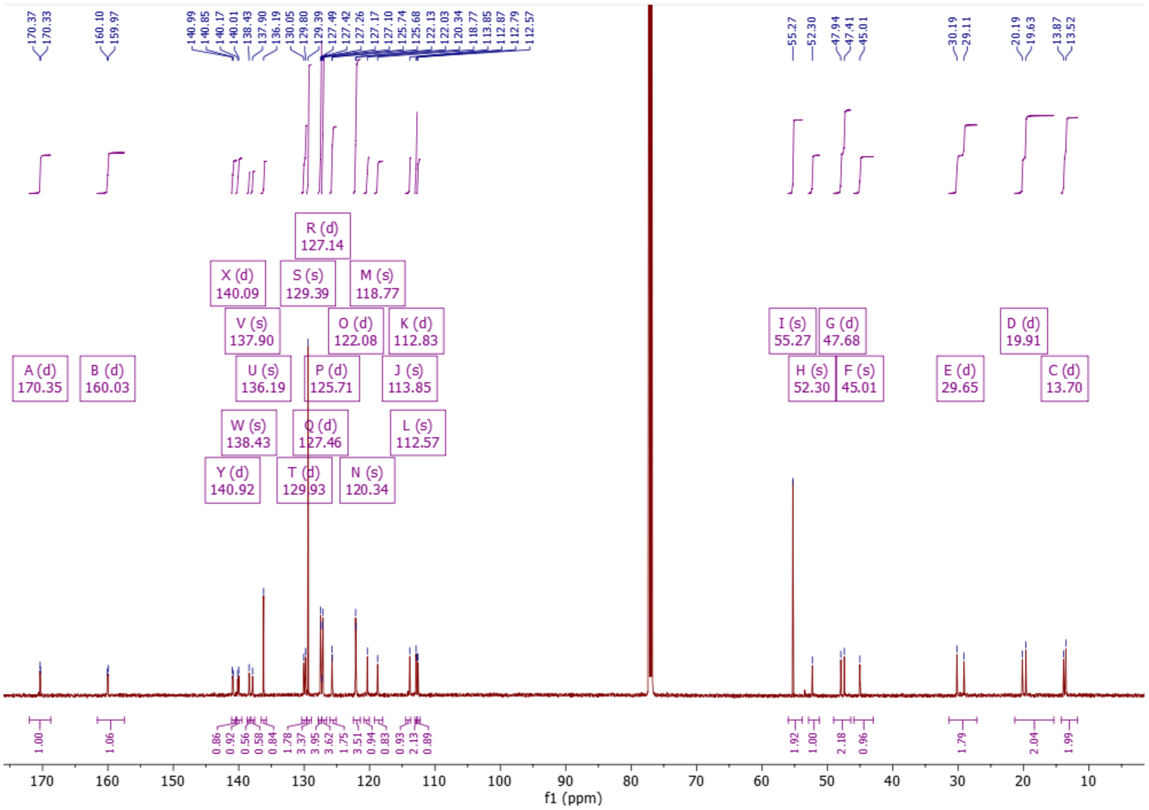

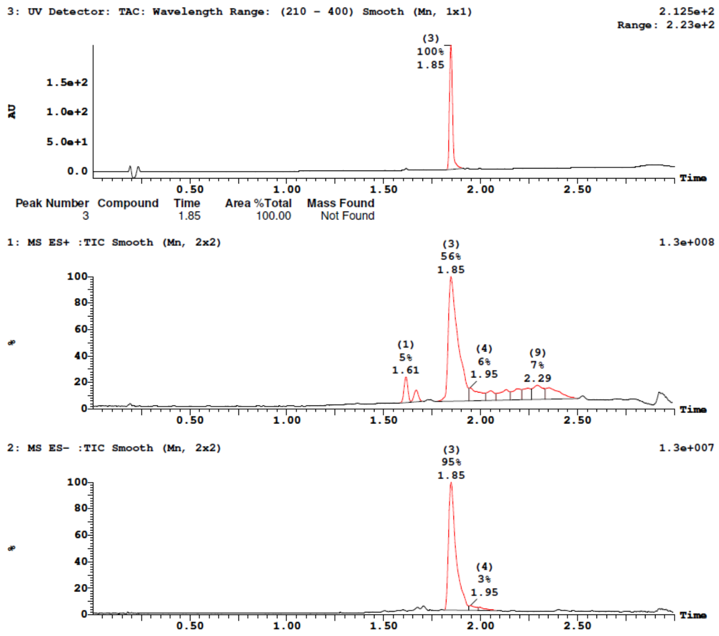

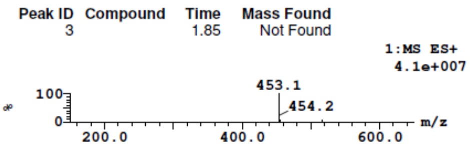

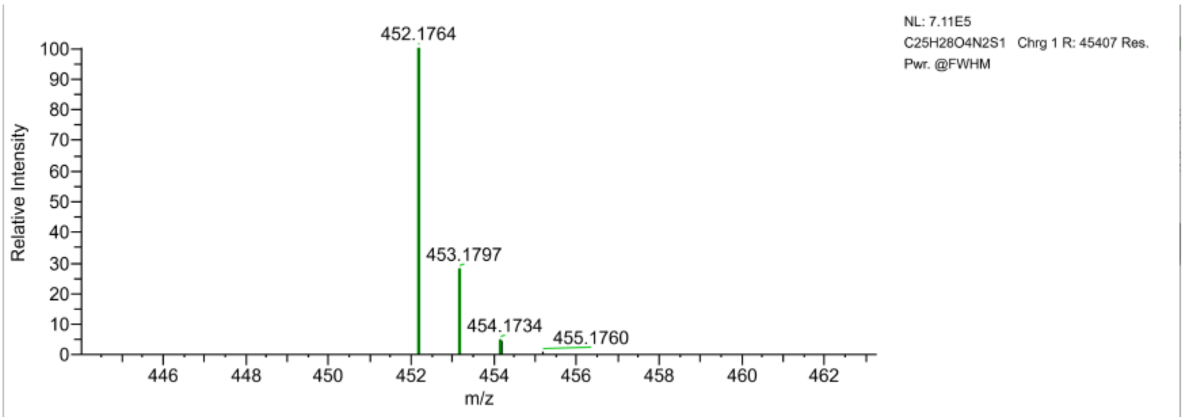

Peak Mass	Display Formula	Delta [ppm]	Theo. mass	Combined Score	MSMS Matched Frag...
452.1760	$C_{25}H_{28}O_4N_2{}^{32}S$	-0.89	452.17643	87.4338338424178	(Collection)

N-Butyl-*N*-(2-methoxybenzyl)-4-(*N*-phenylsulfamoyl)benzamide (**42**)

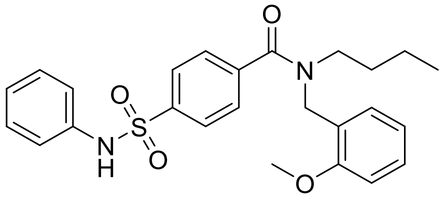

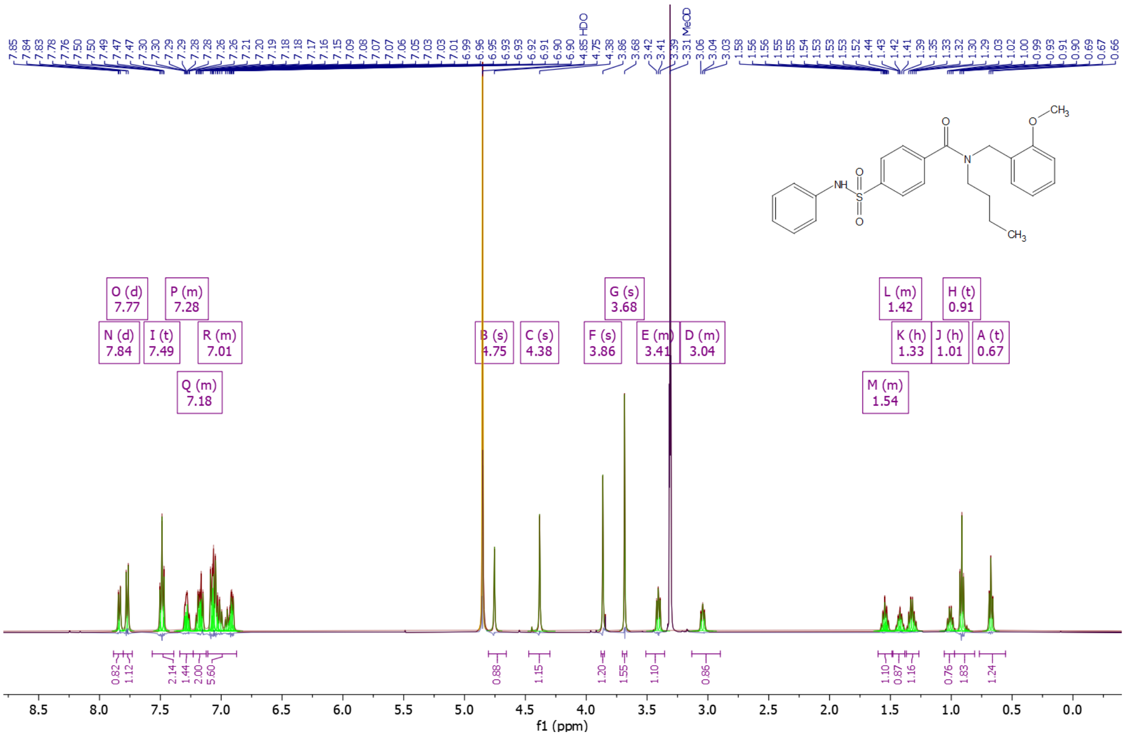

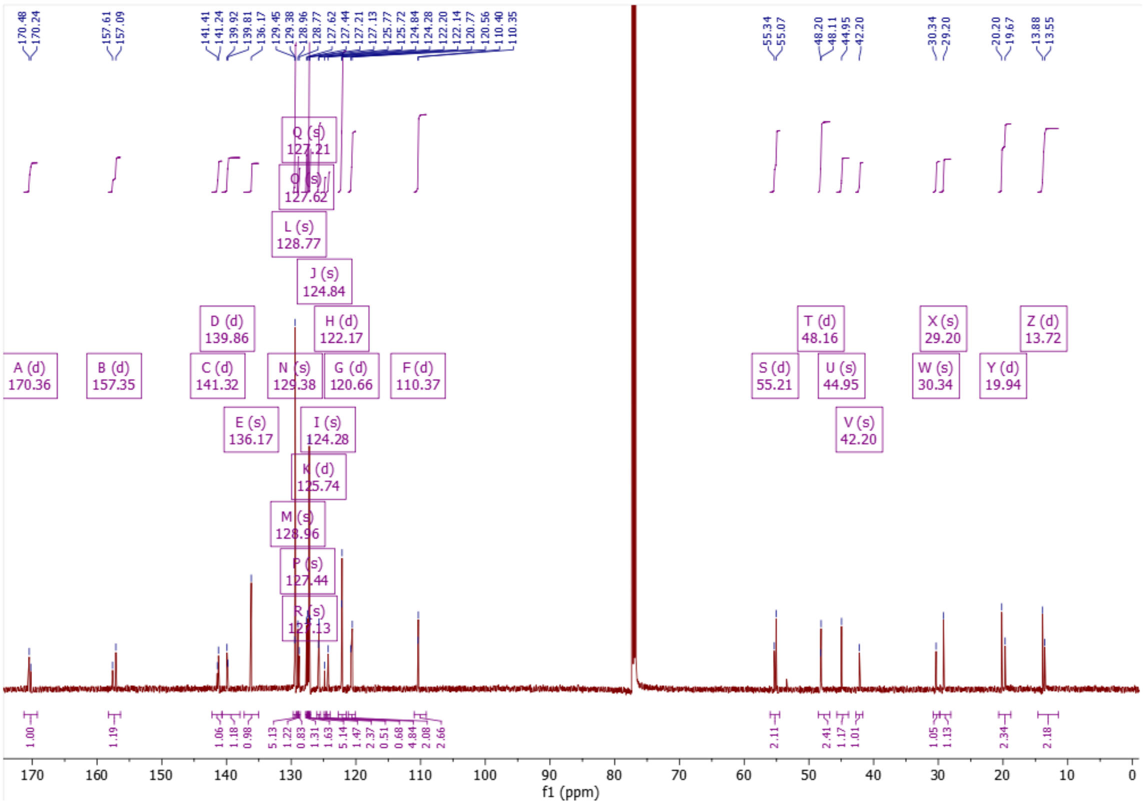

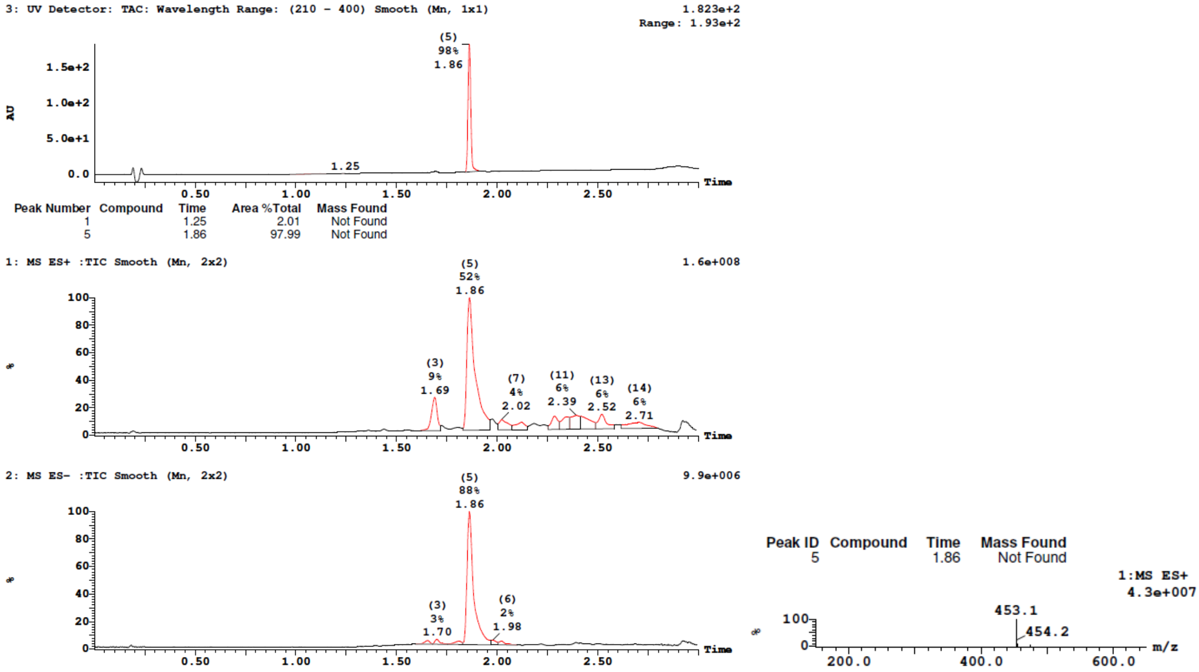

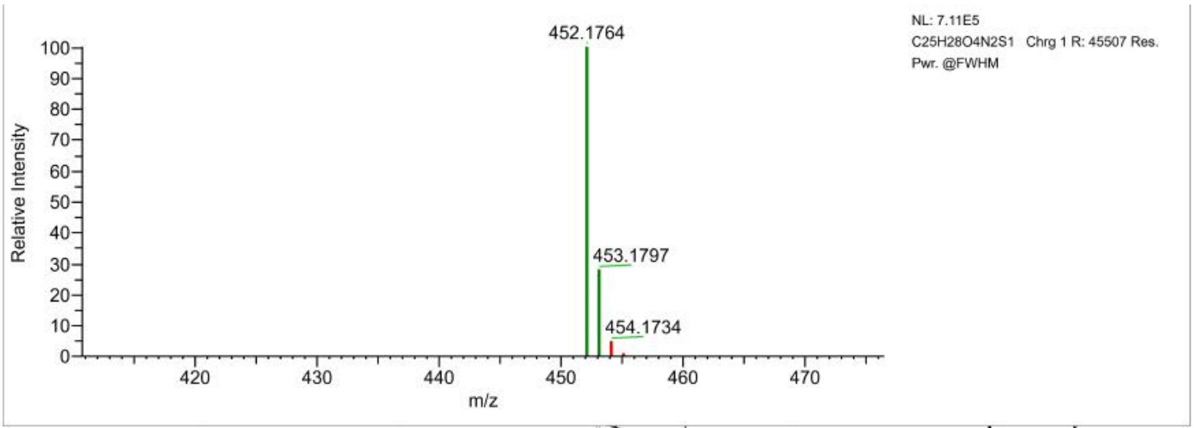

Peak Mass	Display Formula	Delta [ppm]	Theo. mass	Combined Score	MSMS Matched Frag...
452.1766	$C_{25}H_{28}O_4N_2{}^{32}S$	0.46	452.17643	92.3064208172601	(Collection)

N-Butyl-4-(*N*-phenylsulfamoyl)-*N*-(pyridin-4-ylmethyl)benzamide (**43**)

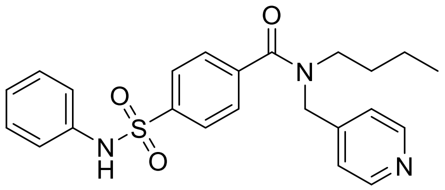

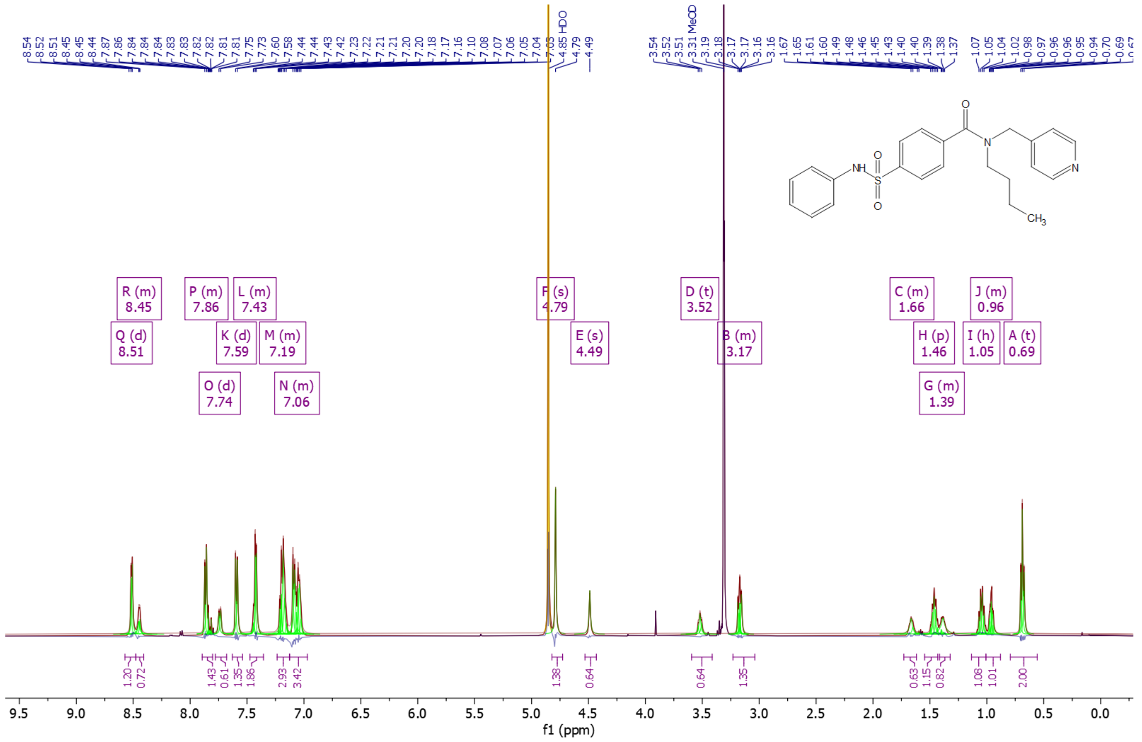

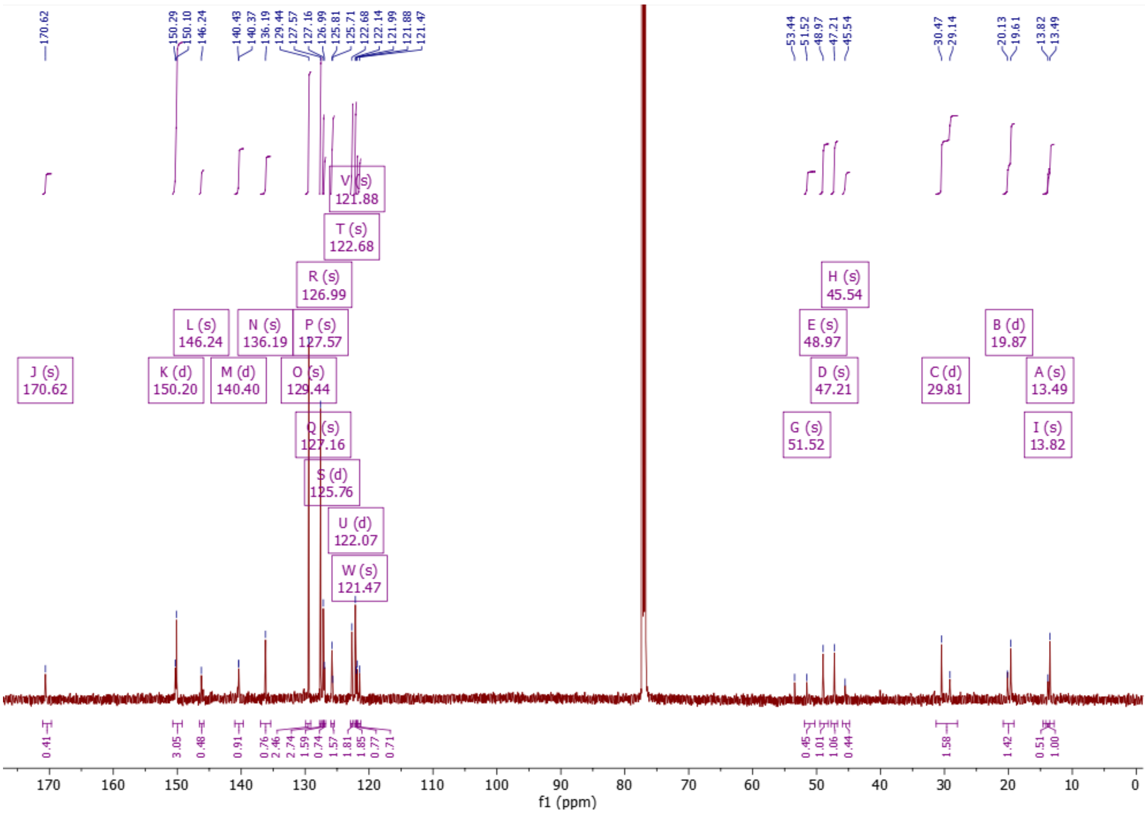

3: UV Detector: TAC: Wavelength Range: (210 - 400) Smooth (Mn, 1x1)

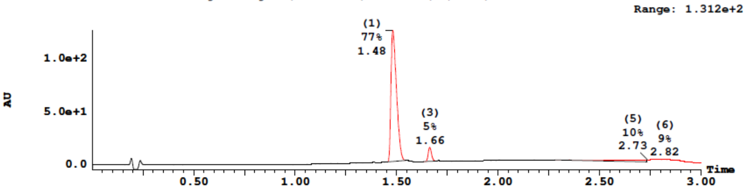

Peak Number	Compound	Time	Area %Total	Mass Found
1		1.48	76.58	Not Found
3		1.66	4.90	Not Found
5		2.73	9.51	Not Found
6		2.82	9.01	Not Found

1: MS ES+ :TIC Smooth (Mn, 2x2)

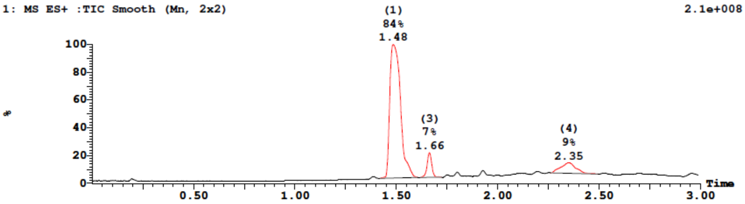

2: MS ES- :TIC Smooth (Mn, 2x2)

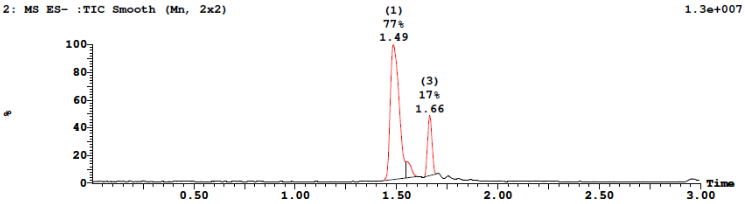

Peak ID	Compound	Time	Mass Found
1		1.48	Not Found

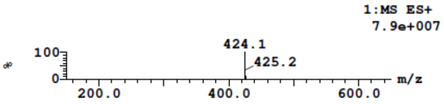

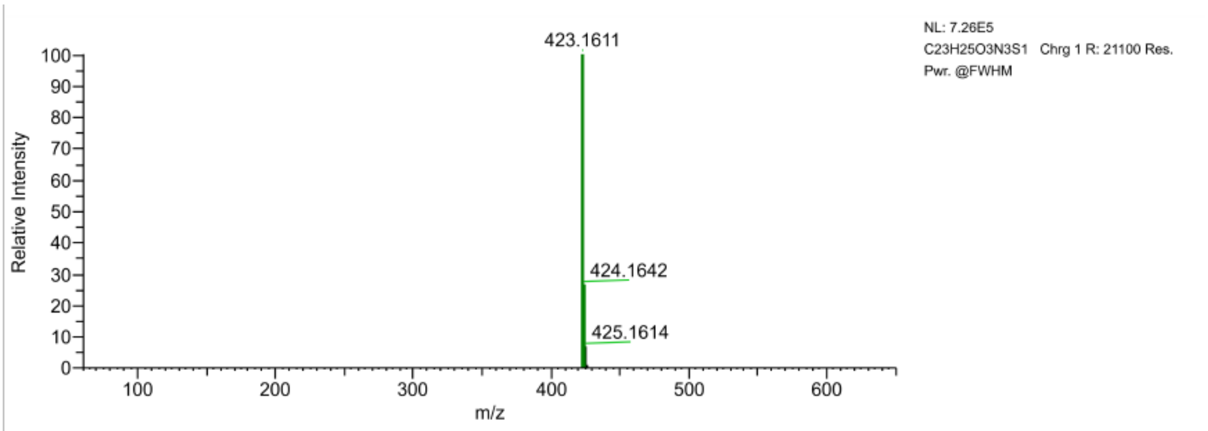

Peak Mass	Display Formula	Delta [ppm]	Theo. mass	Combined Score	MS Cov. [%]	MSMS Matched Fr...
423.1617	$C_{23}H_{25}O_3N_3{}^{32}S$	1.49	423.16111	40.54142599318...	41.82415171235...	(Collection)

N-Butyl-4-(*N*-phenylsulfamoyl)-*N*-(pyridin-3-ylmethyl)benzamide (**44**)

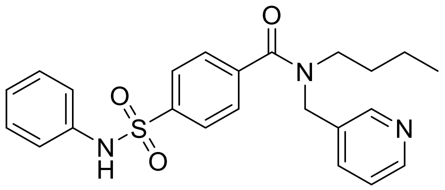

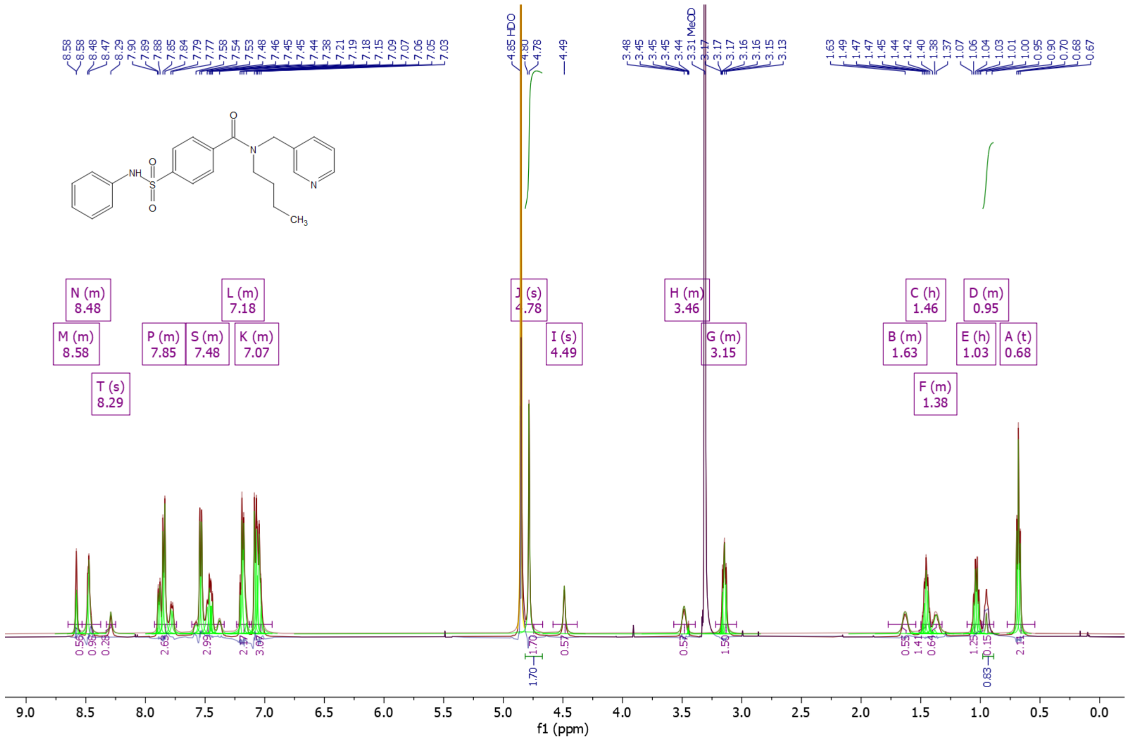

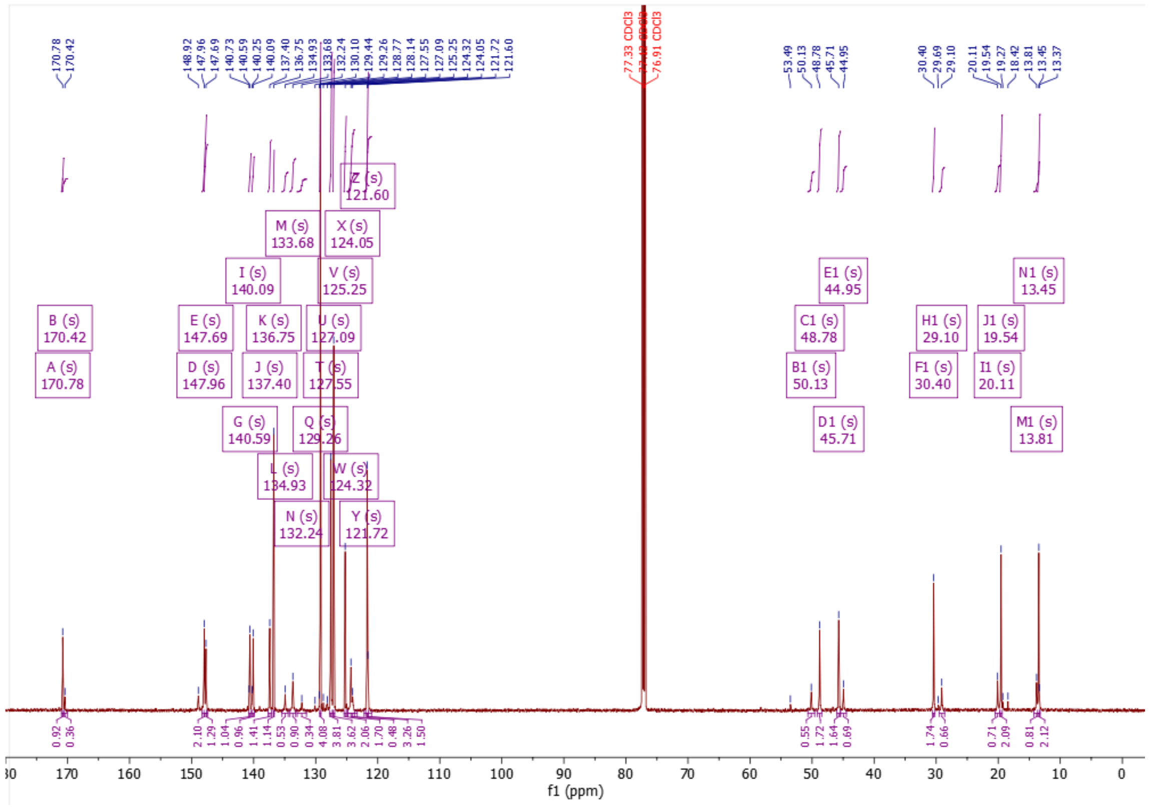

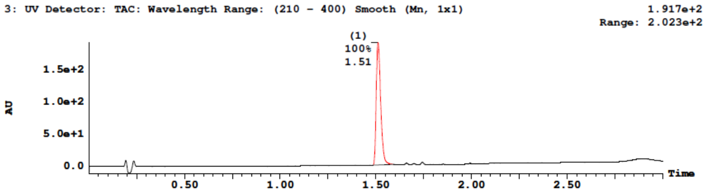

Peak Number	Compound	Time	Area %Total	Mass Found
1		1.51	100.00	Not Found

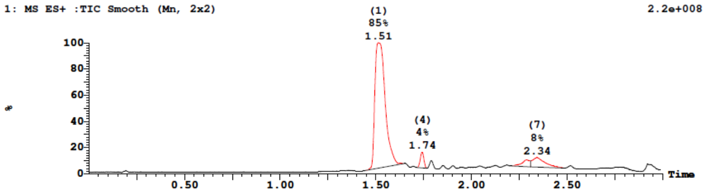

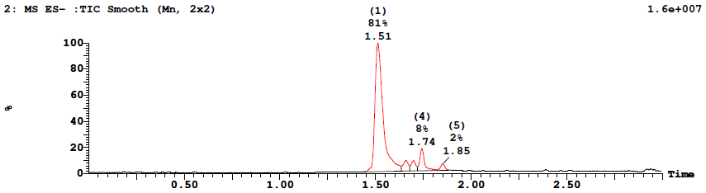

Peak ID	Compound	Time	Mass Found
1		1.51	Not Found

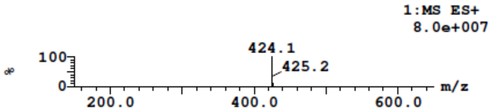

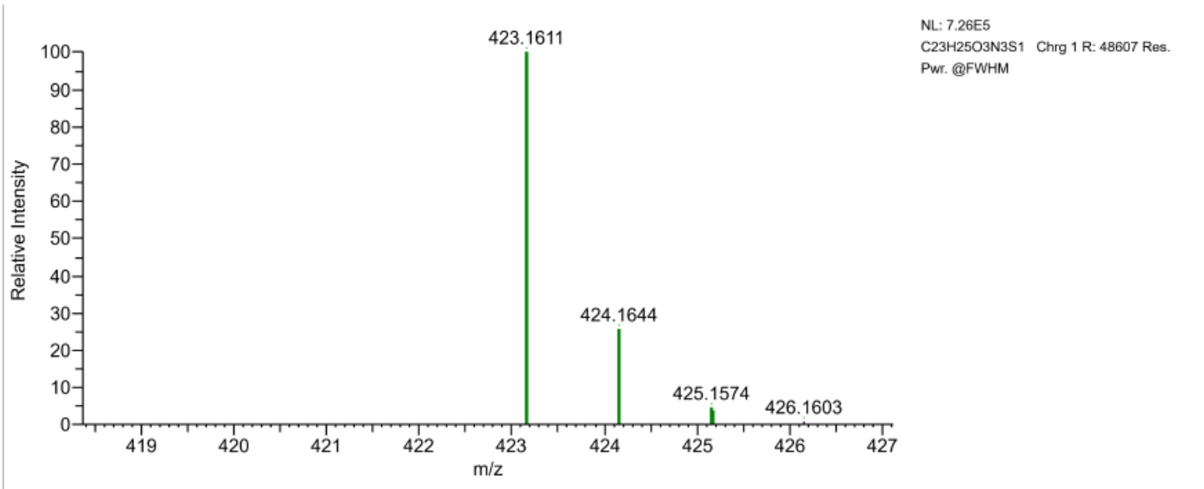

Peak Mass	Display Formula	Delta [ppm]	Theo. mass	Combined Score	MSMS Matched Frag...
423.1612	$C_{23}H_{25}O_3N_3{}^{32}S$	0.19	423.16111	90.34288594945	(Collection)

N-Butyl-4-(*N*-phenylsulfamoyl)-*N*-(pyridin-2-ylmethyl)benzamide (**45**)

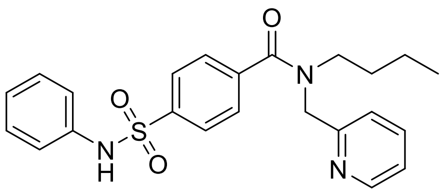

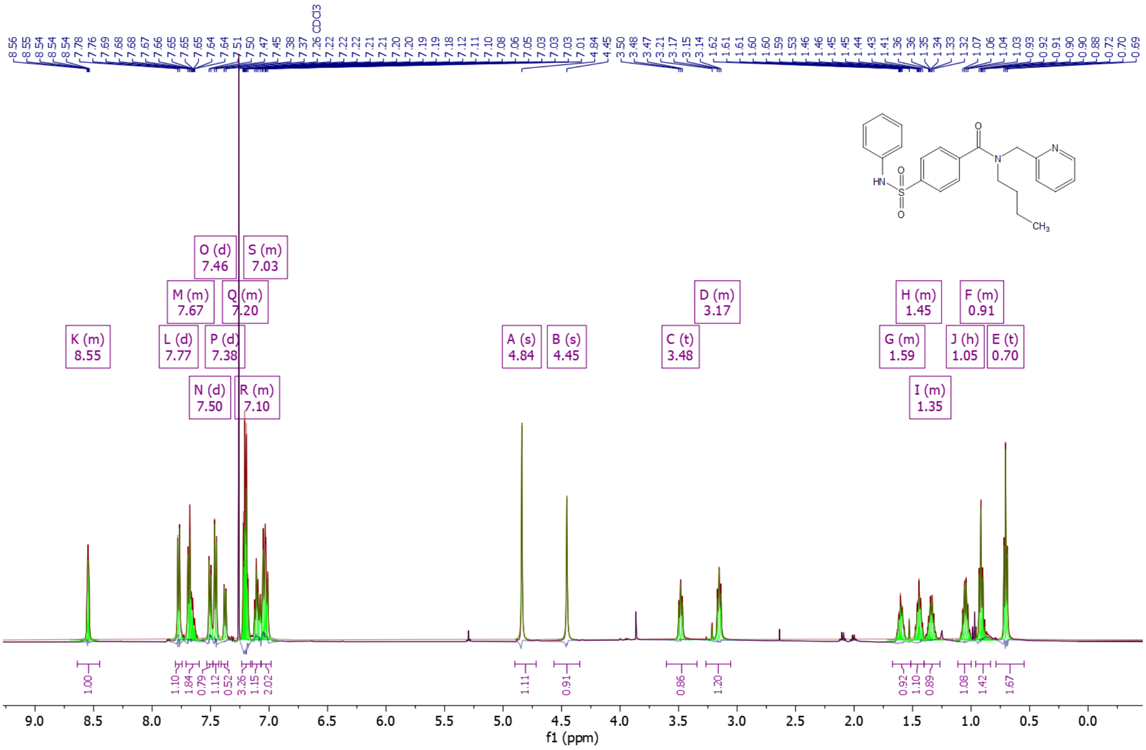

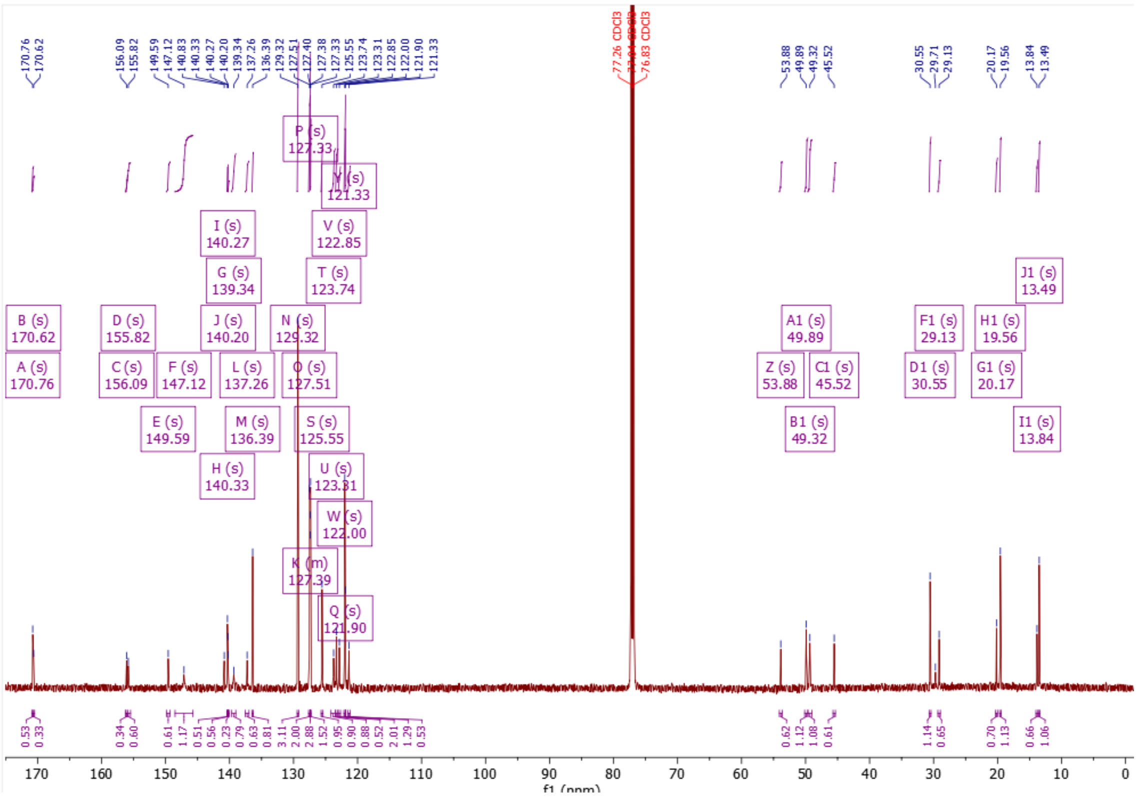

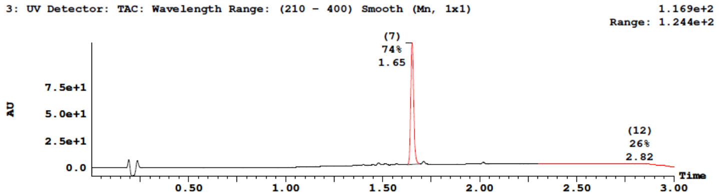

Peak Number	Compound	Time	Area %Total	Mass Found
7		1.65	73.57	Not Found
12		2.82	26.43	Not Found

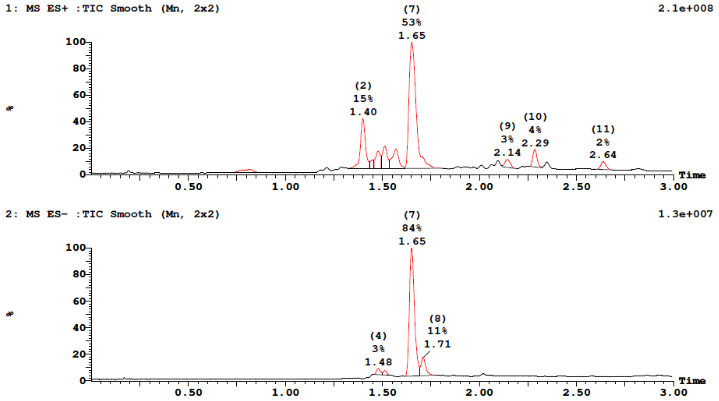

Peak ID	Compound	Time	Mass Found
7		1.65	Not Found

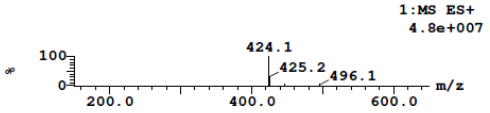

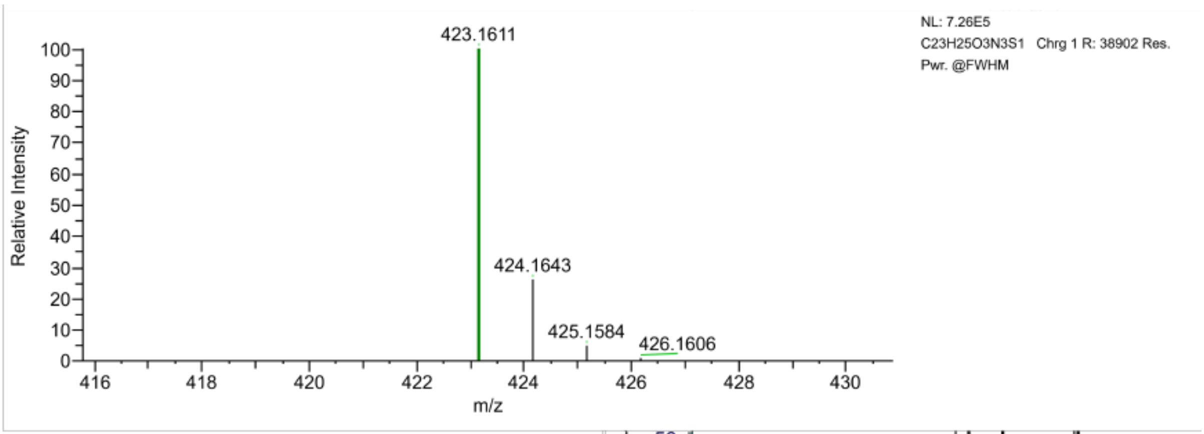

Peak Mass	Display Formula	Delta [ppm]	Theo. mass	Combined Score	MSMS Matched Frag...
423.1608	$C_{23}H_{25}O_3N_3{}^{32}S$	-0.82	423.16111	66.4774356474008	(Collection)

N-Butyl-*N*-(furan-2-ylmethyl)-4-(*N*-phenylsulfamoyl)benzamide (**46**)

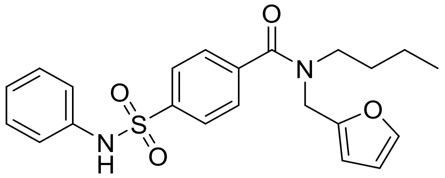

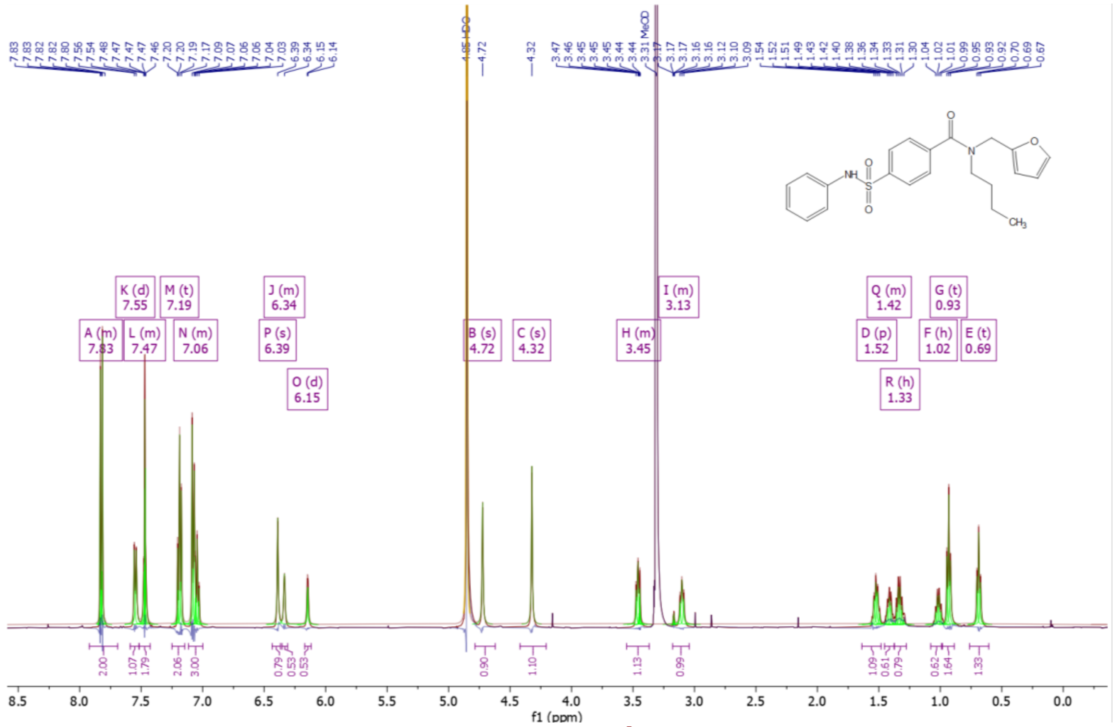

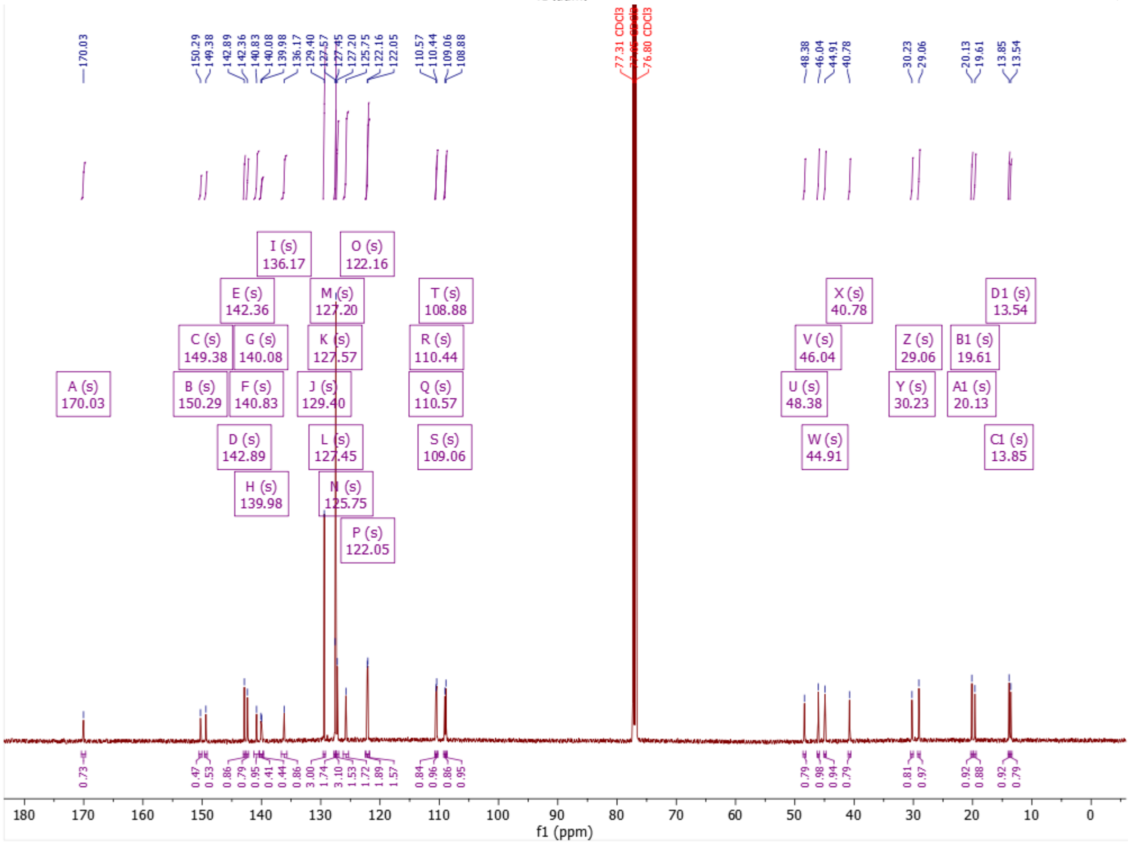

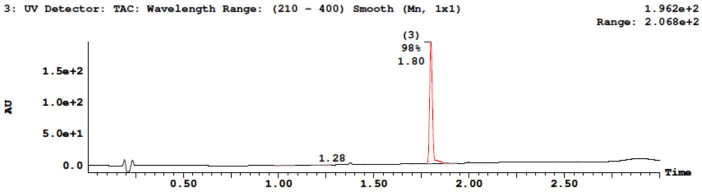

Peak Number	Compound	Time	Area %Total	Mass Found
1		1.28	2.33	Not Found
3		1.80	97.67	Not Found

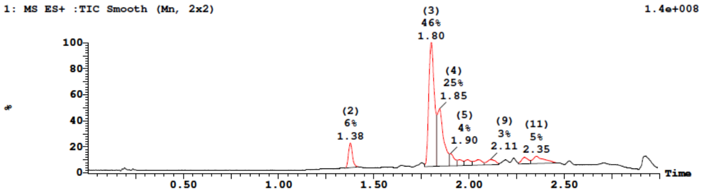

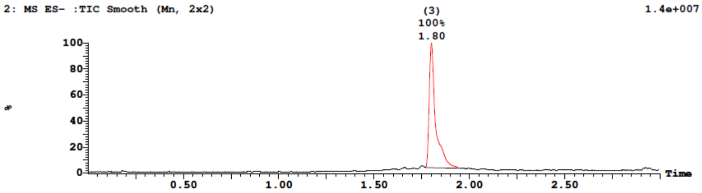

Peak ID	Compound	Time	Mass Found
3		1.80	Not Found

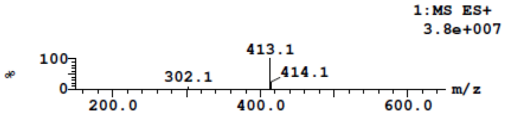

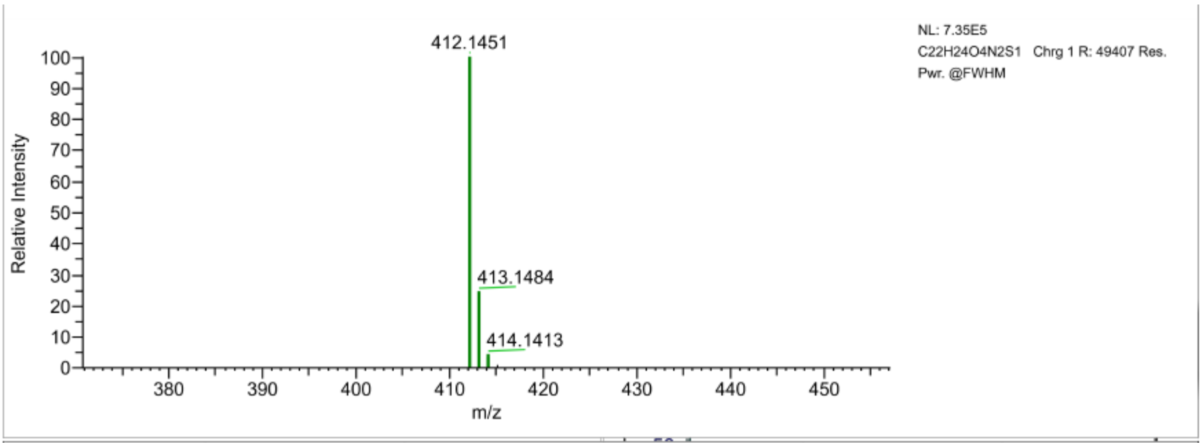

Peak Mass	Display Formula	Delta [ppm]	Theo. mass	Combined Score	MSMS Matched Frag...
412.1451	$C_{22}H_{24}O_4N_2{}^{32}S$	0.03	412.14513	94.910431136023	(Collection)

N-Butyl-4-(*N*-phenylsulfamoyl)-*N*-((tetrahydrofuran-2-yl)methyl)benzamide (**47**)

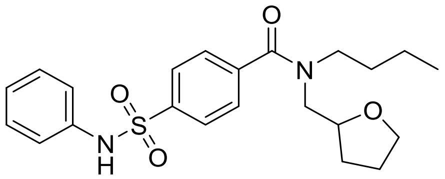

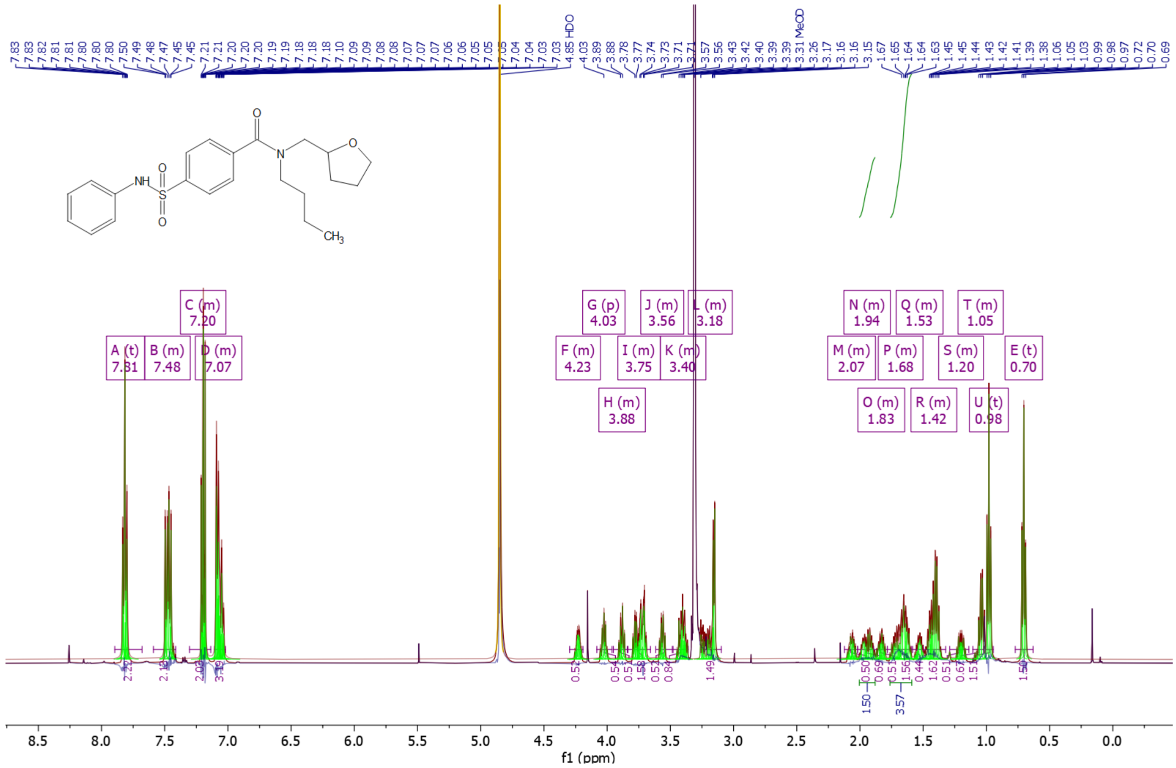

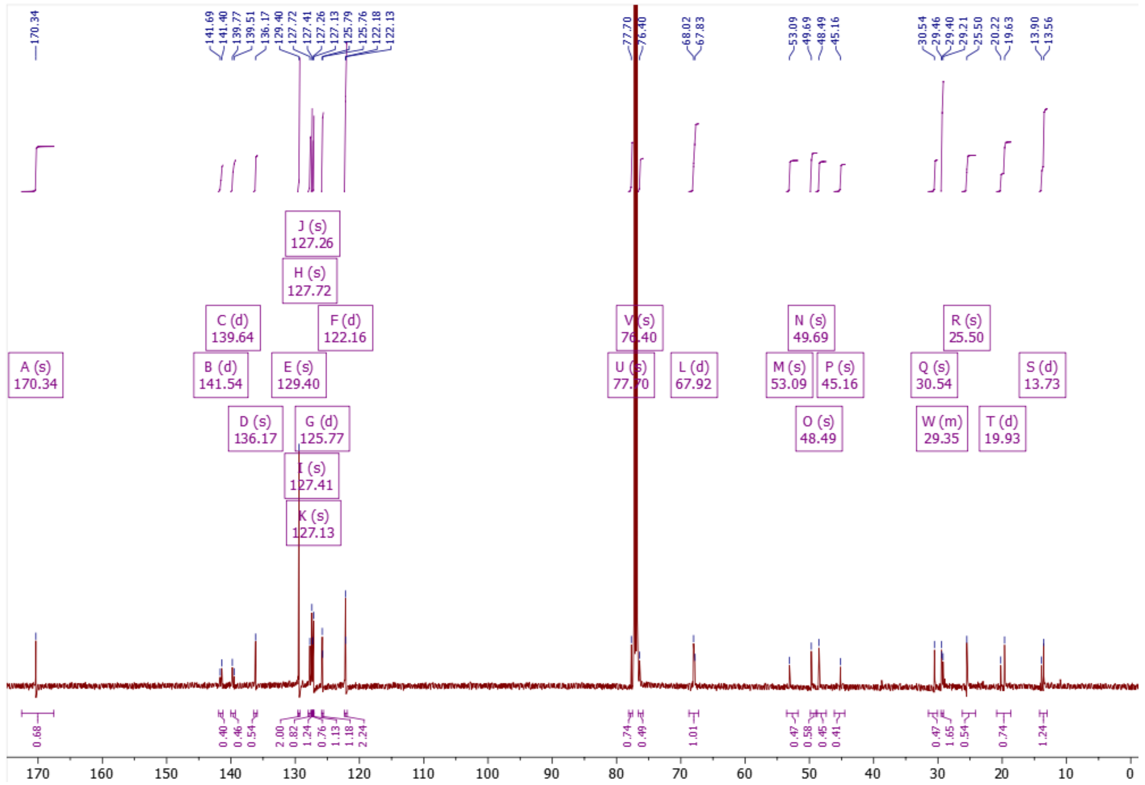

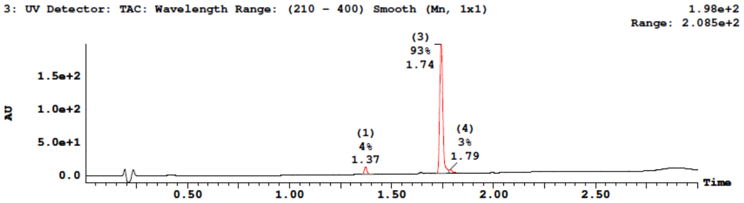

Peak Number	Compound	Time	Area %Total	Mass Found
1		1.37	4.39	Not Found
3		1.74	92.50	Not Found
4		1.79	3.11	Not Found

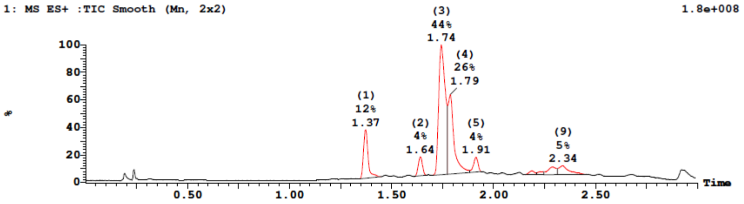

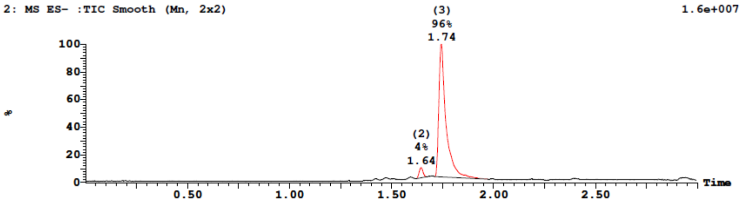

Peak ID	Compound	Time	Mass Found
3		1.74	Not Found

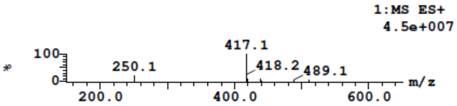

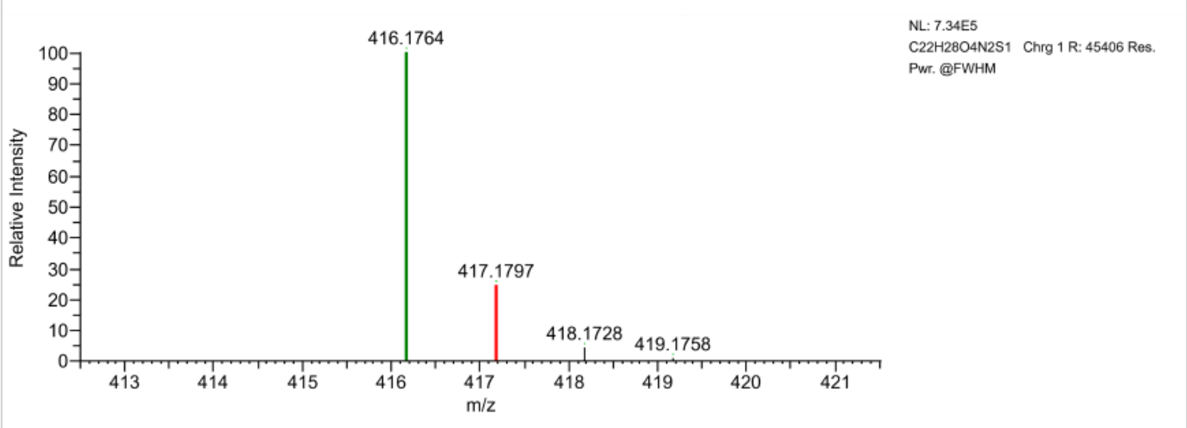

Peak Mass	Display Formula	Delta [ppm]	Theo. mass	Combined Score	MSMS Matched Frag...
416.1766	$C_{22}H_{28}O_4N_2{}^{32}S$	0.42	416.17643	84.13532154529	(Collection)

3-(4-((*N*-Butyl-4-(*N*-phenylsulfamoyl)benzamido)methyl)phenyl)propanoic acid (**48**)

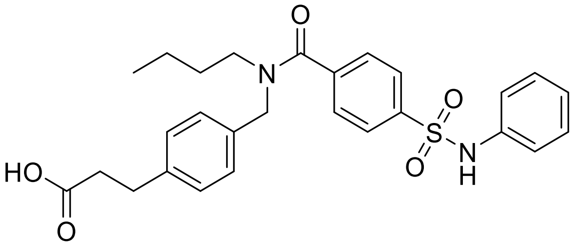

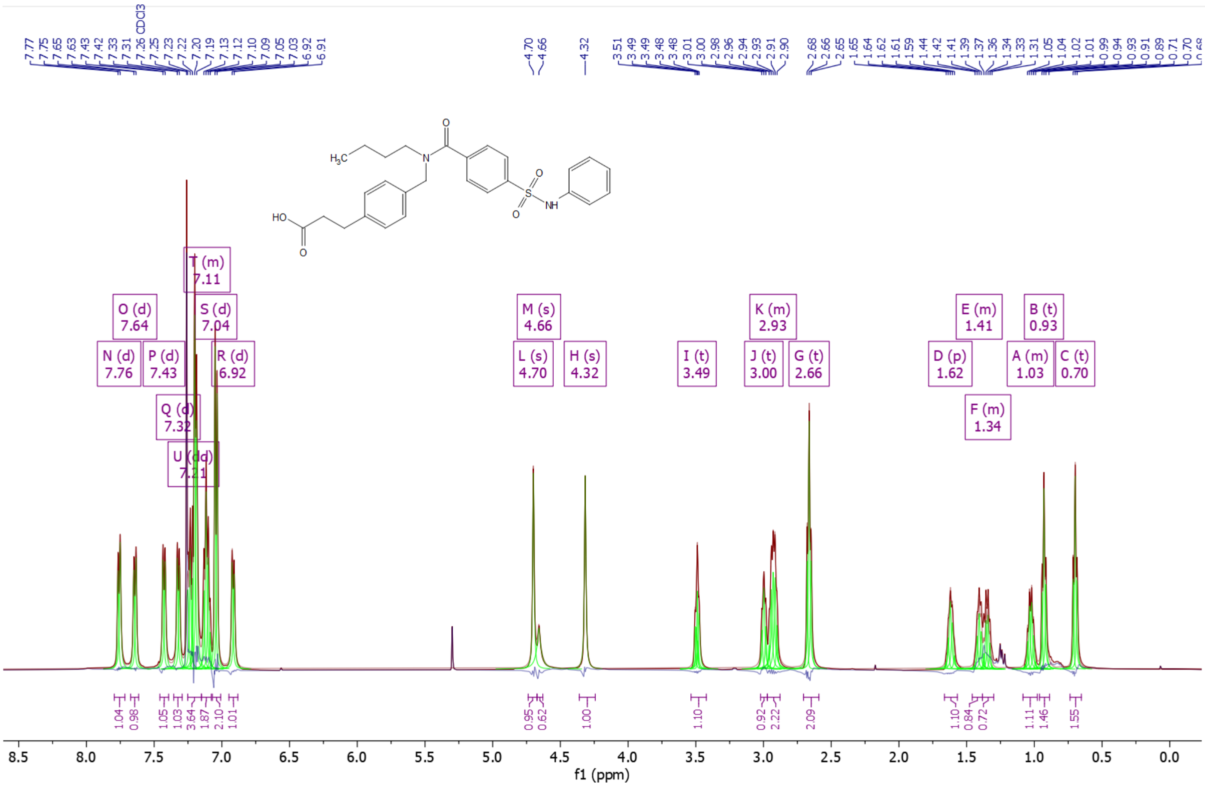

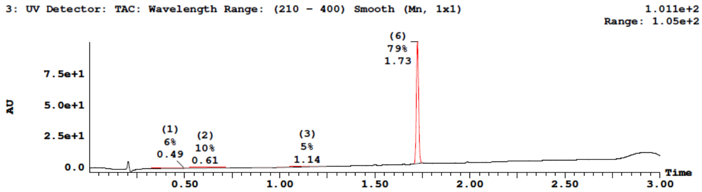

Peak Number	Compound	Time	Area %Total	Mass Found
1		0.49	5.62	Not Found
2		0.61	9.95	Not Found
3		1.14	5.25	Not Found
6		1.73	79.18	Not Found

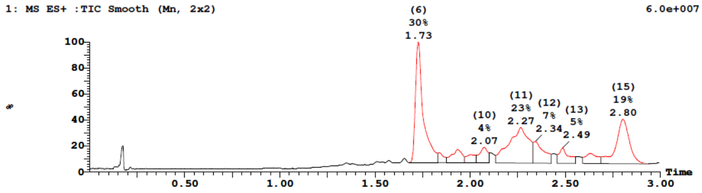

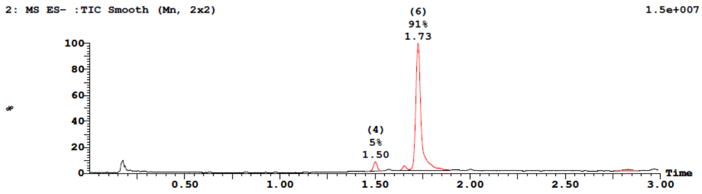

Peak ID	Compound	Time	Mass Found
6		1.73	Not Found

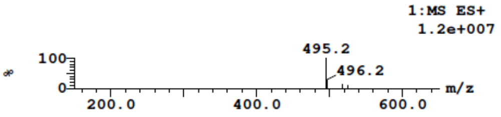

N-Butyl-4-(*N*-phenylsulfamoyl)-*N*-(4-(piperazin-1-yl)benzyl)benzamide (**49**)

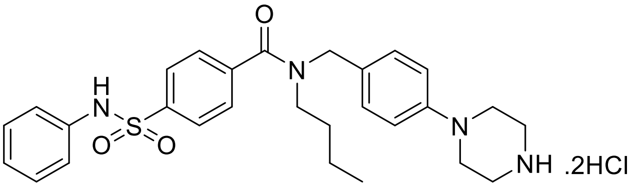

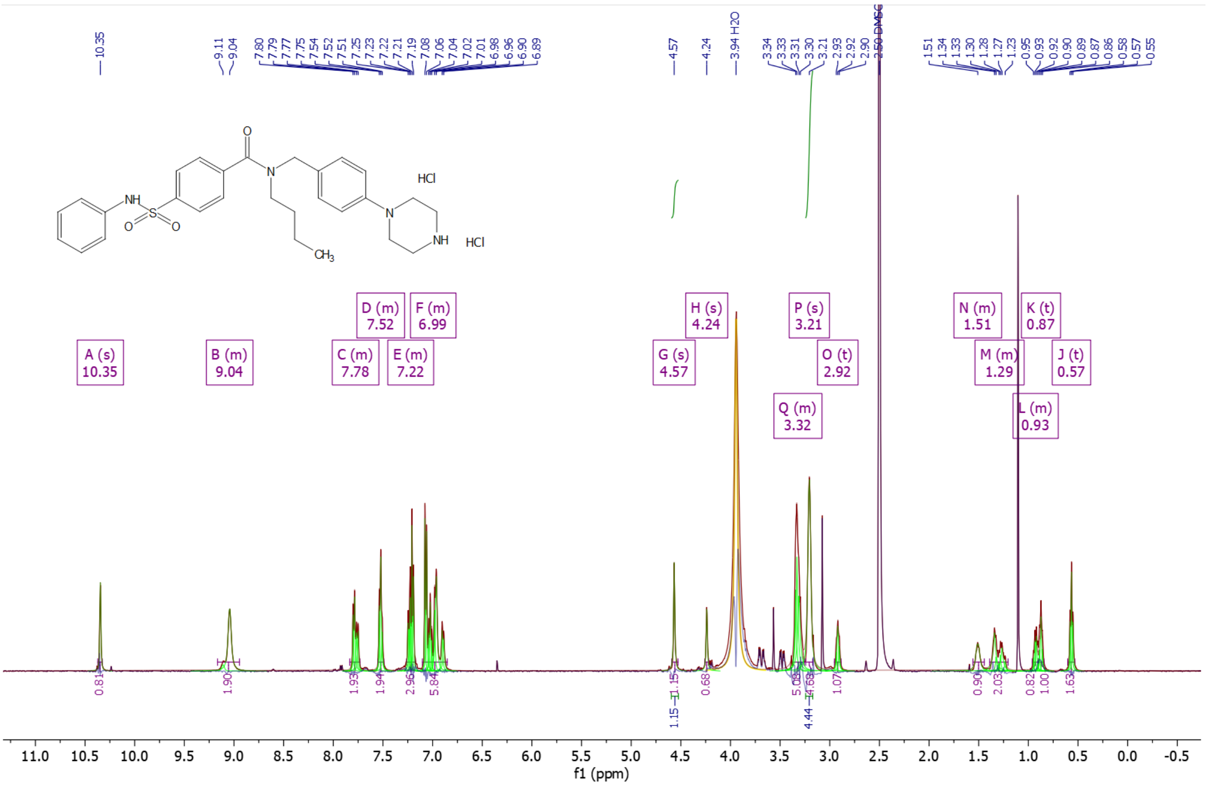

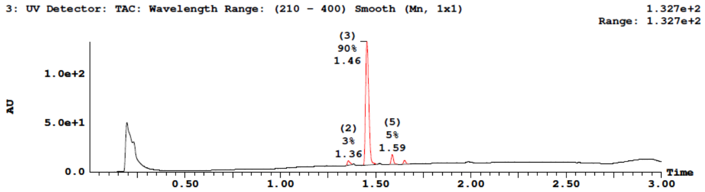

Peak Number	Compound	Time	Area %Total	Mass Found
2		1.36	2.66	Not Found
3		1.46	90.13	Not Found
5		1.59	5.12	Not Found
6		1.65	2.09	Not Found

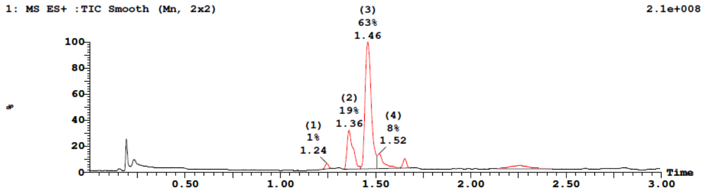

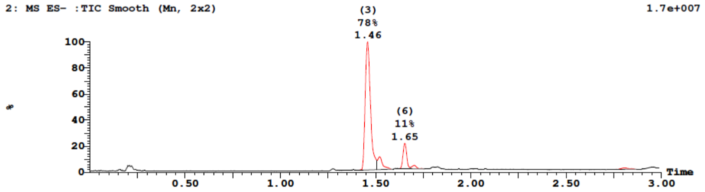

Peak ID	Compound	Time	Mass Found
3		1.46	Not Found

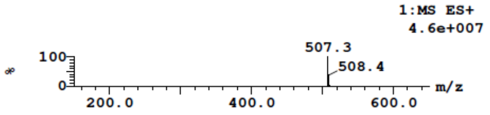

N-[[4-(4-Benzylpiperazin-1-yl)phenyl]methyl]-*N*-butyl-4-(phenylsulfamoyl)benzamide (**50**)

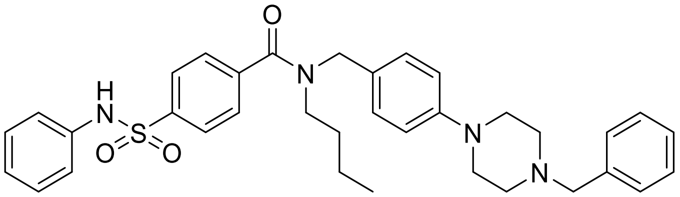

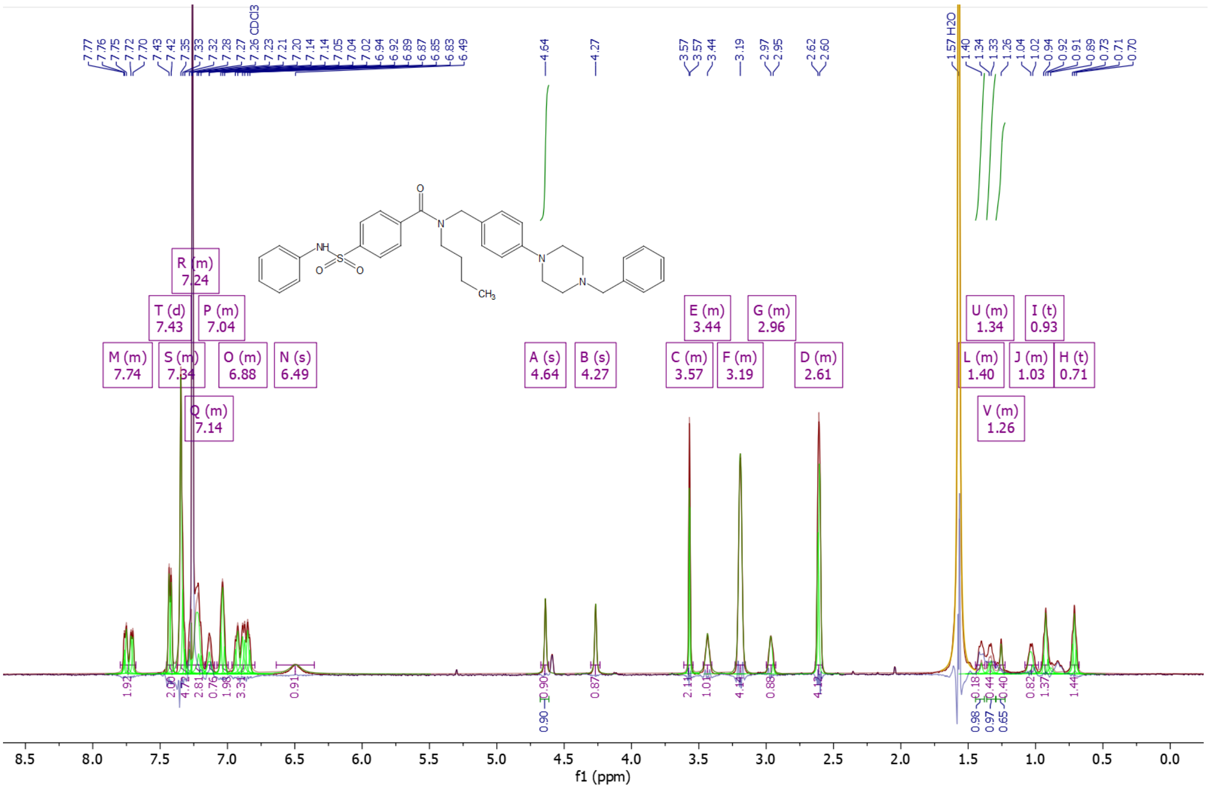

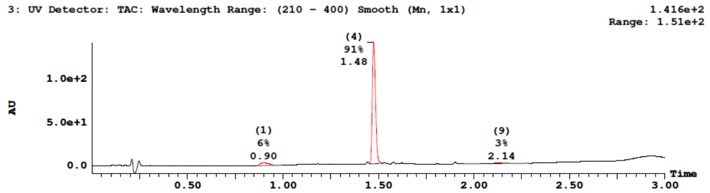

Peak Number	Compound	Time	Area %Total	Mass Found
1		0.90	6.02	Not Found
4		1.48	91.26	Not Found
9		2.14	2.73	Not Found

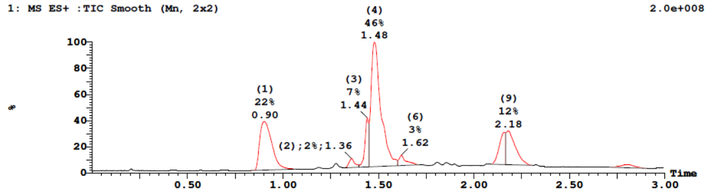

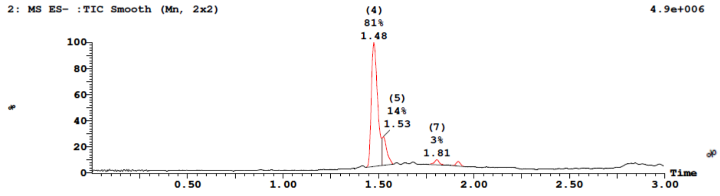

Peak ID	Compound	Time	Mass Found
3		1.44	Not Found

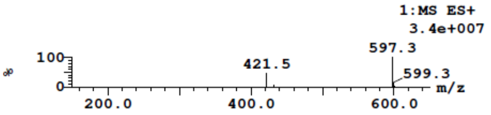

N-Butyl-*N*-(1*H*-indol-5-ylmethyl)-4-(phenylsulfamoyl)benzamide (**51**)

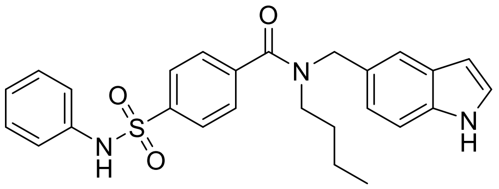

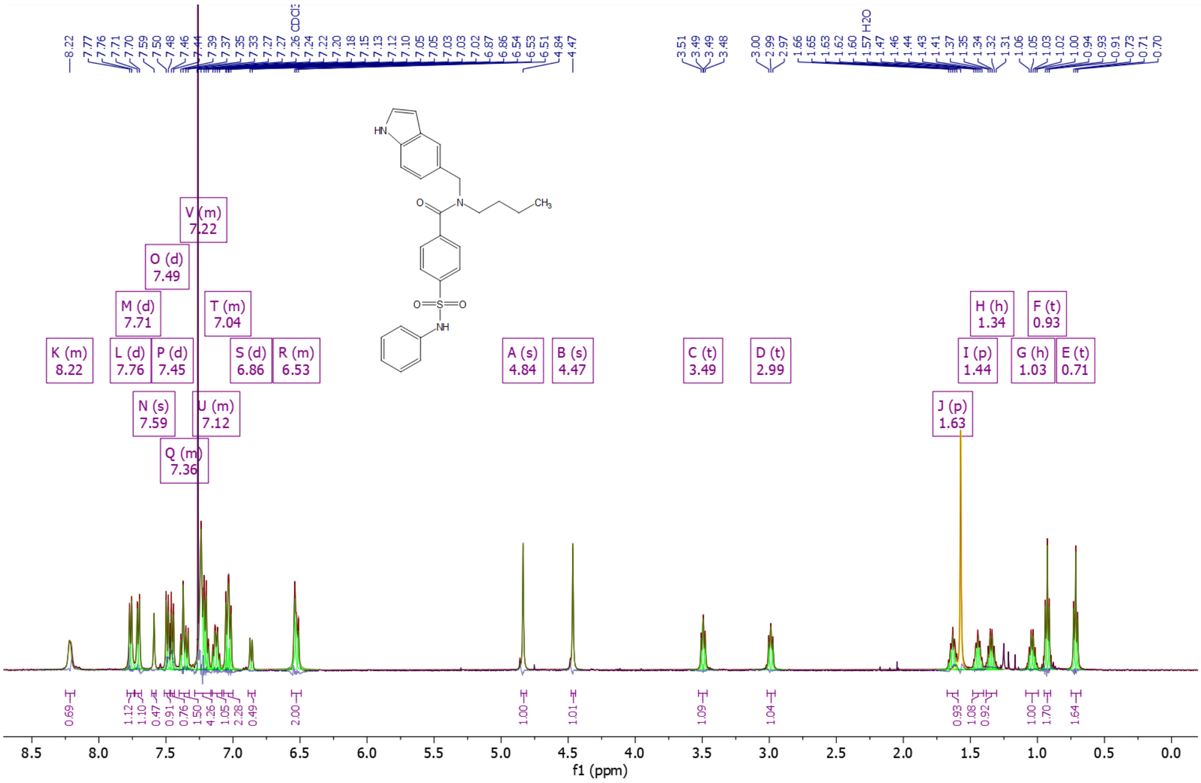

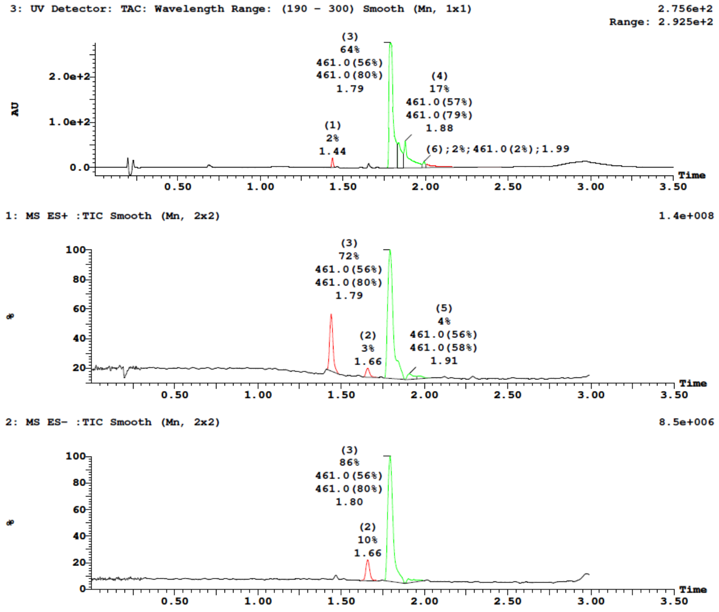

Peak ID	Compound	Time	Mass Found
3	Found	1.79	484,462

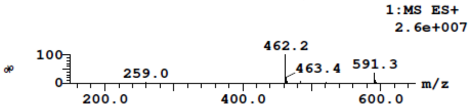

N-Benzyl-*N*-ethyl-4-(*N*-phenylsulfamoyl)benzamide (**52**)

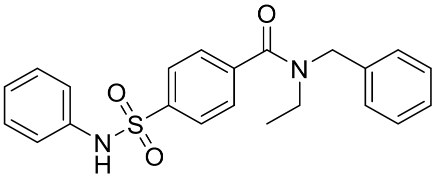

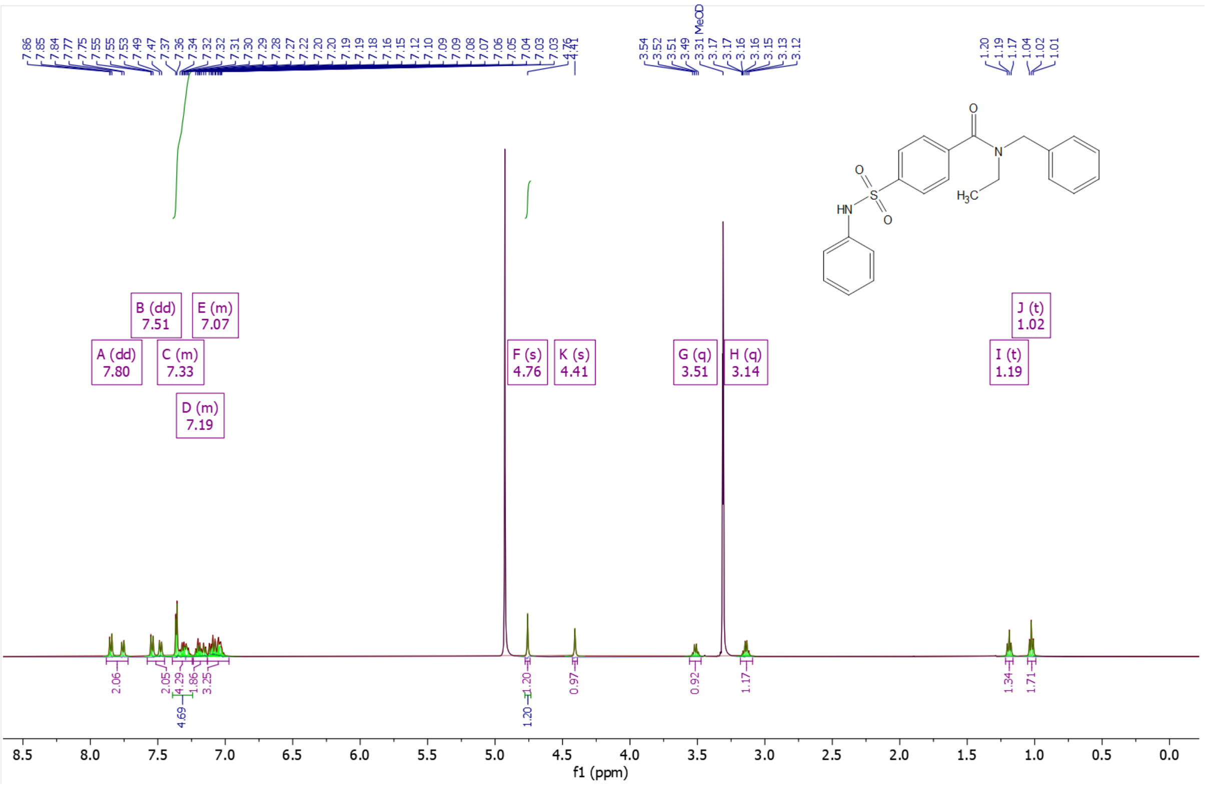

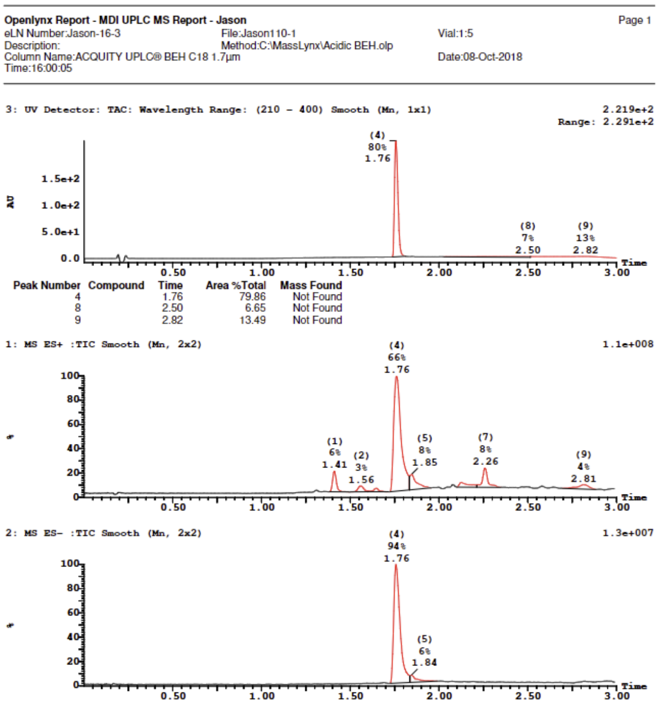

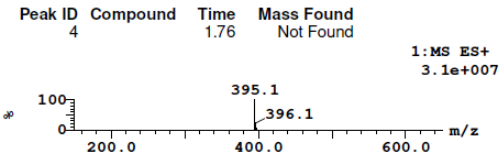

N-Benzyl-*N*-(2-hydroxyethyl)-4-(*N*-phenylsulfamoyl)benzamide (**53**)

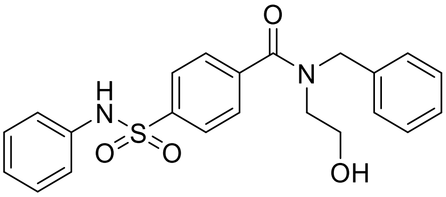

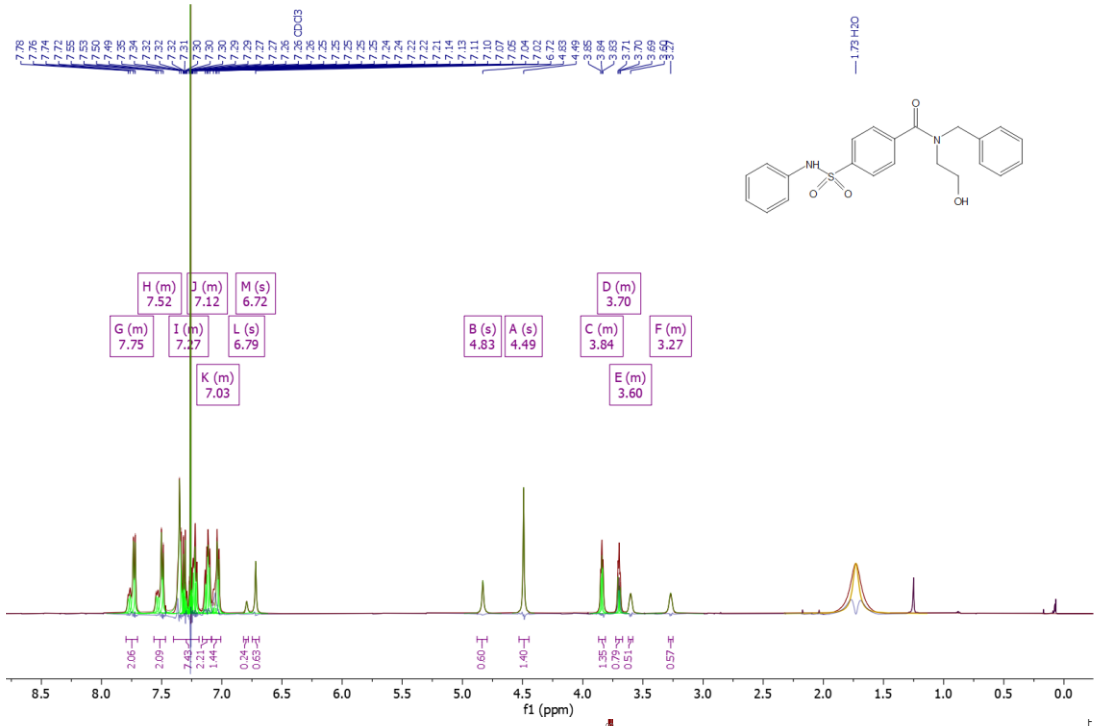

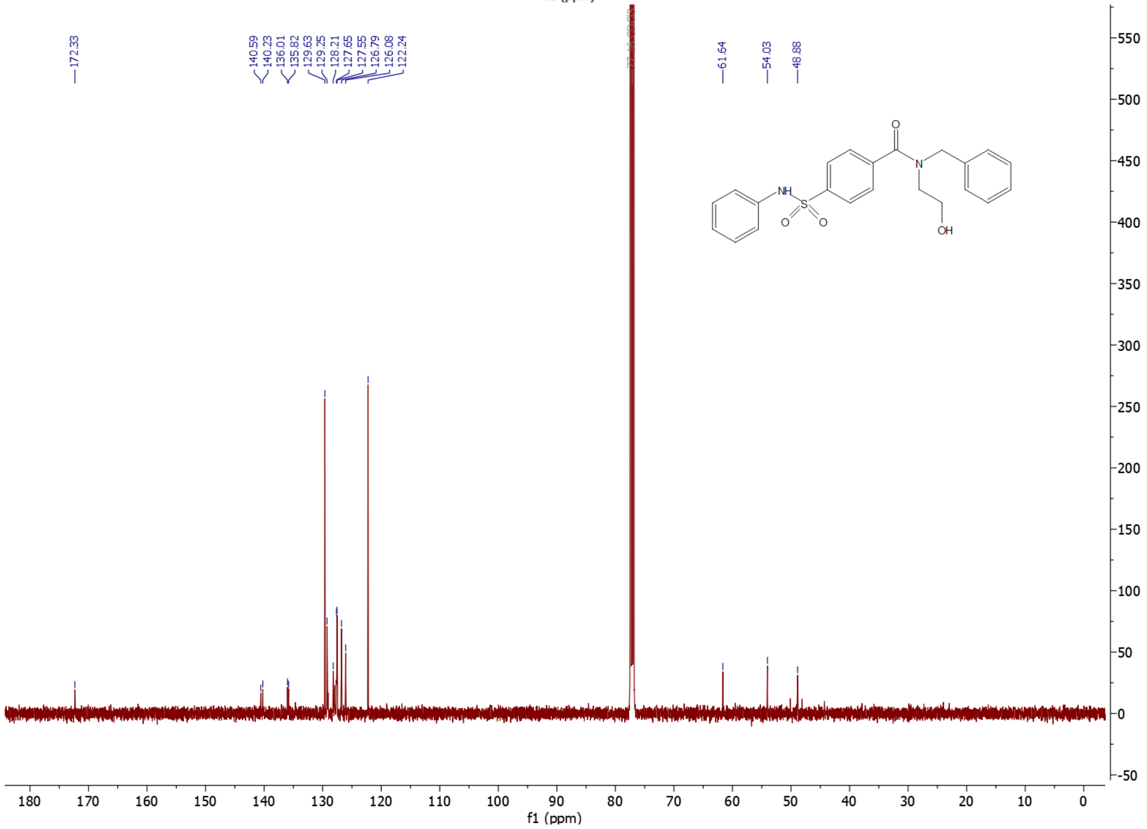

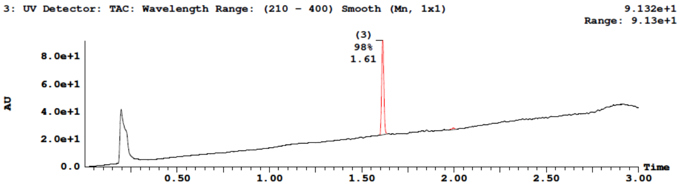

Peak Number	Compound	Time	Area %Total	Mass Found
3		1.61	97.87	Not Found
8		2.00	2.13	Not Found

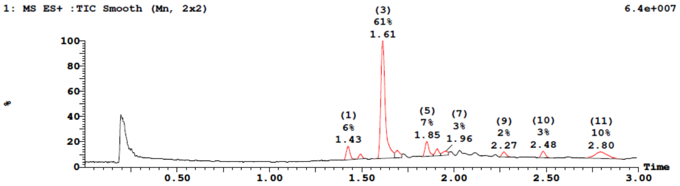

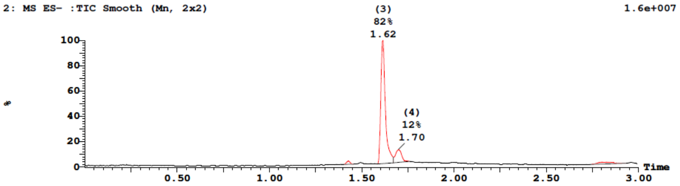

Peak ID	Compound	Time	Mass Found
3		1.61	Not Found

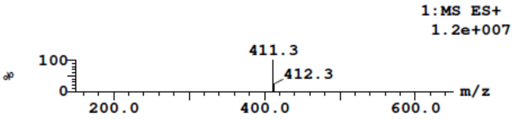

N-Benzyl-*N*-(2-cyanoethyl)-4-(*N*-phenylsulfamoyl)benzamide (**54**)

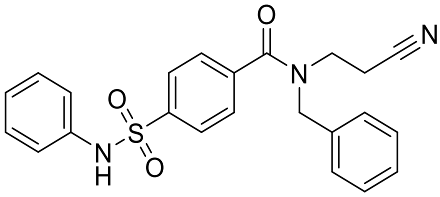

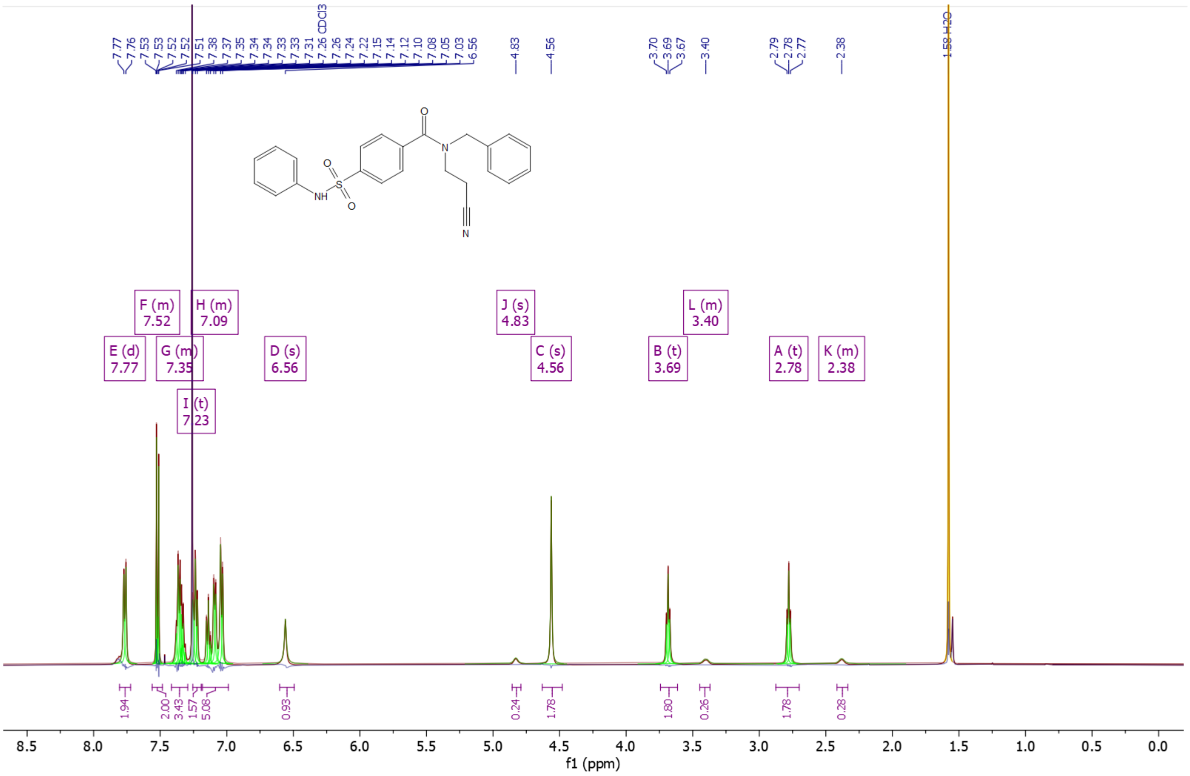

Openlynx Report - MDI UPLC MS Report - Jason Page 1
eLN Number:Jason-15-3 File:Jason112-1 Vial:1:12
Description: Method:C:\MassLynx\Acidic BEH.olp
Column Name:ACQUITY UPLC® BEH C18 1.7µm Date:08-Oct-2018
Time:17:03:24

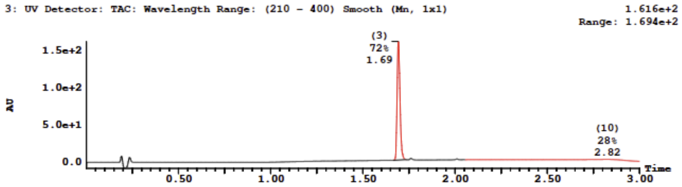

Peak Number	Compound	Time	Area %Total	Mass Found
3		1.69	71.60	Not Found
10		2.82	28.40	Not Found

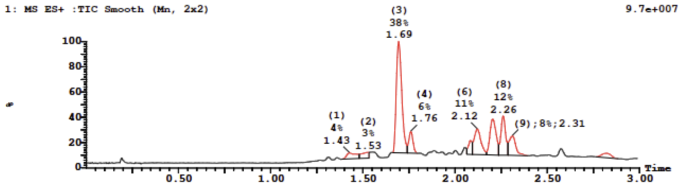

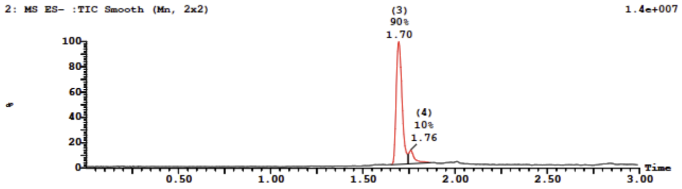

Peak ID	Compound	Time	Mass Found
3		1.69	Not Found

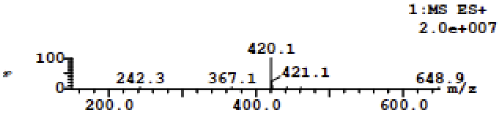

N-(2-Cyanoethyl)-4-(phenylsulfamoyl)-*N*-(4-pyridylmethyl)benzamide (**55**)

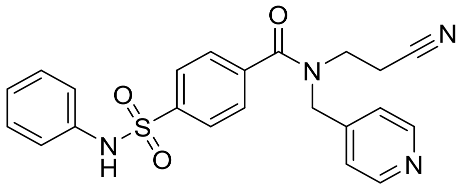

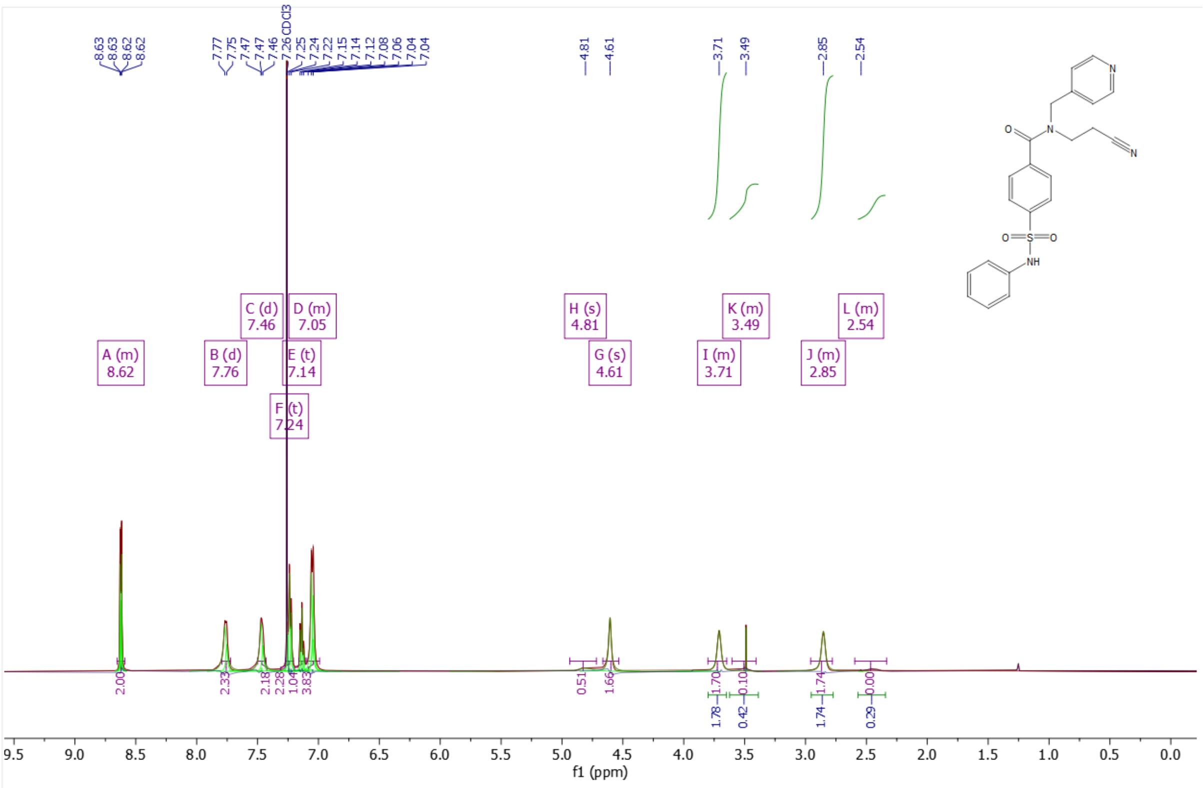

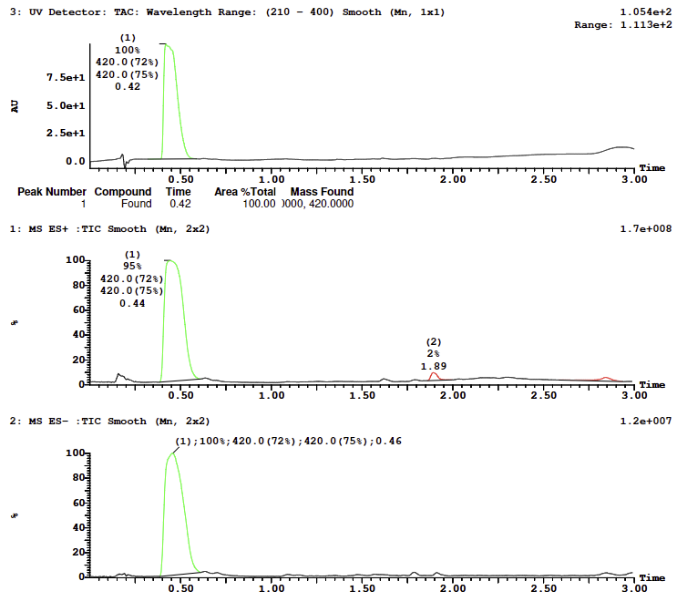

Peak ID	Compound	Time	Mass Found
1	Found	0.44	421

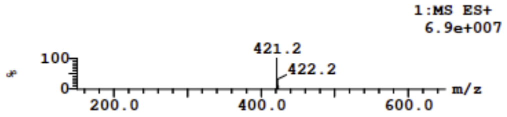

N-Benzyl-*N*-(3-imidazol-1-ylpropyl)-4-(phenylsulfamoyl)benzamide (**56**)

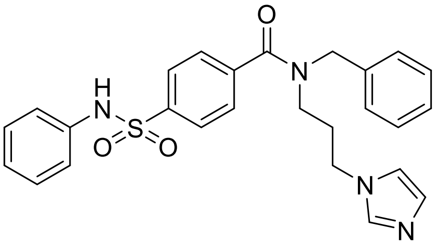

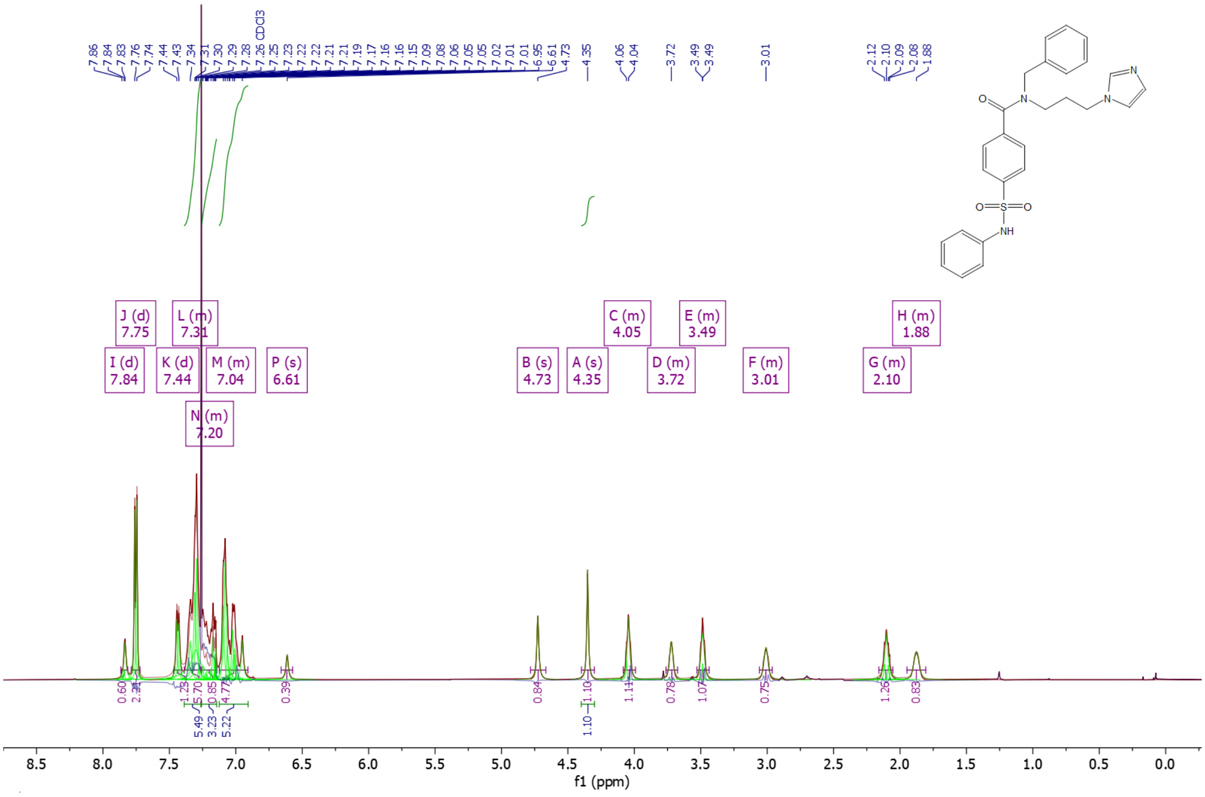

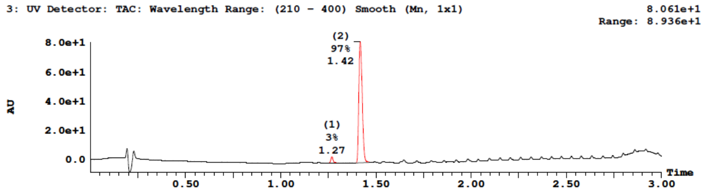

Peak Number	Compound	Time	Area %Total	Mass Found
1		1.27	3.41	Not Found
2		1.42	96.59	Not Found

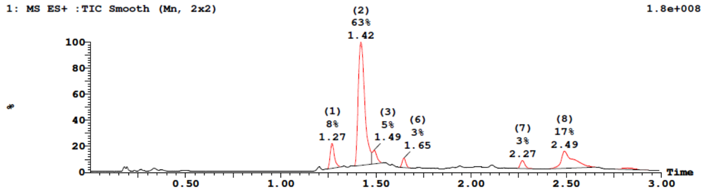

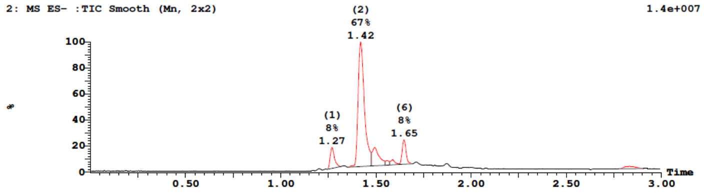

Peak ID	Compound	Time	Mass Found
2		1.42	Not Found

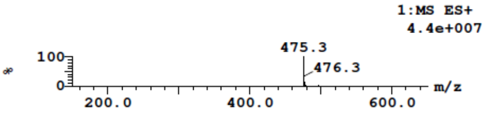

N-Benzyl-*N*-(cyclopropylmethyl)-4-(phenylsulfamoyl)benzamide (**57**)

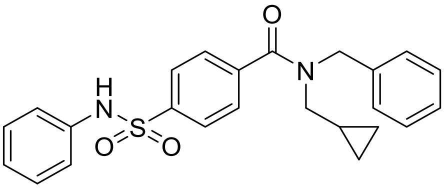

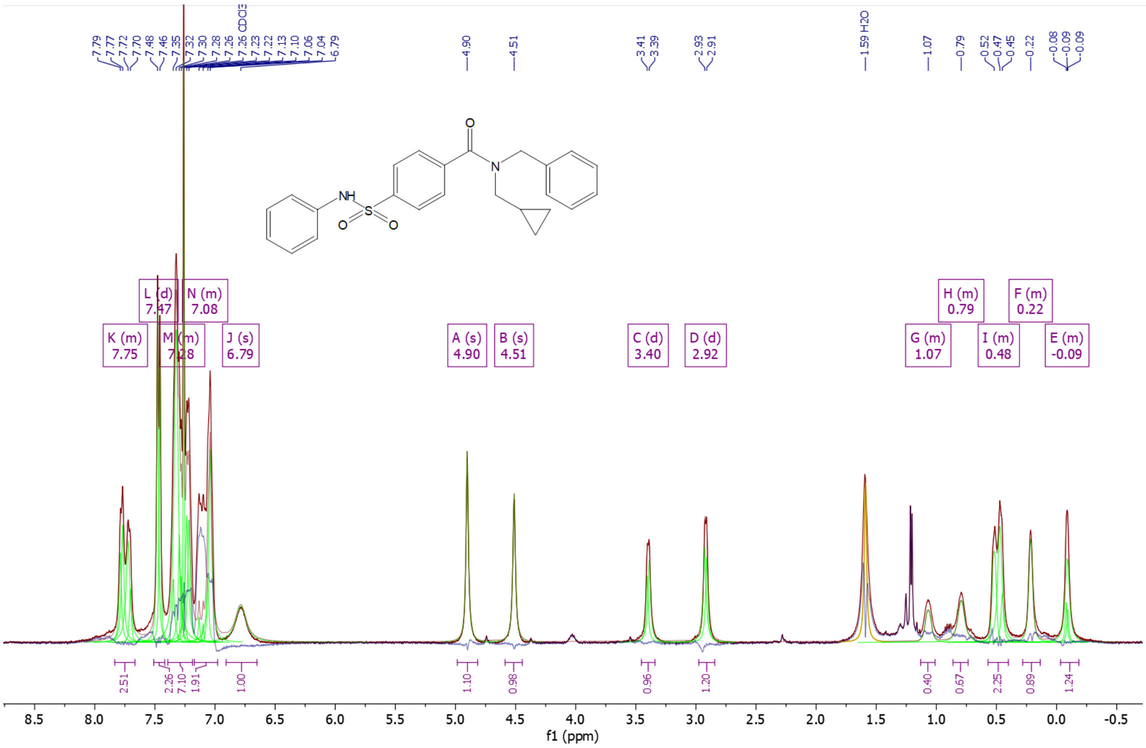

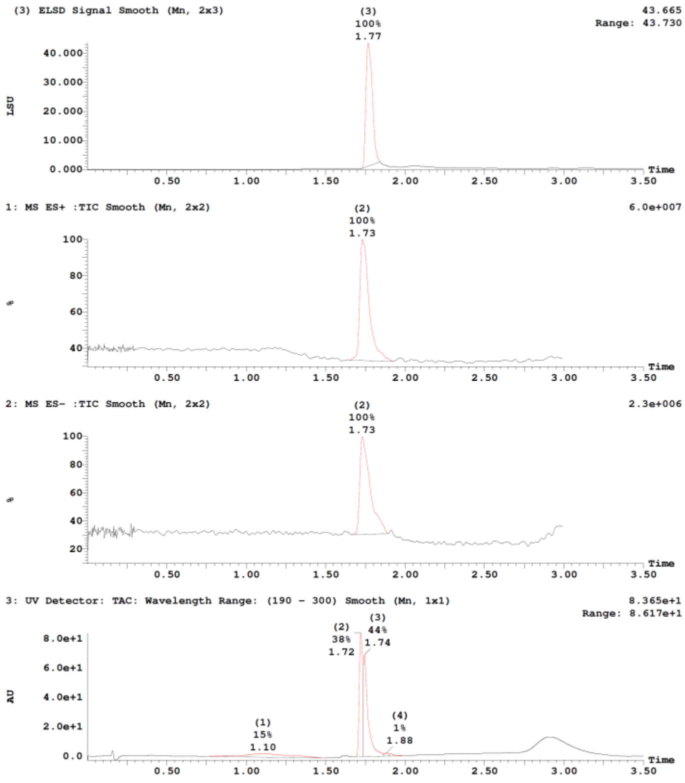

Peak ID	Compound	Time	Mass Found
2		1.73	Not Found

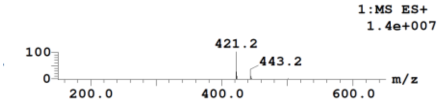

N-Benzyl-*N*-cyclopropyl-4-(phenylsulfamoyl)benzamide (**58**)

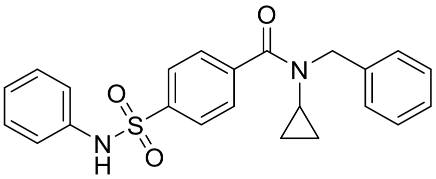

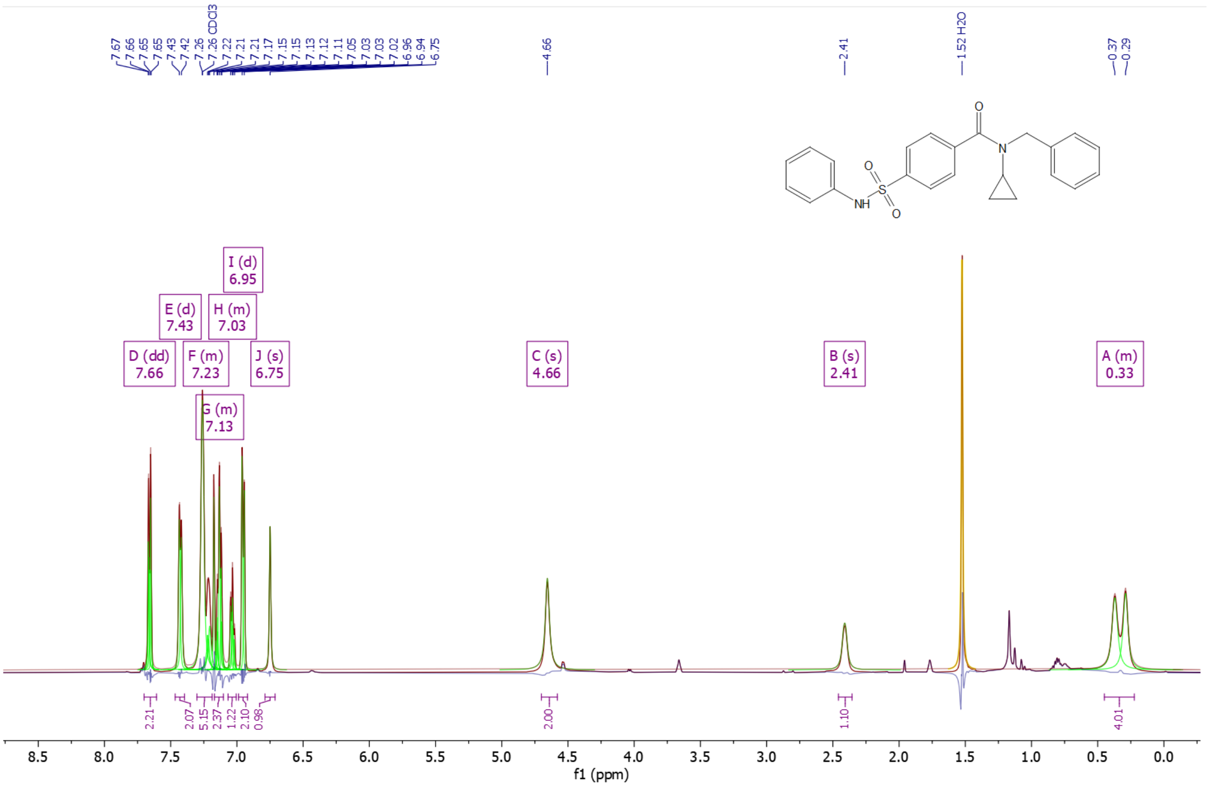

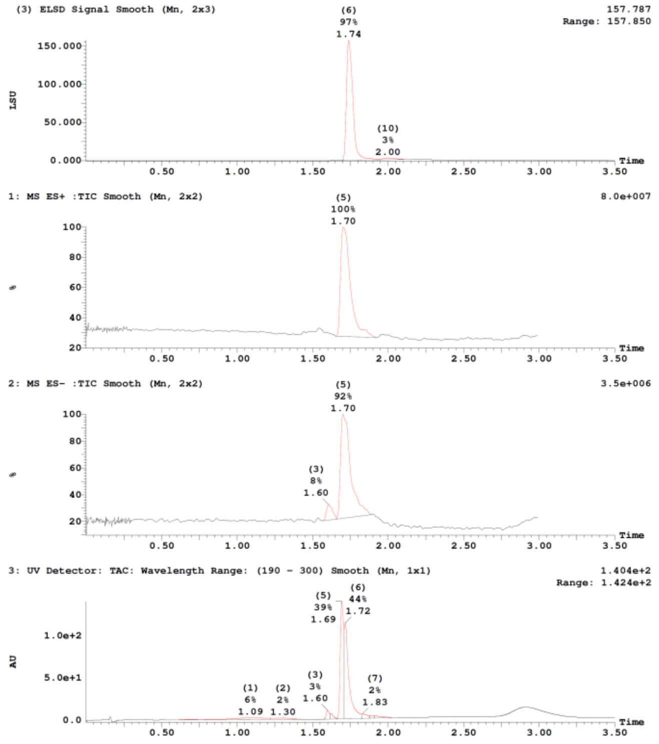

Peak ID	Compound	Time	Mass Found
5		1.70	Not Found

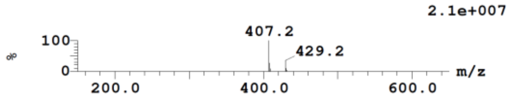

N-Benzyl-*N*-isobutyl-4-(phenylsulfamoyl)benzamide (**59**)

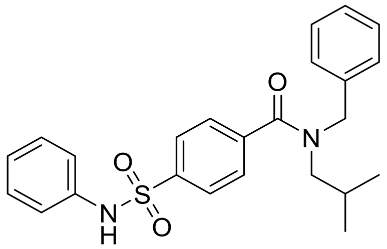

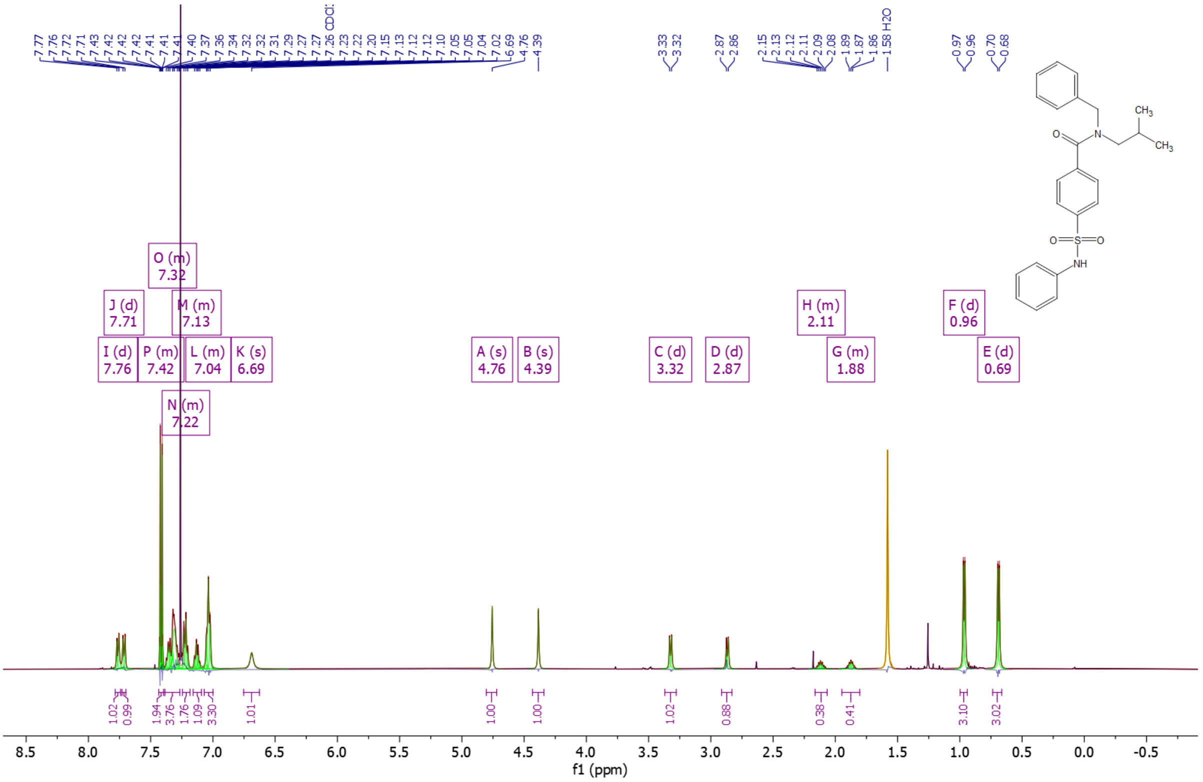

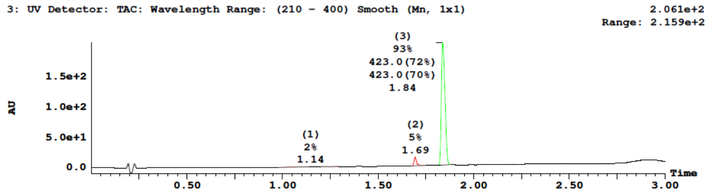

Peak Number	Compound	Time	Area %Total	Mass Found
1		1.14	2.04	Not Found
2		1.69	4.70	Not Found
3	Found	1.84	93.27	)000, 423.0000

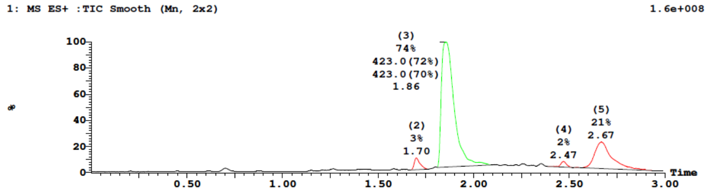

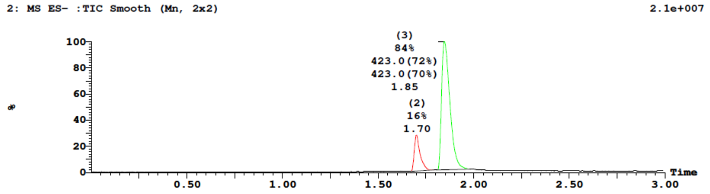

Peak ID	Compound	Time	Mass Found
3	Found	1.86	423

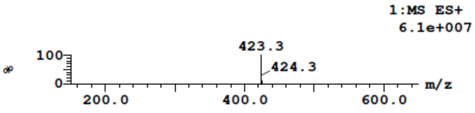

N-Benzyl-*N*-[[1-(imidazol-1-ylmethyl)cyclopropyl]methyl]-4-(phenylsulfamoyl)benzamide (**60**)

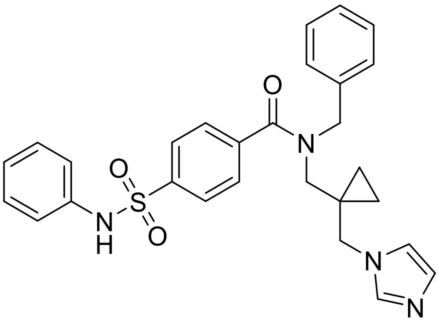

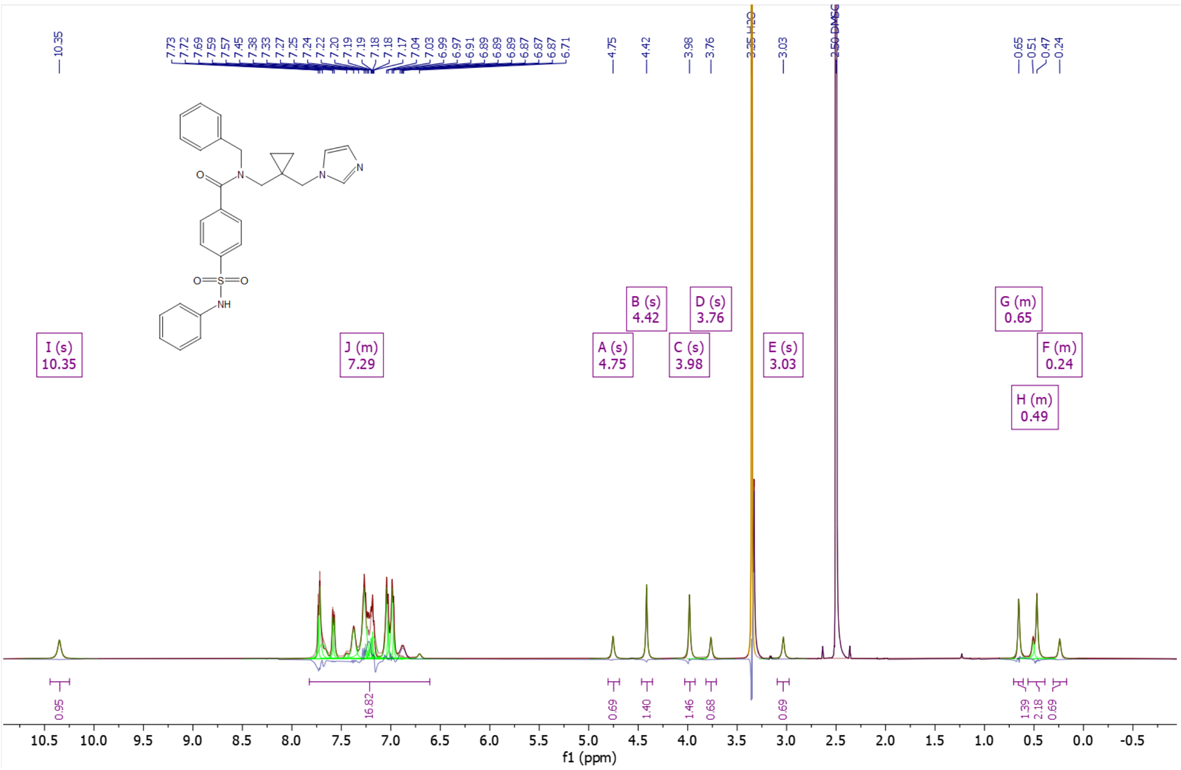

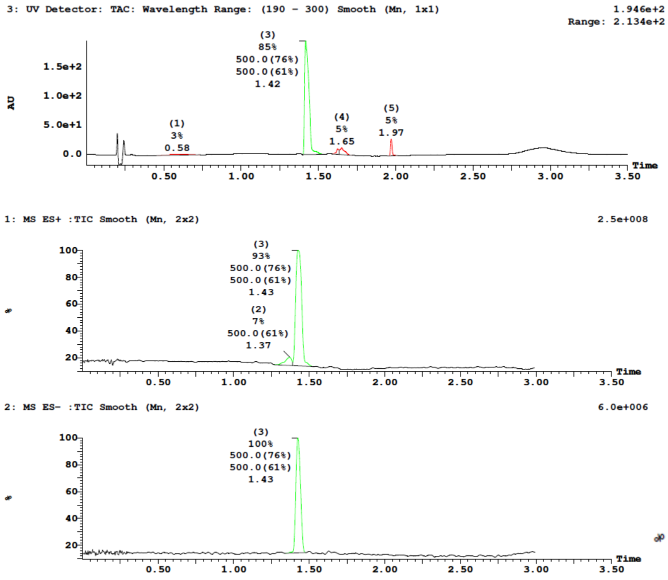

Peak ID	Compound	Time	Mass Found
3	Found	1.43	523,501

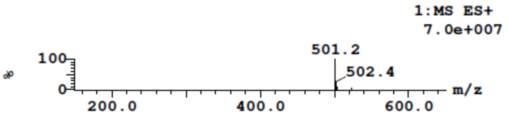

N-(Cyclopropylmethyl)-*N*-(1*H*-indol-5-ylmethyl)-4-(phenylsulfamoyl)benzamide (**61**)

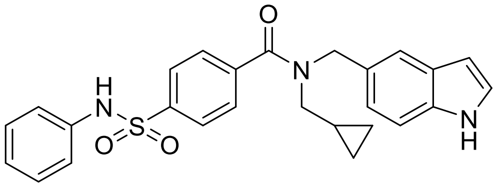

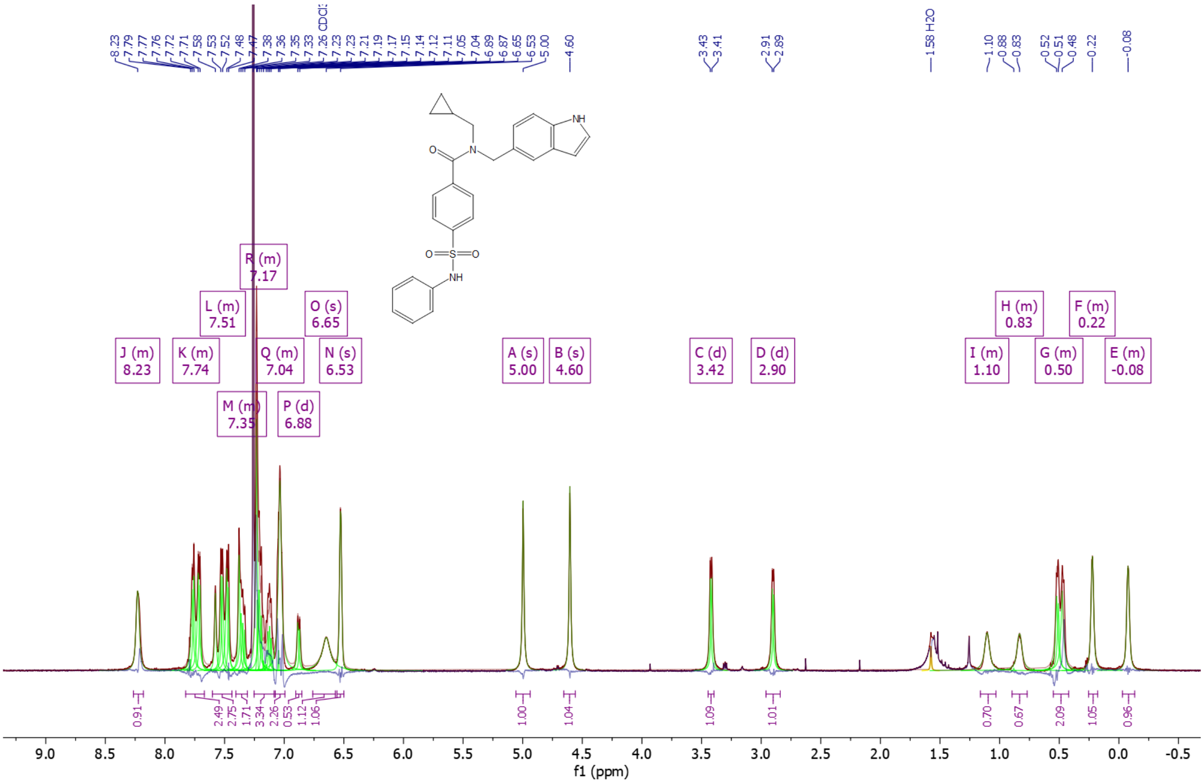

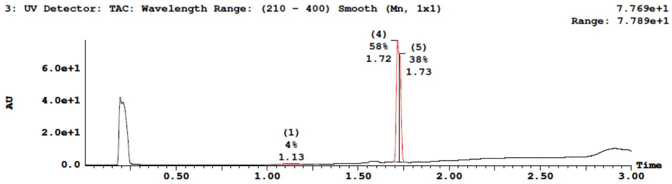

Peak Number	Compound	Time	Area %Total	Mass Found
1		1.13	4.03	Not Found
4		1.72	57.95	Not Found
5		1.73	38.02	Not Found

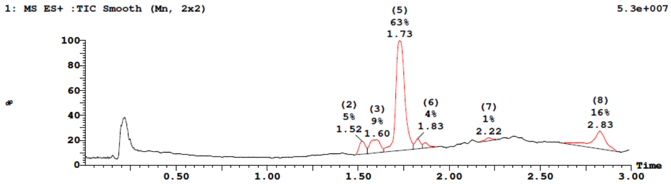

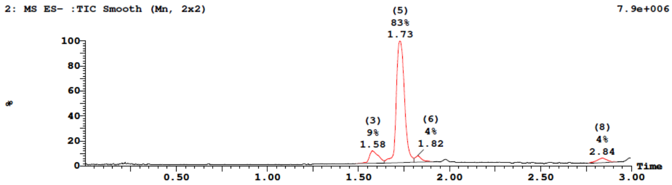

Peak ID	Compound	Time	Mass Found
4		1.72	Not Found

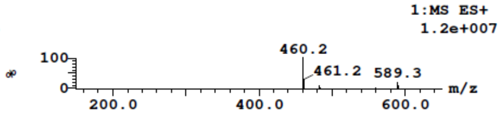

N-(1,3-Benzoxazol-6-ylmethyl)-*N*-(cyclopropylmethyl)-4-(phenylsulfamoyl)benzamide (**62**)

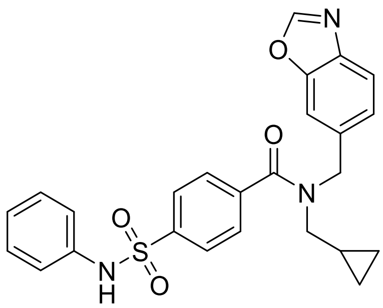

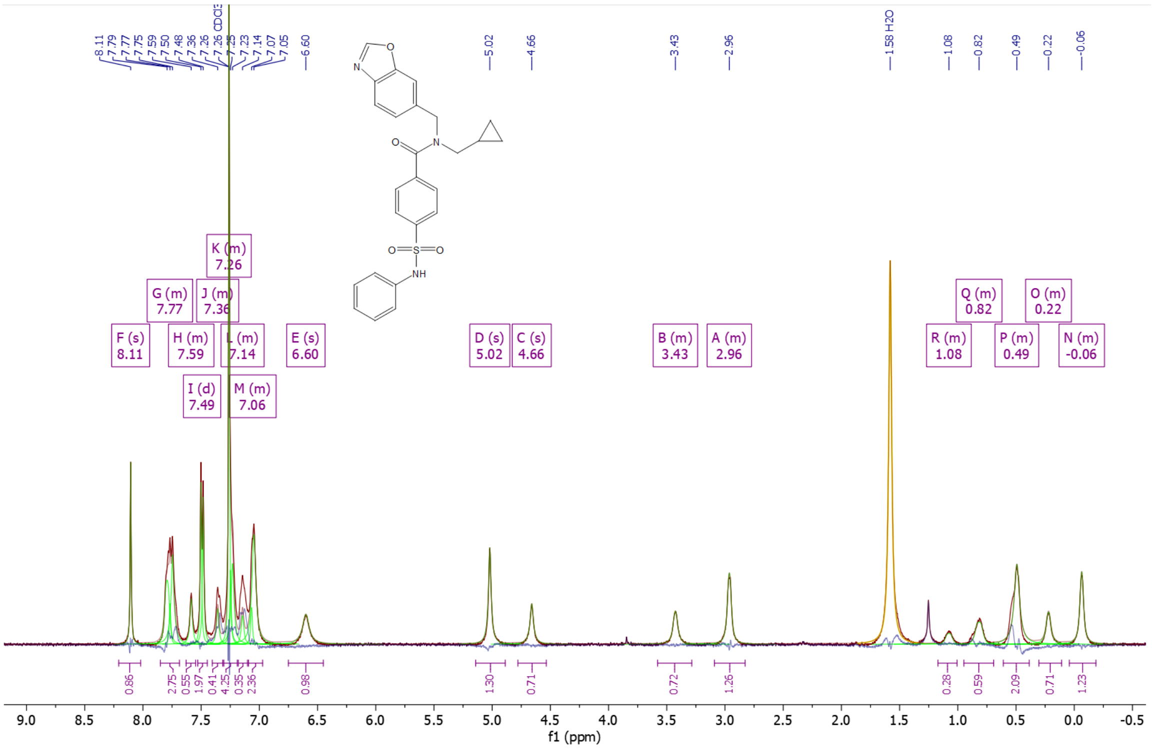

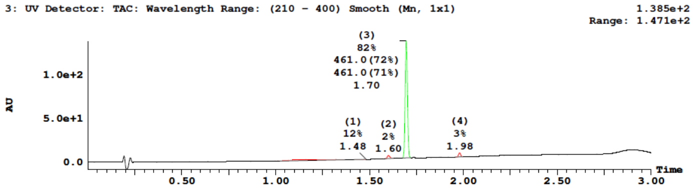

Peak Number	Compound	Time	Area %Total	Mass Found
1		1.48	12.43	Not Found
2		1.60	2.37	Not Found
3	Found	1.70	82.44	)000, 461.0000
4		1.98	2.76	Not Found

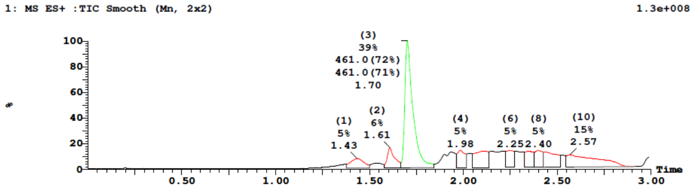

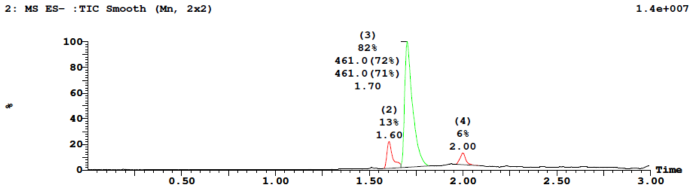

Peak ID	Compound	Time	Mass Found
3	Found	1.70	462

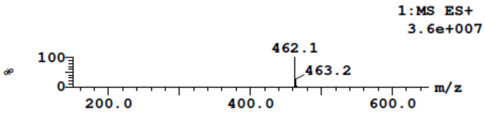

N-[(5-Bromo-2-pyridyl)methyl]-*N*-(cyclopropylmethyl)-4-(phenylsulfamoyl)benzamide (**63**)

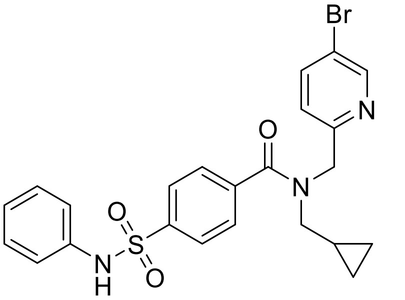

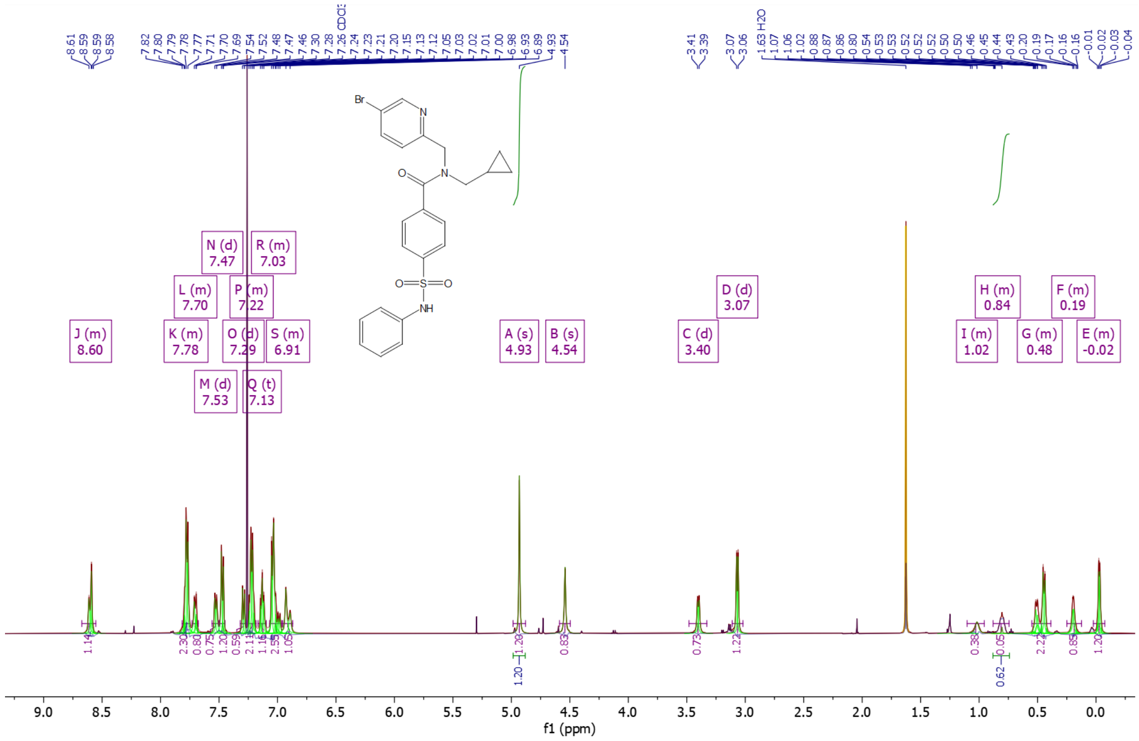

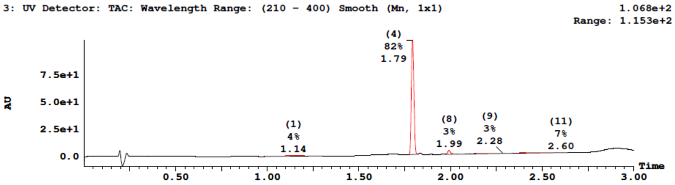

Peak Number	Compound	Time	Area %Total	Mass Found
1		1.14	4.43	Not Found
4		1.79	82.06	Not Found
8		1.99	3.15	Not Found
9		2.28	3.40	Not Found
11		2.60	6.97	Not Found

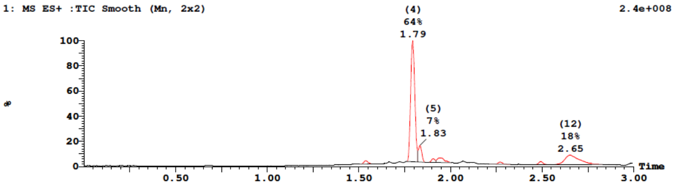

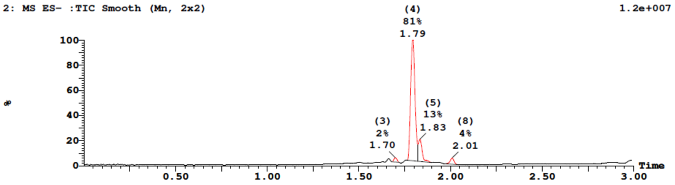

Peak ID	Compound	Time	Mass Found
4		1.79	Not Found

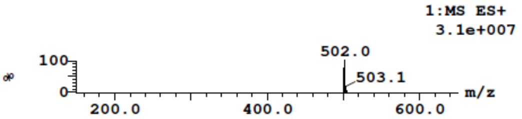

(*S*)-*N*-(Cyclopropylmethyl)-*N*-(1-phenylethyl)-4-(*N*-phenylsulfamoyl)benzamide (**64**)

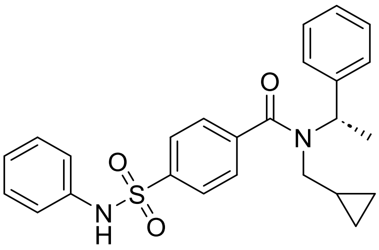

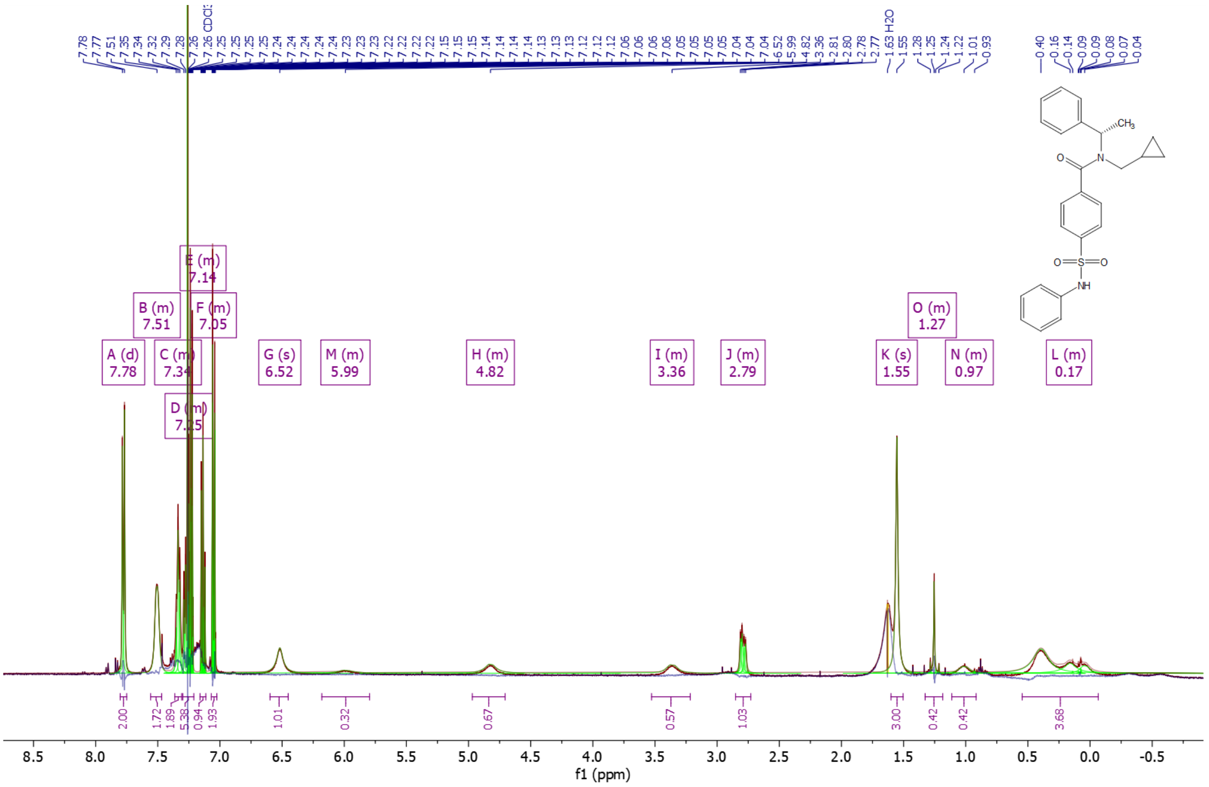

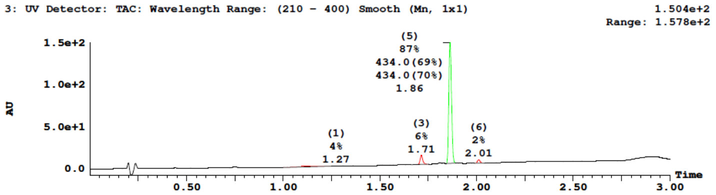

Peak Number	Compound	Time	Area %Total	Mass Found
1		1.27	3.85	Not Found
3		1.71	6.31	Not Found
5	Found	1.86	87.41	)000, 434.0000
6		2.01	2.42	Not Found

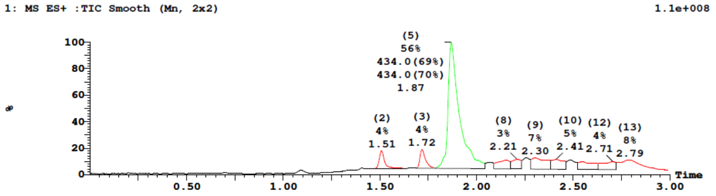

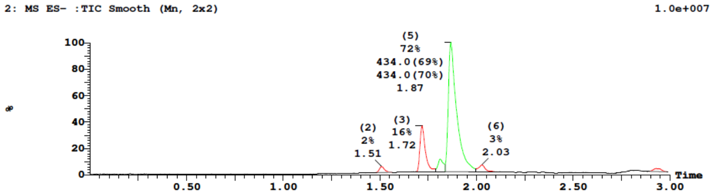

Peak ID	Compound	Time	Mass Found
5	Found	1.87	435

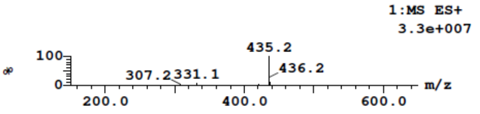

(*R*)-*N*-(Cyclopropylmethyl)-*N*-(1-phenylethyl)-4-(*N*-phenylsulfamoyl)benzamide (**65**)

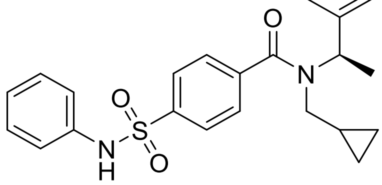

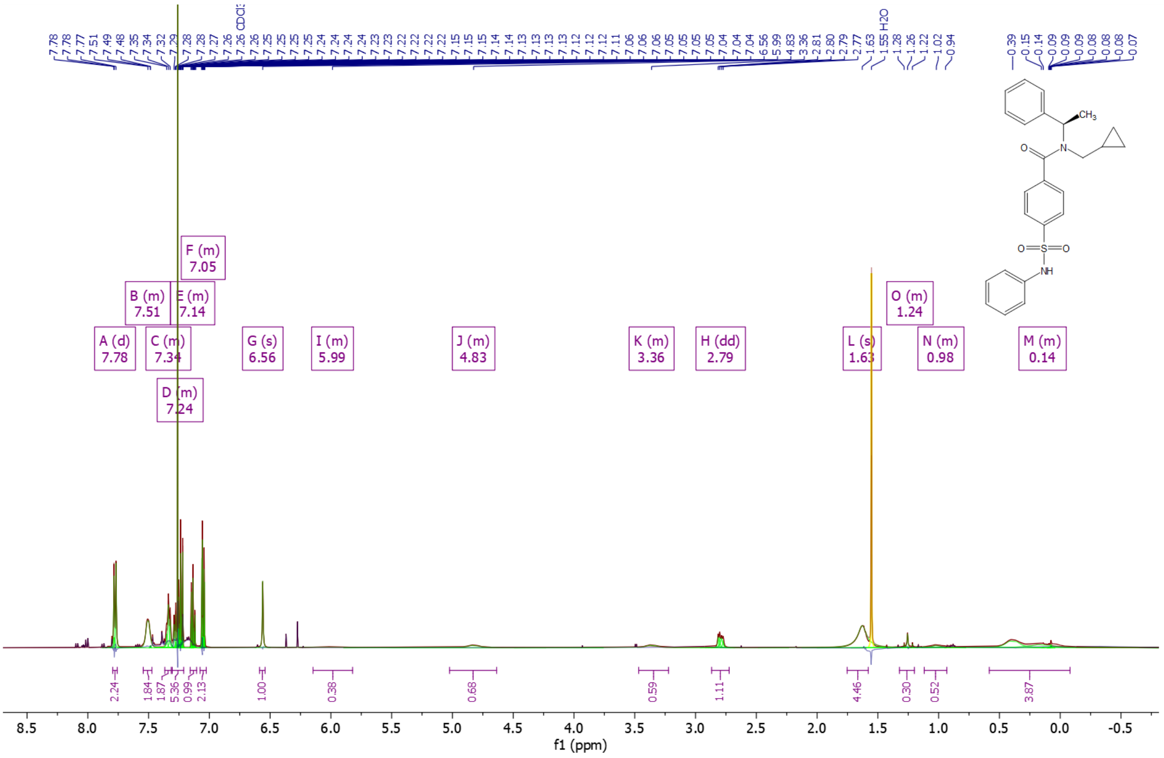

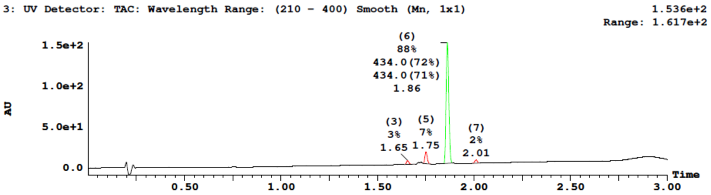

Peak Number	Compound	Time	Area %Total	Mass Found
3		1.65	2.52	Not Found
5		1.75	7.38	Not Found
6	Found	1.86	87.82	)000, 434.0000
7		2.01	2.28	Not Found

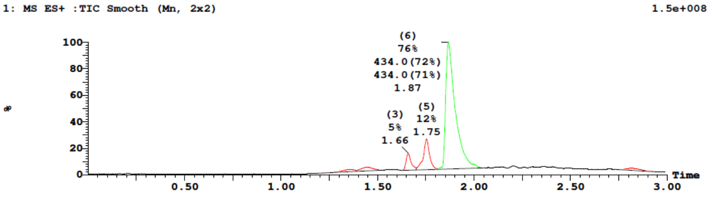

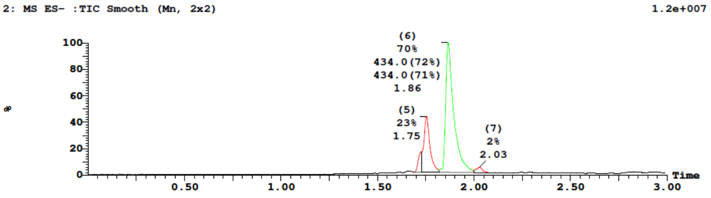

Peak ID	Compound	Time	Mass Found
6	Found	1.87	435

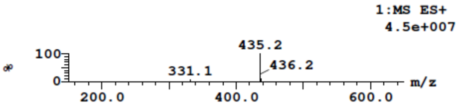

N-(Cyclopropylmethyl)-*N*-((5-fluoropyridin-2-yl)methyl)-4-(*N*-phenylsulfamoyl)benzamide (**71**)

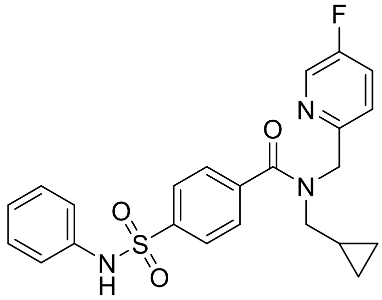

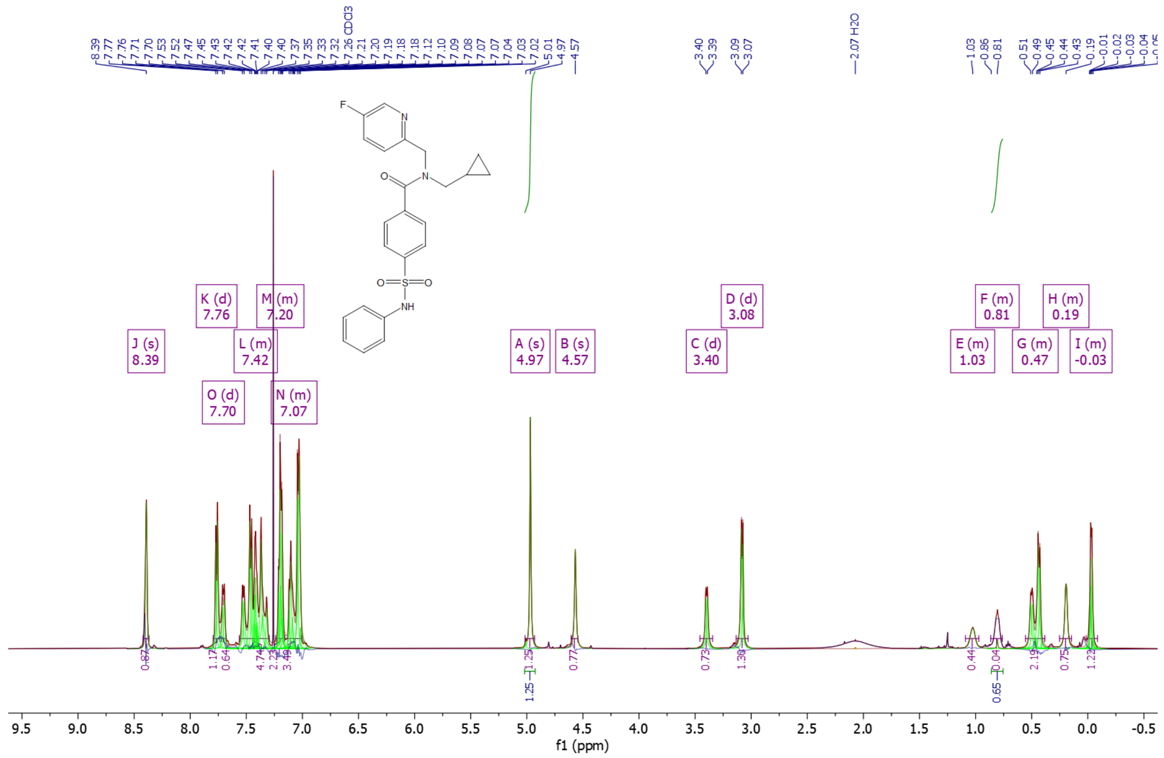

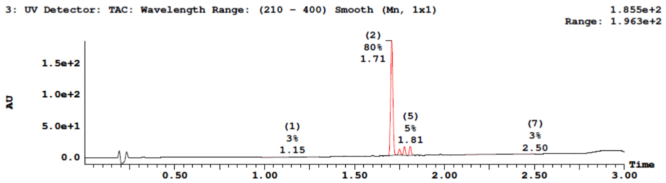

Peak Number	Compound	Time	Area %Total	Mass Found
1		1.15	3.33	Not Found
2		1.71	79.99	Not Found
3		1.75	3.56	Not Found
4		1.78	4.91	Not Found
5		1.81	5.08	Not Found
7		2.50	3.12	Not Found

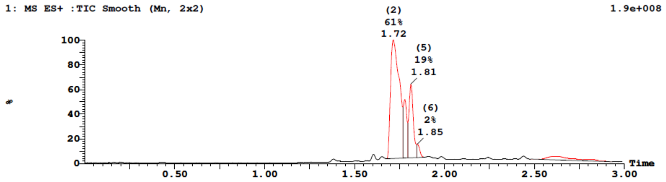

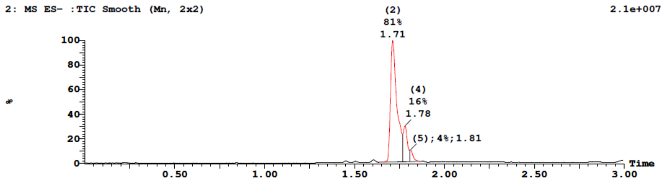

Peak ID	Compound	Time	Mass Found
2		1.72	Not Found

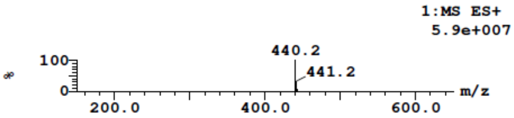

N-[(5-Amino-2-pyridyl)methyl]-*N*-(cyclopropylmethyl)-4-(phenylsulfamoyl)benzamide (**72**)

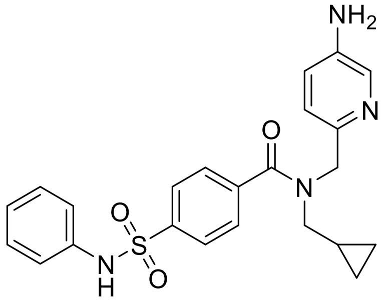

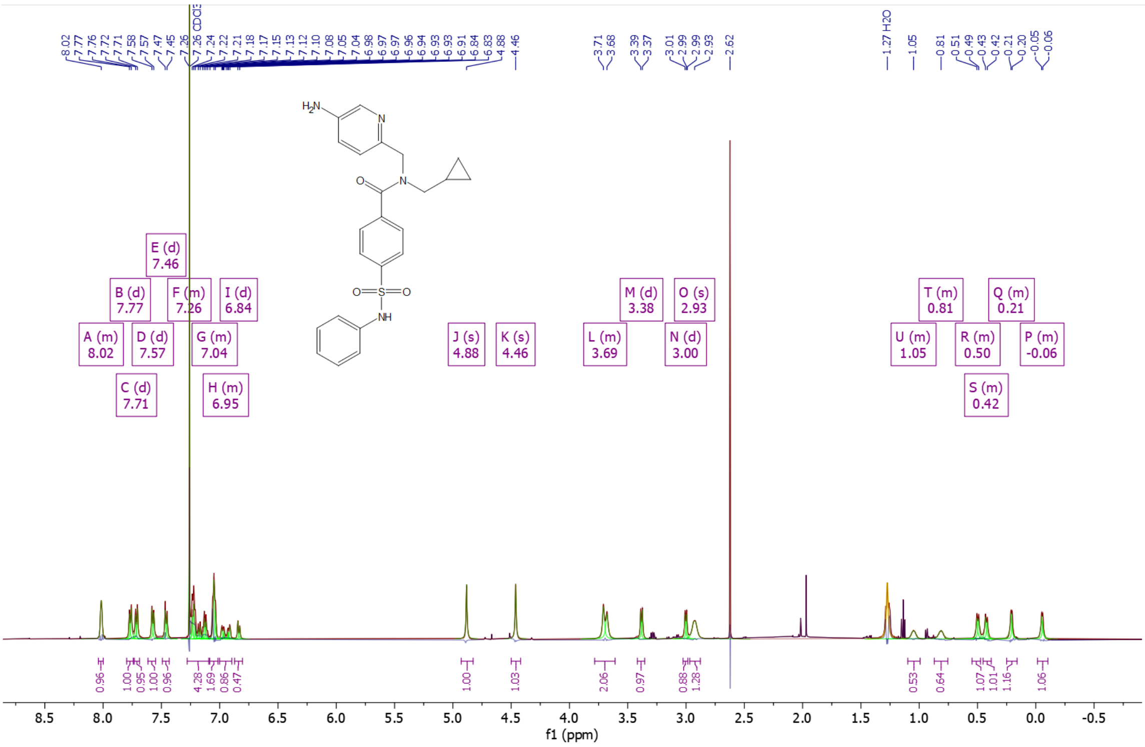

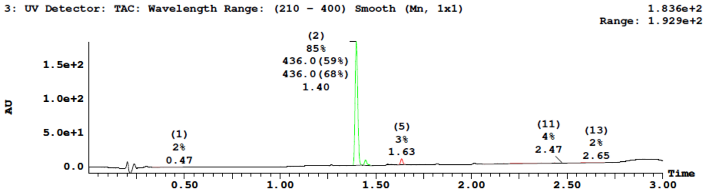

Peak Number	Compound	Time	Area %Total	Mass Found
1		0.47	2.00	Not Found
2	Found	1.40	85.11	)000, 436.0000
3	Found	1.45	3.48	)000, 436.0000
5		1.63	3.42	Not Found
11		2.47	4.18	Not Found
13		2.65	1.81	Not Found

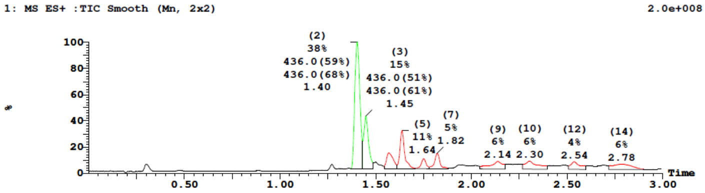

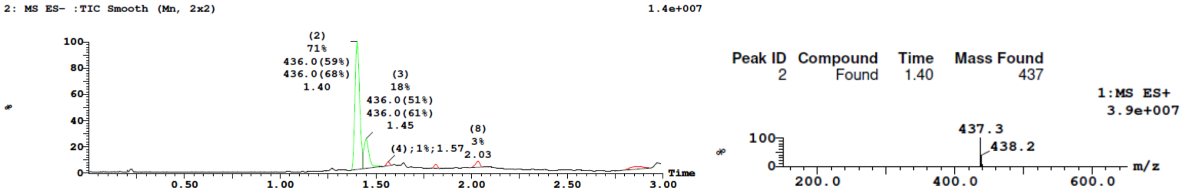

Peak ID	Compound	Time	Mass Found
2	Found	1.40	437

N-(Cyclopropylmethyl)-*N*-[(5-morpholino-2-pyridyl)methyl]-4-(phenylsulfamoyl)benzamide (**73**)

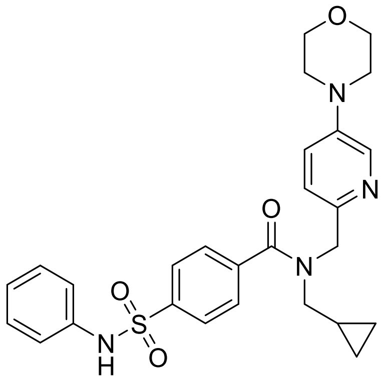

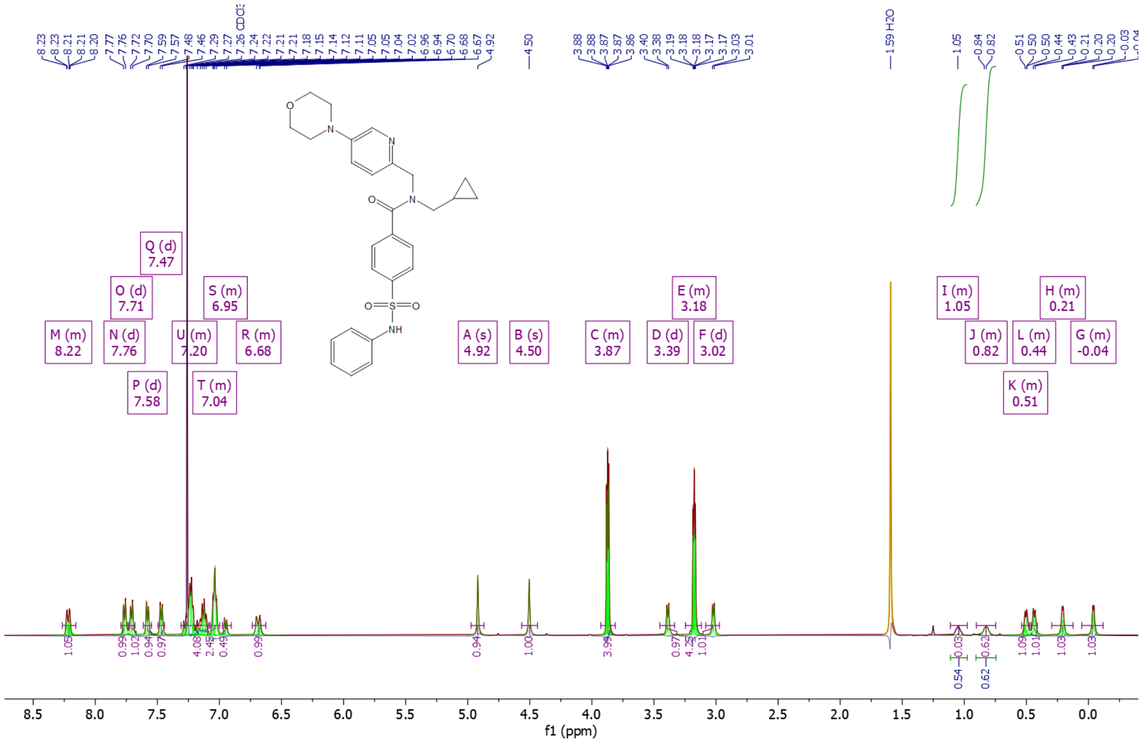

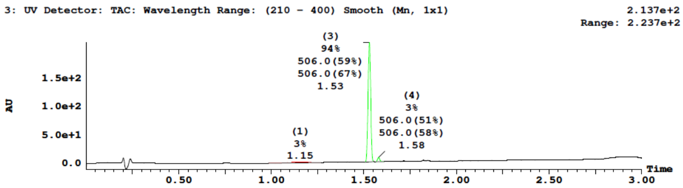

Peak Number	Compound	Time	Area %Total	Mass Found
1		1.15	2.79	Not Found
3	Found	1.53	94.43	)000, 506.0000
4	Found	1.58	2.78	)000, 506.0000

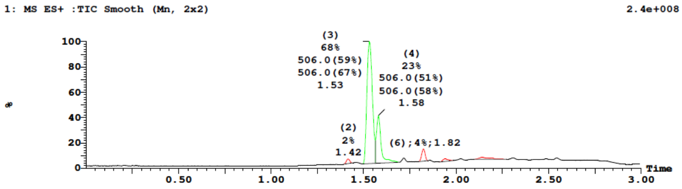

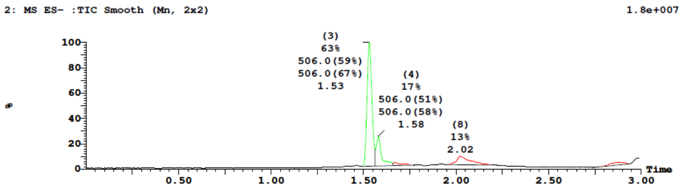

Peak ID	Compound	Time	Mass Found
3	Found	1.53	507

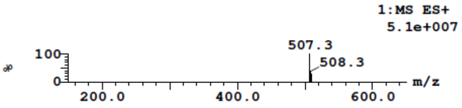

N-(Cyclopropylmethyl)-*N*-[[5-(2-hydroxyethylamino)-2-pyridyl]methyl]-4-(phenylsulfamoyl)benzamide (**74**)

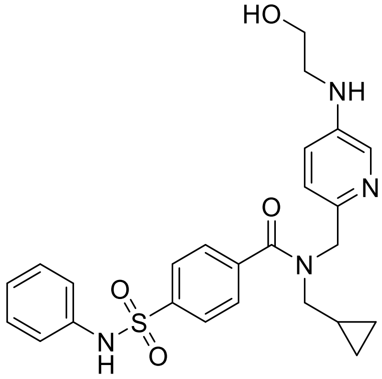

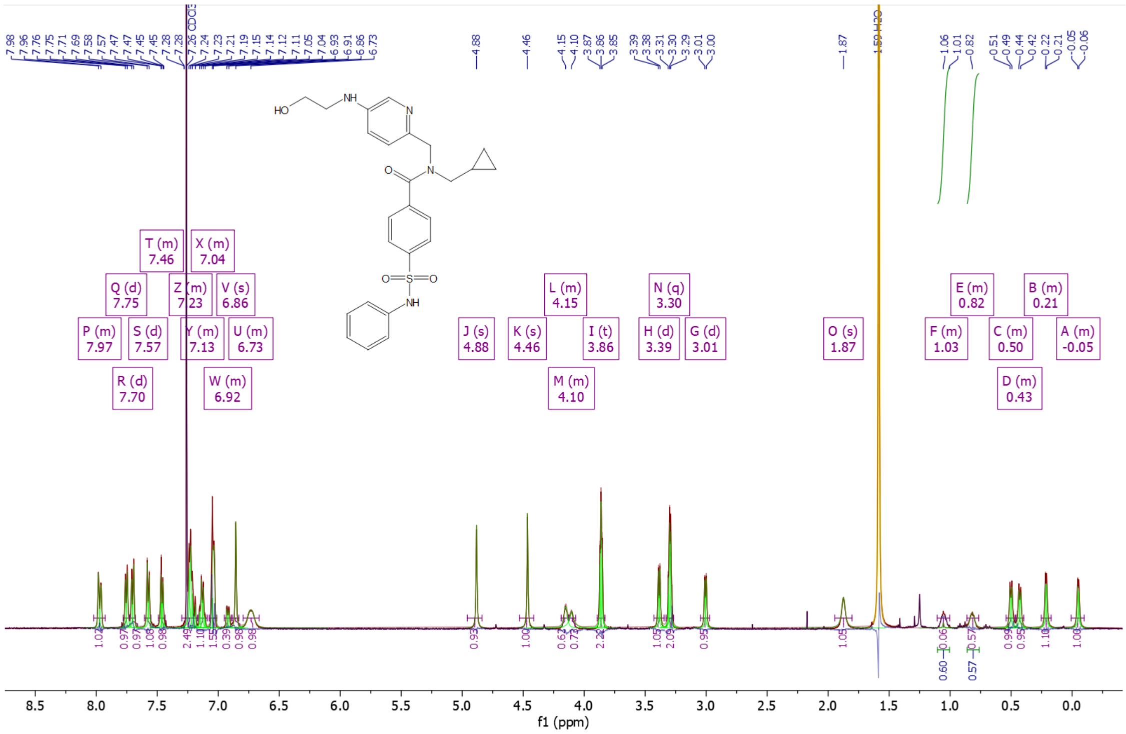

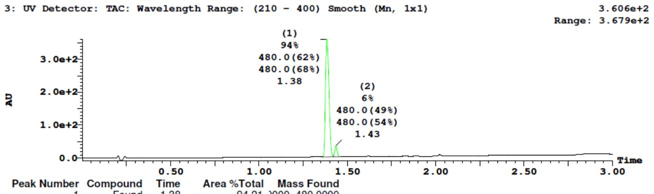

Peak Number	Compound	Time	Area %Total	Mass Found
1	Found	1.38	94.21	)000, 480.0000
2	Found	1.43	5.79	)000, 480.0000

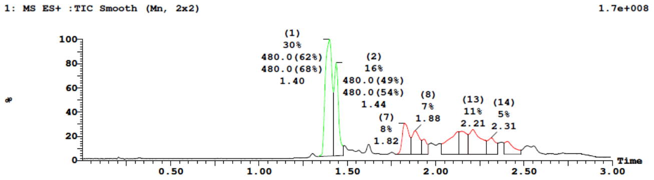

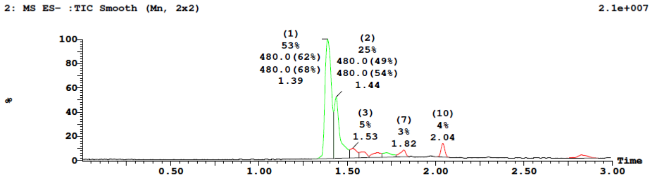

Peak ID	Compound	Time	Mass Found
1	Found	1.40	481

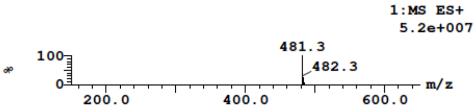

N-(Cyclopropylmethyl)-*N*-[[5-(2-methoxyethylamino)-2-pyridyl]methyl]-4-(phenylsulfamoyl)benzamide (**75**)

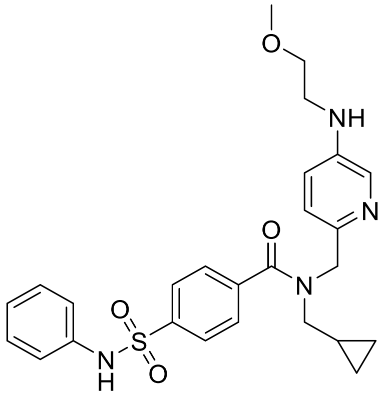

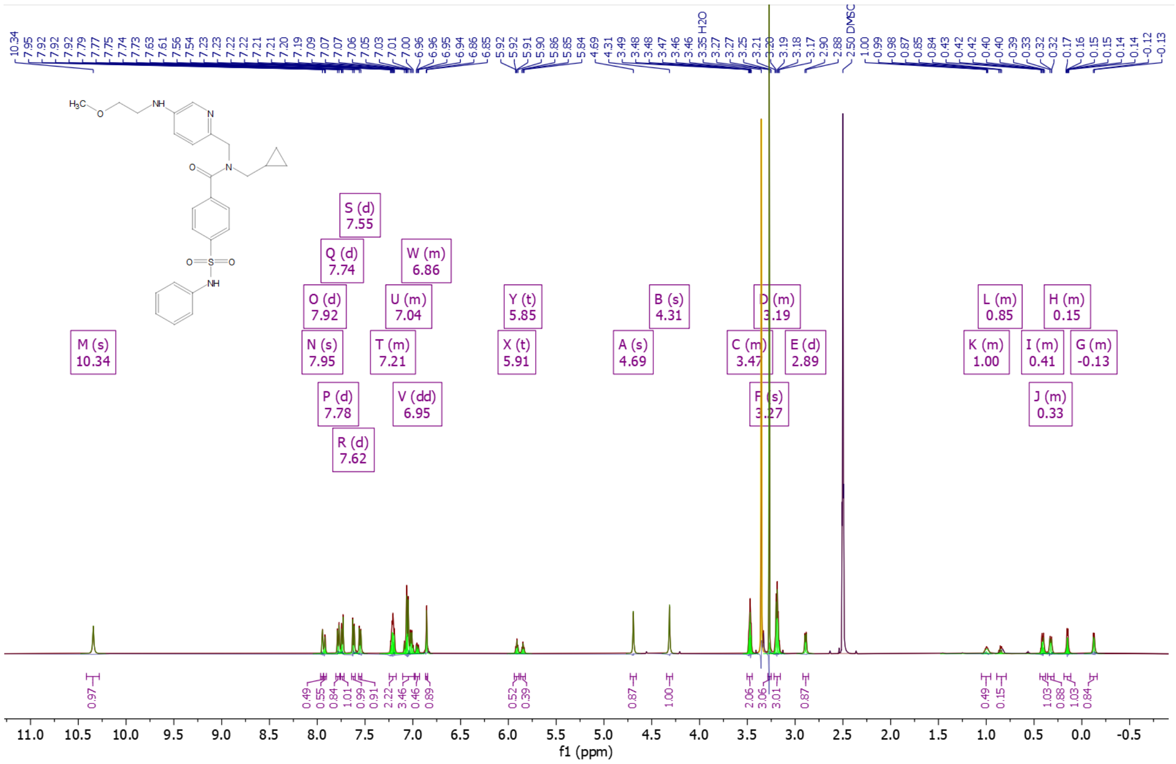

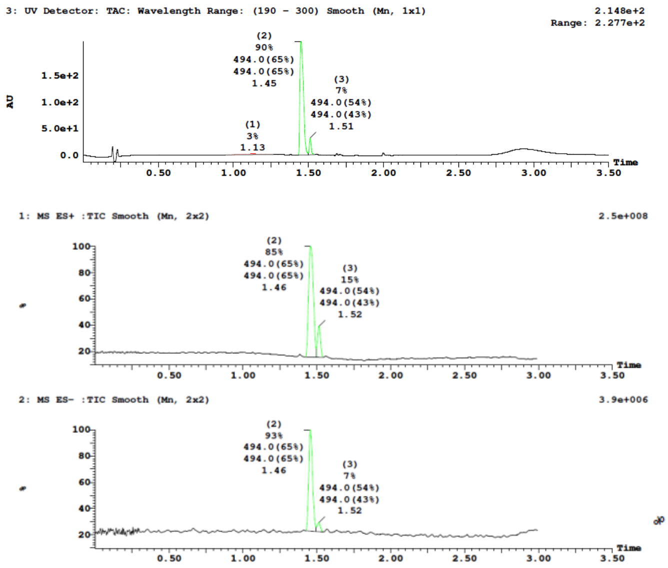

Peak ID	Compound	Time	Mass Found
2	Found	1.46	517,495

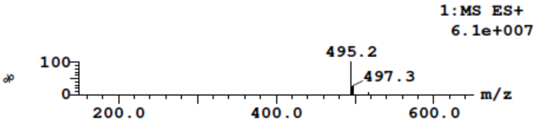

N-(Cyclopropylmethyl)-*N*-[[5-(oxetan-3-ylamino)-2-pyridyl]methyl]-4-(phenylsulfamoyl)benzamide (**76**)

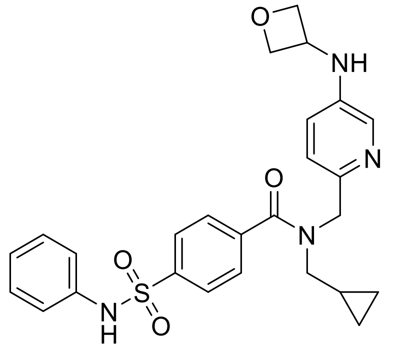

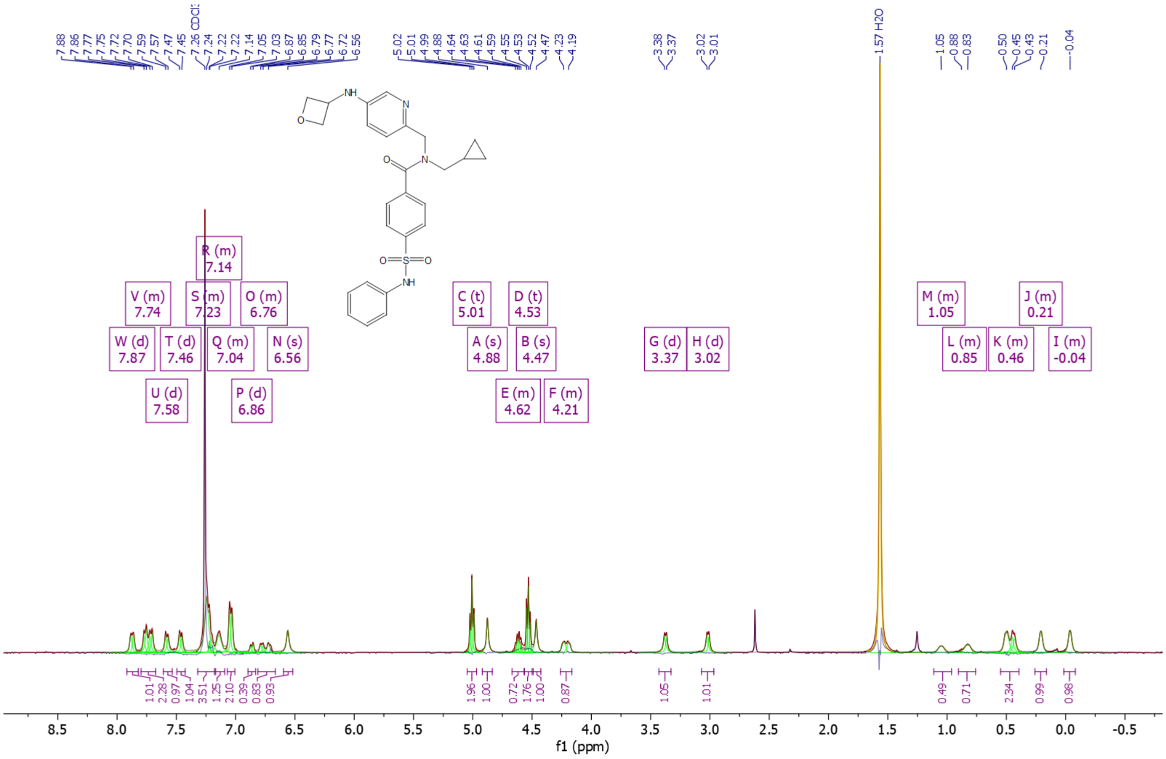

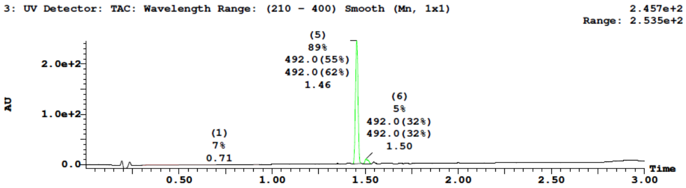

Peak Number	Compound	Time	Area %Total	Mass Found
1		0.71	6.56	Not Found
5	Found	1.46	88.81	[illegible]000, 492.0000
6	Found	1.50	4.62	[illegible]000, 492.0000

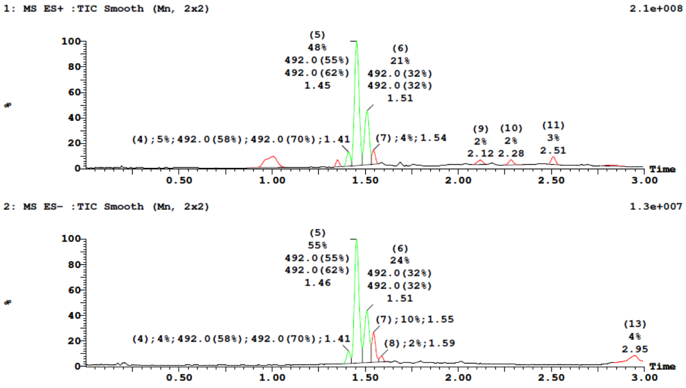

Peak ID	Compound	Time	Mass Found
5	Found	1.45	493

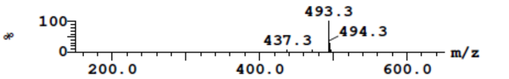

N-(Cyclopropylmethyl)-*N*-[[5-[2-hydroxyethyl(methyl)amino]-2-pyridyl]methyl]-4-(phenylsulfamoyl)benzamide (**77**)

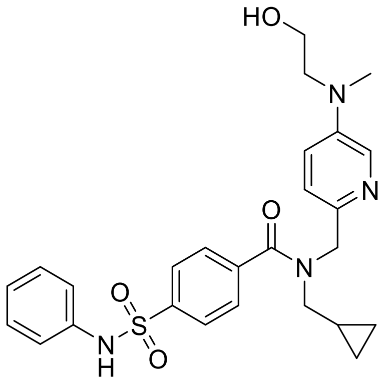

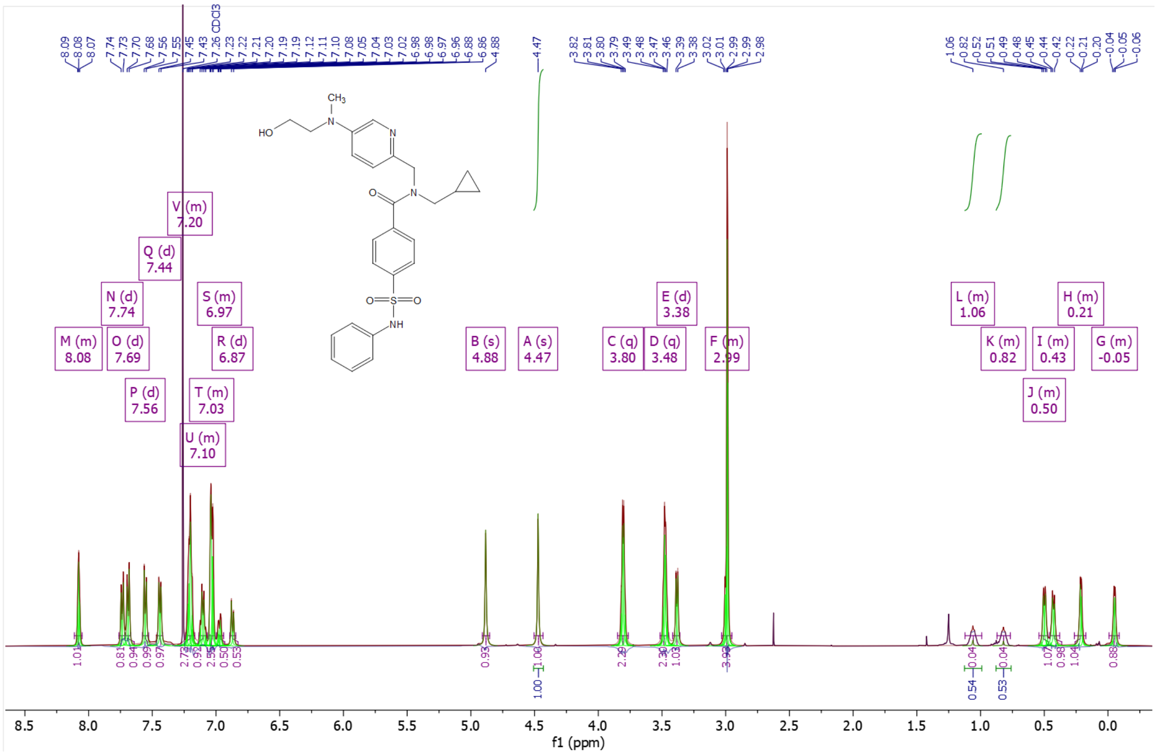

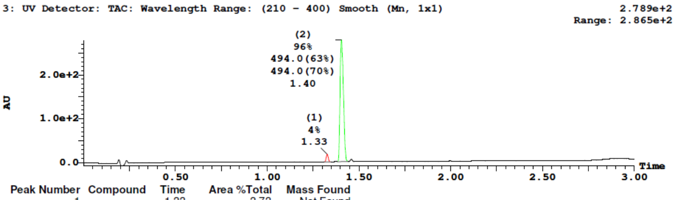

Peak Number	Compound	Time	Area %Total	Mass Found
1		1.33	3.73	Not Found
2	Found	1.40	96.27	)000, 494.0000

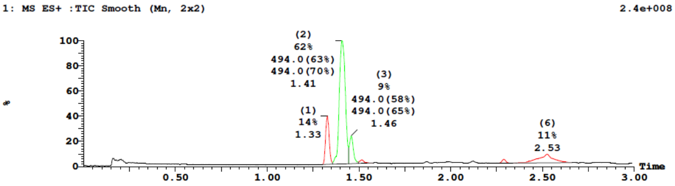

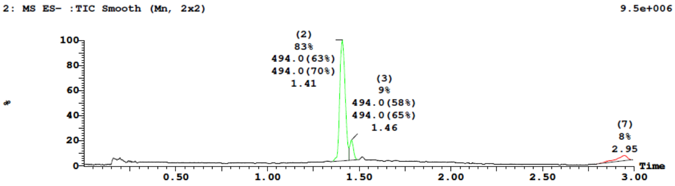

Peak ID	Compound	Time	Mass Found
2	Found	1.41	495

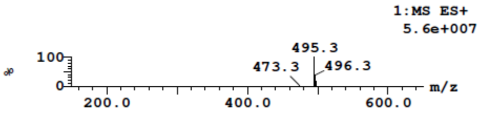

N-(Cyclopropylmethyl)-*N*-[[5-[2-(dimethylamino)ethylamino]-2-pyridyl]methyl]-4-(phenylsulfamoyl)benzamide (**78**)

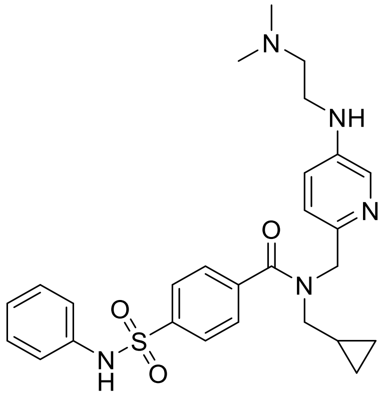

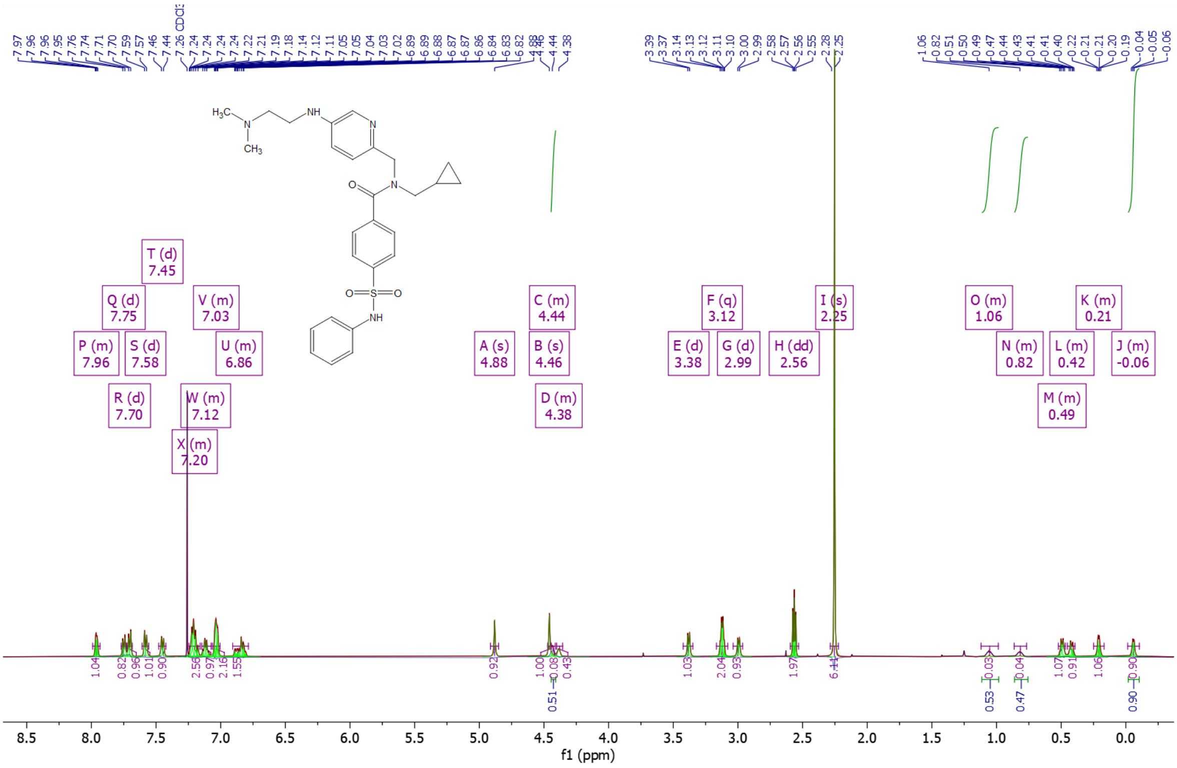

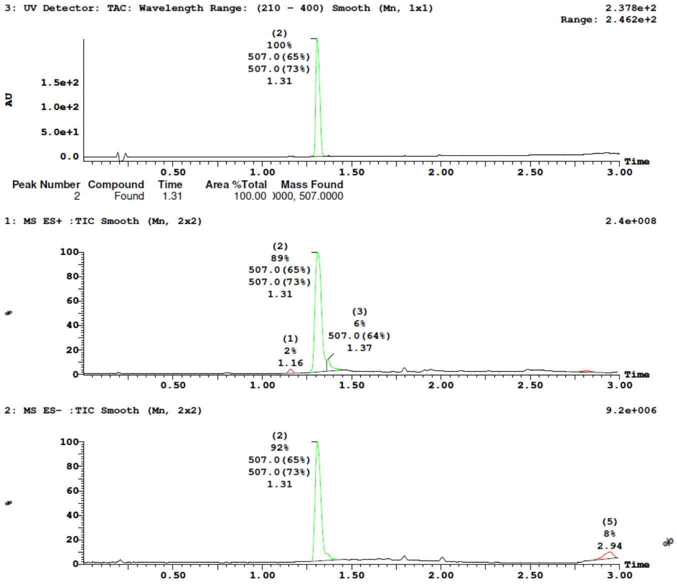

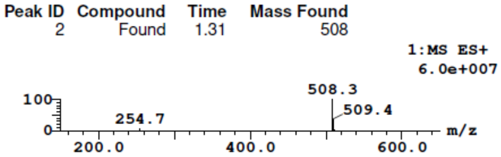

N-(Cyclopropylmethyl)-4-(phenylsulfamoyl)-*N*-[[5-[2-(1*H*-tetrazol-5-yl)ethyl]-2-pyridyl]methyl]benzamide (**79**)

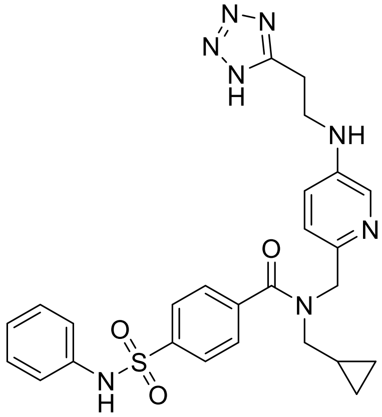

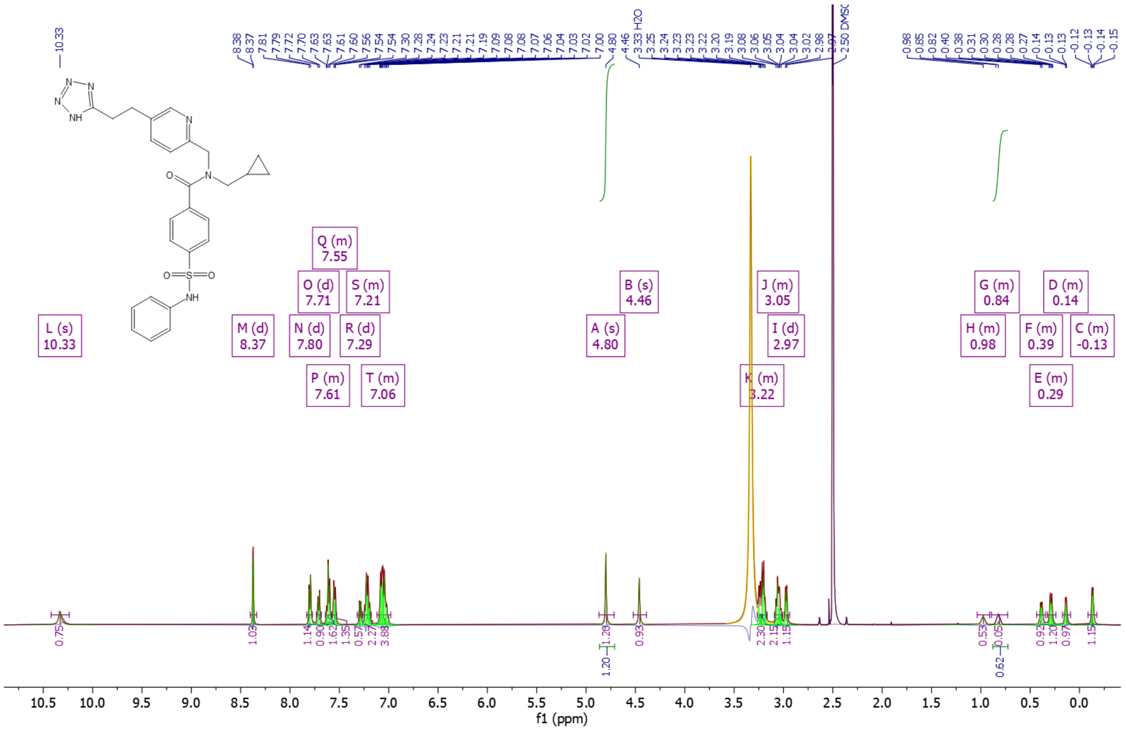

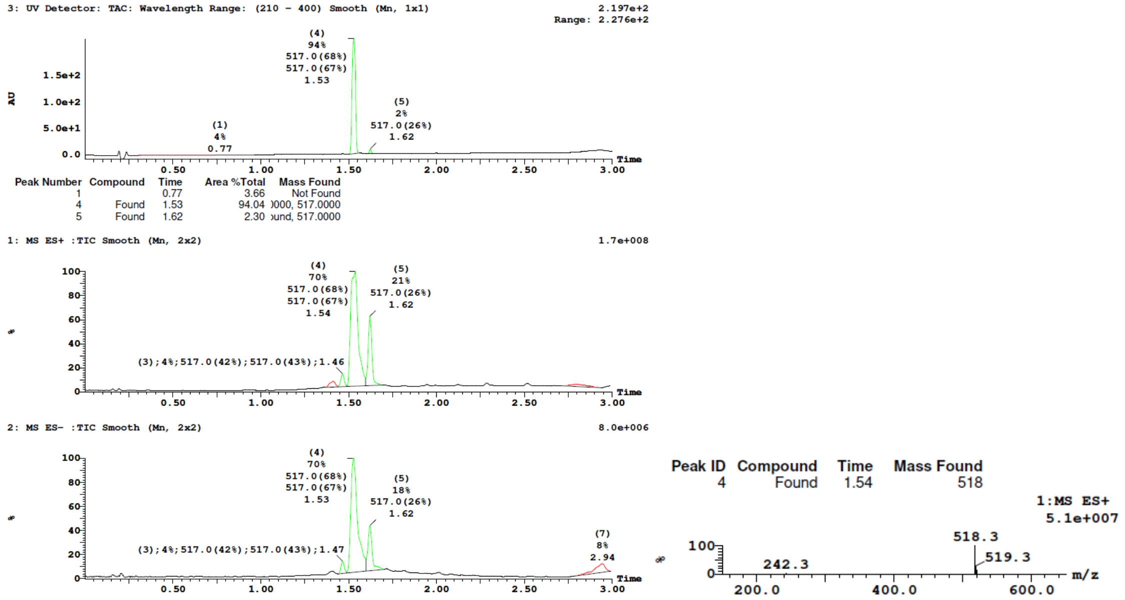

N-Ethyl-*N*-[[5-(2-hydroxyethylamino)-2-pyridyl]methyl]-4-(phenylsulfamoyl)benzamide (**80**)

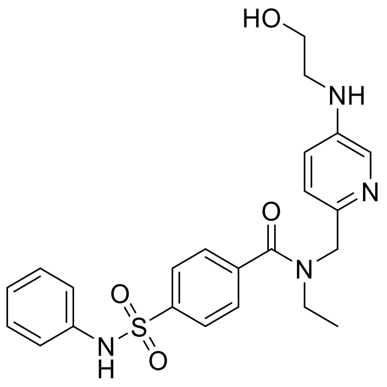

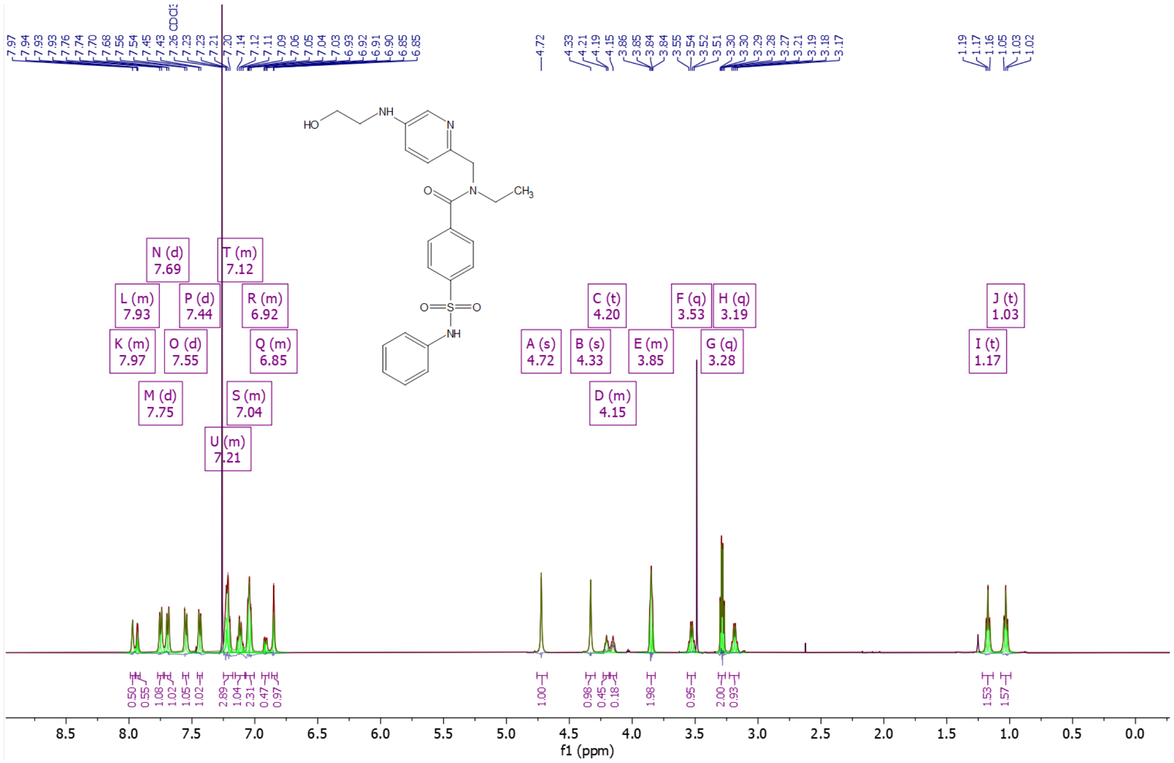

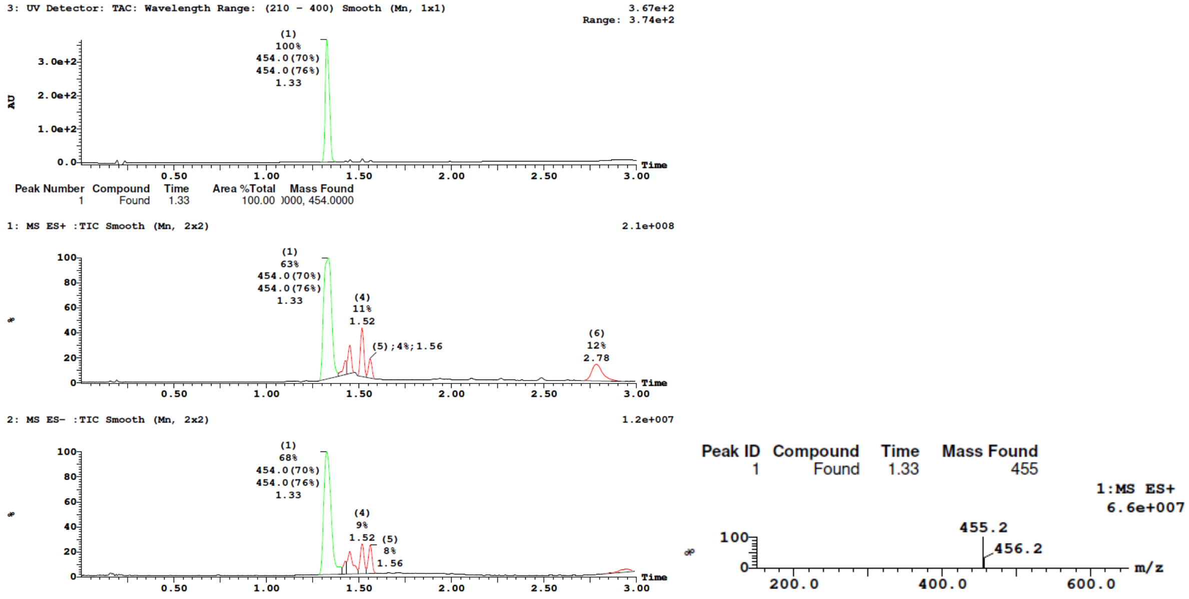

N-(Cyclopropylmethyl)-*N*-[[5-[(2-hydroxy-1,1-dimethyl-ethyl)amino]-2-pyridyl]methyl]-4-(phenylsulfamoyl)benzamide (**81**)

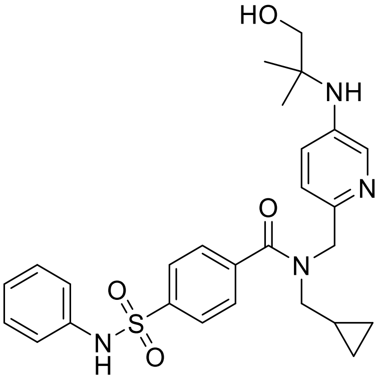

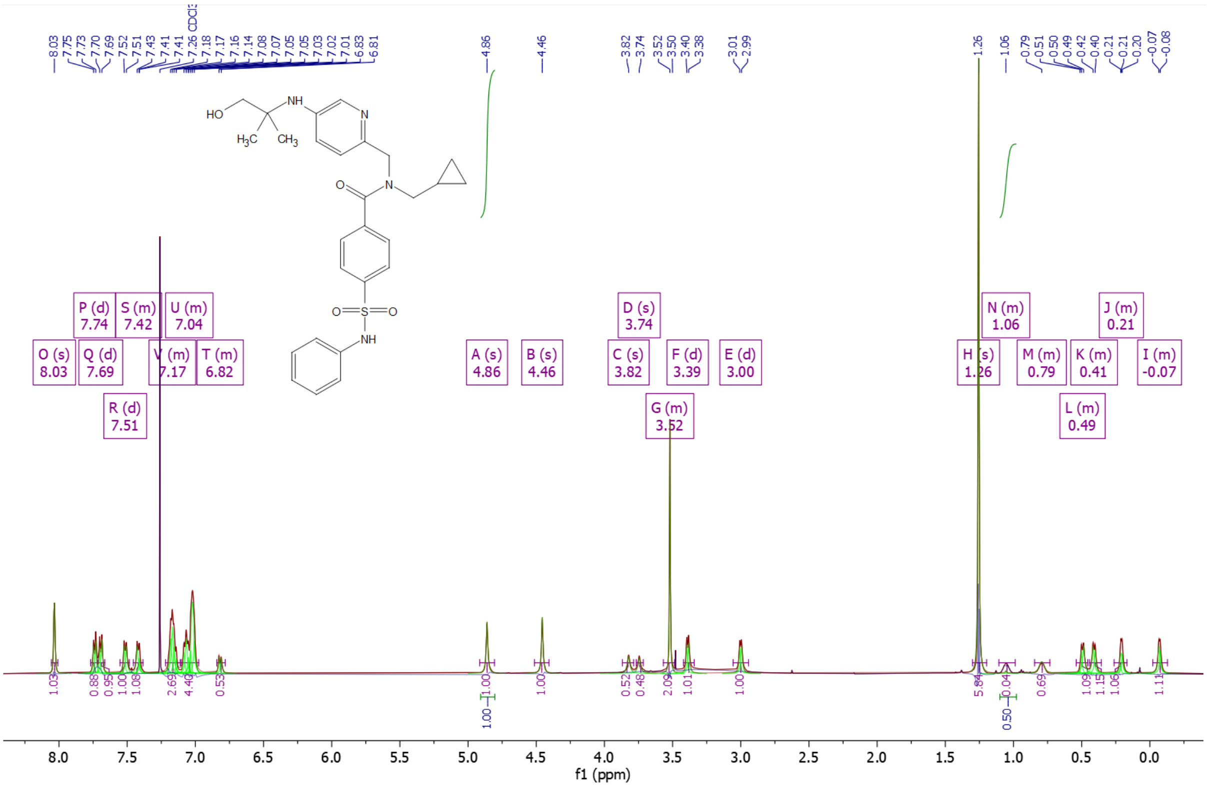

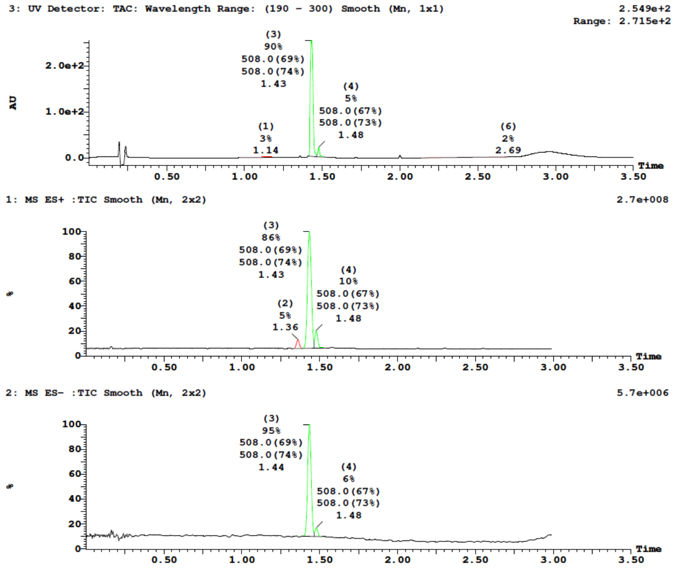

Peak ID	Compound	Time	Mass Found
3	Found	1.43	531,509

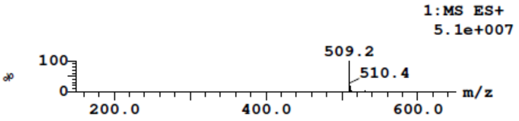

N-(Cyclopropylmethyl)-*N*-[[5-(2-hydroxyethylamino)-6-methyl-2-pyridyl]methyl]-4-(phenylsulfamoyl)benzamide (**82**)

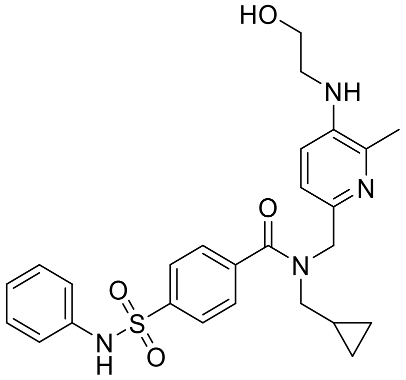

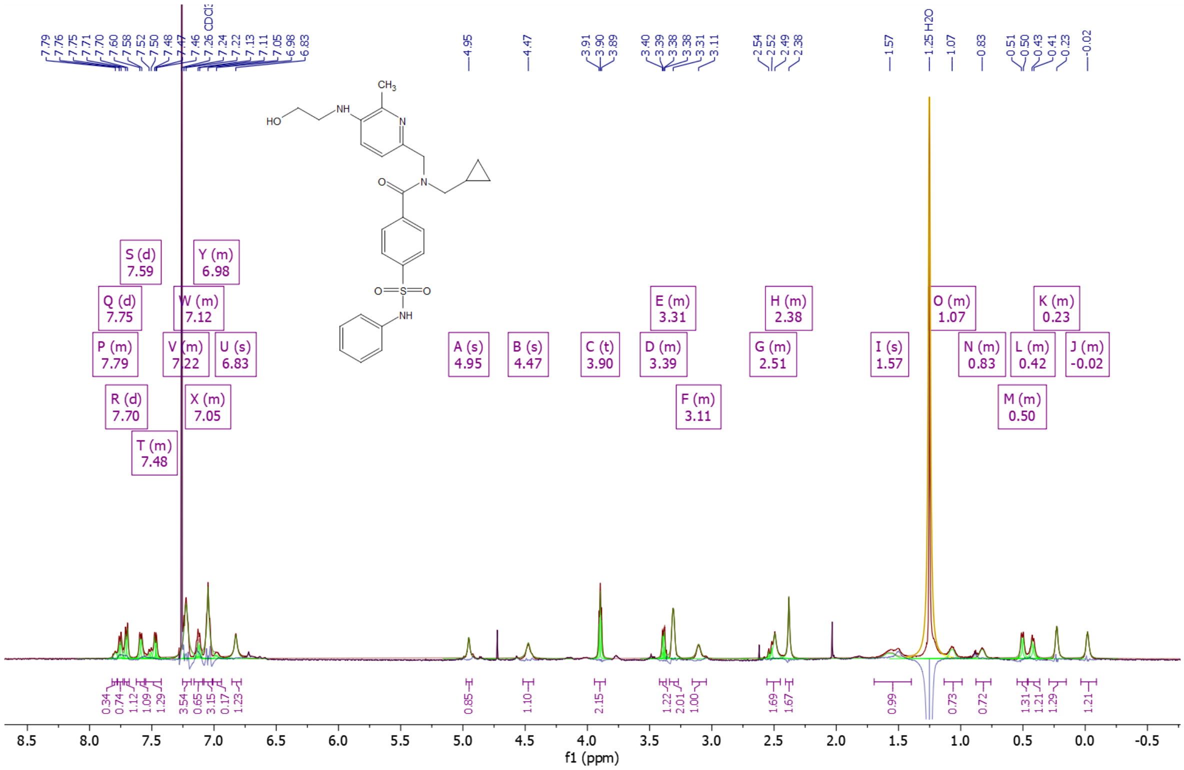

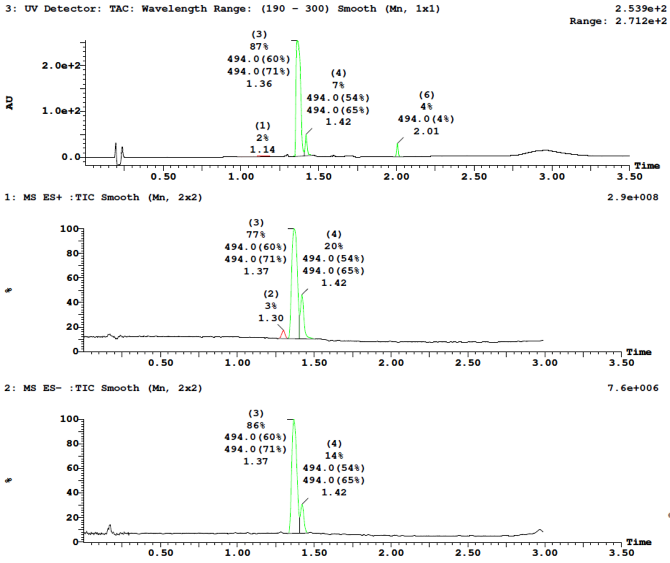

Peak ID	Compound	Time	Mass Found
3	Found	1.37	495

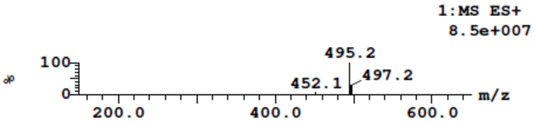

N-(Cyclopropylmethyl)-*N*-[[5-(2-hydroxyethylamino)-4-methyl-2-pyridyl]methyl]-4-(phenylsulfamoyl)benzamide (**83**)

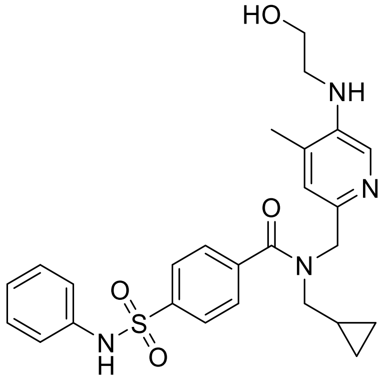

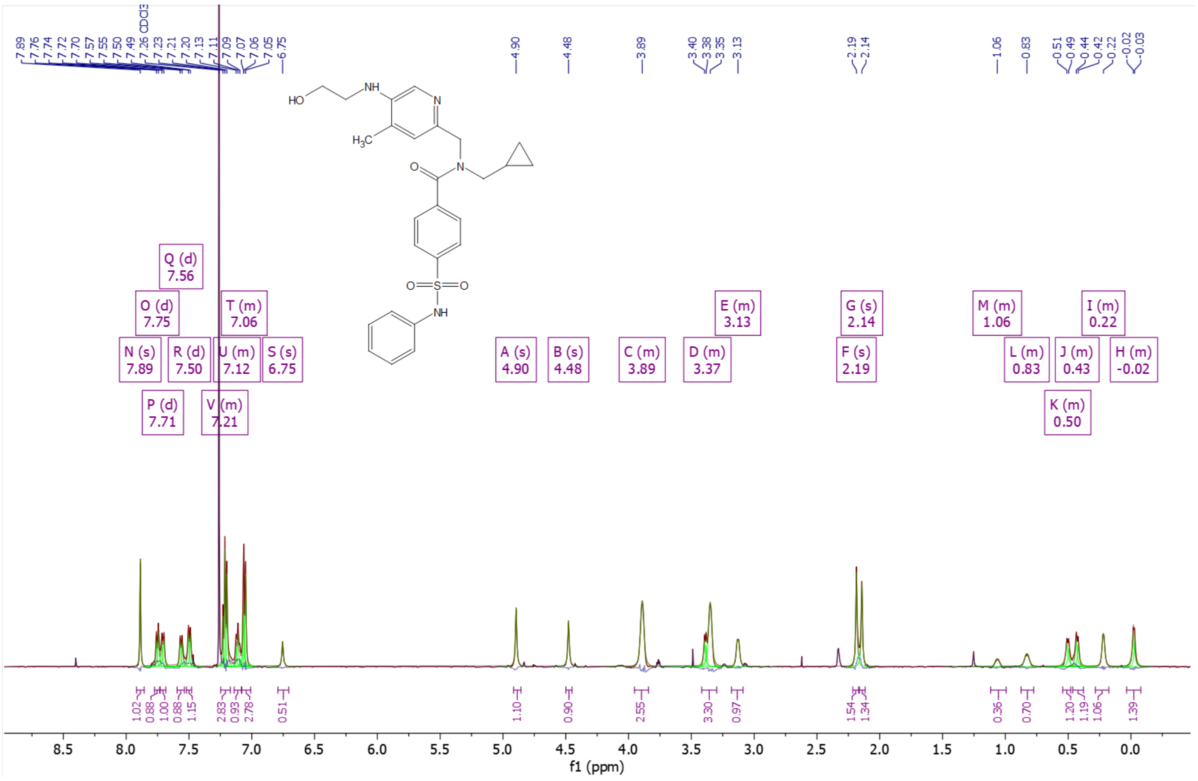

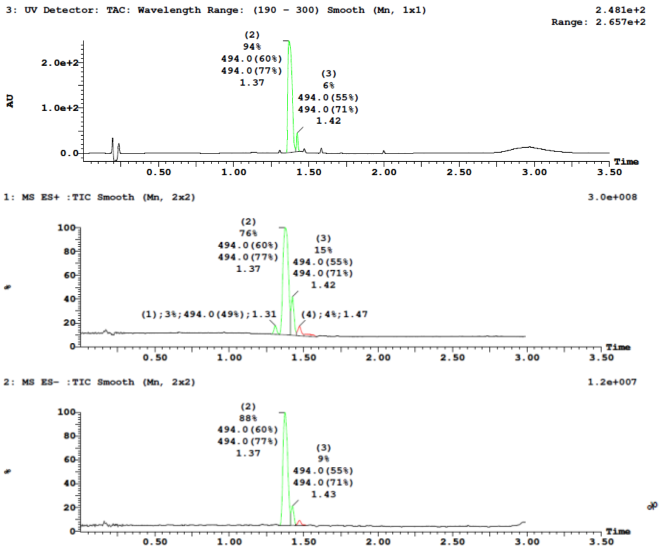

Peak ID	Compound	Time	Mass Found
2	Found	1.37	495

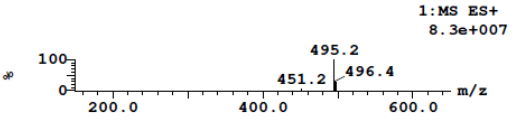

N-(Cyclopropylmethyl)-*N*-[[5-(2-hydroxyethylamino)-2-pyridyl]methyl]-4-[methyl(phenyl)sulfamoyl]benzamide (**84**)

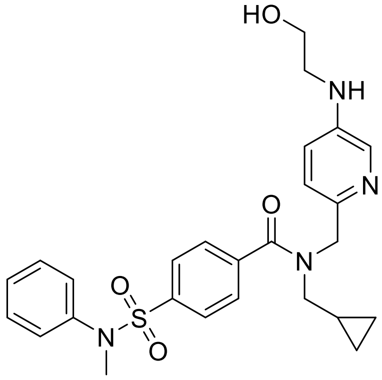

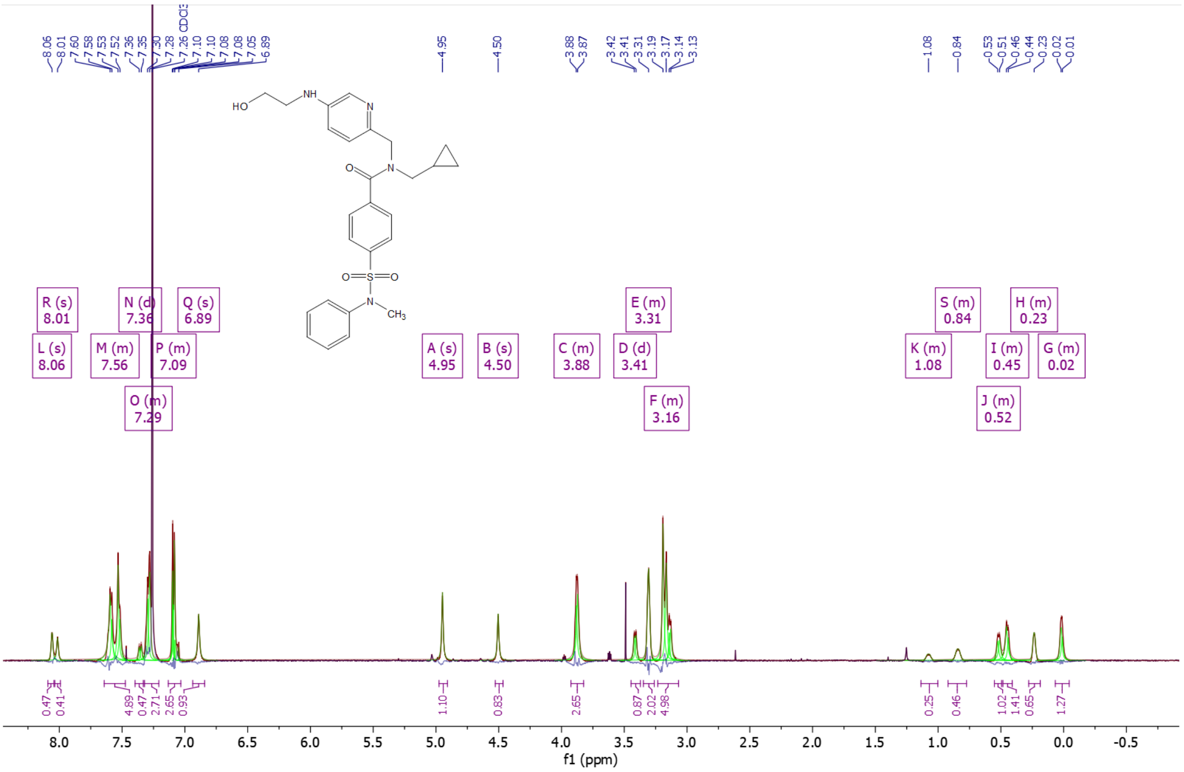

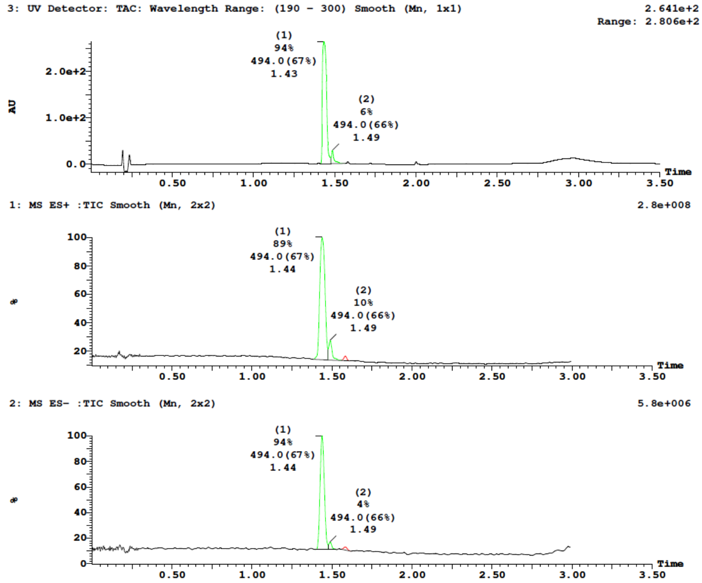

Peak ID	Compound	Time	Mass Found
1	Found	1.44	517,495

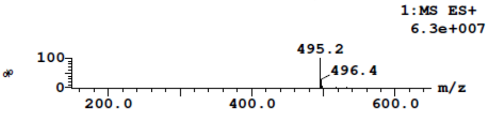

N-(Cyclopropylmethyl)-*N*-[[4-(2-hydroxyethylamino)phenyl]methyl]-4-(phenylsulfamoyl)benzamide (MDI-114215, **85**)

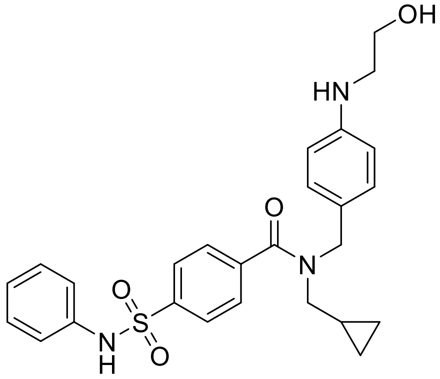

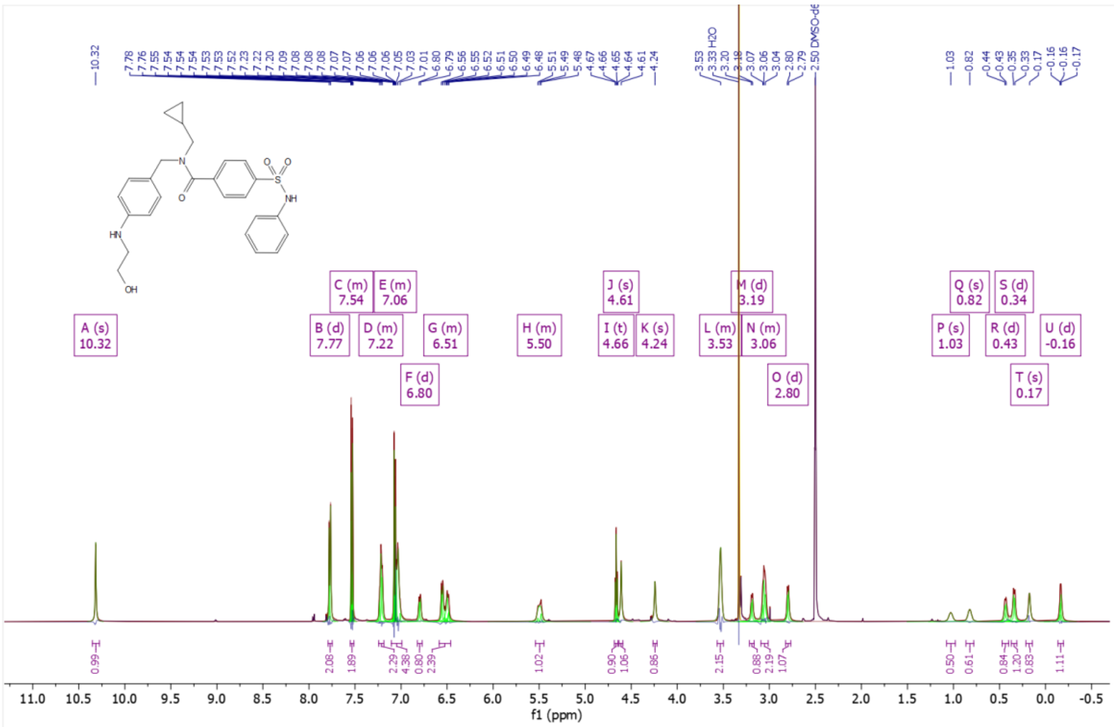

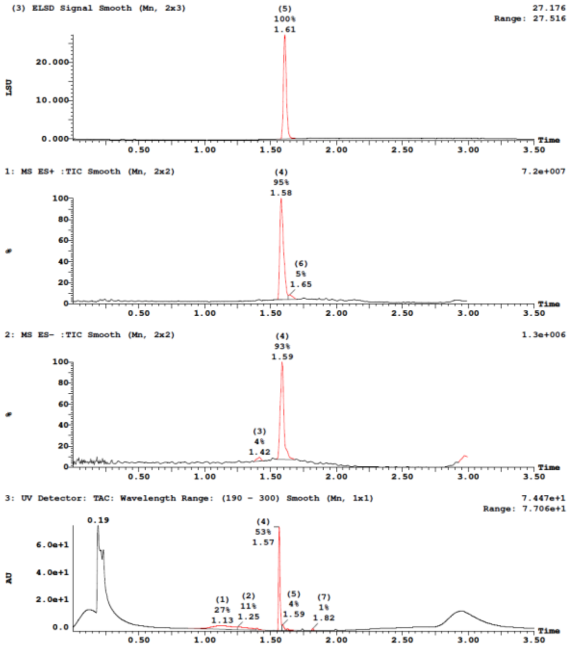

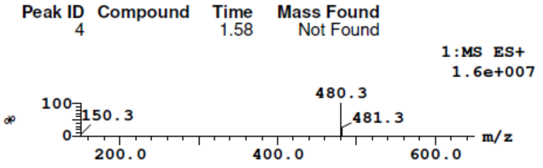

N-(Cyclopropylmethyl)-*N*-[[4-(2-hydroxyethoxy)phenyl]methyl]-4-(phenylsulfamoyl)benzamide (**86**)

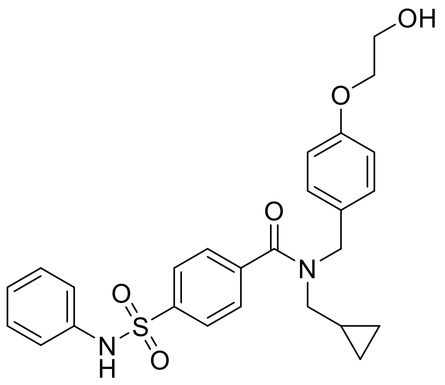

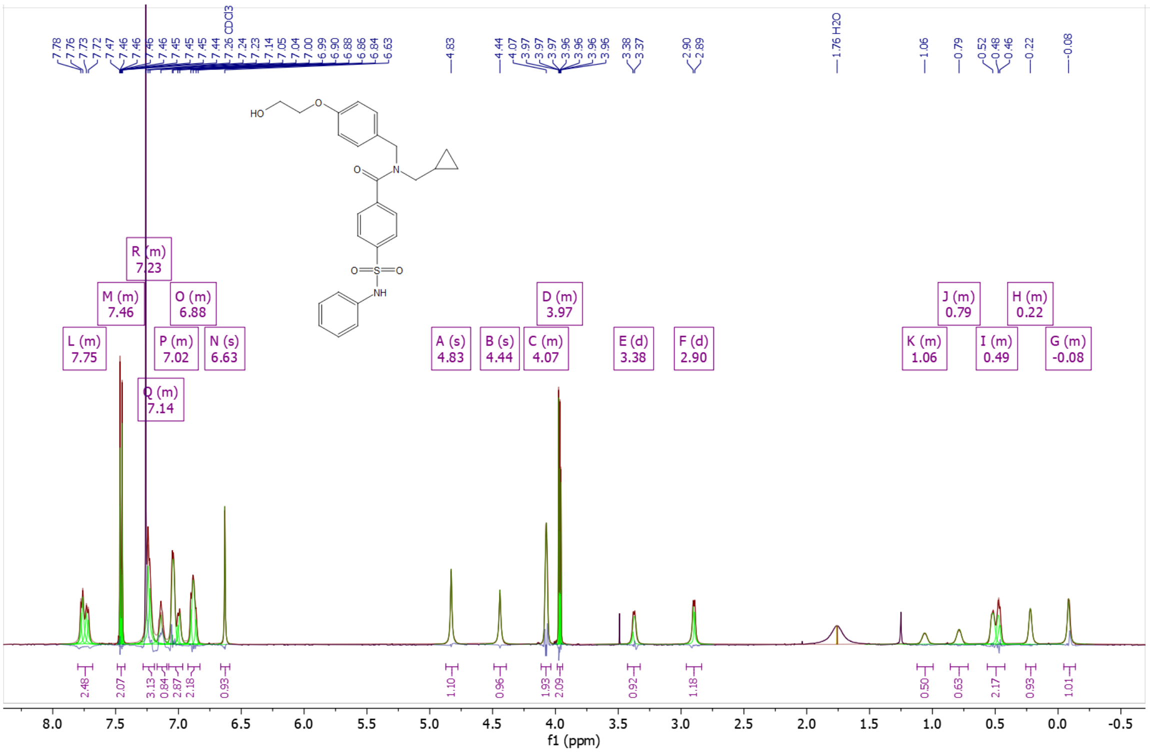

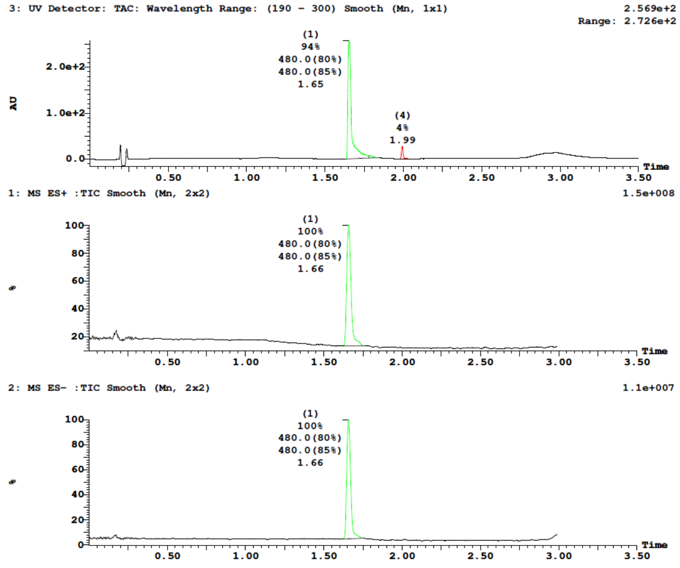

Peak ID	Compound	Time	Mass Found
1	Found	1.66	503,481

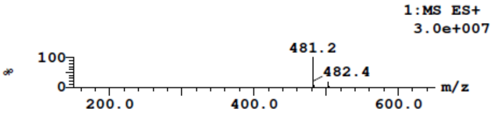

Supplement: Supplementary file 2 — jm4c02694_si_002.pdf [file jm4c02694_si_002.pdf]
